# Rebalance of the Polyamine Metabolism Suppresses Oxidative Stress and Delays Senescence in Nucleus Pulposus Cells

**DOI:** 10.1155/2022/8033353

**Published:** 2022-02-07

**Authors:** Hui Che, Cheng Ma, He Li, Fenglei Yu, Yifan Wei, Hailong Chen, Jun Wu, Yongxin Ren

**Affiliations:** ^1^Department of Orthopaedics, The First Affiliated Hospital of Nanjing Medical University, 210000 Nanjing, China; ^2^Orthopedics and Sports Medicine Center, Suzhou Municipal Hospital (North District), Nanjing Medical University Affiliated Suzhou Hospital, 215006 Suzhou, China; ^3^G.E.R.N. Research Center for Tissue Replacement, Regeneration & Neogenesis, Department of Orthopedics and Trauma Surgery, Faculty of Medicine, Medical Center—Albert-Ludwigs-University of Freiburg, 79085 Freiburg im Breisgau, Germany; ^4^State Key Laboratory of Reproductive Medicine, The Research Center for Bone and Stem Cells, Department of Anatomy, Histology and Embryology, Nanjing Medical University, 210000 Nanjing, China

## Abstract

Intervertebral disk degeneration (IDD) is a major cause of low back pain that becomes a prevalent age-related disease. However, the pathophysiological processes behind IDD are rarely known. Here, we used bioinformatics analysis based on the microarray datasets (GSE34095) to identify the differentially expressed genes (DEGs) as biomarkers and therapeutic targets in degenerated discs. From the previous studies, oxidative stress has been notified as a positive inducement of IDD, which causes DNA damage and accelerates cell senescence. Polyamine oxidase (PAOX), a member of the observed 1057 DEGs, is involved in polyamine metabolism and influences the oxidative balance in cells. However, it is uncertain if the IDD is implicated in the dysregulation of PAOX and polyamine metabolism. This study firstly verified the PAOX upregulation in human degenerated disc samples and applied an IL-1*β*-induced nucleus pulposus (NP) cell degeneration model to demonstrate that spermidine supplementation balanced polyamine metabolism and delayed NP cell senescence. Moreover, we confirmed that spermidine/N-acetylcysteine supplementation or Cdkn2a gene deletion stabilized the polyamine metabolism, suppressed oxidative stress, and therefore delayed the progress of IDD in older mice. Collectively, our study highlights the role of polyamine metabolism in IDD and foresees spermidine would be the advanced therapeutical drug for IDD.

## 1. Introduction

The intervertebral disc is described as a “spinal shock absorber” that helps the spine complete flexion and rotation of the extension side. The nucleus pulposus (NP) is the inner core of the spinal disc, consisting of a jelly-like substance mostly made of water and a loose network of collagen fibres, which permits it to endure compression and torsion forces [1]. However, the microenvironment in NP tissue is increasingly critical beginning with the continuous ageing of the intervertebral disc. NP tissues are more prone to degeneration than other tissues in the body due to their unique physiology of low blood flow, high osmotic pressure, low pH, and low oxygen [2]. Approximately, 40% of populations under 30 years old and up to 90% of those over the age of 55 have mild to severe symptoms in their lumbar spine [3]. In clinical practice, intervertebral disc degeneration (IDD) has been recognized as a major cause of various spinal diseases, e.g., vertebra instability, disc herniation, and spinal stenosis, and low back pain is one of the most common and early symptoms among them [4]. However, most of the current therapies, including physical intervention, medication, and surgery, can only relieve the symptoms but not fundamentally address the pathological process of IDD [5]. Among many causative factors, it has become a consensus that genetic factors play a dominant role in IDD, e.g., cyclin-dependent kinase inhibitor 2A (Cdkn2a) [6], growth differentiation factor 5 (GDF5) [7], and vitamin D receptor [8].

To acquire more differentially expressed gene (DEG) profiles of intervertebral disc diseases, we used gene chip technology and bioinformatics methods to analyze the microarray of the degenerated disc tissues (GSE34095) from the Gene Expression Omnibus (GEO) public repository [9]. The Gene Ontology (GO) analysis and Kyoto Encyclopedia of Genes and Genomes (KEGG) pathway enrichment analyses suggested that the biological implication of DEGs is closely related to DNA damage, cell cycle, and collagen-containing extracellular matrix (ECM) metabolism. Based on our previous studies, excessive accumulation of reactive oxygen species (ROS) leads to a high level of oxidative stress, which causes DNA damage, breaks up the cell cycle, and disorders the ECM metabolism in NP cells [6]. Moreover, the N-acetylcysteine (NAC, an antioxidant) supplementation or Cdkn2a gene deletion can delay NP cell senescence via regulating oxidative stress [10]. Therefore, we screened for oxidative stress-related genes from the observed 1057 DEGs and noticed polyamine oxidase (PAOX) was upregulated in the degenerated disc tissues.

PAOX is one of the catabolic enzymes (another is spermine oxidase) of polyamines (PAs) including putrescine, spermidine, and spermine [11]. In mammalian cells, PAs are naturally occurring polycationic alkylamines, which contribute to the oxidative balance by guarding against ROS-mediated damage and acting as substrates for ROS-producing enzymes both intra- and extracellularly [12, 13]. However, extraordinary activation of PA catabolism is through two linked pathways, one of which is mediated by PAOX resulting in the generation of ROS in the form of H_2_O_2_ [14]. Thus, PAOX-related dysregulation of PA metabolism implies a potential to affect oxidative status. Nonetheless, whether PA metabolism is associated with oxidative stress in IDD has not been reported.

Here, we aim to explore (i) whether PA metabolism participates in the progress of IDD, (ii) whether PA supplementation or PAOX inhibition prevents the oxidative stress and stabilizes the ECM secretory phenotype in NP cells, and (iii) whether NAC supplementation or Cdkn2a gene (encode p16^INK4a^ protein) deletion suppresses oxidative stress and delays IDD via regulating PA metabolism. Collectively, the present study integrated patients' samples, *in vitro* framework, and mouse nature IDD model to investigate the impact of PAOX-mediated PA metabolism in IDD. The findings uncover a novel biomarker and pharmacological strategy in preventing the progress of IDD.

## 2. Materials and Methods

### 2.1. Reagents and Antibodies

DMEM/F-12, penicillin/streptomycin, amphotericin, and fetal bovine serum (FBS) were from Thermo (Massachusetts, USA); collagenase XI, dispase II, IL-1*β*, and diacetyl dichlorofluorescein (DCFH-DA) kit were purchased from Sigma (Ohio, United States); Masson's and Safranin O Stain Kit was from Solarbio (Shanghai, China); anti-collagen II (ab34712), anti-PAOX (ab230441), anti-p16 (ab51243), anti-*β*-actin (ab6276), anti-8-hydroxy-2′-deoxyguanosine (ab48508), anti-gamma H2A.X (ab81299), IgG H&L Alexa Fluor 488 (ab150113), Hydrogen Peroxide Assay Kit (ab138874, ab102500), and Total Polyamine Assay Kit (ab239728) were from Abcam (Cambridge, USA); Trizol reagent and reverse transcriptase kit were from Vazyme (Nanjing, China). SYBR Green Master Mix was from Applied Biosystems (Waltham, USA); Vectastain Elite ABC reagent was obtained from KeyGen (Nanjing, China); spermidine and N-acetylcysteine (NAC) were from Selleck (Shanghai, China); Lipofectamine 2000 transfection reagent (89900) and Protein Extraction Kit (78510) were from Thermo Fisher Scientific (Waltham, USA); ethylenediaminetetraacetic acid (EDTA), paraformaldehyde, and enhanced chemiluminescence (ECL) were from Beyotime (Shanghai, China).

### 2.2. Bioinformatics Analysis of DEGs and Feature Gene Extraction

We retrieved the Affymetrix Human Genome U133A Array (GSE34095) containing three IDD and three normal intervertebral disc tissues from the Gene Expression Omnibus (GEO) public repository (http://www.ncbi.nih.gov/geo). The DEGs between degenerated samples and control samples were generated by an interactive web tool, Affymetrix Expression Console software version 1.4 [15]. A one-way ANOVA was applied on the Robust Multi-array Average expression values to determine whether genes were differentially expressed between the two groups. Genes with an adjusted *F*-statistic *p* value of <0.05 were extracted. Genes with *p* value < 0.05 and log2 fold change (FC) > 1 or <-1 were considered DEGs. To identify gene expression differences, hierarchical clustering analysis of DEGs was performed using Affymetrix Transcriptome Analysis Console software.

### 2.3. KEGG and GO Enrichment Analyses of DEGs

As a knowledge base for systematically visualizing gene functions and biological pathways, we used KEGG with a cut-off *p* < 0.05 to investigate the significantly enriched functions or pathways that are closely related to IDD [16]. Moreover, GO enrichment analyses were used to explore the implication of the DEGs in biological phenomena, including biological process (BP), cellular component (CC), and molecular function (MF). Both KEGG and GO enrichment analyses were performed by using the R package clusterProfiler [17]. *p* value < 0.05 was considered to have statistical significance. The detailed data can be found in the Supplementary file (available here).

### 2.4. Protein-Protein Interaction (PPI) Network Construction

To further clear the functional interactions between proteins analyzed in this study, we built the PPI enrichment network to provide more evidence that PAOX is involved in the pathologic progress of IDD. The Search Tool for the Retrieval of Interacting Genes (STRING) database (http://string-db.org/) was used to generate the network, and a combined score > 0.4 was set as the cut-off criterion. The detailed protein network data (scored links between proteins) can be found in the Supplementary file.

### 2.5. Human Disc Tissue Collection and NP Cell Isolation

To compare the difference between nondegenerated and degenerated disc tissue, we recruited three spine fracture patients without IDD history as the donors of the normal NP tissues, three disc-herniation patients with mild disc degeneration, and three disc-herniation patients with severe disc degeneration. The disc degeneration grade is determined by the Pfirrmann score [18] according to the magnetic resonance imaging (MRI) before surgery, of which grade 1 indicates no degeneration, grade 3 means mild degeneration, and grade 5 means severe degeneration. All patients had surgical indications and signed the informed consent. The disc samples were immediately shipped in a cold DMEM/F-12 medium (Procell, China) from the hospital to the laboratory. This project was implemented following the Declaration of Helsinki [19] and approved by the Ethics Committee of the First Affiliated Hospital of Nanjing Medical University (approval number: 2018-SR-233).

For cell culture, only the NP tissues from grade 1 discs met the nondegeneration criteria and were used for NP cell isolation. Briefly, NP fragments were digested in a mixture of 6 mL culture medium (DMEM/F-12 contains 10% fetal bovine serum, 2% penicillin/streptomycin, and 1% amphotericin), 4 mL collagenase XI, and 2 mL dispase II overnight at 37°C. Afterwards, the cell pellets were filtered, centrifuged, and seeded in a culture flask. The medium was changed every three days, and the cells in the second generation were applied in the experiments.

### 2.6. NP Cell Treatments

To cause a degenerated phenotype of NP cells, cells of 5000/well in 12-well plates were stimulated with IL-1*β* (10 ng/mL) for three days [20]. Besides, NP cells were pretreated with spermidine (50 *μ*mol/L) for 24 hours before the addition of IL-1*β* (10 ng/mL) for another three days [21]. Moreover, PAOX gene-silenced NP cells also got an IL-1*β* (10 ng/mL) stimulation for three days.

Human PAOX siRNA (CAT: SIRGT60880WQ) was purchased from Creative Biolabs (NY, United States) and transfected by Lipofectamine 2000 into NP cells based on the manufacturer's instruction. The transfection efficiency was determined by western blot.

### 2.7. Animal Sources and Treatments

We used Cdkn2a heterozygous mice (a gift from Baojie Li, Shanghai Jiao Tong University) backcrossed with C57BL/6J mice to get wild-type (WT) and Cdkn2a knockout (p16^−/−^) mice in littermates [6]. 15-month-old WT mice were randomly assigned to three groups (*n* = 5 of each): (1) control group with normal drinking water, (2) oral spermidine supplementation group with 25 mg/kg/day spermidine dissolved in the drinking water [22], and (3) oral NAC supplementation group with 150 mg/kg/day NAC dissolved in the drinking water [23]. Meanwhile, the 15-month-old p16^−/−^ mouse group (*n* = 5) was fed with normal drinking water. All drinking water was weighted and refreshed twice a week until the time of sacrifice. All the mice were humanely killed at 18 months old, and the thoracic and lumbar intervertebral discs were collected for testing.

### 2.8. Quantitative Reverse-Transcription Polymerase Chain Reaction (qRT-PCR)

Total RNA was isolated from NP cells (*n* = 5) or mouse disc tissue (*n* = 5) using the Trizol reagent. cDNA was synthesized by a reverse transcriptase kit. All procedures were performed based on the manufacturer's instructions. qRT-PCR was performed using SYBR Green Master Mix and analyzed by a high-throughput real-time PCR system (LightCycler 480, Roche). All the gene expression was normalized by glyceraldehyde phosphate dehydrogenase (GAPDH) and expressed as a fold change compared to the control. The sequence of the primers is listed in Table 1.

### 2.9. Western Blot (WB)

Total proteins from human NP tissue (*n* = 3), NP cells (*n* = 5), and mouse disc tissue (*n* = 5) were isolated with a Protein Extraction Kit. Then, proteins got electrophoresis in the sodium dodecyl sulfate-polyacrylamide gel (SDS-PAGE) and transferred to the polyvinylidene difluoride (PVDF) membrane. Afterwards, the membranes were incubated with the following primary antibodies: collagen II, PAOX, p16, and *β*-actin overnight at 4°C. After being incubated in a secondary antibody for 1 hour, the membranes were then exposed in ECL solution and analyzed by Scion Image Beta 4.02.

### 2.10. Masson's and Safranin O Staining

The human NP tissue (*n* = 3) was fixed in 4% paraformaldehyde (PFA) solution for 24 h. Before embedding in paraffin and sectioning, mouse lumbar needs to be totally decalcified in 10% EDTA solution for 7 days. Afterwards, paraffin-embedded human NP tissues and mouse lumbar were cut into 5 mm thick slices. After deparaffinization and rehydration, the slices underwent Masson's and safranin O staining according to the manufacturer's instructions.

### 2.11. Cellular H_2_O_2_, Polyamine, and ROS Measurement

Quantitative H_2_O_2_ level in human NP cells (*n* = 5) was determined by a Hydrogen Peroxide Assay Kit according to the manufacturer's instructions. The AbGreen reacts with H_2_O_2_ in live NP cells and produces a green fluorescence. Fluorescence intensity was quantified using the Fiji package (https://imagej.net/software/fiji/). Besides, the H_2_O_2_ level in the tissue extracts of mouse disc (*n* = 5) was determined by another Hydrogen Peroxide Assay Kit (ab102500) according to the manufacturer's instructions. Total polyamine in the human NP tissues (*n* = 3) or cells (*n* = 5) was determined by the Total Polyamine Assay Kit. A fluorometric probe (Ex/Em = 535/587 nm) of H_2_O_2_ or polyamine was read out by a microplate reader (BMG LABTECH, Ortenberg, Germany).

The total ROS production in human and mouse NP cells (n =5) was determined using diacetyl dichlorofluorescein (DCFH-DA) staining and analyzed by flow cytometry (ZE5 Cell Analyzer, Bio-Rad, Hercules, USA) at a wavelength of 488 nm based on the manufacturer's instructions.

### 2.12. Microcomputed Tomography (Micro-CT)

Mouse lumbar was fixed with 4% PFA solution for 24 h and scanned by micro-CT (GE Healthcare) at 100 kV and 98 A with a 0.98° rotation between frames. 3D images were reconstructed from 2D and analyzed using SkyScanlgium analysis software.

### 2.13. Immunohistochemistry (IHC), Immunofluorescence (IF), and Hematoxylin and Eosin (H&E) Staining

IHC for the human NP tissue (*n* = 3) and mouse disc (*n* = 5), IF for human NP cells (*n* = 5), and H&E for mouse disc were performed as described previously [6]. The primary antibodies targeting the DNA damage markers are anti-8-hydroxy-2′-deoxyguanosine and anti-gamma H2A.X.

### 2.14. Statistical Analysis

Data are expressed as mean ± standard deviation (SD). Statistical significance was calculated using the Student *t*-test and one-way ANOVA followed by Newman-Keuls multiple comparisons between different groups, and *p* values less than 0.05 were considered as a significant difference.

## 3. Results

### 3.1. Identification of Abnormally DEGs, GO Functional and KEGG Pathway Enrichment Analysis in the Degenerated Disc

**Figure 1:**
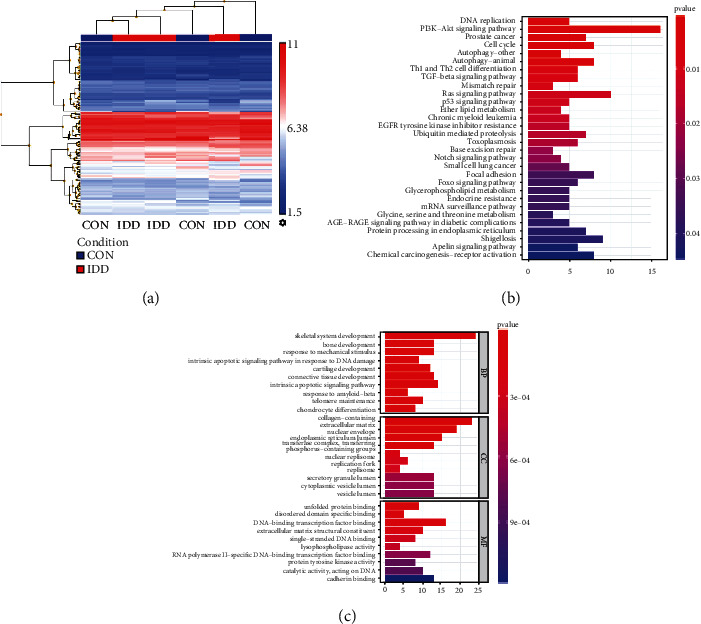
Hierarchical cluster heatmaps of the GSE34095 datasets displaying the (a) 1057 differentially expressed genes (DEGs), (b) KEGG pathways, and (c) GO enrichment analysis of DEGs.

A total of 1057 DEGs (628 up- and 429 downregulation) were obtained in the dataset with a threshold of ∣log2 FC | >1 and *p* < 0.05, which are illustrated with a hierarchical clustering heatmap (Figure1(a), Supplemental Material). Moreover, the top 10 up- and downregulated genes, such as CYP1B1, POSTN, CLIC3, and LOC728392, are given in Table 2. According to the KEGG enrichment analysis, DEGs were mainly enriched in the PI3K-Akt signalling pathway, DNA replication, cell cycle, autophagy, and Ras signalling pathway (Figure1(b)). Besides, we performed the GO functional enrichment analysis regarding BP, CC, and MF to clear the biological roles of these DEGs in degenerated vs. nondegenerated disc samples (Figure1(c)). For BP, DEGs are highly associated with the skeletal system (e.g., bone and cartilage) development, mechanical stimulus, and DNA damage; for CC, DEGs are mainly enriched in collagen-containing ECM, nuclear envelope, and endoplasmic reticulum lumen transferring; for MF, DEGs primarily participate DNA/transcription factor-binding and ECM structural constituent. Collectively, these identified DEGs are frequently mentioned in the ECM (especially collagen) metabolism, DNA replication/damage, and cell cycle, which coincides with our previous research findings that oxidative stress plays a key role in DNA damage, ECM disorder, and cell cycle break-off during IDD development [6]. Therefore, we further screened if some oxidative stress-related genes existed in the 1057 DEGs. As expected, we found significant upregulations of PCYOX1L (prenylcysteine oxidase 1 like), PAOX, MAPK1 (mitogen-activated protein kinase 1), and CTH (cystathionine gamma-lyase) in the degenerated discs (Table 3). As a catabolic enzyme of PAs, PAOX breaks PAs and causes H_2_O_2_-related oxidative damage in many diseases [12]. However, whether the dysregulation of PA metabolism participates in IDD remains unclear.

### 3.2. PAs Decrease and PAOX Increases in Human-Degenerated NP Tissues

**Figure 2:**
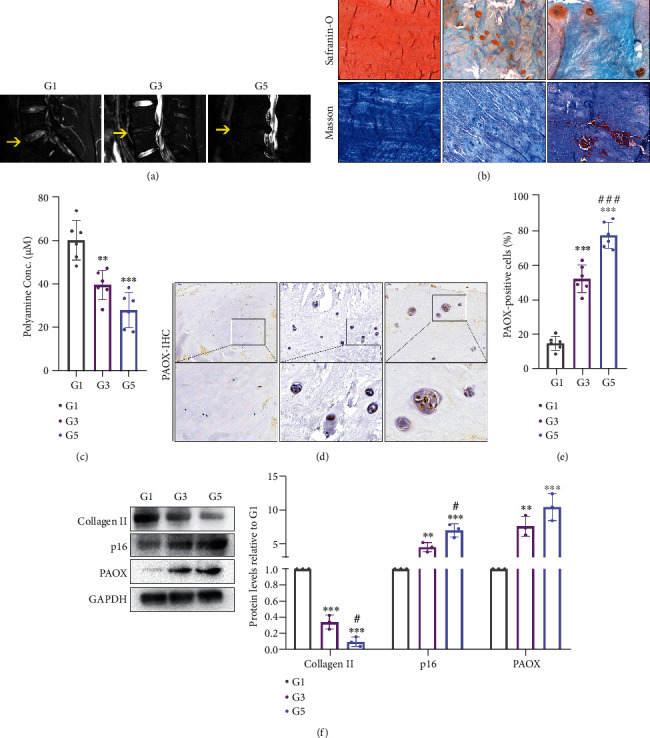
PAs and PAOX expression in NP cells from human intervertebral discs of different degeneration degrees. Representative images of (a) MRI of the disc from patients that yellow arrows refer to the collected segment; (b) safranin O and Masson staining of the NP tissue; (c) total PA concentration in NP tissue (*n* = 6); (d) immunohistochemical staining of PAOX in NP cells and the (e) quantification of positive cell ratio (*n* = 6). (f) Western blotting and measured by densitometry analyses and expressed as folds relative to grade 1 (*n* = 3). Data are presented as mean ± SD; ^∗∗^*p* < 0.01 and ^∗∗∗^*p* < 0.001 compared to G1; ^#^*p* < 0.05 and ^###^*p* < 0.001 compared to G3.

To clear how PAs and PAOX expression change in degenerated NP tissues, we recruited the patients undergoing the discectomy of lumbar surgery in our hospital. The disc samples were divided into three groups based on the degeneration score according to the MRI. As an illustration in Figure 2(a), yellow arrows referred to the operated disc segments and Pfirrmann score: (i) grade I (G1), nondegenerated tissue with the bright signal intensity of water and normal disc height; (ii) grade III (G3), inhomogeneous tissue with a grey signal intensity and a slightly decreased height; (iii) grade V (G5), inhomogeneous tissue with a black signal intensity and a collapsed height. In pathology, the nondegenerated tissue contains a large amount of proteoglycans/collagens, which can be stained in reddish-orange by safranin O-fast green staining and in blue by Masson's trichrome staining. The NP cells in the nondegenerated tissue are small and separated from each other. In contrast, the degenerated tissue presents more fibrin than proteoglycans, which can be stained in blue by safranin O-fast green staining and in red by Masson's trichrome staining. Moreover, NP cells turned out to be larger, vacuolated, and even nested aggregated in the severely degenerated phase (Figure 2(b)). As the degeneration got much more severe, the collagen II synthesis was significantly reduced, and the cell senescence marker p16 was increased (Figure2(f)). Importantly, the total PAs in the NP tissue were reduced from G1 to G3, but no difference was noticed between G3 and G5 (Figure 2(c)). The amount of PAOX-positive NP cells was gradually increased from G1, G3 to G5 (Figures 2(d) and 2(e)). However, the PAOX protein was only increased from G1 to G3, but not changed from G3 to G5 (Figure 2(f)). In general, we found fewer PAs and more PAOX expression in the degenerated NP tissues than in the normal condition.

### 3.3. Rebalance of PA Metabolism Suppresses Oxidative Stress and DNA Damage in Human NP Cells

**Figure 3:**
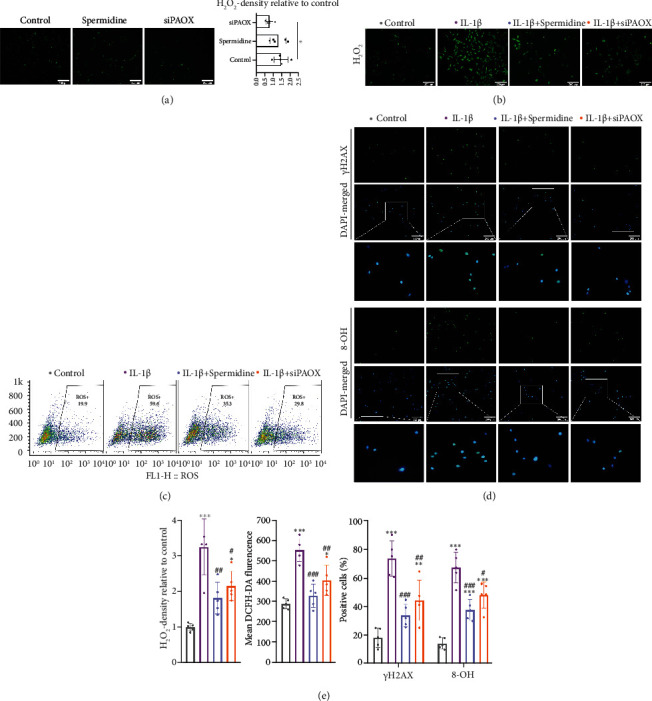
PA metabolism affects oxidative stress and DNA damage in NP cells. Representative images of (a, b) H_2_O_2_ staining, (c) flow cytometry of total ROS, and (d) immunofluorescence staining of *γ*H2AX and 8-OH in NP cells; (e) quantitation of H_2_O_2_, ROS, *γ*H2AX, and 8-OH levels. Data are presented as mean ± SD, *n* = 5; ^∗^*p* < 0.05, ^∗∗^*p* < 0.01, and ^∗∗∗^*p* < 0.001 compared to control, ^#^*p* < 0.05, ^##^*p* < 0.01, and ^###^*p* < 0.01 compared to IL-1*β* treatment.

After confirming the differences of PAs and PAOX in the degenerated vs. nongenerated NP tissue, we further tested whether the dysregulation of PA metabolism triggers oxidative stress and DNA damage *in vitro*-cultured NP cells. We achieved degenerated human NP cells by IL-1*β* stimuli for three days. Meanwhile, cells without any intervention were classified as control, and the spermidine-pretreated or PAOX gene-silenced NP cells were also cultured with IL-1*β* for the same time. As H_2_O_2_ is the main metabolite of polyamine metabolism, the H_2_O_2_ content was measured after three days' culture. Before IL-1*β* stimulation, spermidine pretreatment did not affect the cellular H_2_O_2_ expression, but the silencing of PAOX significantly suppressed the H_2_O_2_ level (Figure 3(a)). After adding IL-1*β*, more H_2_O_2_ was accumulated compared to the control, and spermidine pretreatment or PAOX gene silencing somehow prevented the H_2_O_2_ production (Figures 3(b) and 3(e)). Moreover, the total ROS level was also distinctly triggered by IL-1*β*, which was partially rejected by the spermidine supplement or PAOX gene knockdown (Figures 3(c) and 3(e)). Furthermore, we discerned the DNA damage in NP cells by staining the sensitive markers *γ*H2AX and 8-OH expressing in the nucleus and found the positive cells were both increased after IL-1*β* treatment, which was restrained by spermidine intake or PAOX silencing (Figure 3(e)). Therefore, PA metabolism was disrupted in the inflammatory-caused NP cell degeneration, and the overproduction of H_2_O_2_ is one of the sources of cellular ROS accumulation and DNA damage, which can be alleviated through the rebalance of PA metabolism by either spermidine supplement or PAOX suppression.

### 3.4. Rebalance of PA Metabolism Delays Human NP Cell Senescence and ECM Degradation

**Figure 4:**
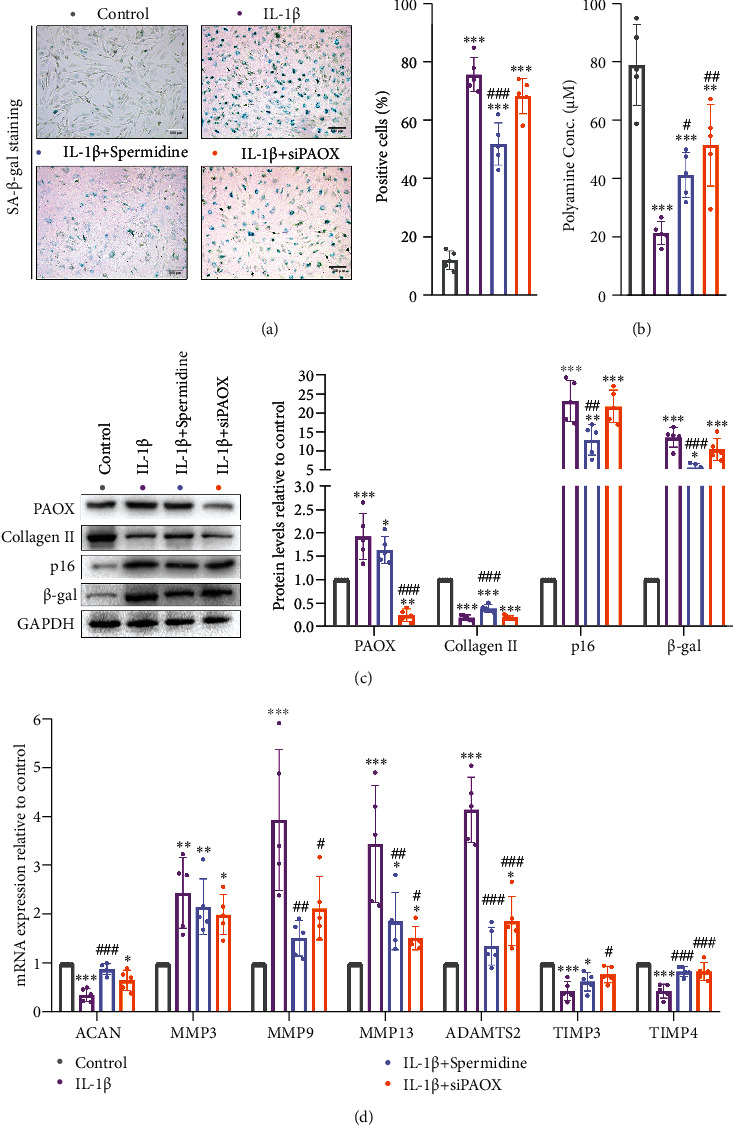
PA metabolism affects senescence and ECM stabilization. (a) SA-*β*-gal staining and quantification of positive cell ratio; (b) total PA concentration in NP cells; (c) western blotting and measured by densitometry analyses and expressed as folds relative to control; (d) target mRNA expression assessed by RT-PCR. Data are presented as mean ± SD, *n* = 5; ^∗^*p* < 0.05, ^∗∗^*p* < 0.01, and ^∗∗∗^*p* < 0.001 compared to control, ^#^*p* < 0.05, ^##^*p* < 0.01, and ^###^*p* < 0.001 compared to IL-1*β* treatment.

Additionally, we explored whether the unbalanced PA metabolism involves the NP cell senescence and the disorder of ECM secretory phenotype. Compared to control, large numbers of NP cells turned out to be senescent after IL-1*β* stimuli, which can be alleviated by spermidine pretreatment. However, PAOX silencing did not significantly rescue NP cells from the ageing process (Figure 4(a)). Interestingly, spermidine supplementation and PAOX silencing both inhibited the IL-1*β*-caused PA reduction (Figure 4(b)). Therefore, we speculated that PA supplementation had more antiageing effects than simple PAOX silencing in NP cells. Other evidence was provided from the protein analysis that only the spermidine-pretreated NP cells showed a depressed *β*-gal and p16 expression compared to the IL-1*β* group (Figure 4(c)). Besides, the pretreatment of spermidine had a limited impact on the increase of PAOX within the IL-1*β* treatment (Figure 4(c)). To further clear the ECM metabolism profile, we tested collagen II, aggrecan (ACAN), representative matrix proteinases (MMPs and ADAMTSs), and antiproteases (TIMPs) synthesized by NP cells. The result indicated that spermidine pretreatment rescued the reduction of collagen II, ACAN, and TIMP4 and inhibited the MMP9/13 and ATAMTS2 expression. Besides, a silenced-PAOX gene also delayed the reduction of TIMP4 and prevented MMP9/13 and ATAMTS2 upregulation (Figures 4(c) and 4(d)).

### 3.5. Supplementation of Spermidine or NAC and Clearance of p16 Suppress Oxidative Stress in Mouse NP Tissues

**Figure 5:**
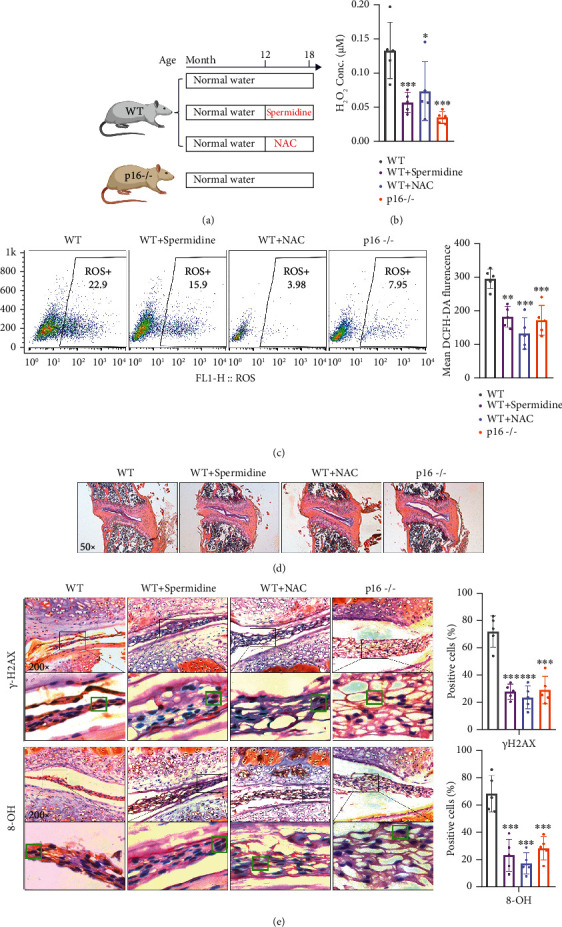
Spermidine and NAC supplementation and p16 clearance alleviate oxidative stress and DNA damage in the old mouse. (a) Illustration of the mouse feeding pattern; (b) H_2_O_2_ levels in disc tissues of mice; (c) flow cytometry of total ROS and the quantitation; (d) representative images of H&E staining of the disc between 1^st^ and 2^nd^ lumbar vertebra; (e) representative images of slices stained immunohistochemically for *γ*H2AX and 8-OH and quantitation for the positive cell (marked in the green boxes) ratio from disc between 1^st^ and 2^nd^ lumbar vertebra. Data are presented as mean ± SD, *n* = 5; ^∗^*p* < 0.05, ^∗∗^*p* < 0.01, and ^∗∗∗^*p* < 0.001 compared to WT.

Knowledge from the in vitro study, PA supplementation may be much more functional than the PAOX suppression in antiageing of NP cells. Thus, we assessed whether the oral supplementation of spermidine influenced the IDD in older mice. Additionally, our previous studies have confirmed that NAC (a cysteine and glutathione precursor has been used as an antioxidant for few decades) [24] supplement and p16^INK4a^ (p16, encoded by Cdkn2a gene) clearance contribute to balance the oxidative stress and delay the ageing process of NP cells. To determine whether NAC and p16 intervene IDD via regulating PA metabolism, NAC-supplemented and CDKN2A gene knocked out (p16^−/−^) mice were also sacrificed as a comparison. White type (WT) and the littermates p16^−/−^ mice were fed with normal water until 12 months old, and then, two groups of WT mice were, respectively, supplied with spermidine- or NAC-contained water for another 6 months. One group of WT and one group of p16^−/−^ mice were unceasingly fed by normal water until 18 months old (Figure 5(a)). Finally, the thoracic and lumbar disc tissues were harvested for the following experiments. In particular, it should be noted that the disc tissues in mice are so small that we were unable to completely separate the NP part, so the experimental results below refer to the intact disc containing the endplate and the annulus fibrosus surrounding the NP. Compared to WT, spermidine/NAC supplementation and p16 clearance significantly inhibited both the H_2_O_2_ and ROS accumulation in the disc tissue (Figures 5(b) and 5(c)). The representative view of the intervertebral disc between the 1^st^ and 2^nd^ lumbar vertebra is shown in Figure5(d). Besides, we also analyzed these two DNA damage markers from the discs between the 1^st^ to 5^th^ lumbar vertebra and found they were both reduced accompanied by the downregulated ROS level (Figure 5(e)). Like p16 clearance, spermidine or NAC supplementation prevents ROS accumulation and DNA damage during the ageing progress in the mouse.

### 3.6. Balance Polyamine Metabolism Delays IDD in Mouse

**Figure 6:**
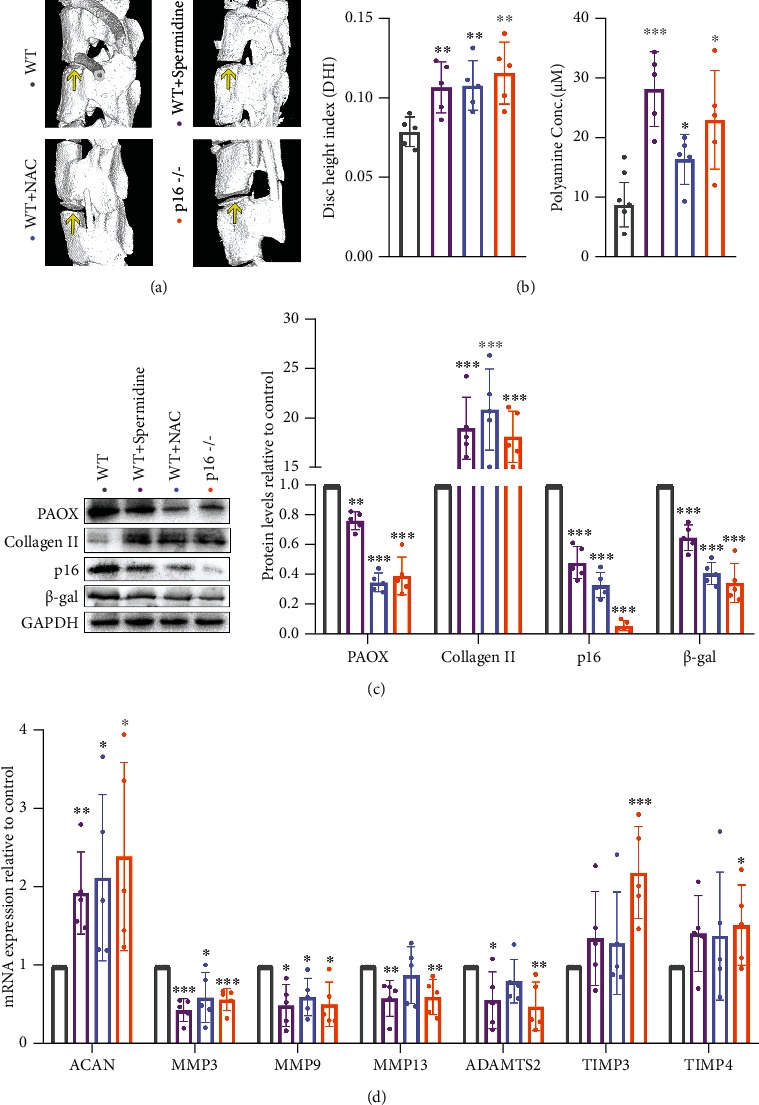
Spermidine and NAC supplementation and p16 clearance delay mouse IDD. (a) Representative micro-CT of the mouse 12^th^ thoracic vertebra to the 1^st^ lumbar vertebra, and the average disc height index (DHI) from 10^th^ thoracic vertebra to the 2^nd^ lumbar vertebra; (b) total PA concentration in disc tissues of mice; (c) western blotting and measured by densitometry analyses and expressed as folds relative to WT; (d) target mRNA expression assessed by RT-PCR. Data are presented as mean ± SD, *n* = 5; ^∗^*p* < 0.05, ^∗∗^*p* < 0.01, and ^∗∗∗^*p* < 0.001 compared to WT.

With the development of IDD, the disc height gradually decreases due to the disordered ECM and the loss of water in NP tissue. The average disc height index (DHI) from the tenth thoracic vertebra to the second lumbar vertebra was calculated as described in our previous study [6]. The result showed that spermidine/NAC supplementation or p16 clearance protected the loss of disc height compared to the WT (Figure 6(a)). In addition to spermidine supplementation, NAC supplementation or p16 clearance can also increase PA content in mouse disc (Figure 6(b)). Meanwhile, supplementation of spermidine/NAC or clearance of p16 can also inhibit PAOX expression (Figure 6(c)), which means the PA metabolism is protected by an additional supply of spermidine/NAC or p16 clearance. As mentioned above, a decreased level of p16 and *β*-gal in the disc also indicated that supplying spermidine/NAC and p16 clearance could delay the ageing process of mouse discs (Figure 6(c)). Focusing on the ECM metabolism, spermidine defended collagen II and ACAN levels and prevented the MMP3/9/13 and ATAMTS2 expressions; NAC protected collagen II and ACAN levels and prevented the MMP3/9 expressions; p16 clearance rescued collagen II, ACAN, and TIMP3/4 levels and prevented the MMP3/9/13 and ATAMTS2 expressions. Thus, maintaining a high level of PAs helps to delay the IDD in mouse, and NAC supplementation or CDKN2A knockout keep a balance of the PA metabolism.

## 4. Discussion

IDD-based spine diseases seriously endanger human health, especially the elderly populations [25]. Due to the complicated pathogenesis, there have been limited treatment options and outcomes for IDD [26]. In recent years, genetic factors have been indicated as critical contributors to the onset and development of IDD [27, 28]. Nowadays, bioinformatics analyses [29], with advantages of high parallel, miniaturization and automation, are frequently utilized in identifying relevant genes of interest and biomarkers for IDD diagnosis and prognosis [30, 31]. This study used the bioinformatics method to identify PAOX as a risk gene involved in oxidative stress during IDD. PAOX is a FAD-dependent amine oxidase with high activity in most tissues, which mainly participates in the catabolism of PAs [32]. The dysregulation of PA metabolism is commonly associated with various diseases [33–35], suggesting that such PAOX upregulation-caused PA dysregulation may give helpful insight into a therapeutic benefit of IDD. To verify the upregulation of PAOX and clear how PAs change in the chronic course of IDD, we tested the total PA content and PAOX protein expression in the non-, mild-, and severe-degenerated human NP tissues. The data showed PAs markedly decreased, and PAOX largely increased in the mild-degenerated NP tissues but stayed at a relatively stable stage from mild- to severe-degenerated status. Differently, the PAOX-positive NP cells were gradually increased in the overall progress from non- to severe-degenerated condition. We speculate that NP cell numbers are decreasing at the later stages of IDD, so that the losing of total PAOX-positive cells leads to the PAOX in a plateau. As expected, these findings support the bioinformatics study that POAX and PAs are dysregulated in the degenerated discs.

PA concentration in mammals decreases with age, which is governed by the dietary intake, intestinal microbiota production, cellular biosynthesis, catabolism, and urine excretion [36]. Additionally, the naturally occurring PA spermidine is progressively making its way into clinical trials due to the functions of life-span extension and protection of age-associated diseases, e.g., high blood pressure, heart failure, and muscle-related disorders [37–39]. To alleviate the loss of PAs in the degenerated NP cells, we intervened in PA metabolism of the degenerated NP cells through (i) enhancing extracellular uptake via spermidine supplementation and (ii) preventing its catabolism via PAOX silencing. As a conventional model *in vitro*, we used IL-1*β* to cause an inflammatory degeneration of NP cells [40, 41]. Because it is not clear whether coculturing with IL-1*β* affects the bioactivity of spermidine, we used sequential treatment of spermidine and IL-1*β* instead of coculture. As expected, PA metabolism was disrupted reflecting in the decreased PAs and increased PAOX in the IL-1*β*-treated NP cells, which can be obviously rebalanced by either spermidine pretreatment or PAOX silencing. As ROS in the form of H_2_O_2_ is a major product of PA catabolism [12], we also verified that the upregulated H_2_O_2_ and total ROS level caused by IL-1*β* stimuli can be alleviated by both spermidine pretreatment and PAOX gene silencing, indicating a rebalance of PA metabolism plays a role in reducing ROS-dependent oxidative stress. Recent studies have suggested the primary role for cell senescence responses to ROS stress in IDD, characterized by accumulation of DNA damaged and cell cycle-arrested NP cells with a high level of catabolism and damaged anabolism in ECM profiles [6, 42]. For instance, excessive H_2_O_2_ triggers the senescent process via arresting NP cells at the G0/G1 phase in the cell cycle [43]. Sustained ROS stimulation damages the serial replication of the DNA with an enhanced formation of *γ*H2AX foci [44] and 8-OH mispairing [45], which results in the replicative senescence. Due to the low level of ROS, we found the *γ*H2AX and 8-OH levels were also prevented when PA metabolism was balanced.

Additionally, senescence-associated *β*-galactosidase (SA-*β*-gal) activity [46] and p16, the sensitive indicators of senescence, were tested to recover whether PA metabolism affects NP cell senescence. Interestingly, different modalities of PA regulation have different efficiency on cellular senescence, although they both can effectively inhibit oxidative stress. The results illustrate that exogenously supplying PAs is more effective than knocking down PAOX on the senescent process of NP cells. Indeed, PAs not only have antioxidative but also immunostimulatory [47], anti-inflammatory [37], and autophagy-regulatory [48] effects. Thus, the complex processes may determine that appropriate PA catabolism is also necessary for life activities and exogenous supply of PAs to maintain the normal level without affecting the breakdown of PAs are much effective in inhibiting NP cell senescence. Senescence of NP cells likewise accelerates the catabolism and decreases the anabolism of ECM, as evidenced by a decrease in collagen II/ACAN content [49]. Even though they can both suppress catabolism via balancing MMPs/ADAMTS2 and TIMPs, pretreatment with spermidine was more likely than PAOX silencing to maintain ACAN and collage II composition.

To further verify whether spermidine supplementation delays IDD *in vivo*, we used the mouse natural IDD model in elderly mice. Unlike our previous application of physical [6] or surgical [50] ways to cause IDD in mice, age-related spontaneous IDD better simulates the natural ageing process of human intervertebral disc tissue. An early study has confirmed that a mild but significant IDD occurred in mice at 14-month-old with slow progress to severe condition at 22 months old [51]. Therefore, we fed mice with spermidine-contained water from 12 months to 18 months old to intervene in the early onset of IDD. NAC is a key precursor to a variety of endogenous antioxidants that aid in the breakdown of peroxides and the suppression of oxidative stress by supplying cellular glutathione. In our previous studies, the antioxidant NAC treatment inhibited the Bmi-1 gene deficiency-caused oxidative stress and IDD progress in mice [10], and deletion p16 also attenuated the mechanical force-caused mouse IDD through inhibiting oxidative stress and cell senescence [6]. However, whether the functions of NAC supplementation and p16 deletion on IDD are associated with regulating PA metabolism is not clear. Therefore, we also fed the WT mice with NAC and utilized Cdkn2a gene knockout mice to determine whether PA metabolism can be also rebalanced. Fortunately, the three strategies all reduced total H_2_O_2_, ROS, and DNA damage levels in the ageing process of disc tissues.

**Figure 7:**
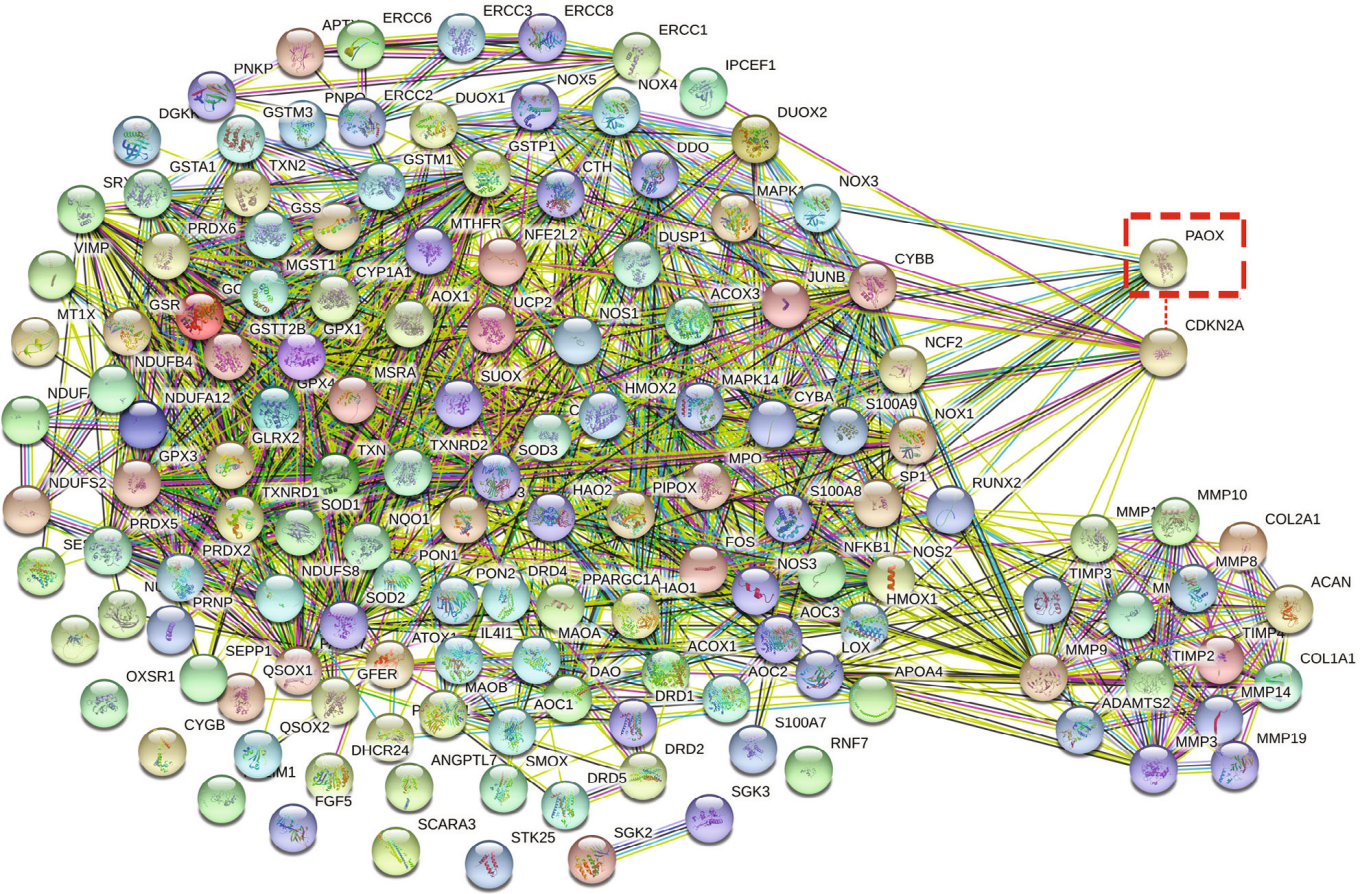
PPI network of oxidative stress-related genes (large spheres on the left) and ECM-related genes (small spheres on the right). We speculate the highlighted PAOX in the red dot-box and CDK2A have some interactions.

In mice, oral spermidine supplementation efficiently increases the inner PA levels, containing whole blood, serum, and tissues [37, 52]. Moreover, a daily intake of 50 to 100 g of natto for two months dramatically raised the whole-blood spermine concentration of healthy adult volunteers [53]. Therefore, ingestion of a high PA-rich diet is conceivable to prevent the age-related decrease of PAs. Different from the three days' in vitro experiments, six months' spermidine supplementation effectively suppressed the age-caused PAOX upregulation in mice, indicating a long-term ectogenic intake effectively rebalances the PA catabolism. In addition to once again confirming the inhibitory effect of NAC supplementation and Cdkn2a knockout on IDD, we found that they also participate in the PA metabolism via maintaining the PA content and inhibiting PAOX expression. Plenty of literature suggests that the Cdkn2a gene is closely linked to many other genes, e.g., Nf*κ*B-p65, CAT, SOD, and NOS, in the oxidative stress pathways [54]. This study revealed silencing the PAOX gene did not affect p16 expression, but Cdkn2a knockout significantly suppressed PAOX expression. To understand the relationship between Cdkn2a and PAOX, we used the STRING platform to achieve a significant module in the PPI networks regarding oxidative stress and ECM metabolism in IDD. Oxidative stress-related genes make up the large spheres on the left of Figure 7, and ECM-related genes make up the small spheres on the right of Figure 7. The highlighted PAOX in the red dot-box only shows an association with oxidative stress but not ECM genes, whereas Cdkn2a interacts with both oxidative stress- and ECM metabolism-relative genes. Interested researchers can access the specific role of the Cdkn2a gene in the regulation of oxidative stress and ECM in IDD from our previous work [6]. Due to Cdkn2a regulating plenty of oxidative stress genes and PAOX having a positive correlation with Cdkn2a from our study, we assume that Cdkn2a may be involved in the upstream activation or regulation of PAOX.

Additionally, the potential limitations of this study are (i) IL-1*β*-caused NP cell degeneration is inflammation but not ageing dependent, and our study did not analyze any inflammatory factors, which can be an individual study in the future focusing on the role of PAs in the inflammation of NP cells; (ii) spermidine is one of the forms of PAs, and the functions of other forms (putrescine and spermine) should be detected in the next step; and (iii) the effects of spermine oxidase another catabolic enzyme in PA metabolism are not analyzed here, which may also influence the IDD progress.

## 5. Conclusion

**Figure 8:**
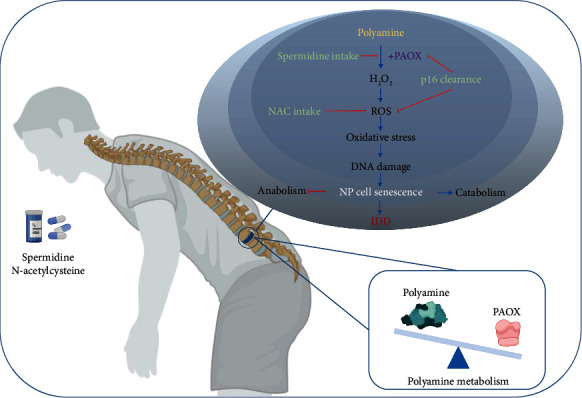
A proposed model depicting the dysregulation of polyamine (PA) metabolism in regulating human IDD. PAOX promotes the catabolism of PA and produces H_2_O_2_ as a source of reactive oxygen species (ROS) to cause oxidative stress and the resulting DAN damage, which accelerates the NP cell senescence and IDD progress. Spermidine and N-acetylcysteine (NAC) supplementation or cellular p16 clearance balance the PA metabolism in the disc and delay the IDD progress (figure is created in http://BioRender.com).

Taken together, our study provides evidence that dysregulation of PA metabolism, especially the excessive anabolism of PAs, participates in the progress of IDD. PAOX may be a biomarker and therapeutic target in disc diseases. Cellular p16 clearance and NAC/spermidine supplementation balance the PA metabolism and oxidative stress via preventing both the age-related PAOX upregulation and PA reduction. For the first time, we suggest that spermidine can be a novel drug in clinical practice for IDD via delaying the ageing of NP cells and preventing ECM catabolism in the disc (Figure 8).

## Figures and Tables

**Table 1:** Primer sequences of the genes for RT-PCR.

Gene name	Forward (5′ > 3′)	Reverse (5′ > 3′)
ACAN	ACTCTGGGTTTTCGTGACTCT	ACACTCAGCGAGTTGTCATGG
MMP3	AGTCTTCCAATCCTACTGTTGCT	TCCCCGTCACCTCCAATCC
MMP9	GCCACTACTGTGCCTTT	CCCTCAGAGAATCGCC
MMP13	ACTGAGAGGCTCCGAGAAATG	GAACCCCGCATCTTGGCTT
ADAMTS2	GTGCATGTGGTGTATCGCC	AGGACCTCGATGTTGTAGTCA
TIMP3	CATGTGCAGTACATCCATACGG	CATCATAGACGCGACCTGTCA
TIMP4	CACTACCATCTGAACTGTGGCTG	GCTTTCGTTCCAACAGCCAGTC
GAPDH	ACAACTTTGGTATCGTGGAAGG	GCCATCACGCCACAGTTTC

RT-PCR: quantitative reverse-transcription polymerase chain reaction.

**Table 2:** The top 10 upregulated and downregulated DEGs.

Gene name	Log fold change	*p* value
CYP1B1	21.19	0.04
POSTN	10.77	0.0031
SULF1	9.15	0.0025
CYP1B1	8.67	0.0387
ARL4C	8.35	0.0073
EFEMP1	8.03	0.0357
SULF1	7.35	0.002
CYP1B1	5.41	0.0437
EPS8	4.97	0.0031
IFI16	3.74	0.0136
SULT1A2	-2.23	0.0237
DDR1	-2.24	0.0258
ST6GALNAC4	-2.29	0.0462
CSDC2	-2.4	0.0499
ECHDC3	-2.45	0.0034
CSPG4	-2.45	0.0041
KRT19	-2.48	0.0487
HCFC1R1	-2.75	0.0365
CLIC3	-3.57	0.0243
LOC728392	-4.1	0.0063

Positive values: upregulated; negative values: downregulated.

**Table 3:** The 4 oxidative-related DEGs.

Gene name	Description	Log fold change	*p* value
PCYOX1L	Prenylcysteine oxidase 1 like	1.71	0.0013
MAPK14	Mitogen-activated protein kinase 14	1.32	0.0125
CTH	Cystathionine gamma-lyase	1.53	0.0216
PAOX	Polyamine oxidase (exo-N4-amino)	1.22	0.0379

Positive values: upregulated.

## Data Availability

The datasets generated and/or analyzed during the present study are available from the corresponding author upon reasonable request.

## Supplementary Materials

Supplementary Materials The raw data of the bioinformatics analysis containing DEGs, GO, KEGG, and PPI are provided in the supplementary file.
