# Supplementary material for: Rebalance of the Polyamine Metabolism Suppresses Oxidative Stress and Delays Senescence in Nucleus Pulposus Cells
Source: Oxid Med Cell Longev. 2022 Feb 7;2022:8033353. doi: 10.1155/2022/8033353 (PMC8844099; doi:10.1155/2022/8033353)
Supplement: Supplementary Materials — The raw data of the bioinformatics analysis containing DEGs, GO, KEGG, and PPI are provided in the supplementary file. [file 8033353.f1.zip › 8033353.f1/go.docx]

**ONTOLOGY ID Description GeneRatio BgRatio pvalue p.adjust qvalue geneID Count**

BP GO:0001501 skeletal system development 24/262 486/18862 8.54E-08 0.000301969 0.00025614 PLS3/SULF1/PDGFC/COL3A1/GJA1/RUNX3/SP3/FGFR3/BBX/INSIG1/DLX2/PRRX1/GREM1/FGF18/CSGALNACT1/SMAD2/EFEMP1/CDH11/MMP2/FBN1/RARG/SNAI1/HOXA10/PTPN11 24

BP GO:0060348 bone development 13/262 193/18862 3.17E-06 0.005609242 0.004757951 PLS3/SULF1/PDGFC/GJA1/FGFR3/BBX/INSIG1/GREM1/FGF18/CSGALNACT1/FBN1/RARG/PTPN11 13

BP GO:0009612 response to mechanical stimulus 13/262 202/18862 5.23E-06 0.006169982 0.00523359 BDKRB1/PIEZO2/POSTN/COL3A1/GJA1/TXNIP/FYN/PKD2/BAD/NFKBIA/CXCL12/PSPH/PTPN11 13

BP GO:0008630 intrinsic apoptotic signaling pathway in response to DNA damage 9/262 100/18862 1.06E-05 0.007896322 0.006697931 MSH6/IFI16/DYRK2/BCL2L1/BAD/SFN/PRKDC/SNAI1/CXCL12 9

BP GO:0051216 cartilage development 12/262 185/18862 1.12E-05 0.007896322 0.006697931 SULF1/RUNX3/FGFR3/DLX2/PRRX1/GREM1/FGF18/CSGALNACT1/EFEMP1/RARG/SNAI1/PTPN11 12

BP GO:0061448 connective tissue development 13/262 243/18862 3.74E-05 0.022052021 0.018705278 SULF1/RUNX3/FGFR3/DLX2/PRRX1/GREM1/FGF18/CSGALNACT1/EFEMP1/RARG/SNAI1/ATF2/PTPN11 13

BP GO:0097193 intrinsic apoptotic signaling pathway 14/262 283/18862 4.45E-05 0.022500468 0.019085667 SIAH1/CASP4/MSH6/IFI16/DYRK2/DNAJC10/BCL2L1/BAD/BCLAF1/SFN/CYP1B1/PRKDC/SNAI1/CXCL12 14

BP GO:1904645 response to amyloid-beta 6/262 51/18862 7.23E-05 0.03199238 0.027137031 ABCC1/GJA1/CASP4/IGF1R/FYN/MMP2 6

BP GO:0000723 telomere maintenance 10/262 161/18862 8.70E-05 0.0335764 0.028480651 RFC4/POLA1/RAD51C/CCT6A/HSP90AA1/PRKDC/POLD3/PRIM1/HNRNPA1/CCT8 10

BP GO:0002062 chondrocyte differentiation 8/262 103/18862 9.49E-05 0.0335764 0.028480651 SULF1/RUNX3/FGFR3/GREM1/FGF18/EFEMP1/RARG/PTPN11 8

BP GO:1901653 cellular response to peptide 16/262 391/18862 0.000123026 0.039569685 0.033564361 ABCC1/ECHDC3/MYO1C/GJA1/CASP4/IGF1R/FYN/INSIG1/PRKAR1A/OSBPL8/ID1/CDC5L/FBN1/PRKDC/GDF15/PTPN11 16

BP GO:0032200 telomere organization 10/262 174/18862 0.000165133 0.042634105 0.036163706 RFC4/POLA1/RAD51C/CCT6A/HSP90AA1/PRKDC/POLD3/PRIM1/HNRNPA1/CCT8 10

BP GO:0044344 cellular response to fibroblast growth factor stimulus 9/262 142/18862 0.000168106 0.042634105 0.036163706 SULF1/POSTN/FGFR3/FGF18/CDC5L/HNRNPA1/FGFBP1/POLR2B/PTPN11 9

BP GO:0051897 positive regulation of protein kinase B signaling 10/262 175/18862 0.000173038 0.042634105 0.036163706 MTDH/IGF1R/FGFR3/FYN/FGF18/OSBPL8/ANGPT1/HSP90AA1/GDF15/PTPN11 10

BP GO:0038083 peptidyl-tyrosine autophosphorylation 4/262 21/18862 0.000180755 0.042634105 0.036163706 IGF1R/GREM1/DYRK1A/DDR1 4

BP GO:0001704 formation of primary germ layer 8/262 115/18862 0.000204675 0.043604928 0.036987191 FN1/GJA1/CDC73/PRKAR1A/COL8A1/SMAD2/MMP2/SNAI1 8

BP GO:0018108 peptidyl-tyrosine phosphorylation 15/262 369/18862 0.000214714 0.043604928 0.036987191 PDGFC/CSPG4/IGF1R/FGFR3/FYN/DYRK2/GREM1/DYRK1A/DDR1/ENPP2/TWF1/ANGPT1/EFEMP1/TNK2/PTPN11 15

BP GO:0071774 response to fibroblast growth factor 9/262 148/18862 0.000229473 0.043604928 0.036987191 SULF1/POSTN/FGFR3/FGF18/CDC5L/HNRNPA1/FGFBP1/POLR2B/PTPN11 9

BP GO:0018212 peptidyl-tyrosine modification 15/262 372/18862 0.000234214 0.043604928 0.036987191 PDGFC/CSPG4/IGF1R/FGFR3/FYN/DYRK2/GREM1/DYRK1A/DDR1/ENPP2/TWF1/ANGPT1/EFEMP1/TNK2/PTPN11 15

BP GO:1904646 cellular response to amyloid-beta 5/262 41/18862 0.000247841 0.043604928 0.036987191 ABCC1/GJA1/CASP4/IGF1R/FYN 5

BP GO:0032331 negative regulation of chondrocyte differentiation 4/262 23/18862 0.000261654 0.043604928 0.036987191 GREM1/EFEMP1/RARG/PTPN11 4

BP GO:2000573 positive regulation of DNA biosynthetic process 6/262 65/18862 0.000282146 0.043604928 0.036987191 RFC4/GREM1/CCT6A/HSP90AA1/HNRNPA1/CCT8 6

BP GO:0045185 maintenance of protein location 7/262 92/18862 0.000295359 0.043604928 0.036987191 GJA1/LTBP1/INSIG1/PKD2/TWF1/NFKBIA/FBN1 7

BP GO:0035581 sequestering of extracellular ligand from receptor 3/262 10/18862 0.000295794 0.043604928 0.036987191 LTBP1/GREM1/FBN1 3

BP GO:0046777 protein autophosphorylation 11/262 226/18862 0.000334883 0.045942234 0.038969775 PDGFC/HTATIP2/IGF1R/FGFR3/PPP2R5E/GREM1/DYRK1A/DDR1/PPP2R5C/SLK/CHP1 11

BP GO:0050673 epithelial cell proliferation 16/262 428/18862 0.000340517 0.045942234 0.038969775 SULF1/GJA1/RUNX3/CDC73/FAP/COL8A1/ID1/BAD/CDH13/SFN/PRKDC/CXCL12/HMGB1/FGFBP1/ATF2/ROBO1 16

BP GO:1900078 positive regulation of cellular response to insulin stimulus 4/262 25/18862 0.000365722 0.045942234 0.038969775 ECHDC3/MYO1C/OSBPL8/PTPN11 4

BP GO:1905475 regulation of protein localization to membrane 10/262 194/18862 0.000395838 0.045942234 0.038969775 MYO1C/LRRC15/CEMIP/FYN/BCL2L1/BAD/LYPLA1/CRIPT/SFN/CHP1 10

BP GO:1903793 positive regulation of anion transport 17/262 478/18862 0.000396795 0.045942234 0.038969775 MYO1C/GJA1/PPID/CEMIP/LPCAT3/FYN/IPO5/MEF2A/OSBPL8/BAD/PLA2G6/PLA2G4A/SFN/NFKBIA/CXCL12/CHP1/PTPN11 17

BP GO:0034975 protein folding in endoplasmic reticulum 3/262 11/18862 0.000402546 0.045942234 0.038969775 DNAJC10/CANX/HSP90B1 3

BP GO:0061314 Notch signaling involved in heart development 3/262 11/18862 0.000402546 0.045942234 0.038969775 JAG1/RBPJ/SNAI1 3

BP GO:0030198 extracellular matrix organization 15/262 393/18862 0.000418786 0.046106037 0.039108717 SULF1/POSTN/COL3A1/FN1/FBLN2/LAMB2/GREM1/FAP/COL8A1/DDR1/CSGALNACT1/COL15A1/CYP1B1/MMP2/FBN1 15

BP GO:0043062 extracellular structure organization 15/262 394/18862 0.000430045 0.046106037 0.039108717 SULF1/POSTN/COL3A1/FN1/FBLN2/LAMB2/GREM1/FAP/COL8A1/DDR1/CSGALNACT1/COL15A1/CYP1B1/MMP2/FBN1 15

BP GO:0045229 external encapsulating structure organization 15/262 396/18862 0.000453344 0.046591237 0.039520281 SULF1/POSTN/COL3A1/FN1/FBLN2/LAMB2/GREM1/FAP/COL8A1/DDR1/CSGALNACT1/COL15A1/CYP1B1/MMP2/FBN1 15

BP GO:0016574 histone ubiquitination 5/262 47/18862 0.000474077 0.046591237 0.039520281 CUL4B/CDC73/TRIP12/WAC/TRIM37 5

BP GO:0031952 regulation of protein autophosphorylation 5/262 47/18862 0.000474077 0.046591237 0.039520281 PDGFC/PPP2R5E/GREM1/PPP2R5C/CHP1 5

BP GO:0032201 telomere maintenance via semi-conservative replication 4/262 27/18862 0.00049644 0.047408972 0.040213912 RFC4/POLA1/POLD3/PRIM1 4

BP GO:1900115 extracellular regulation of signal transduction 3/262 12/18862 0.000531229 0.047408972 0.040213912 LTBP1/GREM1/FBN1 3

BP GO:1900116 extracellular negative regulation of signal transduction 3/262 12/18862 0.000531229 0.047408972 0.040213912 LTBP1/GREM1/FBN1 3

BP GO:0051235 maintenance of location 13/262 319/18862 0.000548952 0.047408972 0.040213912 BDKRB1/GJA1/GPSM2/CEMIP/LTBP1/INSIG1/PKD2/OSBPL8/TWF1/EHD1/HSP90B1/NFKBIA/FBN1 13

BP GO:0043687 post-translational protein modification 14/262 361/18862 0.00055462 0.047408972 0.040213912 ATG12/CUL4B/PENK/FBXL5/FN1/DCAF6/LTBP1/LAMB2/STC2/CAND1/UBA3/HSP90B1/FBN1/FBXO2 14

BP GO:0061037 negative regulation of cartilage development 4/262 28/18862 0.000572904 0.047408972 0.040213912 GREM1/EFEMP1/RARG/PTPN11 4

BP GO:0032330 regulation of chondrocyte differentiation 5/262 49/18862 0.000576197 0.047408972 0.040213912 GREM1/FGF18/EFEMP1/RARG/PTPN11 5

BP GO:0010390 histone monoubiquitination 4/262 29/18862 0.00065737 0.052858544 0.044836425 CUL4B/CDC73/WAC/TRIM37 4

BP GO:0007171 activation of transmembrane receptor protein tyrosine kinase activity 3/262 13/18862 0.000683527 0.05374041 0.045584455 PDGFC/GREM1/ANGPT1 3

BP GO:0051896 regulation of protein kinase B signaling 11/262 247/18862 0.000704872 0.054213852 0.045986044 MTDH/IGF1R/FGFR3/FYN/FGF18/OSBPL8/PPP2R5C/ANGPT1/HSP90AA1/GDF15/PTPN11 11

BP GO:2000278 regulation of DNA biosynthetic process 7/262 107/18862 0.000737375 0.054331389 0.046085742 RFC4/GJA1/GREM1/CCT6A/HSP90AA1/HNRNPA1/CCT8 7

BP GO:0090314 positive regulation of protein targeting to membrane 4/262 30/18862 0.000750292 0.054331389 0.046085742 MYO1C/CEMIP/FYN/CHP1 4

BP GO:0006289 nucleotide-excision repair 7/262 108/18862 0.000779304 0.054331389 0.046085742 CUL4B/RFC4/SLC30A9/TCEA1/POLD3/HMGB1/POLR2B 7

BP GO:0008593 regulation of Notch signaling pathway 7/262 108/18862 0.000779304 0.054331389 0.046085742 POSTN/RFNG/DLX2/JAG1/NFKBIA/RBPJ/ROBO1 7

BP GO:0050678 regulation of epithelial cell proliferation 14/262 374/18862 0.000783183 0.054331389 0.046085742 SULF1/GJA1/RUNX3/CDC73/ID1/BAD/CDH13/SFN/PRKDC/CXCL12/HMGB1/FGFBP1/ATF2/ROBO1 14

BP GO:0048705 skeletal system morphogenesis 10/262 213/18862 0.000818791 0.055334297 0.046936444 FGFR3/INSIG1/DLX2/PRRX1/GREM1/FGF18/CSGALNACT1/SMAD2/MMP2/RARG 10

BP GO:0001706 endoderm formation 5/262 53/18862 0.00082892 0.055334297 0.046936444 FN1/CDC73/COL8A1/SMAD2/MMP2 5

BP GO:0007369 gastrulation 9/262 179/18862 0.000915977 0.059166205 0.050186799 FN1/GJA1/CDC73/PRKAR1A/DLD/COL8A1/SMAD2/MMP2/SNAI1 9

BP GO:0007006 mitochondrial membrane organization 8/262 144/18862 0.000922859 0.059166205 0.050186799 BCL2L1/RHOT1/CHCHD3/HSPA4/BAD/SFN/HSP90AA1/ATF2 8

BP GO:0048048 embryonic eye morphogenesis 4/262 32/18862 0.000963321 0.059166205 0.050186799 SP3/EFEMP1/FBN1/RARG 4

BP GO:0071559 response to transforming growth factor beta 11/262 257/18862 0.000975039 0.059166205 0.050186799 USP15/PENK/POSTN/COL3A1/RUNX3/FYN/LTBP1/ID1/SMAD2/FBN1/GDF15 11

BP GO:0006913 nucleocytoplasmic transport 13/262 340/18862 0.000988315 0.059166205 0.050186799 HTATIP2/TXNIP/IPO5/TNPO1/UPF3A/XPOT/ANGPT1/SFN/NFKBIA/HNRNPA1/CHP1/PTPN11/NPM1 13

BP GO:0008543 fibroblast growth factor receptor signaling pathway 7/262 113/18862 0.001018069 0.059166205 0.050186799 SULF1/FGFR3/FGF18/HNRNPA1/FGFBP1/POLR2B/PTPN11 7

BP GO:0007596 blood coagulation 13/262 342/18862 0.001042346 0.059166205 0.050186799 CAPZA1/COL3A1/FN1/FYN/PRKAR1A/FAP/RAD51C/EHD1/PLA2G4A/F13A1/TFPI/MYL12A/PTPN11 13

BP GO:0034638 phosphatidylcholine catabolic process 3/262 15/18862 0.001065297 0.059166205 0.050186799 ENPP2/PLA2G6/PLA2G4A 3

BP GO:0070885 negative regulation of calcineurin-NFAT signaling cascade 3/262 15/18862 0.001065297 0.059166205 0.050186799 HOMER3/DYRK2/CHP1 3

BP GO:0106057 negative regulation of calcineurin-mediated signaling 3/262 15/18862 0.001065297 0.059166205 0.050186799 HOMER3/DYRK2/CHP1 3

BP GO:0051169 nuclear transport 13/262 343/18862 0.001070276 0.059166205 0.050186799 HTATIP2/TXNIP/IPO5/TNPO1/UPF3A/XPOT/ANGPT1/SFN/NFKBIA/HNRNPA1/CHP1/PTPN11/NPM1 13

BP GO:0007599 hemostasis 13/262 346/18862 0.001157849 0.062397767 0.05292792 CAPZA1/COL3A1/FN1/FYN/PRKAR1A/FAP/RAD51C/EHD1/PLA2G4A/F13A1/TFPI/MYL12A/PTPN11 13

BP GO:0043434 response to peptide hormone 15/262 435/18862 0.001177695 0.062397767 0.05292792 ECHDC3/MYO1C/GJA1/IGF1R/FYN/INSIG1/PRKAR1A/STC2/OSBPL8/CDC5L/FBN1/PRKDC/GDF15/CXCL12/PTPN11 15

BP GO:0050817 coagulation 13/262 347/18862 0.001188338 0.062397767 0.05292792 CAPZA1/COL3A1/FN1/FYN/PRKAR1A/FAP/RAD51C/EHD1/PLA2G4A/F13A1/TFPI/MYL12A/PTPN11 13

BP GO:0007219 Notch signaling pathway 9/262 186/18862 0.001199279 0.062397767 0.05292792 POSTN/RFNG/DLX2/JAG1/NFKBIA/RBPJ/SNAI1/ROBO1/KRT19 9

BP GO:0033260 nuclear DNA replication 5/262 59/18862 0.001351755 0.069050112 0.058570667 RFC4/POLA1/MCM6/POLD3/PRIM1 5

BP GO:0001101 response to acid chemical 7/262 119/18862 0.001376687 0.069050112 0.058570667 PDGFC/FYN/IPO5/PKD2/BAD/MMP2/ATF2 7

BP GO:0051052 regulation of DNA metabolic process 13/262 353/18862 0.001385686 0.069050112 0.058570667 RFC4/GJA1/TRIP12/MSH6/GREM1/ACTR2/CCT6A/HSP90AA1/PRKDC/HMGB1/HNRNPA1/CCT8/NPM1 13

BP GO:0090150 establishment of protein localization to membrane 13/262 354/18862 0.001421089 0.069830722 0.059232807 SRP72/MYO1C/TRAM2/CEMIP/FYN/HSPA4/BAD/SRP9/LYPLA1/ATG3/SFN/HSP90AA1/CHP1 13

BP GO:0071900 regulation of protein serine/threonine kinase activity 16/262 492/18862 0.001495711 0.071183875 0.060380598 PDGFC/CSPG4/IGFBP6/IGF1R/CEMIP/PRKAR1A/IPO5/CCND2/FGF18/PKD2/CDKN1B/SFN/GDF15/CCNG2/ROBO1/PTPN11 16

BP GO:0003176 aortic valve development 4/262 36/18862 0.001510886 0.071183875 0.060380598 JAG1/RBPJ/SNAI1/ROBO1 4

BP GO:0090313 regulation of protein targeting to membrane 4/262 36/18862 0.001510886 0.071183875 0.060380598 MYO1C/CEMIP/FYN/CHP1 4

BP GO:0010631 epithelial cell migration 13/262 357/18862 0.00153184 0.071183875 0.060380598 GREM1/PTPRG/FAP/FGF18/ENPP2/ID1/ANGPT1/CDH13/CYP1B1/HMGB1/FGFBP1/ROBO1/PTPN11 13

BP GO:0003170 heart valve development 5/262 61/18862 0.001570628 0.071183875 0.060380598 MATR3/JAG1/RBPJ/SNAI1/ROBO1 5

BP GO:0043491 protein kinase B signaling 11/262 273/18862 0.001581795 0.071183875 0.060380598 MTDH/IGF1R/FGFR3/FYN/FGF18/OSBPL8/PPP2R5C/ANGPT1/HSP90AA1/GDF15/PTPN11 11

BP GO:0071897 DNA biosynthetic process 9/262 194/18862 0.001605654 0.071183875 0.060380598 RFC4/GJA1/GREM1/POLA1/CCT6A/HSP90AA1/POLD3/HNRNPA1/CCT8 9

BP GO:0031589 cell-substrate adhesion 13/262 359/18862 0.001609585 0.071183875 0.060380598 POSTN/COL3A1/FN1/FBLN2/LAMB2/GREM1/COL8A1/DDR1/ID1/JAG1/ANGPT1/CDH13/SLK 13

BP GO:0090132 epithelium migration 13/262 360/18862 0.001649667 0.072055839 0.061120227 GREM1/PTPRG/FAP/FGF18/ENPP2/ID1/ANGPT1/CDH13/CYP1B1/HMGB1/FGFBP1/ROBO1/PTPN11 13

BP GO:0032355 response to estradiol 7/262 125/18862 0.00182717 0.078455304 0.066548473 MBD4/PENK/POSTN/GJA1/TXNIP/BAD/TFPI 7

BP GO:0051054 positive regulation of DNA metabolic process 9/262 198/18862 0.001846701 0.078455304 0.066548473 RFC4/GREM1/ACTR2/CCT6A/HSP90AA1/PRKDC/HMGB1/HNRNPA1/CCT8 9

BP GO:0090130 tissue migration 13/262 365/18862 0.001862704 0.078455304 0.066548473 GREM1/PTPRG/FAP/FGF18/ENPP2/ID1/ANGPT1/CDH13/CYP1B1/HMGB1/FGFBP1/ROBO1/PTPN11 13

BP GO:0044786 cell cycle DNA replication 5/262 64/18862 0.001946303 0.081012001 0.06871715 RFC4/POLA1/MCM6/POLD3/PRIM1 5

BP GO:0097194 execution phase of apoptosis 6/262 94/18862 0.001991666 0.081936233 0.069501116 CASP4/FAP/BCL2L1/TOP2A/HMGB1/DICER1 6

BP GO:0051170 import into nucleus 8/262 163/18862 0.002034195 0.082506276 0.069984646 HTATIP2/TXNIP/IPO5/TNPO1/ANGPT1/NFKBIA/HNRNPA1/CHP1 8

BP GO:0071375 cellular response to peptide hormone stimulus 12/262 325/18862 0.002052163 0.082506276 0.069984646 ECHDC3/MYO1C/IGF1R/FYN/INSIG1/PRKAR1A/OSBPL8/CDC5L/FBN1/PRKDC/GDF15/PTPN11 12

BP GO:0032479 regulation of type I interferon production 7/262 128/18862 0.002091497 0.083142875 0.070524631 IFI16/POLA1/PCBP2/HSP90AA1/PRKDC/HMGB1/PTPN11 7

BP GO:0006271 DNA strand elongation involved in DNA replication 3/262 19/18862 0.002177503 0.08486391 0.071984471 RFC4/POLA1/POLD3 3

BP GO:0032606 type I interferon production 7/262 129/18862 0.002185874 0.08486391 0.071984471 IFI16/POLA1/PCBP2/HSP90AA1/PRKDC/HMGB1/PTPN11 7

BP GO:1904951 positive regulation of establishment of protein localization 12/262 328/18862 0.002213744 0.08486391 0.071984471 MYO1C/GJA1/PPID/CEMIP/FYN/IPO5/BAD/CCT6A/PLA2G6/SFN/CHP1/CCT8 12

BP GO:0061035 regulation of cartilage development 5/262 66/18862 0.002230736 0.08486391 0.071984471 GREM1/FGF18/EFEMP1/RARG/PTPN11 5

BP GO:0030336 negative regulation of cell migration 12/262 330/18862 0.002327139 0.085764751 0.072748596 SULF1/COL3A1/GJA1/GREM1/PTPRG/OSBPL8/JAG1/CYP1B1/CXCL12/HMGB1/ROBO1/PTPRU 12

BP GO:0071214 cellular response to abiotic stimulus 12/262 330/18862 0.002327139 0.085764751 0.072748596 PIEZO2/CUL4B/GJA1/PPID/IFI16/PKD2/BAD/MMP2/POLD3/CHP1/PTPN11/NPM1 12

BP GO:0104004 cellular response to environmental stimulus 12/262 330/18862 0.002327139 0.085764751 0.072748596 PIEZO2/CUL4B/GJA1/PPID/IFI16/PKD2/BAD/MMP2/POLD3/CHP1/PTPN11/NPM1 12

BP GO:1905314 semi-lunar valve development 4/262 41/18862 0.002460479 0.08910327 0.075580441 JAG1/RBPJ/SNAI1/ROBO1 4

BP GO:0040013 negative regulation of locomotion 13/262 377/18862 0.002468095 0.08910327 0.075580441 SULF1/COL3A1/GJA1/GREM1/PTPRG/PLXNA3/OSBPL8/JAG1/CYP1B1/CXCL12/HMGB1/ROBO1/PTPRU 13

BP GO:0050849 negative regulation of calcium-mediated signaling 3/262 20/18862 0.002535663 0.08977639 0.076151405 HOMER3/DYRK2/CHP1 3

BP GO:0033157 regulation of intracellular protein transport 10/262 248/18862 0.002538884 0.08977639 0.076151405 MYO1C/CEMIP/FYN/INSIG1/IPO5/BAD/ANGPT1/SFN/CHP1/PTPN11 10

BP GO:1903311 regulation of mRNA metabolic process 12/262 334/18862 0.00256818 0.08977639 0.076151405 SERBP1/CDC73/TNPO1/PAPOLA/PUM2/DYRK1A/PAIP1/HNRNPA1/SRSF10/RBM7/NPM1/CSDC2 12

BP GO:0019886 antigen processing and presentation of exogenous peptide antigen via MHC class II 6/262 99/18862 0.00258824 0.08977639 0.076151405 CAPZA1/AP1S2/KIF2A/SEC24D/CANX/HLA-DMA 6

BP GO:0001667 ameboidal-type cell migration 15/262 473/18862 0.002648705 0.090981735 0.07717382 FN1/GJA1/GREM1/PTPRG/FAP/FGF18/ENPP2/ID1/ANGPT1/CDH13/CYP1B1/HMGB1/FGFBP1/ROBO1/PTPN11 15

BP GO:0003197 endocardial cushion development 4/262 42/18862 0.002690196 0.091399135 0.077527873 JAG1/RBPJ/SNAI1/ROBO1 4

BP GO:0071229 cellular response to acid chemical 5/262 69/18862 0.002712524 0.091399135 0.077527873 PDGFC/FYN/IPO5/PKD2/MMP2 5

BP GO:0071560 cellular response to transforming growth factor beta stimulus 10/262 251/18862 0.002768312 0.092398947 0.078375948 USP15/PENK/POSTN/COL3A1/FYN/LTBP1/ID1/SMAD2/FBN1/GDF15 10

BP GO:0001890 placenta development 7/262 135/18862 0.002823145 0.093331397 0.079166884 GJA1/SP3/STC2/PKD2/RBPJ/SNAI1/KRT19 7

BP GO:0000079 regulation of cyclin-dependent protein serine/threonine kinase activity 6/262 101/18862 0.002861051 0.093331397 0.079166884 IPO5/CCND2/PKD2/CDKN1B/SFN/CCNG2 6

BP GO:0007162 negative regulation of cell adhesion 11/262 295/18862 0.002889448 0.093331397 0.079166884 POSTN/RUNX3/PRKAR1A/PLXNA3/JAG1/ANGPT1/CDH13/CYP1B1/CXCL12/HMGB1/PTPN11 11

BP GO:0003177 pulmonary valve development 3/262 21/18862 0.002928147 0.093331397 0.079166884 JAG1/RBPJ/ROBO1 3

BP GO:0046599 regulation of centriole replication 3/262 21/18862 0.002928147 0.093331397 0.079166884 VPS4B/TRIM37/NPM1 3

BP GO:0002495 antigen processing and presentation of peptide antigen via MHC class II 6/262 103/18862 0.003154833 0.099658932 0.084534115 CAPZA1/AP1S2/KIF2A/SEC24D/CANX/HLA-DMA 6

BP GO:0035987 endodermal cell differentiation 4/262 44/18862 0.003192891 0.099968574 0.084796765 FN1/CDC73/COL8A1/MMP2 4

BP GO:0002504 antigen processing and presentation of peptide or polysaccharide antigen via MHC class II 6/262 104/18862 0.003309893 0.100622603 0.085351534 CAPZA1/AP1S2/KIF2A/SEC24D/CANX/HLA-DMA 6

BP GO:2000146 negative regulation of cell motility 12/262 345/18862 0.003337651 0.100622603 0.085351534 SULF1/COL3A1/GJA1/GREM1/PTPRG/OSBPL8/JAG1/CYP1B1/CXCL12/HMGB1/ROBO1/PTPRU 12

BP GO:0032069 regulation of nuclease activity 3/262 22/18862 0.003355983 0.100622603 0.085351534 TCEA1/HMGB1/NPM1 3

BP GO:0036150 phosphatidylserine acyl-chain remodeling 3/262 22/18862 0.003355983 0.100622603 0.085351534 LPCAT3/OSBPL8/PLA2G4A 3

BP GO:1903077 negative regulation of protein localization to plasma membrane 3/262 22/18862 0.003355983 0.100622603 0.085351534 LRRC15/BCL2L1/LYPLA1 3

BP GO:0006283 transcription-coupled nucleotide-excision repair 5/262 73/18862 0.003466632 0.10232369 0.086794454 CUL4B/RFC4/TCEA1/POLD3/POLR2B 5

BP GO:1904029 regulation of cyclin-dependent protein kinase activity 6/262 105/18862 0.00347056 0.10232369 0.086794454 IPO5/CCND2/PKD2/CDKN1B/SFN/CCNG2 6

BP GO:0010810 regulation of cell-substrate adhesion 9/262 218/18862 0.003520656 0.102653109 0.087073879 POSTN/FN1/FBLN2/GREM1/COL8A1/DDR1/JAG1/CDH13/SLK 9

BP GO:0090068 positive regulation of cell cycle process 11/262 303/18862 0.003539762 0.102653109 0.087073879 GPSM2/CDC73/VPS4B/CCND2/FAP/PKD2/CDKN1B/CDC27/RAD51C/SFN/NPM1 11

BP GO:0032386 regulation of intracellular transport 12/262 350/18862 0.003744229 0.107699858 0.091354704 MYO1C/CEMIP/FYN/INSIG1/IPO5/RHOT1/BAD/ANGPT1/EHD1/SFN/CHP1/PTPN11 12

BP GO:1901989 positive regulation of cell cycle phase transition 6/262 107/18862 0.003809212 0.108685406 0.09219068 CDC73/VPS4B/CCND2/CDC27/RAD51C/NPM1 6

BP GO:2001020 regulation of response to DNA damage stimulus 9/262 221/18862 0.003850961 0.108997611 0.092455503 TRIP12/BCL2L1/DYRK1A/ACTR2/BCLAF1/PRKDC/SNAI1/CXCL12/HMGB1 9

BP GO:0046902 regulation of mitochondrial membrane permeability 5/262 75/18862 0.003895341 0.109138786 0.092575252 BCL2L1/RHOT1/BAD/SFN/ATF2 5

BP GO:0051271 negative regulation of cellular component movement 12/262 352/18862 0.003917644 0.109138786 0.092575252 SULF1/COL3A1/GJA1/GREM1/PTPRG/OSBPL8/JAG1/CYP1B1/CXCL12/HMGB1/ROBO1/PTPRU 12

BP GO:0007492 endoderm development 5/262 76/18862 0.004123341 0.113971716 0.096674708 FN1/CDC73/COL8A1/SMAD2/MMP2 5

BP GO:0001503 ossification 13/262 401/18862 0.004164814 0.11402297 0.096718184 PENK/RUNX3/SP3/FGFR3/GREM1/FGF18/CSGALNACT1/JAG1/CDH11/MMP2/RBPJ/SNAI1/PTPN11 13

BP GO:0003272 endocardial cushion formation 3/262 24/18862 0.004321497 0.11402297 0.096718184 RBPJ/SNAI1/ROBO1 3

BP GO:0006929 substrate-dependent cell migration 3/262 24/18862 0.004321497 0.11402297 0.096718184 CSPG4/FN1/ROBO1 3

BP GO:1904376 negative regulation of protein localization to cell periphery 3/262 24/18862 0.004321497 0.11402297 0.096718184 LRRC15/BCL2L1/LYPLA1 3

BP GO:0032481 positive regulation of type I interferon production 5/262 77/18862 0.004360715 0.11402297 0.096718184 IFI16/HSP90AA1/PRKDC/HMGB1/PTPN11 5

BP GO:0051205 protein insertion into membrane 5/262 77/18862 0.004360715 0.11402297 0.096718184 TRAM2/HSPA4/BAD/SFN/HSP90AA1 5

BP GO:0042149 cellular response to glucose starvation 4/262 48/18862 0.00438302 0.11402297 0.096718184 IFI16/PIK3R4/PIK3C3/HNRNPA1 4

BP GO:0051204 protein insertion into mitochondrial membrane 4/262 48/18862 0.00438302 0.11402297 0.096718184 HSPA4/BAD/SFN/HSP90AA1 4

BP GO:0000077 DNA damage checkpoint 7/262 148/18862 0.004685196 0.120175016 0.10193656 WAC/CDKN1B/CDC5L/SFN/PRKDC/ATF2/PTPN11 7

BP GO:0050982 detection of mechanical stimulus 4/262 49/18862 0.004721404 0.120175016 0.10193656 PIEZO2/FYN/PKD2/CXCL12 4

BP GO:0072132 mesenchyme morphogenesis 4/262 49/18862 0.004721404 0.120175016 0.10193656 SMAD2/RBPJ/SNAI1/ROBO1 4

BP GO:1903828 negative regulation of cellular protein localization 6/262 112/18862 0.004762609 0.120357932 0.102091716 LRRC15/INSIG1/BCL2L1/LYPLA1/ANGPT1/CHP1 6

BP GO:0001892 embryonic placenta development 5/262 79/18862 0.004864414 0.121694273 0.103225245 SP3/PKD2/RBPJ/SNAI1/KRT19 5

BP GO:0001933 negative regulation of protein phosphorylation 12/262 362/18862 0.004884281 0.121694273 0.103225245 BDKRB1/IGF1R/PRKAR1A/IPO5/GREM1/DNAJC10/CDKN1B/ANGPT1/SFN/PRKDC/CHP1/NPM1 12

BP GO:0007566 embryo implantation 4/262 50/18862 0.005076914 0.125609231 0.106546047 GJA1/STC2/DDR1/MMP2 4

BP GO:0048568 embryonic organ development 13/262 412/18862 0.005208251 0.125776817 0.106688199 PDGFC/SP3/INSIG1/DLX2/PRRX1/PKD2/SMAD2/EFEMP1/FBN1/RARG/RBPJ/SNAI1/KRT19 13

BP GO:0000082 G1/S transition of mitotic cell cycle 10/262 275/18862 0.005260865 0.125776817 0.106688199 CUL4B/CDC73/CCND2/POLA1/MCM6/PKD2/CDKN1B/SFN/PRKDC/PRIM1 10

BP GO:0034504 protein localization to nucleus 10/262 275/18862 0.005260865 0.125776817 0.106688199 TXNIP/FYN/IPO5/TNPO1/OSBPL8/ANGPT1/CCT6A/NFKBIA/CHP1/CCT8 10

BP GO:0018904 ether metabolic process 3/262 26/18862 0.00543919 0.125776817 0.106688199 EPHX1/PLA2G6/PLA2G4A 3

BP GO:0022616 DNA strand elongation 3/262 26/18862 0.00543919 0.125776817 0.106688199 RFC4/POLA1/POLD3 3

BP GO:0033522 histone H2A ubiquitination 3/262 26/18862 0.00543919 0.125776817 0.106688199 CUL4B/TRIP12/TRIM37 3

BP GO:0034162 toll-like receptor 9 signaling pathway 3/262 26/18862 0.00543919 0.125776817 0.106688199 PIK3R4/PIK3C3/HMGB1 3

BP GO:0036152 phosphatidylethanolamine acyl-chain remodeling 3/262 26/18862 0.00543919 0.125776817 0.106688199 LPCAT3/PLA2G6/PLA2G4A 3

BP GO:0051016 barbed-end actin filament capping 3/262 26/18862 0.00543919 0.125776817 0.106688199 EPS8/CAPZA1/TWF1 3

BP GO:2000810 regulation of bicellular tight junction assembly 3/262 26/18862 0.00543919 0.125776817 0.106688199 MYO1C/GJA1/SNAI1 3

BP GO:0048015 phosphatidylinositol-mediated signaling 8/262 192/18862 0.005503758 0.12644347 0.107253677 PDGFC/FN1/IGF1R/FYN/PPP2R5C/PIK3C3/ANGPT1/PI4KB 8

BP GO:0043542 endothelial cell migration 10/262 278/18862 0.00566762 0.128346342 0.108867758 GREM1/FAP/FGF18/ID1/ANGPT1/CDH13/CYP1B1/HMGB1/FGFBP1/ROBO1 10

BP GO:0050731 positive regulation of peptidyl-tyrosine phosphorylation 8/262 193/18862 0.005674751 0.128346342 0.108867758 CSPG4/FGFR3/FYN/GREM1/ENPP2/ANGPT1/TNK2/PTPN11 8

BP GO:2001021 negative regulation of response to DNA damage stimulus 5/262 82/18862 0.005695414 0.128346342 0.108867758 TRIP12/BCL2L1/DYRK1A/SNAI1/CXCL12 5

BP GO:0030509 BMP signaling pathway 7/262 154/18862 0.005804812 0.129352955 0.109721602 SULF1/USP15/GREM1/SMAD2/FBN1/RBPJ/GDF15 7

BP GO:0060350 endochondral bone morphogenesis 4/262 52/18862 0.005840821 0.129352955 0.109721602 FGFR3/FGF18/CSGALNACT1/RARG 4

BP GO:0017038 protein import 8/262 194/18862 0.005849766 0.129352955 0.109721602 TXNIP/IPO5/TNPO1/HSPA4/ANGPT1/HSP90AA1/NFKBIA/CHP1 8

BP GO:0007143 female meiotic nuclear division 3/262 27/18862 0.006057033 0.132282616 0.112206641 PRKAR1A/TOP2A/RAD51C 3

BP GO:0090025 regulation of monocyte chemotaxis 3/262 27/18862 0.006057033 0.132282616 0.112206641 GREM1/CXCL12/HMGB1 3

BP GO:0008637 apoptotic mitochondrial changes 6/262 118/18862 0.006125756 0.132494554 0.112386413 BCL2L1/RHOT1/BAD/ATG3/SFN/ATF2 6

BP GO:0048017 inositol lipid-mediated signaling 8/262 196/18862 0.006212099 0.132494554 0.112386413 PDGFC/FN1/IGF1R/FYN/PPP2R5C/PIK3C3/ANGPT1/PI4KB 8

BP GO:0031570 DNA integrity checkpoint 7/262 156/18862 0.006219144 0.132494554 0.112386413 WAC/CDKN1B/CDC5L/SFN/PRKDC/ATF2/PTPN11 7

BP GO:0090151 establishment of protein localization to mitochondrial membrane 4/262 53/18862 0.006249955 0.132494554 0.112386413 HSPA4/BAD/SFN/HSP90AA1 4

BP GO:0016049 cell growth 14/262 470/18862 0.006253983 0.132494554 0.112386413 BDKRB1/POSTN/FN1/GJA1/CDC73/LAMB2/GREM1/PLXNA3/CDKN1B/DDR1/SFN/HSP90AA1/RARG/CXCL12 14

BP GO:0001958 endochondral ossification 3/262 28/18862 0.006715129 0.138936408 0.117850614 FGFR3/FGF18/CSGALNACT1 3

BP GO:0036075 replacement ossification 3/262 28/18862 0.006715129 0.138936408 0.117850614 FGFR3/FGF18/CSGALNACT1 3

BP GO:0072376 protein activation cascade 3/262 28/18862 0.006715129 0.138936408 0.117850614 FN1/F13A1/TFPI 3

BP GO:0072378 "blood coagulation, fibrin clot formation" 3/262 28/18862 0.006715129 0.138936408 0.117850614 FN1/F13A1/TFPI 3

BP GO:0000819 sister chromatid segregation 8/262 199/18862 0.006787254 0.139612236 0.118423874 VPS4B/TOP2A/NSL1/PDS5B/STAG2/CDC27/RAD51C/PDS5A 8

BP GO:0007389 pattern specification process 13/262 426/18862 0.006829281 0.139664723 0.118468396 RFNG/BICC1/DLX2/GREM1/PKD2/BARX1/SMAD2/RARG/RBPJ/PRKDC/SNAI1/HOXA10/ROBO1 13

BP GO:0048013 ephrin receptor signaling pathway 5/262 86/18862 0.00695275 0.141372581 0.119917059 FYN/ACTR2/MMP2/RBPJ/PTPN11 5

BP GO:0050680 negative regulation of epithelial cell proliferation 7/262 160/18862 0.007113498 0.143216065 0.121480766 SULF1/GJA1/RUNX3/CDC73/SFN/ATF2/ROBO1 7

BP GO:0035904 aorta development 4/262 55/18862 0.007124372 0.143216065 0.121480766 PKD2/JAG1/RBPJ/ROBO1 4

BP GO:0002063 chondrocyte development 3/262 29/18862 0.007414101 0.146542398 0.124302275 SULF1/FGF18/RARG 3

BP GO:0061311 cell surface receptor signaling pathway involved in heart development 3/262 29/18862 0.007414101 0.146542398 0.124302275 JAG1/RBPJ/SNAI1 3

BP GO:0072337 modified amino acid transport 3/262 29/18862 0.007414101 0.146542398 0.124302275 ABCC1/GJA1/SLC6A8 3

BP GO:0031050 dsRNA processing 4/262 56/18862 0.007590343 0.147197483 0.12485794 TSN/PUM2/SMAD2/DICER1 4

BP GO:0070918 production of small RNA involved in gene silencing by RNA 4/262 56/18862 0.007590343 0.147197483 0.12485794 TSN/PUM2/SMAD2/DICER1 4

BP GO:0034644 cellular response to UV 5/262 88/18862 0.007648985 0.147197483 0.12485794 CUL4B/PPID/MMP2/POLD3/NPM1 5

BP GO:0034250 positive regulation of cellular amide metabolic process 7/262 163/18862 0.007844373 0.147197483 0.12485794 RMND1/NSMAF/UPF3A/PAIP1/IFNGR1/PRKDC/NPM1 7

BP GO:0032070 regulation of deoxyribonuclease activity 2/262 10/18862 0.008036185 0.147197483 0.12485794 HMGB1/NPM1 2

BP GO:0035860 glial cell-derived neurotrophic factor receptor signaling pathway 2/262 10/18862 0.008036185 0.147197483 0.12485794 SULF1/GDF15 2

BP GO:1904851 positive regulation of establishment of protein localization to telomere 2/262 10/18862 0.008036185 0.147197483 0.12485794 CCT6A/CCT8 2

BP GO:2000343 positive regulation of chemokine (C-X-C motif) ligand 2 production 2/262 10/18862 0.008036185 0.147197483 0.12485794 POSTN/HMGB1 2

BP GO:0001952 regulation of cell-matrix adhesion 6/262 125/18862 0.008051949 0.147197483 0.12485794 POSTN/GREM1/DDR1/JAG1/CDH13/SLK 6

BP GO:0045747 positive regulation of Notch signaling pathway 4/262 57/18862 0.008075935 0.147197483 0.12485794 RFNG/JAG1/RBPJ/ROBO1 4

BP GO:0048008 platelet-derived growth factor receptor signaling pathway 4/262 57/18862 0.008075935 0.147197483 0.12485794 PDGFC/CSPG4/TXNIP/PTPN11 4

BP GO:2000242 negative regulation of reproductive process 4/262 57/18862 0.008075935 0.147197483 0.12485794 SULF1/GJA1/PRKAR1A/SNAI1 4

BP GO:1990138 neuron projection extension 7/262 164/18862 0.008099893 0.147197483 0.12485794 POSTN/FN1/LAMB2/PLXNA3/DDR1/HSP90AA1/CXCL12 7

BP GO:0036151 phosphatidylcholine acyl-chain remodeling 3/262 30/18862 0.008154524 0.147197483 0.12485794 LPCAT3/PLA2G6/PLA2G4A 3

BP GO:1902230 negative regulation of intrinsic apoptotic signaling pathway in response to DNA damage 3/262 30/18862 0.008154524 0.147197483 0.12485794 BCL2L1/SNAI1/CXCL12 3

BP GO:1902751 positive regulation of cell cycle G2/M phase transition 3/262 30/18862 0.008154524 0.147197483 0.12485794 VPS4B/RAD51C/NPM1 3

BP GO:1905476 negative regulation of protein localization to membrane 3/262 30/18862 0.008154524 0.147197483 0.12485794 LRRC15/BCL2L1/LYPLA1 3

BP GO:0014068 positive regulation of phosphatidylinositol 3-kinase signaling 5/262 90/18862 0.008392327 0.147594772 0.125194935 PDGFC/FN1/IGF1R/FYN/ANGPT1 5

BP GO:0060349 bone morphogenesis 5/262 90/18862 0.008392327 0.147594772 0.125194935 FGFR3/INSIG1/FGF18/CSGALNACT1/RARG 5

BP GO:0090559 regulation of membrane permeability 5/262 90/18862 0.008392327 0.147594772 0.125194935 BCL2L1/RHOT1/BAD/SFN/ATF2 5

BP GO:1903829 positive regulation of cellular protein localization 10/262 295/18862 0.008457555 0.147594772 0.125194935 MYO1C/GPSM2/CEMIP/FYN/IPO5/BAD/CCT6A/SFN/CHP1/CCT8 10

BP GO:0035265 organ growth 7/262 166/18862 0.008629274 0.147594772 0.125194935 GJA1/FGFR3/PRKAR1A/SMAD2/RARG/RBPJ/PTPN11 7

BP GO:0034976 response to endoplasmic reticulum stress 10/262 296/18862 0.008649601 0.147594772 0.125194935 CASP4/LPCAT3/DNAJC10/BCL2L1/CTH/TMEM259/CANX/HSP90B1/UBXN4/FBXO2 10

BP GO:0090287 regulation of cellular response to growth factor stimulus 10/262 296/18862 0.008649601 0.147594772 0.125194935 SULF1/MYO1C/LTBP1/GREM1/FGF18/SMAD2/FBN1/RBPJ/FGFBP1/ROBO1 10

BP GO:0014066 regulation of phosphatidylinositol 3-kinase signaling 6/262 127/18862 0.008674239 0.147594772 0.125194935 PDGFC/FN1/IGF1R/FYN/PPP2R5C/ANGPT1 6

BP GO:1905477 positive regulation of protein localization to membrane 6/262 127/18862 0.008674239 0.147594772 0.125194935 MYO1C/CEMIP/FYN/BAD/SFN/CHP1 6

BP GO:0030510 regulation of BMP signaling pathway 5/262 91/18862 0.008782137 0.147594772 0.125194935 SULF1/GREM1/SMAD2/FBN1/RBPJ 5

BP GO:0071772 response to BMP 7/262 167/18862 0.008903302 0.147594772 0.125194935 SULF1/USP15/GREM1/SMAD2/FBN1/RBPJ/GDF15 7

BP GO:0071773 cellular response to BMP stimulus 7/262 167/18862 0.008903302 0.147594772 0.125194935 SULF1/USP15/GREM1/SMAD2/FBN1/RBPJ/GDF15 7

BP GO:0003180 aortic valve morphogenesis 3/262 31/18862 0.008936924 0.147594772 0.125194935 JAG1/SNAI1/ROBO1 3

BP GO:0060317 cardiac epithelial to mesenchymal transition 3/262 31/18862 0.008936924 0.147594772 0.125194935 JAG1/RBPJ/SNAI1 3

BP GO:0002576 platelet degranulation 6/262 128/18862 0.008997981 0.147594772 0.125194935 PCYOX1L/FN1/TIMP3/F13A1/PHACTR2/OLA1 6

BP GO:0000075 cell cycle checkpoint 8/262 209/18862 0.008998425 0.147594772 0.125194935 WAC/BCL2L1/CDKN1B/CDC5L/SFN/PRKDC/ATF2/PTPN11 8

BP GO:0051651 maintenance of location in cell 8/262 209/18862 0.008998425 0.147594772 0.125194935 BDKRB1/GJA1/GPSM2/CEMIP/INSIG1/PKD2/TWF1/HSP90B1 8

BP GO:0044843 cell cycle G1/S phase transition 10/262 298/18862 0.009043733 0.147594772 0.125194935 CUL4B/CDC73/CCND2/POLA1/MCM6/PKD2/CDKN1B/SFN/PRKDC/PRIM1 10

BP GO:0045787 positive regulation of cell cycle 12/262 395/18862 0.009490137 0.147594772 0.125194935 GPSM2/CDC73/VPS4B/CCND2/FAP/PKD2/CDKN1B/CDC27/RAD51C/SFN/PTPN11/NPM1 12

BP GO:0006612 protein targeting to membrane 8/262 211/18862 0.009498574 0.147594772 0.125194935 SRP72/MYO1C/TRAM2/CEMIP/FYN/SRP9/ATG3/CHP1 8

BP GO:1902110 positive regulation of mitochondrial membrane permeability involved in apoptotic process 4/262 60/18862 0.009653635 0.147594772 0.125194935 RHOT1/BAD/SFN/ATF2 4

BP GO:2001244 positive regulation of intrinsic apoptotic signaling pathway 4/262 60/18862 0.009653635 0.147594772 0.125194935 SIAH1/BCL2L1/BAD/BCLAF1 4

BP GO:1900180 regulation of protein localization to nucleus 6/262 130/18862 0.009671251 0.147594772 0.125194935 FYN/IPO5/ANGPT1/CCT6A/CHP1/CCT8 6

BP GO:0030210 heparin biosynthetic process 2/262 11/18862 0.009732549 0.147594772 0.125194935 CSGALNACT1/ANGPT1 2

BP GO:0030422 production of siRNA involved in RNA interference 2/262 11/18862 0.009732549 0.147594772 0.125194935 TSN/DICER1 2

BP GO:0032836 glomerular basement membrane development 2/262 11/18862 0.009732549 0.147594772 0.125194935 SULF1/LAMB2 2

BP GO:0045602 negative regulation of endothelial cell differentiation 2/262 11/18862 0.009732549 0.147594772 0.125194935 ID1/JAG1 2

BP GO:0060379 cardiac muscle cell myoblast differentiation 2/262 11/18862 0.009732549 0.147594772 0.125194935 GREM1/RBPJ 2

BP GO:0070203 regulation of establishment of protein localization to telomere 2/262 11/18862 0.009732549 0.147594772 0.125194935 CCT6A/CCT8 2

BP GO:0071492 cellular response to UV-A 2/262 11/18862 0.009732549 0.147594772 0.125194935 PPID/MMP2 2

BP GO:0072070 loop of Henle development 2/262 11/18862 0.009732549 0.147594772 0.125194935 PKD2/JAG1 2

BP GO:0075522 IRES-dependent viral translational initiation 2/262 11/18862 0.009732549 0.147594772 0.125194935 PCBP2/CSDE1 2

BP GO:1904869 regulation of protein localization to Cajal body 2/262 11/18862 0.009732549 0.147594772 0.125194935 CCT6A/CCT8 2

BP GO:1904871 positive regulation of protein localization to Cajal body 2/262 11/18862 0.009732549 0.147594772 0.125194935 CCT6A/CCT8 2

BP GO:0009954 proximal/distal pattern formation 3/262 32/18862 0.00976178 0.147594772 0.125194935 DLX2/GREM1/HOXA10 3

BP GO:0046475 glycerophospholipid catabolic process 3/262 32/18862 0.00976178 0.147594772 0.125194935 ENPP2/PLA2G6/PLA2G4A 3

BP GO:0050974 detection of mechanical stimulus involved in sensory perception 3/262 32/18862 0.00976178 0.147594772 0.125194935 PIEZO2/FYN/CXCL12 3

BP GO:0070884 regulation of calcineurin-NFAT signaling cascade 3/262 32/18862 0.00976178 0.147594772 0.125194935 HOMER3/DYRK2/CHP1 3

BP GO:0042770 signal transduction in response to DNA damage 6/262 131/18862 0.010021017 0.150869608 0.127972762 CDKN1B/DYRK1A/CDC5L/SFN/PRKDC/SNAI1 6

BP GO:0071496 cellular response to external stimulus 10/262 303/18862 0.01008934 0.15125459 0.128299318 PIEZO2/PENK/POSTN/GJA1/IFI16/PIK3R4/PIK3C3/BAD/HNRNPA1/PTPN11 10

BP GO:0071230 cellular response to amino acid stimulus 4/262 61/18862 0.010220863 0.152210447 0.129110109 PDGFC/FYN/IPO5/MMP2 4

BP GO:0060840 artery development 5/262 95/18862 0.010466592 0.152210447 0.129110109 PRRX1/PKD2/JAG1/RBPJ/ROBO1 5

BP GO:0003203 endocardial cushion morphogenesis 3/262 33/18862 0.010629524 0.152210447 0.129110109 RBPJ/SNAI1/ROBO1 3

BP GO:0030262 apoptotic nuclear changes 3/262 33/18862 0.010629524 0.152210447 0.129110109 TOP2A/HMGB1/DICER1 3

BP GO:0032212 positive regulation of telomere maintenance via telomerase 3/262 33/18862 0.010629524 0.152210447 0.129110109 CCT6A/HNRNPA1/CCT8 3

BP GO:0032728 positive regulation of interferon-beta production 3/262 33/18862 0.010629524 0.152210447 0.129110109 HSP90AA1/HMGB1/PTPN11 3

BP GO:0106056 regulation of calcineurin-mediated signaling 3/262 33/18862 0.010629524 0.152210447 0.129110109 HOMER3/DYRK2/CHP1 3

BP GO:0006027 glycosaminoglycan catabolic process 4/262 62/18862 0.010809245 0.152210447 0.129110109 CSPG4/CEMIP/SDC3/CHP1 4

BP GO:0007062 sister chromatid cohesion 4/262 62/18862 0.010809245 0.152210447 0.129110109 PDS5B/STAG2/RAD51C/PDS5A 4

BP GO:0031122 cytoplasmic microtubule organization 4/262 62/18862 0.010809245 0.152210447 0.129110109 VPS4B/CRIPT/SLK/CHP1 4

BP GO:0032835 glomerulus development 4/262 62/18862 0.010809245 0.152210447 0.129110109 SULF1/LAMB2/JAG1/ANGPT1 4

BP GO:1902686 mitochondrial outer membrane permeabilization involved in programmed cell death 4/262 62/18862 0.010809245 0.152210447 0.129110109 RHOT1/BAD/SFN/ATF2 4

BP GO:0050730 regulation of peptidyl-tyrosine phosphorylation 9/262 262/18862 0.01125378 0.152210447 0.129110109 PDGFC/CSPG4/FGFR3/FYN/GREM1/ENPP2/ANGPT1/TNK2/PTPN11 9

BP GO:0043534 blood vessel endothelial cell migration 7/262 175/18862 0.011330502 0.152210447 0.129110109 GREM1/FGF18/ID1/ANGPT1/HMGB1/FGFBP1/ROBO1 7

BP GO:0048608 reproductive structure development 12/262 405/18862 0.011405134 0.152210447 0.129110109 SULF1/GJA1/SP3/STC2/PKD2/CSDE1/RARG/RBPJ/SNAI1/HOXA10/KRT19/PTPN11 12

BP GO:0044783 G1 DNA damage checkpoint 4/262 63/18862 0.011419059 0.152210447 0.129110109 WAC/CDKN1B/SFN/PRKDC 4

BP GO:0030513 positive regulation of BMP signaling pathway 3/262 34/18862 0.011540545 0.152210447 0.129110109 SULF1/SMAD2/RBPJ 3

BP GO:0009886 post-embryonic animal morphogenesis 2/262 12/18862 0.011572812 0.152210447 0.129110109 EFEMP1/FBN1 2

BP GO:0010826 negative regulation of centrosome duplication 2/262 12/18862 0.011572812 0.152210447 0.129110109 TRIM37/NPM1 2

BP GO:0030202 heparin metabolic process 2/262 12/18862 0.011572812 0.152210447 0.129110109 CSGALNACT1/ANGPT1 2

BP GO:0033523 histone H2B ubiquitination 2/262 12/18862 0.011572812 0.152210447 0.129110109 CDC73/WAC 2

BP GO:0033629 negative regulation of cell adhesion mediated by integrin 2/262 12/18862 0.011572812 0.152210447 0.129110109 CYP1B1/PTPN11 2

BP GO:0035672 oligopeptide transmembrane transport 2/262 12/18862 0.011572812 0.152210447 0.129110109 ABCC1/GJA1 2

BP GO:0042308 negative regulation of protein import into nucleus 2/262 12/18862 0.011572812 0.152210447 0.129110109 ANGPT1/CHP1 2

BP GO:0046606 negative regulation of centrosome cycle 2/262 12/18862 0.011572812 0.152210447 0.129110109 TRIM37/NPM1 2

BP GO:0061430 bone trabecula morphogenesis 2/262 12/18862 0.011572812 0.152210447 0.129110109 GREM1/MMP2 2

BP GO:0070202 regulation of establishment of protein localization to chromosome 2/262 12/18862 0.011572812 0.152210447 0.129110109 CCT6A/CCT8 2

BP GO:0072015 glomerular visceral epithelial cell development 2/262 12/18862 0.011572812 0.152210447 0.129110109 LAMB2/JAG1 2

BP GO:0072017 distal tubule development 2/262 12/18862 0.011572812 0.152210447 0.129110109 PKD2/JAG1 2

BP GO:1903405 protein localization to nuclear body 2/262 12/18862 0.011572812 0.152210447 0.129110109 CCT6A/CCT8 2

BP GO:1904590 negative regulation of protein import 2/262 12/18862 0.011572812 0.152210447 0.129110109 ANGPT1/CHP1 2

BP GO:1904816 "positive regulation of protein localization to chromosome, telomeric region" 2/262 12/18862 0.011572812 0.152210447 0.129110109 CCT6A/CCT8 2

BP GO:1904867 protein localization to Cajal body 2/262 12/18862 0.011572812 0.152210447 0.129110109 CCT6A/CCT8 2

BP GO:0032388 positive regulation of intracellular transport 8/262 219/18862 0.011708388 0.153423242 0.130138842 MYO1C/CEMIP/FYN/IPO5/BAD/EHD1/SFN/CHP1 8

BP GO:0042326 negative regulation of phosphorylation 12/262 407/18862 0.011821573 0.154334784 0.130912043 BDKRB1/IGF1R/PRKAR1A/IPO5/GREM1/DNAJC10/CDKN1B/ANGPT1/SFN/PRKDC/CHP1/NPM1 12

BP GO:0071902 positive regulation of protein serine/threonine kinase activity 10/262 311/18862 0.011951032 0.15545129 0.131859102 PDGFC/CSPG4/IGFBP6/CEMIP/CCND2/FGF18/PKD2/GDF15/ROBO1/PTPN11 10

BP GO:0061458 reproductive system development 12/262 408/18862 0.012034138 0.155601961 0.131986906 SULF1/GJA1/SP3/STC2/PKD2/CSDE1/RARG/RBPJ/SNAI1/HOXA10/KRT19/PTPN11 12

BP GO:0035794 positive regulation of mitochondrial membrane permeability 4/262 64/18862 0.012050576 0.155601961 0.131986906 RHOT1/BAD/SFN/ATF2 4

BP GO:0051222 positive regulation of protein transport 10/262 312/18862 0.012200854 0.156969526 0.133146922 MYO1C/GJA1/PPID/CEMIP/FYN/IPO5/BAD/PLA2G6/SFN/CHP1 10

BP GO:0003158 endothelium development 6/262 137/18862 0.012310745 0.157809479 0.133859399 VEZF1/GJA1/ID1/JAG1/COL15A1/RBPJ 6

BP GO:0006921 cellular component disassembly involved in execution phase of apoptosis 3/262 35/18862 0.012495189 0.15902151 0.134887485 TOP2A/HMGB1/DICER1 3

BP GO:0018149 peptide cross-linking 3/262 35/18862 0.012495189 0.15902151 0.134887485 COL3A1/FN1/F13A1 3

BP GO:0033002 muscle cell proliferation 8/262 222/18862 0.012627679 0.159088552 0.134944352 GJA1/PRKAR1A/CDKN1B/ANGPT1/CDH13/MMP2/RBPJ/PRKDC 8

BP GO:0048588 developmental cell growth 8/262 222/18862 0.012627679 0.159088552 0.134944352 POSTN/FN1/LAMB2/PLXNA3/DDR1/HSP90AA1/RARG/CXCL12 8

BP GO:1904888 cranial skeletal system development 4/262 65/18862 0.012704057 0.159088552 0.134944352 INSIG1/DLX2/PRRX1/SMAD2 4

BP GO:0090316 positive regulation of intracellular protein transport 7/262 179/18862 0.012709231 0.159088552 0.134944352 MYO1C/CEMIP/FYN/IPO5/BAD/SFN/CHP1 7

BP GO:0072006 nephron development 6/262 138/18862 0.012725286 0.159088552 0.134944352 SULF1/LAMB2/GREM1/PKD2/JAG1/ANGPT1 6

BP GO:0050684 regulation of mRNA processing 6/262 139/18862 0.013149529 0.163093166 0.138341202 CDC73/PAPOLA/DYRK1A/HNRNPA1/SRSF10/RBM7 6

BP GO:0051146 striated muscle cell differentiation 9/262 269/18862 0.013185429 0.163093166 0.138341202 PRKAR1A/MEF2A/GREM1/EHD1/POPDC3/RBPJ/GDF15/PLD3/KRT19 9

BP GO:0006401 RNA catabolic process 12/262 414/18862 0.013371949 0.163093166 0.138341202 HBS1L/SERBP1/TNPO1/PUM2/UPF3A/CSDE1/PAIP1/ISG20/LRPPRC/RBM7/NPM1/CSDC2 12

BP GO:0006513 protein monoubiquitination 4/262 66/18862 0.013379756 0.163093166 0.138341202 CUL4B/CDC73/WAC/TRIM37 4

BP GO:1900076 regulation of cellular response to insulin stimulus 4/262 66/18862 0.013379756 0.163093166 0.138341202 ECHDC3/MYO1C/OSBPL8/PTPN11 4

BP GO:1902108 regulation of mitochondrial membrane permeability involved in apoptotic process 4/262 66/18862 0.013379756 0.163093166 0.138341202 RHOT1/BAD/SFN/ATF2 4

BP GO:1904358 positive regulation of telomere maintenance via telomere lengthening 3/262 36/18862 0.013493762 0.163093166 0.138341202 CCT6A/HNRNPA1/CCT8 3

BP GO:0006857 oligopeptide transport 2/262 13/18862 0.013552683 0.163093166 0.138341202 ABCC1/GJA1 2

BP GO:0070914 UV-damage excision repair 2/262 13/18862 0.013552683 0.163093166 0.138341202 CUL4B/POLD3 2

BP GO:0071236 cellular response to antibiotic 2/262 13/18862 0.013552683 0.163093166 0.138341202 ACTR2/PLA2G4A 2

BP GO:0072310 glomerular epithelial cell development 2/262 13/18862 0.013552683 0.163093166 0.138341202 LAMB2/JAG1 2

BP GO:0060560 developmental growth involved in morphogenesis 8/262 225/18862 0.013598904 0.163094653 0.138342464 POSTN/FN1/LAMB2/PLXNA3/DDR1/HSP90AA1/RARG/CXCL12 8

BP GO:0060249 anatomical structure homeostasis 13/262 466/18862 0.013735482 0.16417613 0.13925981 RFC4/GJA1/POLA1/RAD51C/ANGPT1/CCT6A/HSP90AA1/PRKDC/POLD3/PRIM1/HNRNPA1/CCT8/PTPN11 13

BP GO:0036503 ERAD pathway 5/262 102/18862 0.013923541 0.165307009 0.14021906 DNAJC10/TMEM259/HSP90B1/UBXN4/FBXO2 5

BP GO:0043200 response to amino acid 5/262 102/18862 0.013923541 0.165307009 0.14021906 PDGFC/FYN/IPO5/BAD/MMP2 5

BP GO:0050848 regulation of calcium-mediated signaling 4/262 67/18862 0.014077913 0.166025524 0.140828529 HOMER3/DYRK2/CDH13/CHP1 4

BP GO:0050918 positive chemotaxis 4/262 67/18862 0.014077913 0.166025524 0.140828529 ANGPT1/CDH13/CXCL12/HMGB1 4

BP GO:0001655 urogenital system development 10/262 320/18862 0.014343864 0.168072677 0.142564994 SULF1/BICC1/LAMB2/GREM1/PKD2/SMAD2/JAG1/ANGPT1/FBN1/RARG 10

BP GO:0098813 nuclear chromosome segregation 9/262 273/18862 0.014394309 0.168072677 0.142564994 VPS4B/TOP2A/NSL1/PDS5B/STAG2/CDC27/RAD51C/ACTR2/PDS5A 9

BP GO:0046822 regulation of nucleocytoplasmic transport 5/262 103/18862 0.014472965 0.168072677 0.142564994 IPO5/ANGPT1/SFN/CHP1/PTPN11 5

BP GO:0006296 "nucleotide-excision repair, DNA incision, 5'-to lesion" 3/262 37/18862 0.014536529 0.168072677 0.142564994 CUL4B/RFC4/POLD3 3

BP GO:0006658 phosphatidylserine metabolic process 3/262 37/18862 0.014536529 0.168072677 0.142564994 LPCAT3/OSBPL8/PLA2G4A 3

BP GO:1902229 regulation of intrinsic apoptotic signaling pathway in response to DNA damage 3/262 37/18862 0.014536529 0.168072677 0.142564994 BCL2L1/SNAI1/CXCL12 3

BP GO:0001707 mesoderm formation 4/262 68/18862 0.014798764 0.169443456 0.143727736 GJA1/PRKAR1A/SMAD2/SNAI1 4

BP GO:0006026 aminoglycan catabolic process 4/262 68/18862 0.014798764 0.169443456 0.143727736 CSPG4/CEMIP/SDC3/CHP1 4

BP GO:0007004 telomere maintenance via telomerase 4/262 68/18862 0.014798764 0.169443456 0.143727736 CCT6A/HSP90AA1/HNRNPA1/CCT8 4

BP GO:0006606 protein import into nucleus 6/262 143/18862 0.014945711 0.170025483 0.144221431 TXNIP/IPO5/TNPO1/ANGPT1/NFKBIA/CHP1 6

BP GO:0048592 eye morphogenesis 6/262 143/18862 0.014945711 0.170025483 0.144221431 SP3/COL8A1/JAG1/EFEMP1/FBN1/RARG 6

BP GO:0006986 response to unfolded protein 7/262 185/18862 0.01499811 0.170074724 0.144263199 CTH/HSPA13/HSPA4/CANX/HSP90AA1/HSP90B1/UBXN4 7

BP GO:0007160 cell-matrix adhesion 8/262 230/18862 0.015337335 0.170206859 0.144375281 POSTN/COL3A1/FN1/GREM1/DDR1/JAG1/CDH13/SLK 8

BP GO:1905710 positive regulation of membrane permeability 4/262 69/18862 0.015542533 0.170206859 0.144375281 RHOT1/BAD/SFN/ATF2 4

BP GO:0006298 mismatch repair 3/262 38/18862 0.015623716 0.170206859 0.144375281 MSH6/POLD3/HMGB1 3

BP GO:0046326 positive regulation of glucose import 3/262 38/18862 0.015623716 0.170206859 0.144375281 MEF2A/OSBPL8/PTPN11 3

BP GO:0009415 response to water 2/262 14/18862 0.015667956 0.170206859 0.144375281 PKD2/ATF2 2

BP GO:0035437 maintenance of protein localization in endoplasmic reticulum 2/262 14/18862 0.015667956 0.170206859 0.144375281 GJA1/INSIG1 2

BP GO:0044804 autophagy of nucleus 2/262 14/18862 0.015667956 0.170206859 0.144375281 ATG12/ATG3 2

BP GO:0045898 regulation of RNA polymerase II transcription preinitiation complex assembly 2/262 14/18862 0.015667956 0.170206859 0.144375281 CAND1/HMGB1 2

BP GO:0048569 post-embryonic animal organ development 2/262 14/18862 0.015667956 0.170206859 0.144375281 EFEMP1/FBN1 2

BP GO:0050966 detection of mechanical stimulus involved in sensory perception of pain 2/262 14/18862 0.015667956 0.170206859 0.144375281 FYN/CXCL12 2

BP GO:0070141 response to UV-A 2/262 14/18862 0.015667956 0.170206859 0.144375281 PPID/MMP2 2

BP GO:1904814 "regulation of protein localization to chromosome, telomeric region" 2/262 14/18862 0.015667956 0.170206859 0.144375281 CCT6A/CCT8 2

BP GO:1990173 protein localization to nucleoplasm 2/262 14/18862 0.015667956 0.170206859 0.144375281 CCT6A/CCT8 2

BP GO:0048562 embryonic organ morphogenesis 9/262 277/18862 0.015683278 0.170206859 0.144375281 SP3/INSIG1/DLX2/PRRX1/PKD2/SMAD2/EFEMP1/FBN1/RARG 9

BP GO:0038127 ERBB signaling pathway 6/262 145/18862 0.015904813 0.172083268 0.145966915 EPS15/CDH13/EFEMP1/HSP90AA1/RBPJ/PTPN11 6

BP GO:0043161 proteasome-mediated ubiquitin-dependent protein catabolic process 12/262 425/18862 0.016116835 0.172905005 0.14666394 CUL4B/SIAH1/FBXL5/CSNK1A1/WAC/DNAJC10/PCBP2/PPP2R5C/CDC27/HSP90B1/UBXN4/FBXO2 12

BP GO:0045785 positive regulation of cell adhesion 12/262 425/18862 0.016116835 0.172905005 0.14666394 FN1/RUNX3/FBLN2/FYN/COL8A1/BAD/ANGPT1/CDH13/CXCL12/HMGB1/PTPRU/PTPN11 12

BP GO:0003002 regionalization 10/262 326/18862 0.016127375 0.172905005 0.14666394 DLX2/GREM1/BARX1/SMAD2/RARG/RBPJ/PRKDC/SNAI1/HOXA10/ROBO1 10

BP GO:0048332 mesoderm morphogenesis 4/262 70/18862 0.016309434 0.173197455 0.146912006 GJA1/PRKAR1A/SMAD2/SNAI1 4

BP GO:0048844 artery morphogenesis 4/262 70/18862 0.016309434 0.173197455 0.146912006 PRRX1/PKD2/JAG1/RBPJ 4

BP GO:0009411 response to UV 6/262 146/18862 0.016399981 0.173197455 0.146912006 CUL4B/PPID/MSH6/MMP2/POLD3/NPM1 6

BP GO:0019827 stem cell population maintenance 6/262 146/18862 0.016399981 0.173197455 0.146912006 CDC73/PRRX1/SMAD2/JAG1/RBPJ/POLR2B 6

BP GO:0006457 protein folding 8/262 233/18862 0.016454779 0.173197455 0.146912006 PPID/DNAJC10/HSPA13/CANX/CCT6A/HSP90AA1/HSP90B1/CCT8 8

BP GO:0001822 kidney development 9/262 280/18862 0.016704306 0.173197455 0.146912006 SULF1/BICC1/LAMB2/GREM1/PKD2/SMAD2/JAG1/ANGPT1/FBN1 9

BP GO:0033683 "nucleotide-excision repair, DNA incision" 3/262 39/18862 0.016755512 0.173197455 0.146912006 CUL4B/RFC4/POLD3 3

BP GO:0042769 "DNA damage response, detection of DNA damage" 3/262 39/18862 0.016755512 0.173197455 0.146912006 CUL4B/RFC4/POLD3 3

BP GO:0072210 metanephric nephron development 3/262 39/18862 0.016755512 0.173197455 0.146912006 LAMB2/GREM1/PKD2 3

BP GO:0032147 activation of protein kinase activity 10/262 328/18862 0.016757012 0.173197455 0.146912006 PDGFC/CSPG4/IGFBP6/PRKAR1A/GREM1/OSBPL8/ANGPT1/GDF15/HMGB1/PTPN11 10

BP GO:0032984 protein-containing complex disassembly 10/262 328/18862 0.016757012 0.173197455 0.146912006 MRPS10/EPS8/CAPZA1/CSNK1A1/IGF1R/SMARCE1/KIF2A/VPS4B/TWF1/MRPL19 10

BP GO:0006024 glycosaminoglycan biosynthetic process 5/262 107/18862 0.016815828 0.173197455 0.146912006 CSPG4/CEMIP/SDC3/CSGALNACT1/ANGPT1 5

BP GO:0048640 negative regulation of developmental growth 5/262 107/18862 0.016815828 0.173197455 0.146912006 GJA1/FGFR3/PLXNA3/CDKN1B/GDF15 5

BP GO:0006403 RNA localization 8/262 234/18862 0.016840001 0.173197455 0.146912006 MYO1C/UPF3A/XPOT/CCT6A/HNRNPA1/LRPPRC/CCT8/NPM1 8

BP GO:0098586 cellular response to virus 4/262 71/18862 0.017099674 0.174305382 0.147851788 USP15/PENK/PUM2/HSP90AA1 4

BP GO:0002218 activation of innate immune response 6/262 148/18862 0.017422053 0.174305382 0.147851788 FYN/IFI16/MATR3/HSP90AA1/PRKDC/HMGB1 6

BP GO:0098727 maintenance of cell number 6/262 148/18862 0.017422053 0.174305382 0.147851788 CDC73/PRRX1/SMAD2/JAG1/RBPJ/POLR2B 6

BP GO:0051656 establishment of organelle localization 12/262 431/18862 0.017783391 0.174305382 0.147851788 WDR11/MYO1C/GJA1/GPSM2/VPS4B/RHOT1/SEC24D/ACTR2/CNIH1/LRPPRC/CHP1/NPM1 12

BP GO:0014855 striated muscle cell proliferation 4/262 72/18862 0.017913449 0.174305382 0.147851788 GJA1/PRKAR1A/ANGPT1/RBPJ 4

BP GO:0051298 centrosome duplication 4/262 72/18862 0.017913449 0.174305382 0.147851788 VPS4B/PKD2/TRIM37/NPM1 4

BP GO:0006266 DNA ligation 2/262 15/18862 0.017914519 0.174305382 0.147851788 TOP2A/HMGB1 2

BP GO:0032933 SREBP signaling pathway 2/262 15/18862 0.017914519 0.174305382 0.147851788 LPCAT3/INSIG1 2

BP GO:0042994 cytoplasmic sequestering of transcription factor 2/262 15/18862 0.017914519 0.174305382 0.147851788 PKD2/NFKBIA 2

BP GO:0043518 "negative regulation of DNA damage response, signal transduction by p53 class mediator" 2/262 15/18862 0.017914519 0.174305382 0.147851788 DYRK1A/SNAI1 2

BP GO:0050651 dermatan sulfate proteoglycan biosynthetic process 2/262 15/18862 0.017914519 0.174305382 0.147851788 CSPG4/CSGALNACT1 2

BP GO:0090110 COPII-coated vesicle cargo loading 2/262 15/18862 0.017914519 0.174305382 0.147851788 INSIG1/SEC24D 2

BP GO:0097202 activation of cysteine-type endopeptidase activity 2/262 15/18862 0.017914519 0.174305382 0.147851788 IFI16/BAD 2

BP GO:1901741 positive regulation of myoblast fusion 2/262 15/18862 0.017914519 0.174305382 0.147851788 EHD1/GDF15 2

BP GO:1904874 positive regulation of telomerase RNA localization to Cajal body 2/262 15/18862 0.017914519 0.174305382 0.147851788 CCT6A/CCT8 2

BP GO:0006270 DNA replication initiation 3/262 40/18862 0.017932071 0.174305382 0.147851788 POLA1/MCM6/PRIM1 3

BP GO:0006308 DNA catabolic process 3/262 40/18862 0.017932071 0.174305382 0.147851788 HMGB1/ISG20/DICER1 3

BP GO:0051281 positive regulation of release of sequestered calcium ion into cytosol 3/262 40/18862 0.017932071 0.174305382 0.147851788 BDKRB1/CEMIP/PKD2 3

BP GO:0071634 regulation of transforming growth factor beta production 3/262 40/18862 0.017932071 0.174305382 0.147851788 FN1/LTBP1/ATF2 3

BP GO:0010498 proteasomal protein catabolic process 13/262 483/18862 0.017933058 0.174305382 0.147851788 CUL4B/SIAH1/FBXL5/CSNK1A1/WAC/DNAJC10/TMEM259/PCBP2/PPP2R5C/CDC27/HSP90B1/UBXN4/FBXO2 13

BP GO:0032963 collagen metabolic process 5/262 109/18862 0.018076392 0.175217187 0.148625212 TRAM2/FAP/P3H3/COL15A1/MMP2 5

BP GO:0003151 outflow tract morphogenesis 4/262 73/18862 0.018750945 0.179785487 0.1525002 JAG1/RBPJ/ATF2/ROBO1 4

BP GO:0006809 nitric oxide biosynthetic process 4/262 73/18862 0.018750945 0.179785487 0.1525002 PKD2/GCHFR/HSP90AA1/CYP1B1 4

BP GO:0072401 signal transduction involved in DNA integrity checkpoint 4/262 73/18862 0.018750945 0.179785487 0.1525002 CDKN1B/CDC5L/SFN/PRKDC 4

BP GO:0072422 signal transduction involved in DNA damage checkpoint 4/262 73/18862 0.018750945 0.179785487 0.1525002 CDKN1B/CDC5L/SFN/PRKDC 4

BP GO:0001701 in utero embryonic development 10/262 335/18862 0.019104774 0.180226404 0.152874202 SP3/PKD2/EDNRA/SEC24D/SMAD2/ANGPT1/RBPJ/SNAI1/PSPH/KRT19 10

BP GO:0001709 cell fate determination 3/262 41/18862 0.019153513 0.180226404 0.152874202 PRRX1/IFRD1/JAG1 3

BP GO:0007099 centriole replication 3/262 41/18862 0.019153513 0.180226404 0.152874202 VPS4B/TRIM37/NPM1 3

BP GO:0033173 calcineurin-NFAT signaling cascade 3/262 41/18862 0.019153513 0.180226404 0.152874202 HOMER3/DYRK2/CHP1 3

BP GO:0051693 actin filament capping 3/262 41/18862 0.019153513 0.180226404 0.152874202 EPS8/CAPZA1/TWF1 3

BP GO:0090279 regulation of calcium ion import 3/262 41/18862 0.019153513 0.180226404 0.152874202 FYN/PKD2/CXCL12 3

BP GO:0090317 negative regulation of intracellular protein transport 3/262 41/18862 0.019153513 0.180226404 0.152874202 INSIG1/ANGPT1/CHP1 3

BP GO:0000280 nuclear division 12/262 436/18862 0.019268601 0.18040986 0.153029815 EPS8/PRKAR1A/KIF2A/VPS4B/TOP2A/NSL1/PDS5B/STAG2/CDC27/RAD51C/ACTR2/PDS5A 12

BP GO:0060485 mesenchyme development 9/262 287/18862 0.019274994 0.18040986 0.153029815 FN1/GREM1/PKD2/EDNRA/SMAD2/JAG1/RBPJ/SNAI1/ROBO1 9

BP GO:0071260 cellular response to mechanical stimulus 4/262 74/18862 0.019612342 0.183083022 0.155297282 PIEZO2/GJA1/BAD/PTPN11 4

BP GO:0072001 renal system development 9/262 288/18862 0.019664396 0.18308588 0.155299706 SULF1/BICC1/LAMB2/GREM1/PKD2/SMAD2/JAG1/ANGPT1/FBN1 9

BP GO:0007059 chromosome segregation 10/262 337/18862 0.019817892 0.184030718 0.15610115 VPS4B/TOP2A/PUM2/NSL1/PDS5B/STAG2/CDC27/RAD51C/ACTR2/PDS5A 10

BP GO:0051261 protein depolymerization 5/262 112/18862 0.020081738 0.184524843 0.156520284 EPS8/CAPZA1/KIF2A/VPS4B/TWF1 5

BP GO:0003198 epithelial to mesenchymal transition involved in endocardial cushion formation 2/262 16/18862 0.020288345 0.184524843 0.156520284 RBPJ/SNAI1 2

BP GO:0019081 viral translation 2/262 16/18862 0.020288345 0.184524843 0.156520284 PCBP2/CSDE1 2

BP GO:0033151 V(D)J recombination 2/262 16/18862 0.020288345 0.184524843 0.156520284 PRKDC/HMGB1 2

BP GO:0050655 dermatan sulfate proteoglycan metabolic process 2/262 16/18862 0.020288345 0.184524843 0.156520284 CSPG4/CSGALNACT1 2

BP GO:0051709 regulation of killing of cells of other organism 2/262 16/18862 0.020288345 0.184524843 0.156520284 BCL2L1/BAD 2

BP GO:0071501 cellular response to sterol depletion 2/262 16/18862 0.020288345 0.184524843 0.156520284 LPCAT3/INSIG1 2

BP GO:1902969 mitotic DNA replication 2/262 16/18862 0.020288345 0.184524843 0.156520284 POLA1/MCM6 2

BP GO:0062208 positive regulation of pattern recognition receptor signaling pathway 3/262 42/18862 0.020419922 0.184532417 0.156526709 USP15/PUM2/HMGB1 3

BP GO:0071604 transforming growth factor beta production 3/262 42/18862 0.020419922 0.184532417 0.156526709 FN1/LTBP1/ATF2 3

BP GO:0006278 RNA-dependent DNA biosynthetic process 4/262 75/18862 0.020497807 0.184532417 0.156526709 CCT6A/HSP90AA1/HNRNPA1/CCT8 4

BP GO:0043536 positive regulation of blood vessel endothelial cell migration 4/262 75/18862 0.020497807 0.184532417 0.156526709 FGF18/ANGPT1/HMGB1/FGFBP1 4

BP GO:0014065 phosphatidylinositol 3-kinase signaling 6/262 154/18862 0.020749172 0.186321247 0.158044056 PDGFC/FN1/IGF1R/FYN/PPP2R5C/ANGPT1 6

BP GO:0006605 protein targeting 12/262 441/18862 0.020844797 0.186706054 0.158370462 SRP72/MYO1C/TRAM2/CEMIP/FYN/HOMER3/PIK3R4/HSPA4/SRP9/ATG3/HSP90AA1/CHP1 12

BP GO:0090596 sensory organ morphogenesis 8/262 244/18862 0.021056879 0.188129393 0.159577787 SP3/INSIG1/PRRX1/COL8A1/JAG1/EFEMP1/FBN1/RARG 8

BP GO:1901342 regulation of vasculature development 10/262 341/18862 0.021302396 0.189662064 0.16087785 SULF1/HTATIP2/MTDH/GREM1/FGF18/ENPP2/ID1/CYP1B1/HMGB1/ATF2 10

BP GO:0072395 signal transduction involved in cell cycle checkpoint 4/262 76/18862 0.021407499 0.189662064 0.16087785 CDKN1B/CDC5L/SFN/PRKDC 4

BP GO:0006023 aminoglycan biosynthetic process 5/262 114/18862 0.021496463 0.189662064 0.16087785 CSPG4/CEMIP/SDC3/CSGALNACT1/ANGPT1 5

BP GO:0032392 DNA geometric change 5/262 114/18862 0.021496463 0.189662064 0.16087785 CUL4B/RFC4/MCM6/TOP2A/HMGB1 5

BP GO:0048675 axon extension 5/262 114/18862 0.021496463 0.189662064 0.16087785 FN1/LAMB2/PLXNA3/HSP90AA1/CXCL12 5

BP GO:0009069 serine family amino acid metabolic process 3/262 43/18862 0.021731351 0.189840794 0.161029456 CTH/THNSL2/PSPH 3

BP GO:0046677 response to antibiotic 3/262 43/18862 0.021731351 0.189840794 0.161029456 ACTR2/PLA2G4A/HSP90AA1 3

BP GO:0061383 trabecula morphogenesis 3/262 43/18862 0.021731351 0.189840794 0.161029456 GREM1/MMP2/RBPJ 3

BP GO:2000142 "regulation of DNA-templated transcription, initiation" 3/262 43/18862 0.021731351 0.189840794 0.161029456 CAND1/BCLAF1/HMGB1 3

BP GO:1903510 mucopolysaccharide metabolic process 5/262 115/18862 0.022227538 0.192859031 0.163589628 CSPG4/CEMIP/CSGALNACT1/ANGPT1/CHP1 5

BP GO:0010827 regulation of glucose transmembrane transport 4/262 77/18862 0.02234157 0.192859031 0.163589628 MEF2A/OSBPL8/EDNRA/PTPN11 4

BP GO:0046209 nitric oxide metabolic process 4/262 77/18862 0.02234157 0.192859031 0.163589628 PKD2/GCHFR/HSP90AA1/CYP1B1 4

BP GO:0031346 positive regulation of cell projection organization 10/262 344/18862 0.022467847 0.192859031 0.163589628 EPS8/FN1/FYN/VLDLR/PLXNA3/ENPP2/TWF1/ACTR2/CXCL12/ROBO1 10

BP GO:0030168 platelet activation 6/262 157/18862 0.022564088 0.192859031 0.163589628 COL3A1/FN1/FYN/PLA2G4A/MYL12A/PTPN11 6

BP GO:0003184 pulmonary valve morphogenesis 2/262 17/18862 0.022785493 0.192859031 0.163589628 JAG1/ROBO1 2

BP GO:0010715 regulation of extracellular matrix disassembly 2/262 17/18862 0.022785493 0.192859031 0.163589628 FAP/DDR1 2

BP GO:0016246 RNA interference 2/262 17/18862 0.022785493 0.192859031 0.163589628 TSN/DICER1 2

BP GO:0035518 histone H2A monoubiquitination 2/262 17/18862 0.022785493 0.192859031 0.163589628 CUL4B/TRIM37 2

BP GO:0042474 middle ear morphogenesis 2/262 17/18862 0.022785493 0.192859031 0.163589628 INSIG1/PRRX1 2

BP GO:0071850 mitotic cell cycle arrest 2/262 17/18862 0.022785493 0.192859031 0.163589628 FAP/CDKN1B 2

BP GO:0072567 chemokine (C-X-C motif) ligand 2 production 2/262 17/18862 0.022785493 0.192859031 0.163589628 POSTN/HMGB1 2

BP GO:2000341 regulation of chemokine (C-X-C motif) ligand 2 production 2/262 17/18862 0.022785493 0.192859031 0.163589628 POSTN/HMGB1 2

BP GO:0007179 transforming growth factor beta receptor signaling pathway 7/262 202/18862 0.023054906 0.193058458 0.163758788 USP15/COL3A1/LTBP1/ID1/SMAD2/FBN1/GDF15 7

BP GO:0010824 regulation of centrosome duplication 3/262 44/18862 0.023087824 0.193058458 0.163758788 VPS4B/TRIM37/NPM1 3

BP GO:0060324 face development 3/262 44/18862 0.023087824 0.193058458 0.163758788 MMP2/RARG/PTPN11 3

BP GO:0030203 glycosaminoglycan metabolic process 6/262 158/18862 0.023192073 0.193058458 0.163758788 CSPG4/CEMIP/SDC3/CSGALNACT1/ANGPT1/CHP1 6

BP GO:0051494 negative regulation of cytoskeleton organization 6/262 158/18862 0.023192073 0.193058458 0.163758788 EPS8/CAPZA1/DYRK1A/TWF1/TRIM37/NPM1 6

BP GO:0010639 negative regulation of organelle organization 10/262 346/18862 0.023270122 0.193058458 0.163758788 EPS8/CAPZA1/PRKAR1A/BCL2L1/TOP2A/DYRK1A/TWF1/HNRNPA1/TRIM37/NPM1 10

BP GO:0032272 negative regulation of protein polymerization 4/262 78/18862 0.023300159 0.193058458 0.163758788 EPS8/CAPZA1/DYRK1A/TWF1 4

BP GO:0050772 positive regulation of axonogenesis 4/262 78/18862 0.023300159 0.193058458 0.163758788 FN1/PLXNA3/CXCL12/ROBO1 4

BP GO:2001057 reactive nitrogen species metabolic process 4/262 78/18862 0.023300159 0.193058458 0.163758788 PKD2/GCHFR/HSP90AA1/CYP1B1 4

BP GO:0048706 embryonic skeletal system development 5/262 117/18862 0.023737683 0.196224121 0.166444012 SULF1/SP3/DLX2/PRRX1/SMAD2 5

BP GO:0048145 regulation of fibroblast proliferation 4/262 79/18862 0.024283398 0.196490358 0.166669843 PDGFC/FN1/CDC73/PRKDC 4

BP GO:0070830 bicellular tight junction assembly 4/262 79/18862 0.024283398 0.196490358 0.166669843 MTDH/MYO1C/GJA1/SNAI1 4

BP GO:0045165 cell fate commitment 8/262 251/18862 0.024422328 0.196490358 0.166669843 CDC73/DLX2/PRRX1/IFRD1/SMAD2/JAG1/RBPJ/PRKDC 8

BP GO:2001242 regulation of intrinsic apoptotic signaling pathway 6/262 160/18862 0.024483062 0.196490358 0.166669843 SIAH1/BCL2L1/BAD/BCLAF1/SNAI1/CXCL12 6

BP GO:0010828 positive regulation of glucose transmembrane transport 3/262 45/18862 0.024489332 0.196490358 0.166669843 MEF2A/OSBPL8/PTPN11 3

BP GO:0030835 negative regulation of actin filament depolymerization 3/262 45/18862 0.024489332 0.196490358 0.166669843 EPS8/CAPZA1/TWF1 3

BP GO:0045601 regulation of endothelial cell differentiation 3/262 45/18862 0.024489332 0.196490358 0.166669843 VEZF1/ID1/JAG1 3

BP GO:0048701 embryonic cranial skeleton morphogenesis 3/262 45/18862 0.024489332 0.196490358 0.166669843 DLX2/PRRX1/SMAD2 3

BP GO:0098534 centriole assembly 3/262 45/18862 0.024489332 0.196490358 0.166669843 VPS4B/TRIM37/NPM1 3

BP GO:0045931 positive regulation of mitotic cell cycle 5/262 118/18862 0.024516972 0.196490358 0.166669843 VPS4B/CCND2/CDC27/RAD51C/PTPN11 5

BP GO:0031396 regulation of protein ubiquitination 7/262 205/18862 0.02473625 0.196490358 0.166669843 FYN/TRIP12/ANGPT1/HSP90AA1/DCUN1D4/FBXO2/CHP1 7

BP GO:0006446 regulation of translational initiation 4/262 80/18862 0.025291412 0.196490358 0.166669843 MTIF2/CSDE1/PAIP1/NPM1 4

BP GO:0010833 telomere maintenance via telomere lengthening 4/262 80/18862 0.025291412 0.196490358 0.166669843 CCT6A/HSP90AA1/HNRNPA1/CCT8 4

BP GO:0030433 ubiquitin-dependent ERAD pathway 4/262 80/18862 0.025291412 0.196490358 0.166669843 DNAJC10/HSP90B1/UBXN4/FBXO2 4

BP GO:0045445 myoblast differentiation 4/262 80/18862 0.025291412 0.196490358 0.166669843 GREM1/IFRD1/JAG1/RBPJ 4

BP GO:0048144 fibroblast proliferation 4/262 80/18862 0.025291412 0.196490358 0.166669843 PDGFC/FN1/CDC73/PRKDC 4

BP GO:0045446 endothelial cell differentiation 5/262 119/18862 0.025312545 0.196490358 0.166669843 VEZF1/ID1/JAG1/COL15A1/RBPJ 5

BP GO:2000134 negative regulation of G1/S transition of mitotic cell cycle 5/262 119/18862 0.025312545 0.196490358 0.166669843 CDC73/PKD2/CDKN1B/SFN/PRKDC 5

BP GO:0035966 response to topologically incorrect protein 7/262 206/18862 0.025314921 0.196490358 0.166669843 CTH/HSPA13/HSPA4/CANX/HSP90AA1/HSP90B1/UBXN4 7

BP GO:0003128 heart field specification 2/262 18/18862 0.025402107 0.196490358 0.166669843 RBPJ/ROBO1 2

BP GO:0006991 response to sterol depletion 2/262 18/18862 0.025402107 0.196490358 0.166669843 LPCAT3/INSIG1 2

BP GO:0019054 modulation by virus of host cellular process 2/262 18/18862 0.025402107 0.196490358 0.166669843 BCL2L1/BAD 2

BP GO:0030214 hyaluronan catabolic process 2/262 18/18862 0.025402107 0.196490358 0.166669843 CEMIP/CHP1 2

BP GO:0032048 cardiolipin metabolic process 2/262 18/18862 0.025402107 0.196490358 0.166669843 PLA2G6/PLA2G4A 2

BP GO:0045116 protein neddylation 2/262 18/18862 0.025402107 0.196490358 0.166669843 UBA3/DCUN1D4 2

BP GO:0070200 establishment of protein localization to telomere 2/262 18/18862 0.025402107 0.196490358 0.166669843 CCT6A/CCT8 2

BP GO:0070875 positive regulation of glycogen metabolic process 2/262 18/18862 0.025402107 0.196490358 0.166669843 DYRK2/HMGB1 2

BP GO:1901739 regulation of myoblast fusion 2/262 18/18862 0.025402107 0.196490358 0.166669843 EHD1/GDF15 2

BP GO:1904872 regulation of telomerase RNA localization to Cajal body 2/262 18/18862 0.025402107 0.196490358 0.166669843 CCT6A/CCT8 2

BP GO:0015931 nucleobase-containing compound transport 8/262 253/18862 0.025449384 0.196490358 0.166669843 MYO1C/GJA1/UPF3A/XPOT/SLC25A36/HNRNPA1/LRPPRC/NPM1 8

BP GO:0002683 negative regulation of immune system process 11/262 403/18862 0.025789163 0.196490358 0.166669843 COL3A1/RUNX3/PRKAR1A/IFI16/GREM1/PCBP2/ANGPT1/FBN1/PRKDC/CXCL12/HMGB1 11

BP GO:0002064 epithelial cell development 7/262 207/18862 0.025902805 0.196490358 0.166669843 VEZF1/LAMB2/BAD/JAG1/SFN/COL15A1/RARG 7

BP GO:0034764 positive regulation of transmembrane transport 7/262 207/18862 0.025902805 0.196490358 0.166669843 BDKRB1/CEMIP/MEF2A/PKD2/OSBPL8/CHP1/PTPN11 7

BP GO:0034767 positive regulation of ion transmembrane transport 7/262 207/18862 0.025902805 0.196490358 0.166669843 BDKRB1/CEMIP/MEF2A/PKD2/OSBPL8/CHP1/PTPN11 7

BP GO:0031648 protein destabilization 3/262 46/18862 0.025935839 0.196490358 0.166669843 SIAH1/CDC73/PRKDC 3

BP GO:0048146 positive regulation of fibroblast proliferation 3/262 46/18862 0.025935839 0.196490358 0.166669843 PDGFC/FN1/PRKDC 3

BP GO:0048483 autonomic nervous system development 3/262 46/18862 0.025935839 0.196490358 0.166669843 FN1/PLXNA3/EDNRA 3

BP GO:0050798 activated T cell proliferation 3/262 46/18862 0.025935839 0.196490358 0.166669843 FYN/PRKAR1A/HMGB1 3

BP GO:0097720 calcineurin-mediated signaling 3/262 46/18862 0.025935839 0.196490358 0.166669843 HOMER3/DYRK2/CHP1 3

BP GO:0021954 central nervous system neuron development 4/262 81/18862 0.026324312 0.198160458 0.168086479 PLXNA3/HSP90AA1/CDH11/ROBO1 4

BP GO:0120192 tight junction assembly 4/262 81/18862 0.026324312 0.198160458 0.168086479 MTDH/MYO1C/GJA1/SNAI1 4

BP GO:1903533 regulation of protein targeting 4/262 81/18862 0.026324312 0.198160458 0.168086479 MYO1C/CEMIP/FYN/CHP1 4

BP GO:0002221 pattern recognition receptor signaling pathway 7/262 208/18862 0.026499963 0.199059167 0.168848795 USP15/PUM2/PIK3R4/PIK3C3/HSP90B1/NFKBIA/HMGB1 7

BP GO:0022604 regulation of cell morphogenesis 9/262 305/18862 0.027185747 0.203347086 0.172485955 POSTN/EPS8/FN1/FYN/ZMYM4/PLXNA3/ENPP2/ACTR2/PRKDC 9

BP GO:0090305 nucleic acid phosphodiester bond hydrolysis 9/262 305/18862 0.027185747 0.203347086 0.172485955 CUL4B/RFC4/TSN/ENPP2/POLD3/HMGB1/ISG20/PLD3/DICER1 9

BP GO:0001656 metanephros development 4/262 82/18862 0.027382204 0.203433364 0.172559138 LAMB2/GREM1/PKD2/FBN1 4

BP GO:0071158 positive regulation of cell cycle arrest 4/262 82/18862 0.027382204 0.203433364 0.172559138 FAP/PKD2/CDKN1B/SFN 4

BP GO:0030574 collagen catabolic process 3/262 47/18862 0.027427279 0.203433364 0.172559138 FAP/COL15A1/MMP2 3

BP GO:0051646 mitochondrion localization 3/262 47/18862 0.027427279 0.203433364 0.172559138 MEF2A/RHOT1/LRPPRC 3

BP GO:0140053 mitochondrial gene expression 6/262 165/18862 0.027918515 0.204494569 0.173459289 MRPS10/RMND1/MTIF2/MRPL19/LRPPRC/TFAM 6

BP GO:0010002 cardioblast differentiation 2/262 19/18862 0.028134414 0.204494569 0.173459289 GREM1/RBPJ 2

BP GO:0039535 regulation of RIG-I signaling pathway 2/262 19/18862 0.028134414 0.204494569 0.173459289 USP15/PUM2 2

BP GO:0046485 ether lipid metabolic process 2/262 19/18862 0.028134414 0.204494569 0.173459289 PLA2G6/PLA2G4A 2

BP GO:0090670 RNA localization to Cajal body 2/262 19/18862 0.028134414 0.204494569 0.173459289 CCT6A/CCT8 2

BP GO:0090671 telomerase RNA localization to Cajal body 2/262 19/18862 0.028134414 0.204494569 0.173459289 CCT6A/CCT8 2

BP GO:0090672 telomerase RNA localization 2/262 19/18862 0.028134414 0.204494569 0.173459289 CCT6A/CCT8 2

BP GO:0090685 RNA localization to nucleus 2/262 19/18862 0.028134414 0.204494569 0.173459289 CCT6A/CCT8 2

BP GO:0097150 neuronal stem cell population maintenance 2/262 19/18862 0.028134414 0.204494569 0.173459289 PRRX1/JAG1 2

BP GO:0002833 positive regulation of response to biotic stimulus 8/262 258/18862 0.028148348 0.204494569 0.173459289 USP15/FYN/IFI16/PUM2/MATR3/HSP90AA1/PRKDC/HMGB1 8

BP GO:0045069 regulation of viral genome replication 4/262 83/18862 0.028465184 0.206372584 0.175052286 PPID/IFI16/TOP2A/ISG20 4

BP GO:0007178 transmembrane receptor protein serine/threonine kinase signaling pathway 10/262 358/18862 0.028524717 0.206381288 0.175059668 SULF1/USP15/COL3A1/LTBP1/GREM1/ID1/SMAD2/FBN1/RBPJ/GDF15 10

BP GO:0051224 negative regulation of protein transport 5/262 123/18862 0.028659623 0.20642675 0.175098231 INSIG1/LYPLA1/ANGPT1/CHP1/PTPN11 5

BP GO:1903409 reactive oxygen species biosynthetic process 5/262 123/18862 0.028659623 0.20642675 0.175098231 FYN/PKD2/GCHFR/HSP90AA1/CYP1B1 5

BP GO:0035850 epithelial cell differentiation involved in kidney development 3/262 48/18862 0.028963563 0.20642675 0.175098231 LAMB2/GREM1/JAG1 3

BP GO:0046605 regulation of centrosome cycle 3/262 48/18862 0.028963563 0.20642675 0.175098231 VPS4B/TRIM37/NPM1 3

BP GO:1990090 cellular response to nerve growth factor stimulus 3/262 48/18862 0.028963563 0.20642675 0.175098231 ID1/CDC5L/EHD1 3

BP GO:0030178 negative regulation of Wnt signaling pathway 7/262 212/18862 0.028982482 0.20642675 0.175098231 CSNK1A1/IGFBP6/BICC1/TLE2/GREM1/BARX1/PTPRU 7

BP GO:0001654 eye development 10/262 359/18862 0.028997766 0.20642675 0.175098231 SP3/LAMB2/DLX2/COL8A1/JAG1/EFEMP1/CYP1B1/FBN1/RARG/HMGB1 10

BP GO:0009615 response to virus 10/262 359/18862 0.028997766 0.20642675 0.175098231 USP15/PENK/IFI16/BCL2L1/PUM2/PCBP2/HSP90AA1/IFNGR1/CXCL12/ISG20 10

BP GO:0030323 respiratory tube development 6/262 167/18862 0.02937746 0.208709743 0.177034743 SP3/HECA/FGF18/SMAD2/RBPJ/HMGB1 6

BP GO:1902807 negative regulation of cell cycle G1/S phase transition 5/262 124/18862 0.029538046 0.209260947 0.177502294 CDC73/PKD2/CDKN1B/SFN/PRKDC 5

BP GO:0120193 tight junction organization 4/262 84/18862 0.029573339 0.209260947 0.177502294 MTDH/MYO1C/GJA1/SNAI1 4

BP GO:0043010 camera-type eye development 9/262 310/18862 0.029738878 0.210012276 0.178139596 SP3/LAMB2/DLX2/COL8A1/JAG1/EFEMP1/CYP1B1/FBN1/RARG 9

BP GO:0031349 positive regulation of defense response 10/262 361/18862 0.02996056 0.211156296 0.179109993 ABCC1/PENK/GJA1/FYN/IFI16/MATR3/HSP90AA1/NFKBIA/PRKDC/HMGB1 10

BP GO:0042692 muscle cell differentiation 10/262 362/18862 0.030450366 0.211588243 0.179476386 PRKAR1A/MEF2A/GREM1/CTH/EHD1/POPDC3/RBPJ/GDF15/PLD3/KRT19 10

BP GO:0009395 phospholipid catabolic process 3/262 49/18862 0.030544574 0.211588243 0.179476386 ENPP2/PLA2G6/PLA2G4A 3

BP GO:0021879 forebrain neuron differentiation 3/262 49/18862 0.030544574 0.211588243 0.179476386 DLX2/PLXNA3/ROBO1 3

BP GO:1900182 positive regulation of protein localization to nucleus 4/262 85/18862 0.030706748 0.211588243 0.179476386 FYN/IPO5/CCT6A/CCT8 4

BP GO:0150063 visual system development 10/262 363/18862 0.030945818 0.211588243 0.179476386 SP3/LAMB2/DLX2/COL8A1/JAG1/EFEMP1/CYP1B1/FBN1/RARG/HMGB1 10

BP GO:0030220 platelet formation 2/262 20/18862 0.03097872 0.211588243 0.179476386 PRKDC/PTPN11 2

BP GO:0032495 response to muramyl dipeptide 2/262 20/18862 0.03097872 0.211588243 0.179476386 JAG1/NFKBIA 2

BP GO:0046697 decidualization 2/262 20/18862 0.03097872 0.211588243 0.179476386 GJA1/STC2 2

BP GO:0046823 negative regulation of nucleocytoplasmic transport 2/262 20/18862 0.03097872 0.211588243 0.179476386 ANGPT1/CHP1 2

BP GO:0046885 regulation of hormone biosynthetic process 2/262 20/18862 0.03097872 0.211588243 0.179476386 STC2/H6PD 2

BP GO:0046931 pore complex assembly 2/262 20/18862 0.03097872 0.211588243 0.179476386 BAD/CCT8 2

BP GO:0048025 "negative regulation of mRNA splicing, via spliceosome" 2/262 20/18862 0.03097872 0.211588243 0.179476386 DYRK1A/SRSF10 2

BP GO:0061318 renal filtration cell differentiation 2/262 20/18862 0.03097872 0.211588243 0.179476386 LAMB2/JAG1 2

BP GO:0072112 glomerular visceral epithelial cell differentiation 2/262 20/18862 0.03097872 0.211588243 0.179476386 LAMB2/JAG1 2

BP GO:0090026 positive regulation of monocyte chemotaxis 2/262 20/18862 0.03097872 0.211588243 0.179476386 CXCL12/HMGB1 2

BP GO:0097062 dendritic spine maintenance 2/262 20/18862 0.03097872 0.211588243 0.179476386 IGF1R/FYN 2

BP GO:2001235 positive regulation of apoptotic signaling pathway 5/262 126/18862 0.031345463 0.213680635 0.181251225 SIAH1/BCL2L1/TIMP3/BAD/BCLAF1 5

BP GO:0060021 roof of mouth development 4/262 86/18862 0.031865479 0.216807818 0.183903808 INSIG1/PRRX1/SMAD2/SNAI1 4

BP GO:0030111 regulation of Wnt signaling pathway 10/262 365/18862 0.031953778 0.2169913 0.184059444 SULF1/CSNK1A1/IGFBP6/BICC1/CDC73/TLE2/GREM1/BARX1/RBPJ/PTPRU 10

BP GO:0032456 endocytic recycling 3/262 50/18862 0.032170169 0.217210035 0.184244982 ARL4C/EPS15/EHD1 3

BP GO:0032648 regulation of interferon-beta production 3/262 50/18862 0.032170169 0.217210035 0.184244982 HSP90AA1/HMGB1/PTPN11 3

BP GO:1990089 response to nerve growth factor 3/262 50/18862 0.032170169 0.217210035 0.184244982 ID1/CDC5L/EHD1 3

BP GO:1904950 negative regulation of establishment of protein localization 5/262 127/18862 0.032274612 0.217500147 0.184491065 INSIG1/LYPLA1/ANGPT1/CHP1/PTPN11 5

BP GO:0051302 regulation of cell division 6/262 171/18862 0.032443704 0.218224 0.185105062 PDGFC/TXNIP/BCL2L1/PIK3R4/PIK3C3/SFN 6

BP GO:0030099 myeloid cell differentiation 11/262 419/18862 0.032897353 0.220855476 0.18733717 SP3/CDC73/IFI16/JAG1/NFKBIA/FBN1/RARG/RBPJ/PRKDC/HMGB1/PTPN11 11

BP GO:0043297 apical junction assembly 4/262 87/18862 0.033049595 0.221457324 0.187847678 MTDH/MYO1C/GJA1/SNAI1 4

BP GO:0006022 aminoglycan metabolic process 6/262 172/18862 0.033241544 0.222322464 0.188581519 CSPG4/CEMIP/SDC3/CSGALNACT1/ANGPT1/CHP1 6

BP GO:0002831 regulation of response to biotic stimulus 11/262 420/18862 0.033383521 0.22285075 0.18902963 USP15/FYN/IFI16/PUM2/PCBP2/MATR3/HSP90AA1/IFNGR1/PRKDC/HMGB1/PTPN11 11

BP GO:0010718 positive regulation of epithelial to mesenchymal transition 3/262 51/18862 0.033840184 0.223555556 0.18962747 SMAD2/JAG1/SNAI1 3

BP GO:0032206 positive regulation of telomere maintenance 3/262 51/18862 0.033840184 0.223555556 0.18962747 CCT6A/HNRNPA1/CCT8 3

BP GO:0000002 mitochondrial genome maintenance 2/262 21/18862 0.033931411 0.223555556 0.18962747 MEF2A/SLC25A36 2

BP GO:0006662 glycerol ether metabolic process 2/262 21/18862 0.033931411 0.223555556 0.18962747 PLA2G6/PLA2G4A 2

BP GO:0009070 serine family amino acid biosynthetic process 2/262 21/18862 0.033931411 0.223555556 0.18962747 CTH/PSPH 2

BP GO:0036344 platelet morphogenesis 2/262 21/18862 0.033931411 0.223555556 0.18962747 PRKDC/PTPN11 2

BP GO:0072311 glomerular epithelial cell differentiation 2/262 21/18862 0.033931411 0.223555556 0.18962747 LAMB2/JAG1 2

BP GO:0048880 sensory system development 10/262 369/18862 0.034038729 0.223845767 0.189873637 SP3/LAMB2/DLX2/COL8A1/JAG1/EFEMP1/CYP1B1/FBN1/RARG/HMGB1 10

BP GO:0019079 viral genome replication 5/262 129/18862 0.034184145 0.224384979 0.190331015 PPID/IFI16/TOP2A/PCBP2/ISG20 5

BP GO:0048638 regulation of developmental growth 9/262 319/18862 0.034749417 0.227234587 0.19274815 FN1/GJA1/FGFR3/PLXNA3/CDKN1B/RBPJ/PRKDC/GDF15/CXCL12 9

BP GO:0008654 phospholipid biosynthetic process 8/262 269/18862 0.03477024 0.227234587 0.19274815 LPCAT3/PIK3R4/PIK3C3/PLA2G6/PLA2G4A/PI4KB/CHP1/MTMR6 8

BP GO:0010721 negative regulation of cell development 6/262 174/18862 0.034875178 0.227234587 0.19274815 POSTN/DLX2/PLXNA3/ID1/FBN1/DICER1 6

BP GO:2000045 regulation of G1/S transition of mitotic cell cycle 6/262 174/18862 0.034875178 0.227234587 0.19274815 CDC73/CCND2/PKD2/CDKN1B/SFN/PRKDC 6

BP GO:0043624 cellular protein complex disassembly 7/262 221/18862 0.035132274 0.228488945 0.193812139 MRPS10/EPS8/CAPZA1/KIF2A/VPS4B/TWF1/MRPL19 7

BP GO:0097581 lamellipodium organization 4/262 89/18862 0.035494182 0.229128555 0.194354678 ENPP2/TWF1/ACTR2/CDH13 4

BP GO:0032210 regulation of telomere maintenance via telomerase 3/262 52/18862 0.035554431 0.229128555 0.194354678 CCT6A/HNRNPA1/CCT8 3

BP GO:0032608 interferon-beta production 3/262 52/18862 0.035554431 0.229128555 0.194354678 HSP90AA1/HMGB1/PTPN11 3

BP GO:0035196 production of miRNAs involved in gene silencing by miRNA 3/262 52/18862 0.035554431 0.229128555 0.194354678 PUM2/SMAD2/DICER1 3

BP GO:0043392 negative regulation of DNA binding 3/262 52/18862 0.035554431 0.229128555 0.194354678 IFI16/ID1/NFKBIA 3

BP GO:0048771 tissue remodeling 6/262 175/18862 0.035711084 0.229680031 0.194822459 CSPG4/GJA1/GREM1/JAG1/MMP2/RBPJ 6

BP GO:0045930 negative regulation of mitotic cell cycle 9/262 321/18862 0.035937324 0.229680031 0.194822459 CDC73/HECA/FAP/BCL2L1/PKD2/CDKN1B/SFN/PRKDC/ATF2 9

BP GO:1903532 positive regulation of secretion by cell 8/262 271/18862 0.036078574 0.229680031 0.194822459 GJA1/PPID/VPS4B/BAD/PLA2G6/PLA2G4A/CXCL12/PTPN11 8

BP GO:0072073 kidney epithelium development 5/262 131/18862 0.036162424 0.229680031 0.194822459 LAMB2/GREM1/PKD2/SMAD2/JAG1 5

BP GO:0072503 cellular divalent inorganic cation homeostasis 12/262 480/18862 0.036597936 0.229680031 0.194822459 BDKRB1/GJA1/CEMIP/FYN/CCR10/STC2/PKD2/EDNRA/SLC30A9/HSP90B1/CXCL12/HMGB1 12

BP GO:0046470 phosphatidylcholine metabolic process 4/262 90/18862 0.036754733 0.229680031 0.194822459 LPCAT3/ENPP2/PLA2G6/PLA2G4A 4

BP GO:0010888 negative regulation of lipid storage 2/262 22/18862 0.036988954 0.229680031 0.194822459 OSBPL8/NFKBIA 2

BP GO:0034643 "establishment of mitochondrion localization, microtubule-mediated" 2/262 22/18862 0.036988954 0.229680031 0.194822459 RHOT1/LRPPRC 2

BP GO:0043371 "negative regulation of CD4-positive, alpha-beta T cell differentiation" 2/262 22/18862 0.036988954 0.229680031 0.194822459 RUNX3/HMGB1 2

BP GO:0047497 mitochondrion transport along microtubule 2/262 22/18862 0.036988954 0.229680031 0.194822459 RHOT1/LRPPRC 2

BP GO:0050765 negative regulation of phagocytosis 2/262 22/18862 0.036988954 0.229680031 0.194822459 ATG3/HMGB1 2

BP GO:0051131 chaperone-mediated protein complex assembly 2/262 22/18862 0.036988954 0.229680031 0.194822459 HSPA4/HSP90AA1 2

BP GO:0051220 cytoplasmic sequestering of protein 2/262 22/18862 0.036988954 0.229680031 0.194822459 PKD2/NFKBIA 2

BP GO:0060343 trabecula formation 2/262 22/18862 0.036988954 0.229680031 0.194822459 GREM1/MMP2 2

BP GO:0072243 metanephric nephron epithelium development 2/262 22/18862 0.036988954 0.229680031 0.194822459 LAMB2/PKD2 2

BP GO:0000731 DNA synthesis involved in DNA repair 3/262 53/18862 0.037312699 0.229680031 0.194822459 RFC4/POLA1/POLD3 3

BP GO:0003179 heart valve morphogenesis 3/262 53/18862 0.037312699 0.229680031 0.194822459 JAG1/SNAI1/ROBO1 3

BP GO:0006984 ER-nucleus signaling pathway 3/262 53/18862 0.037312699 0.229680031 0.194822459 LPCAT3/INSIG1/HSP90B1 3

BP GO:0010524 positive regulation of calcium ion transport into cytosol 3/262 53/18862 0.037312699 0.229680031 0.194822459 BDKRB1/CEMIP/PKD2 3

BP GO:0048016 inositol phosphate-mediated signaling 3/262 53/18862 0.037312699 0.229680031 0.194822459 HOMER3/DYRK2/CHP1 3

BP GO:0051653 spindle localization 3/262 53/18862 0.037312699 0.229680031 0.194822459 GJA1/GPSM2/ACTR2 3

BP GO:1902743 regulation of lamellipodium organization 3/262 53/18862 0.037312699 0.229680031 0.194822459 ENPP2/TWF1/ACTR2 3

BP GO:0000302 response to reactive oxygen species 7/262 224/18862 0.037360594 0.229680031 0.194822459 TXNIP/ZNF277/FYN/PKD2/BAD/CYP1B1/MMP2 7

BP GO:0050920 regulation of chemotaxis 7/262 224/18862 0.037360594 0.229680031 0.194822459 GREM1/FGF18/PLXNA3/CDH13/CXCL12/HMGB1/ROBO1 7

BP GO:0001936 regulation of endothelial cell proliferation 6/262 177/18862 0.037421339 0.229680031 0.194822459 SULF1/GJA1/CDH13/CXCL12/HMGB1/FGFBP1 6

BP GO:0002478 antigen processing and presentation of exogenous peptide antigen 6/262 177/18862 0.037421339 0.229680031 0.194822459 CAPZA1/AP1S2/KIF2A/SEC24D/CANX/HLA-DMA 6

BP GO:0043433 negative regulation of DNA-binding transcription factor activity 6/262 177/18862 0.037421339 0.229680031 0.194822459 PKD2/ID1/CYP1B1/NFKBIA/CHP1/TRIM37 6

BP GO:0010975 regulation of neuron projection development 11/262 428/18862 0.03745771 0.229680031 0.194822459 FN1/FYN/PRRX1/PTPRG/VLDLR/PLXNA3/ID1/TWF1/ACTR2/CXCL12/ROBO1 11

BP GO:0048524 positive regulation of viral process 4/262 91/18862 0.03804083 0.231754706 0.19658227 PPID/VPS4B/TOP2A/POLR2B 4

BP GO:0050810 regulation of steroid biosynthetic process 4/262 91/18862 0.03804083 0.231754706 0.19658227 LPCAT3/INSIG1/SNAI1/H6PD 4

BP GO:1901992 positive regulation of mitotic cell cycle phase transition 4/262 91/18862 0.03804083 0.231754706 0.19658227 VPS4B/CCND2/CDC27/RAD51C 4

BP GO:0006839 mitochondrial transport 8/262 274/18862 0.038102828 0.231754706 0.19658227 BCL2L1/RHOT1/HSPA4/BAD/SLC25A36/SFN/HSP90AA1/ATF2 8

BP GO:0032271 regulation of protein polymerization 7/262 225/18862 0.038123584 0.231754706 0.19658227 EPS8/CAPZA1/MYO1C/DYRK1A/TWF1/ACTR2/HSP90AA1 7

BP GO:0045727 positive regulation of translation 5/262 133/18862 0.038209918 0.231881114 0.196689494 RMND1/UPF3A/PAIP1/PRKDC/NPM1 5

BP GO:0032869 cellular response to insulin stimulus 7/262 226/18862 0.038896752 0.235052806 0.199379831 ECHDC3/MYO1C/IGF1R/INSIG1/OSBPL8/PRKDC/PTPN11 7

BP GO:0060284 regulation of cell development 12/262 485/18862 0.039101503 0.235052806 0.199379831 POSTN/FN1/DLX2/PLXNA3/ID1/BAD/ACTR2/FBN1/PRKDC/CXCL12/DICER1/ROBO1 12

BP GO:0030199 collagen fibril organization 3/262 54/18862 0.039114759 0.235052806 0.199379831 COL3A1/GREM1/CYP1B1 3

BP GO:0030834 regulation of actin filament depolymerization 3/262 54/18862 0.039114759 0.235052806 0.199379831 EPS8/CAPZA1/TWF1 3

BP GO:0090090 negative regulation of canonical Wnt signaling pathway 6/262 179/18862 0.039183183 0.235052806 0.199379831 CSNK1A1/IGFBP6/BICC1/TLE2/GREM1/PTPRU 6

BP GO:0032543 mitochondrial translation 5/262 134/18862 0.039259751 0.235052806 0.199379831 MRPS10/RMND1/MTIF2/MRPL19/LRPPRC 5

BP GO:0071482 cellular response to light stimulus 5/262 134/18862 0.039259751 0.235052806 0.199379831 CUL4B/PPID/MMP2/POLD3/NPM1 5

BP GO:0032088 negative regulation of NF-kappaB transcription factor activity 4/262 92/18862 0.039352491 0.235052806 0.199379831 CYP1B1/NFKBIA/CHP1/TRIM37 4

BP GO:0048285 organelle fission 12/262 486/18862 0.039616353 0.235052806 0.199379831 EPS8/PRKAR1A/KIF2A/VPS4B/TOP2A/NSL1/PDS5B/STAG2/CDC27/RAD51C/ACTR2/PDS5A 12

BP GO:0046474 glycerophospholipid biosynthetic process 7/262 227/18862 0.039680139 0.235052806 0.199379831 LPCAT3/PIK3R4/PIK3C3/PLA2G6/PLA2G4A/PI4KB/MTMR6 7

BP GO:0003416 endochondral bone growth 2/262 23/18862 0.04014789 0.235052806 0.199379831 FGFR3/RARG 2

BP GO:0005980 glycogen catabolic process 2/262 23/18862 0.04014789 0.235052806 0.199379831 PHKB/HMGB1 2

BP GO:0006297 "nucleotide-excision repair, DNA gap filling" 2/262 23/18862 0.04014789 0.235052806 0.199379831 RFC4/POLD3 2

BP GO:0034063 stress granule assembly 2/262 23/18862 0.04014789 0.235052806 0.199379831 PUM2/CSDE1 2

BP GO:0039531 regulation of viral-induced cytoplasmic pattern recognition receptor signaling pathway 2/262 23/18862 0.04014789 0.235052806 0.199379831 USP15/PUM2 2

BP GO:0042026 protein refolding 2/262 23/18862 0.04014789 0.235052806 0.199379831 HSPA13/HSP90AA1 2

BP GO:0046628 positive regulation of insulin receptor signaling pathway 2/262 23/18862 0.04014789 0.235052806 0.199379831 OSBPL8/PTPN11 2

BP GO:0060143 positive regulation of syncytium formation by plasma membrane fusion 2/262 23/18862 0.04014789 0.235052806 0.199379831 EHD1/GDF15 2

BP GO:0060706 cell differentiation involved in embryonic placenta development 2/262 23/18862 0.04014789 0.235052806 0.199379831 SNAI1/KRT19 2

BP GO:0070977 bone maturation 2/262 23/18862 0.04014789 0.235052806 0.199379831 FGFR3/GREM1 2

BP GO:0046486 glycerolipid metabolic process 11/262 433/18862 0.040174433 0.235052806 0.199379831 LPCAT3/INSIG1/OSBPL8/PIK3R4/ENPP2/PIK3C3/PLA2G6/PLA2G4A/PI4KB/PTPN11/MTMR6 11

BP GO:0007411 axon guidance 8/262 277/18862 0.04020204 0.235052806 0.199379831 SIAH1/FYN/LAMB2/VLDLR/PLXNA3/CXCL12/ROBO1/PTPN11 8

BP GO:0006898 receptor-mediated endocytosis 9/262 328/18862 0.040314212 0.235052806 0.199379831 EPS15/GREM1/VLDLR/CANX/ANGPT1/TNK2/HSP90AA1/HSP90B1/PI4KB 9

BP GO:0042542 response to hydrogen peroxide 5/262 135/18862 0.040327036 0.235052806 0.199379831 TXNIP/ZNF277/FYN/BAD/CYP1B1 5

BP GO:0097485 neuron projection guidance 8/262 278/18862 0.040918593 0.237570077 0.201515066 SIAH1/FYN/LAMB2/VLDLR/PLXNA3/CXCL12/ROBO1/PTPN11 8

BP GO:0001836 release of cytochrome c from mitochondria 3/262 55/18862 0.040960358 0.237570077 0.201515066 BCL2L1/BAD/SFN 3

BP GO:0097345 mitochondrial outer membrane permeabilization 3/262 55/18862 0.040960358 0.237570077 0.201515066 RHOT1/BAD/SFN 3

BP GO:0043406 positive regulation of MAP kinase activity 7/262 230/18862 0.042091995 0.243734008 0.206743523 PDGFC/CSPG4/IGFBP6/FGF18/GDF15/ROBO1/PTPN11 7

BP GO:0010883 regulation of lipid storage 3/262 56/18862 0.042849227 0.24669991 0.209259303 OSBPL8/EHD1/NFKBIA 3

BP GO:0021872 forebrain generation of neurons 3/262 56/18862 0.042849227 0.24669991 0.209259303 DLX2/PLXNA3/ROBO1 3

BP GO:0042306 regulation of protein import into nucleus 3/262 56/18862 0.042849227 0.24669991 0.209259303 IPO5/ANGPT1/CHP1 3

BP GO:0048762 mesenchymal cell differentiation 7/262 231/18862 0.042916636 0.24669991 0.209259303 FN1/GREM1/EDNRA/SMAD2/JAG1/RBPJ/SNAI1 7

BP GO:0003094 glomerular filtration 2/262 24/18862 0.043404834 0.24669991 0.209259303 SULF1/GJA1 2

BP GO:0006309 apoptotic DNA fragmentation 2/262 24/18862 0.043404834 0.24669991 0.209259303 HMGB1/DICER1 2

BP GO:0009251 glucan catabolic process 2/262 24/18862 0.043404834 0.24669991 0.209259303 PHKB/HMGB1 2

BP GO:0044068 modulation by symbiont of host cellular process 2/262 24/18862 0.043404834 0.24669991 0.209259303 BCL2L1/BAD 2

BP GO:0062149 detection of stimulus involved in sensory perception of pain 2/262 24/18862 0.043404834 0.24669991 0.209259303 FYN/CXCL12 2

BP GO:0072010 glomerular epithelium development 2/262 24/18862 0.043404834 0.24669991 0.209259303 LAMB2/JAG1 2

BP GO:0006903 vesicle targeting 4/262 95/18862 0.043440939 0.24669991 0.209259303 WDR11/SEC24D/CNIH1/CHP1 4

BP GO:0010522 regulation of calcium ion transport into cytosol 4/262 95/18862 0.043440939 0.24669991 0.209259303 BDKRB1/CEMIP/FYN/PKD2 4

BP GO:0002040 sprouting angiogenesis 6/262 184/18862 0.04381587 0.248012628 0.210372795 GREM1/ANGPT1/CDH13/HMGB1/FGFBP1/ROBO1 6

BP GO:0034248 regulation of cellular amide metabolic process 12/262 494/18862 0.043907912 0.248012628 0.210372795 RMND1/NSMAF/MTIF2/PUM2/UPF3A/CSDE1/PAIP1/SRP9/IFNGR1/PRKDC/LRPPRC/NPM1 12

BP GO:0002433 immune response-regulating cell surface receptor signaling pathway involved in phagocytosis 5/262 139/18862 0.044771491 0.248012628 0.210372795 MYO1C/FYN/ACTR2/PLA2G6/HSP90AA1 5

BP GO:0031333 negative regulation of protein-containing complex assembly 5/262 139/18862 0.044771491 0.248012628 0.210372795 EPS8/CAPZA1/DYRK1A/TWF1/HMGB1 5

BP GO:0038096 Fc-gamma receptor signaling pathway involved in phagocytosis 5/262 139/18862 0.044771491 0.248012628 0.210372795 MYO1C/FYN/ACTR2/PLA2G6/HSP90AA1 5

BP GO:0048813 dendrite morphogenesis 5/262 139/18862 0.044771491 0.248012628 0.210372795 FYN/MEF2A/VLDLR/ID1/ACTR2 5

BP GO:0000381 "regulation of alternative mRNA splicing, via spliceosome" 3/262 57/18862 0.044781076 0.248012628 0.210372795 DYRK1A/HNRNPA1/RBM7 3

BP GO:0032387 negative regulation of intracellular transport 3/262 57/18862 0.044781076 0.248012628 0.210372795 INSIG1/ANGPT1/CHP1 3

BP GO:0072431 signal transduction involved in mitotic G1 DNA damage checkpoint 3/262 57/18862 0.044781076 0.248012628 0.210372795 CDKN1B/SFN/PRKDC 3

BP GO:1902400 intracellular signal transduction involved in G1 DNA damage checkpoint 3/262 57/18862 0.044781076 0.248012628 0.210372795 CDKN1B/SFN/PRKDC 3

BP GO:0008064 regulation of actin polymerization or depolymerization 6/262 185/18862 0.044781828 0.248012628 0.210372795 EPS8/CAPZA1/MYO1C/TWF1/ACTR2/CXCL12 6

BP GO:0009749 response to glucose 6/262 185/18862 0.044781828 0.248012628 0.210372795 GJA1/TXNIP/IGF1R/SMAD2/BAD/PLA2G6 6

BP GO:0019884 antigen processing and presentation of exogenous antigen 6/262 185/18862 0.044781828 0.248012628 0.210372795 CAPZA1/AP1S2/KIF2A/SEC24D/CANX/HLA-DMA 6

BP GO:0000045 autophagosome assembly 4/262 96/18862 0.044854897 0.248012628 0.210372795 ATG12/TRAPPC8/PIK3C3/ATG3 4

BP GO:0003279 cardiac septum development 4/262 96/18862 0.044854897 0.248012628 0.210372795 MATR3/JAG1/RBPJ/ROBO1 4

BP GO:0048010 vascular endothelial growth factor receptor signaling pathway 4/262 96/18862 0.044854897 0.248012628 0.210372795 SULF1/FYN/FGF18/HSP90AA1 4

BP GO:0045765 regulation of angiogenesis 9/262 335/18862 0.045039665 0.248012628 0.210372795 SULF1/HTATIP2/MTDH/GREM1/FGF18/ENPP2/CYP1B1/HMGB1/ATF2 9

BP GO:0006874 cellular calcium ion homeostasis 11/262 442/18862 0.045404736 0.248012628 0.210372795 BDKRB1/GJA1/CEMIP/FYN/CCR10/STC2/PKD2/EDNRA/HSP90B1/CXCL12/HMGB1 11

BP GO:0001763 morphogenesis of a branching structure 6/262 186/18862 0.045761011 0.248012628 0.210372795 SULF1/DLX2/GREM1/PKD2/DDR1/EDNRA 6

BP GO:0030832 regulation of actin filament length 6/262 186/18862 0.045761011 0.248012628 0.210372795 EPS8/CAPZA1/MYO1C/TWF1/ACTR2/CXCL12 6

BP GO:0050792 regulation of viral process 6/262 186/18862 0.045761011 0.248012628 0.210372795 PPID/IFI16/VPS4B/TOP2A/ISG20/POLR2B 6

BP GO:0038094 Fc-gamma receptor signaling pathway 5/262 140/18862 0.045926599 0.248012628 0.210372795 MYO1C/FYN/ACTR2/PLA2G6/HSP90AA1 5

BP GO:0051090 regulation of DNA-binding transcription factor activity 11/262 444/18862 0.046627604 0.248012628 0.210372795 MTDH/GREM1/CTH/PKD2/ID1/CYP1B1/NFKBIA/CHP1/ATF2/TRIM37/NPM1 11

BP GO:0072507 divalent inorganic cation homeostasis 12/262 499/18862 0.046748795 0.248012628 0.210372795 BDKRB1/GJA1/CEMIP/FYN/CCR10/STC2/PKD2/EDNRA/SLC30A9/HSP90B1/CXCL12/HMGB1 12

BP GO:0060038 cardiac muscle cell proliferation 3/262 58/18862 0.0467556 0.248012628 0.210372795 GJA1/PRKAR1A/RBPJ 3

BP GO:0061005 cell differentiation involved in kidney development 3/262 58/18862 0.0467556 0.248012628 0.210372795 LAMB2/GREM1/JAG1 3

BP GO:0002068 glandular epithelial cell development 2/262 25/18862 0.046756479 0.248012628 0.210372795 BAD/RARG 2

BP GO:0002092 positive regulation of receptor internalization 2/262 25/18862 0.046756479 0.248012628 0.210372795 GREM1/ANGPT1 2

BP GO:0021884 forebrain neuron development 2/262 25/18862 0.046756479 0.248012628 0.210372795 PLXNA3/ROBO1 2

BP GO:0030206 chondroitin sulfate biosynthetic process 2/262 25/18862 0.046756479 0.248012628 0.210372795 CSPG4/CSGALNACT1 2

BP GO:0033119 negative regulation of RNA splicing 2/262 25/18862 0.046756479 0.248012628 0.210372795 DYRK1A/SRSF10 2

BP GO:0033622 integrin activation 2/262 25/18862 0.046756479 0.248012628 0.210372795 FN1/CXCL12 2

BP GO:0039529 RIG-I signaling pathway 2/262 25/18862 0.046756479 0.248012628 0.210372795 USP15/PUM2 2

BP GO:0044247 cellular polysaccharide catabolic process 2/262 25/18862 0.046756479 0.248012628 0.210372795 PHKB/HMGB1 2

BP GO:0050927 positive regulation of positive chemotaxis 2/262 25/18862 0.046756479 0.248012628 0.210372795 CDH13/CXCL12 2

BP GO:0051123 RNA polymerase II preinitiation complex assembly 2/262 25/18862 0.046756479 0.248012628 0.210372795 CAND1/HMGB1 2

BP GO:0051654 establishment of mitochondrion localization 2/262 25/18862 0.046756479 0.248012628 0.210372795 RHOT1/LRPPRC 2

BP GO:0060740 prostate gland epithelium morphogenesis 2/262 25/18862 0.046756479 0.248012628 0.210372795 SULF1/RARG 2

BP GO:0070129 regulation of mitochondrial translation 2/262 25/18862 0.046756479 0.248012628 0.210372795 RMND1/LRPPRC 2

BP GO:0072202 cell differentiation involved in metanephros development 2/262 25/18862 0.046756479 0.248012628 0.210372795 LAMB2/GREM1 2

BP GO:0072207 metanephric epithelium development 2/262 25/18862 0.046756479 0.248012628 0.210372795 LAMB2/PKD2 2

BP GO:0072273 metanephric nephron morphogenesis 2/262 25/18862 0.046756479 0.248012628 0.210372795 GREM1/PKD2 2

BP GO:0090169 regulation of spindle assembly 2/262 25/18862 0.046756479 0.248012628 0.210372795 GPSM2/VPS4B 2

BP GO:0097205 renal filtration 2/262 25/18862 0.046756479 0.248012628 0.210372795 SULF1/GJA1 2

BP GO:0043488 regulation of mRNA stability 6/262 188/18862 0.047759176 0.252573936 0.214241853 SERBP1/TNPO1/PUM2/PAIP1/NPM1/CSDC2 6

BP GO:0060541 respiratory system development 6/262 188/18862 0.047759176 0.252573936 0.214241853 SP3/FGF18/SMAD2/RARG/RBPJ/HMGB1 6

BP GO:0043254 regulation of protein-containing complex assembly 11/262 446/18862 0.047872828 0.252797115 0.214431161 CUL4B/EPS8/CAPZA1/MYO1C/DYRK1A/CAND1/TWF1/ACTR2/HSP90AA1/HMGB1/PTPN11 11

BP GO:1903320 regulation of protein modification by small protein conjugation or removal 7/262 237/18862 0.048084063 0.253361109 0.21490956 FYN/TRIP12/ANGPT1/HSP90AA1/DCUN1D4/FBXO2/CHP1 7

BP GO:0009314 response to radiation 11/262 447/18862 0.048503869 0.253361109 0.21490956 CUL4B/PENK/COL3A1/PPID/MSH6/IFI16/MMP2/PRKDC/CXCL12/POLD3/NPM1 11

BP GO:0019369 arachidonic acid metabolic process 3/262 59/18862 0.048772475 0.253361109 0.21490956 EPHX1/PLA2G4A/CYP1B1 3

BP GO:0030042 actin filament depolymerization 3/262 59/18862 0.048772475 0.253361109 0.21490956 EPS8/CAPZA1/TWF1 3

BP GO:0030239 myofibril assembly 3/262 59/18862 0.048772475 0.253361109 0.21490956 PRKAR1A/MEF2A/KRT19 3

BP GO:0046324 regulation of glucose import 3/262 59/18862 0.048772475 0.253361109 0.21490956 MEF2A/OSBPL8/PTPN11 3

BP GO:0048663 neuron fate commitment 3/262 59/18862 0.048772475 0.253361109 0.21490956 DLX2/PRRX1/RBPJ 3

BP GO:1902402 signal transduction involved in mitotic DNA damage checkpoint 3/262 59/18862 0.048772475 0.253361109 0.21490956 CDKN1B/SFN/PRKDC 3

BP GO:1902403 signal transduction involved in mitotic DNA integrity checkpoint 3/262 59/18862 0.048772475 0.253361109 0.21490956 CDKN1B/SFN/PRKDC 3

BP GO:1904589 regulation of protein import 3/262 59/18862 0.048772475 0.253361109 0.21490956 IPO5/ANGPT1/CHP1 3

BP GO:1905952 regulation of lipid localization 6/262 189/18862 0.048778216 0.253361109 0.21490956 LPCAT3/OSBPL8/EHD1/PLA2G4A/NFKBIA/PTPN11 6

BP GO:0051924 regulation of calcium ion transport 7/262 238/18862 0.048982184 0.253361109 0.21490956 BDKRB1/GJA1/CEMIP/FYN/HOMER3/PKD2/CXCL12 7

BP GO:0019233 sensory perception of pain 4/262 99/18862 0.049249926 0.253361109 0.21490956 BDKRB1/PENK/FYN/CXCL12 4

BP GO:1905037 autophagosome organization 4/262 99/18862 0.049249926 0.253361109 0.21490956 ATG12/TRAPPC8/PIK3C3/ATG3 4

BP GO:0032102 negative regulation of response to external stimulus 10/262 394/18862 0.049254011 0.253361109 0.21490956 GJA1/TNFAIP6/LPCAT3/IFI16/GREM1/FAP/PLXNA3/PCBP2/TFPI/ROBO1 10

BP GO:1902850 microtubule cytoskeleton organization involved in mitosis 5/262 143/18862 0.049497907 0.253361109 0.21490956 GJA1/GPSM2/KIF2A/VPS4B/STAG2 5

BP GO:0009746 response to hexose 6/262 190/18862 0.049810597 0.253361109 0.21490956 GJA1/TXNIP/IGF1R/SMAD2/BAD/PLA2G6 6

CC GO:0062023 collagen-containing extracellular matrix 23/268 423/19520 2.31E-08 9.21E-06 8.24E-06 SULF1/POSTN/COL3A1/CSPG4/FN1/LRRC15/FBLN2/LTBP1/SDC3/LAMB2/GREM1/COL8A1/TIMP3/ANGPT1/CDH13/EFEMP1/F13A1/COL15A1/HSP90B1/MMP2/FBN1/GDF15/CXCL12 23

CC GO:0005635 nuclear envelope 19/268 462/19520 2.30E-05 0.003098387 0.002771015 HTATIP2/DPY19L4/MTDH/MYO1C/IPO5/CCND2/BCL2L1/POLA1/OSBPL8/PUM2/GCHFR/MATR3/LYPLA1/XPOT/PLA2G4A/UBXN4/MRPL19/LRPPRC/MTMR6 19

CC GO:0005788 endoplasmic reticulum lumen 15/268 306/19520 2.33E-05 0.003098387 0.002771015 PDGFC/PENK/COL3A1/FN1/GPX7/LTBP1/LAMB2/STC2/DNAJC10/COL8A1/CANX/COL15A1/HSP90B1/FBN1/H6PD 15

CC GO:0061695 "transferase complex, transferring phosphorus-containing groups" 13/268 253/19520 5.06E-05 0.00504278 0.004509965 IGF1R/PHKB/CDC73/PRKAR1A/CCND2/POLA1/PIK3R4/PIK3C3/TCEA1/POLD3/PRIM1/CCNG2/POLR2B 13

CC GO:0043601 nuclear replisome 4/268 22/19520 0.000209145 0.016689792 0.014926367 POLA1/CDC5L/POLD3/PRIM1 4

CC GO:0005657 replication fork 6/268 65/19520 0.000265449 0.016947351 0.015156712 RFC4/POLA1/CDC5L/RAD51C/POLD3/PRIM1 6

CC GO:0030894 replisome 4/268 24/19520 0.000297322 0.016947351 0.015156712 POLA1/CDC5L/POLD3/PRIM1 4

CC GO:0034774 secretory granule lumen 13/268 322/19520 0.000539714 0.024358378 0.021784699 PCYOX1L/PENK/FN1/QPCT/CAND1/TIMP3/ACTR2/F13A1/HSP90AA1/GDI2/HMGB1/OLA1/CCT8 13

CC GO:0060205 cytoplasmic vesicle lumen 13/268 326/19520 0.000605629 0.024358378 0.021784699 PCYOX1L/PENK/FN1/QPCT/CAND1/TIMP3/ACTR2/F13A1/HSP90AA1/GDI2/HMGB1/OLA1/CCT8 13

CC GO:0031983 vesicle lumen 13/268 328/19520 0.000641059 0.024358378 0.021784699 PCYOX1L/PENK/FN1/QPCT/CAND1/TIMP3/ACTR2/F13A1/HSP90AA1/GDI2/HMGB1/OLA1/CCT8 13

CC GO:0005925 focal adhesion 15/268 416/19520 0.000671534 0.024358378 0.021784699 CSPG4/GJA1/DCAF6/FGFR3/TLE2/FAP/TNS1/PCBP2/TWF1/ACTR2/CDH13/HSP90B1/GDI2/CHP1/NPM1 15

CC GO:0030055 cell-substrate junction 15/268 423/19520 0.000795647 0.026455278 0.023660043 CSPG4/GJA1/DCAF6/FGFR3/TLE2/FAP/TNS1/PCBP2/TWF1/ACTR2/CDH13/HSP90B1/GDI2/CHP1/NPM1 15

CC GO:0098687 chromosomal region 13/268 345/19520 0.001019274 0.031283858 0.027978441 CSNK1A1/CBX1/CDC73/MCM6/TOP2A/NSL1/PDS5B/STAG2/PPP2R5C/PRKDC/SNAI1/POLR2B/PDS5A 13

CC GO:0043596 nuclear replication fork 4/268 35/19520 0.001301472 0.037091961 0.033172866 POLA1/CDC5L/POLD3/PRIM1 4

CC GO:0000775 "chromosome, centromeric region" 9/268 196/19520 0.001594657 0.040796507 0.036485994 CSNK1A1/CBX1/TOP2A/NSL1/PDS5B/STAG2/PPP2R5C/SNAI1/PDS5A 9

CC GO:1904813 ficolin-1-rich granule lumen 7/268 124/19520 0.00163595 0.040796507 0.036485994 TNFAIP6/QPCT/CAND1/ACTR2/HSP90AA1/HMGB1/CCT8 7

CC GO:0005793 endoplasmic reticulum-Golgi intermediate compartment 7/268 130/19520 0.002142331 0.050281767 0.044969052 FN1/CNIH1/HMGB1/CHP1/DICER1/ROBO1/MTMR6 7

CC GO:0042575 DNA polymerase complex 3/268 20/19520 0.002454071 0.054398583 0.04865089 POLA1/POLD3/PRIM1 3

CC GO:0031965 nuclear membrane 11/268 295/19520 0.002650659 0.055663836 0.049782457 DPY19L4/MTDH/CCND2/BCL2L1/OSBPL8/PUM2/GCHFR/MATR3/LYPLA1/MRPL19/LRPPRC 11

CC GO:0005905 clathrin-coated pit 5/268 71/19520 0.002925739 0.058368499 0.052201349 AP1S2/CEMIP/EPS15/VLDLR/TNK2 5

CC GO:0005884 actin filament 6/268 113/19520 0.004706733 0.089427929 0.079979074 PLS3/MYO1C/FYN/PKD2/TWF1/PDLIM4 6

CC GO:0000151 ubiquitin ligase complex 10/268 289/19520 0.006832033 0.123908233 0.110816227 CUL4B/FBXL5/DCAF6/DYRK2/CDKN1B/CAND1/CDC27/ATG3/DCUN1D4/FBXO2 10

CC GO:0031461 cullin-RING ubiquitin ligase complex 7/268 163/19520 0.007390063 0.128201535 0.114655904 CUL4B/FBXL5/DCAF6/CDKN1B/CAND1/CDC27/FBXO2 7

CC GO:0032993 protein-DNA complex 8/268 208/19520 0.008205788 0.129038209 0.115404176 SP3/POLA1/MCM6/CDC5L/PRKDC/POLD3/PRIM1/NPM1 8

CC GO:0031091 platelet alpha granule 5/268 91/19520 0.008382029 0.129038209 0.115404176 PCYOX1L/FN1/F13A1/PHACTR2/OLA1 5

CC GO:0000407 phagophore assembly site 3/268 31/19520 0.008660263 0.129038209 0.115404176 ATG12/TRAPPC8/PIK3C3 3

CC GO:0031941 filamentous actin 3/268 32/19520 0.009460658 0.129038209 0.115404176 MYO1C/PKD2/PDLIM4 3

CC GO:0005832 chaperonin-containing T-complex 2/268 11/19520 0.009518227 0.129038209 0.115404176 CCT6A/CCT8 2

CC GO:0005604 basement membrane 5/268 94/19520 0.009573029 0.129038209 0.115404176 FN1/LAMB2/COL8A1/COL15A1/FBN1 5

CC GO:0032587 ruffle membrane 5/268 95/19520 0.009994595 0.129038209 0.115404176 EPS8/MYO1C/FAP/TWF1/MTMR6 5

CC GO:0005775 vacuolar lumen 7/268 173/19520 0.01007722 0.129038209 0.115404176 CSPG4/SDC3/ACTR2/HSP90AA1/GDI2/PLD3/CCT8 7

CC GO:0031968 organelle outer membrane 8/268 220/19520 0.011271642 0.129038209 0.115404176 GJA1/BCL2L1/RHOT1/CHCHD3/BAD/PI4KB/LRPPRC/ATF2 8

CC GO:0005667 transcription regulator complex 12/268 409/19520 0.011280755 0.129038209 0.115404176 RUNX3/SP3/TLE2/MEF2A/SMAD2/RARG/RBPJ/PRKDC/SNAPC1/TCEA1/HMGB1/HOXA10 12

CC GO:0001527 microfibril 2/268 12/19520 0.011319141 0.129038209 0.115404176 LTBP1/FBN1 2

CC GO:0030877 beta-catenin destruction complex 2/268 12/19520 0.011319141 0.129038209 0.115404176 SIAH1/CSNK1A1 2

CC GO:0019867 outer membrane 8/268 222/19520 0.011855154 0.130633318 0.116830747 GJA1/BCL2L1/RHOT1/CHCHD3/BAD/PI4KB/LRPPRC/ATF2 8

CC GO:0080008 Cul4-RING E3 ubiquitin ligase complex 3/268 35/19520 0.012113867 0.130633318 0.116830747 CUL4B/DCAF6/CDKN1B 3

CC GO:0031093 platelet alpha granule lumen 4/268 67/19520 0.01354692 0.136555721 0.122127396 PCYOX1L/FN1/F13A1/OLA1 4

CC GO:0055029 nuclear DNA-directed RNA polymerase complex 5/268 103/19520 0.013833668 0.136555721 0.122127396 CDC73/POLA1/TCEA1/PRIM1/POLR2B 5

CC GO:0101002 ficolin-1-rich granule 7/268 185/19520 0.014168195 0.136555721 0.122127396 TNFAIP6/QPCT/CAND1/ACTR2/HSP90AA1/HMGB1/CCT8 7

CC GO:0000428 DNA-directed RNA polymerase complex 5/268 104/19520 0.014374286 0.136555721 0.122127396 CDC73/POLA1/TCEA1/PRIM1/POLR2B 5

CC GO:1902911 protein kinase complex 5/268 104/19520 0.014374286 0.136555721 0.122127396 IGF1R/PHKB/PRKAR1A/CCND2/CCNG2 5

CC GO:0031594 neuromuscular junction 4/268 69/19520 0.014959813 0.138813146 0.124146304 POSTN/PRKAR1A/LAMB2/SYNGR3 4

CC GO:0045239 tricarboxylic acid cycle enzyme complex 2/268 14/19520 0.015327691 0.138994285 0.124308304 DLD/SUCLG2 2

CC GO:0030880 RNA polymerase complex 5/268 108/19520 0.016678295 0.147880879 0.13225595 CDC73/POLA1/TCEA1/PRIM1/POLR2B 5

CC GO:0005741 mitochondrial outer membrane 7/268 195/19520 0.018389316 0.159507329 0.142653963 GJA1/BCL2L1/RHOT1/CHCHD3/BAD/PI4KB/ATF2 7

CC GO:0005786 "signal recognition particle, endoplasmic reticulum targeting" 2/268 16/19520 0.019851824 0.165018285 0.147582637 SRP72/SRP9 2

CC GO:0048500 signal recognition particle 2/268 16/19520 0.019851824 0.165018285 0.147582637 SRP72/SRP9 2

CC GO:0000228 nuclear chromosome 8/268 250/19520 0.022534745 0.183497205 0.164109095 SMARCE1/POLA1/MCM6/TOP2A/CDC5L/POLD3/PRIM1/LRPPRC 8

CC GO:0016607 nuclear speck 11/268 411/19520 0.027173131 0.215440681 0.192677459 MBD4/CSNK1A1/TRIP12/WAC/IFI16/DHX15/DYRK1A/NSL1/CDC5L/BCLAF1/SRSF10 11

CC GO:0000159 protein phosphatase type 2A complex 2/268 19/19520 0.027537531 0.215440681 0.192677459 PPP2R5E/PPP2R5C 2

CC GO:0005721 pericentric heterochromatin 2/268 20/19520 0.030324582 0.228292606 0.204171464 CBX1/SNAI1 2

CC GO:0071682 endocytic vesicle lumen 2/268 20/19520 0.030324582 0.228292606 0.204171464 HSP90AA1/HSP90B1 2

CC GO:0031256 leading edge membrane 6/268 173/19520 0.032468767 0.23990811 0.214559687 EPS8/CSPG4/MYO1C/FAP/TWF1/MTMR6 6

CC GO:0098644 complex of collagen trimers 2/268 21/19520 0.0332183 0.240983671 0.215521605 COL3A1/COL8A1 2

CC GO:0001726 ruffle 6/268 175/19520 0.034058843 0.242669259 0.217029096 EPS8/CSPG4/MYO1C/FAP/TWF1/MTMR6 6

CC GO:0031258 lamellipodium membrane 2/268 22/19520 0.036215256 0.253506793 0.226721548 CSPG4/FAP 2

CC GO:0043202 lysosomal lumen 4/268 96/19520 0.043308202 0.297930561 0.26645155 CSPG4/SDC3/HSP90AA1/PLD3 4

CC GO:0030867 rough endoplasmic reticulum membrane 2/268 26/19520 0.049169374 0.328622632 0.293900731 SRP9/PI4KB 2

CC GO:0005637 nuclear inner membrane 3/268 60/19520 0.049416937 0.328622632 0.293900731 DPY19L4/MATR3/LRPPRC 3

MF GO:0051082 unfolded protein binding 9/266 120/18337 6.39E-05 0.025717639 0.022687382 SCG5/HSPA13/CANX/CCT6A/HSP90AA1/HSP90B1/SRSF10/CCT8/NPM1 9

MF GO:0097718 disordered domain specific binding 5/266 34/18337 0.000121998 0.025717639 0.022687382 FN1/GJA1/FYN/SMAD2/HSP90AA1 5

MF GO:0140297 DNA-binding transcription factor binding 16/266 376/18337 0.000128803 0.025717639 0.022687382 MTDH/PPID/SMARCE1/TRIP12/TLE2/PRRX1/MEF2A/SLC30A9/SMAD2/UBA3/NFKBIA/RARG/RBPJ/HMGB1/ATF2/NPM1 16

MF GO:0005201 extracellular matrix structural constituent 10/266 170/18337 0.000193515 0.028978855 0.025564335 POSTN/COL3A1/FN1/FBLN2/LTBP1/LAMB2/COL8A1/EFEMP1/COL15A1/FBN1 10

MF GO:0003697 single-stranded DNA binding 8/266 116/18337 0.000290574 0.034810812 0.030709125 TSN/POLA1/MCM6/PCBP2/SSBP3/HMGB1/HNRNPA1/LRPPRC 8

MF GO:0004622 lysophospholipase activity 4/266 24/18337 0.000365959 0.036534938 0.032230101 ENPP2/LYPLA1/PLA2G6/PLA2G4A 4

MF GO:0061629 RNA polymerase II-specific DNA-binding transcription factor binding 12/266 271/18337 0.000628047 0.053742857 0.047410446 MTDH/PPID/SMARCE1/TRIP12/PRRX1/SLC30A9/UBA3/NFKBIA/RARG/RBPJ/ATF2/NPM1 12

MF GO:0004713 protein tyrosine kinase activity 8/266 135/18337 0.000800493 0.054387013 0.047978702 IGF1R/FGFR3/FYN/DYRK2/DYRK1A/DDR1/EFEMP1/TNK2 8

MF GO:0140097 "catalytic activity, acting on DNA" 10/266 204/18337 0.000817167 0.054387013 0.047978702 MBD4/RFC4/POLA1/MCM6/TOP2A/RAD51C/POLD3/ISG20/PLD3/DICER1 10

MF GO:0045296 cadherin binding 13/266 332/18337 0.001176982 0.070501215 0.062194201 BZW1/SERBP1/CAPZA1/EPS15/TWF1/CDH13/EHD1/SFN/CDH11/SLK/OLA1/CCT8/PTPN11 13

MF GO:0004536 deoxyribonuclease activity 5/266 59/18337 0.00163505 0.079929218 0.070511322 MBD4/RAD51C/ISG20/PLD3/DICER1 5

MF GO:0000400 four-way junction DNA binding 3/266 17/18337 0.001765445 0.079929218 0.070511322 MSH6/RAD51C/HMGB1 3

MF GO:0044183 protein folding chaperone 4/266 36/18337 0.001770048 0.079929218 0.070511322 HSPA13/CCT6A/HSP90AA1/CCT8 4

MF GO:0019887 protein kinase regulator activity 9/266 190/18337 0.001868129 0.079929218 0.070511322 PRKAR1A/GREM1/CCND2/CDKN1B/SFN/HMGB1/CHP1/CCNG2/NPM1 9

MF GO:0008301 "DNA binding, bending" 3/266 18/18337 0.002095962 0.083698763 0.073836709 TOP2A/HMGB1/TFAM 3

MF GO:0004860 protein kinase inhibitor activity 5/266 69/18337 0.00326488 0.108881653 0.09605235 PRKAR1A/CDKN1B/SFN/CHP1/NPM1 5

MF GO:0072349 modified amino acid transmembrane transporter activity 3/266 21/18337 0.003308335 0.108881653 0.09605235 ABCC1/GJA1/SLC6A8 3

MF GO:0048156 tau protein binding 4/266 44/18337 0.003726321 0.108881653 0.09605235 FYN/DYRK1A/SMAD2/HSP90AA1 4

MF GO:0051427 hormone receptor binding 8/266 173/18337 0.003818952 0.108881653 0.09605235 PPID/SMARCE1/FYN/TRIP12/SLC30A9/UBA3/RARG/PTPN11 8

MF GO:0061980 regulatory RNA binding 4/266 45/18337 0.00404397 0.108881653 0.09605235 PUM2/MATR3/HNRNPA1/DICER1 4

MF GO:0019210 kinase inhibitor activity 5/266 73/18337 0.004164381 0.108881653 0.09605235 PRKAR1A/CDKN1B/SFN/CHP1/NPM1 5

MF GO:0004857 enzyme inhibitor activity 13/266 385/18337 0.004252061 0.108881653 0.09605235 TXNIP/SCG5/PRKAR1A/IPO5/CDKN1B/GCHFR/TIMP3/SFN/TNK2/TFPI/PHACTR2/CHP1/NPM1 13

MF GO:0004620 phospholipase activity 6/266 105/18337 0.004282873 0.108881653 0.09605235 ENPP2/EDNRA/LYPLA1/PLA2G6/PLA2G4A/PLD3 6

MF GO:0016887 ATPase activity 15/266 478/18337 0.004362537 0.108881653 0.09605235 ABCC1/RFC4/MYO1C/MSH6/KIF2A/VPS4B/MCM6/TOP2A/DHX15/HSPA13/RAD51C/HSP90AA1/DICER1/OLA1/CCT8 15

MF GO:0005178 integrin binding 7/266 142/18337 0.004723632 0.113178215 0.099842657 COL3A1/FN1/LAMB2/FAP/FBN1/CXCL12/HMGB1 7

MF GO:0019207 kinase regulator activity 9/266 220/18337 0.004944411 0.113911621 0.100489647 PRKAR1A/GREM1/CCND2/CDKN1B/SFN/HMGB1/CHP1/CCNG2/NPM1 9

MF GO:0033613 activating transcription factor binding 5/266 78/18337 0.005521595 0.122497599 0.108063957 PRRX1/MEF2A/SMAD2/ATF2/NPM1 5

MF GO:0005048 signal sequence binding 4/266 50/18337 0.005908298 0.126395382 0.111502472 CEMIP/IPO5/TNPO1/NFKBIA 4

MF GO:0019199 transmembrane receptor protein kinase activity 5/266 80/18337 0.006142924 0.126576826 0.111662537 IGF1R/FGFR3/LTBP1/DDR1/EFEMP1 5

MF GO:0008094 DNA-dependent ATPase activity 4/266 51/18337 0.006339407 0.126576826 0.111662537 RFC4/MSH6/TOP2A/RAD51C 4

MF GO:0008139 nuclear localization sequence binding 3/266 27/18337 0.006825019 0.131876985 0.116338189 IPO5/TNPO1/NFKBIA 3

MF GO:0019902 phosphatase binding 8/266 193/18337 0.007293678 0.136528527 0.120441649 CDKN1B/CHCHD3/SMAD2/BAD/CDC27/CDC5L/HSP90B1/PDLIM4 8

MF GO:0051059 NF-kappaB binding 3/266 29/18337 0.008346683 0.149517704 0.131900338 MTDH/NFKBIA/NPM1 3

MF GO:0034511 U3 snoRNA binding 2/266 10/18337 0.008736427 0.149517704 0.131900338 PRKDC/ISG20 2

MF GO:0043560 insulin receptor substrate binding 2/266 10/18337 0.008736427 0.149517704 0.131900338 IGF1R/PTPN11 2

MF GO:0071889 14-3-3 protein binding 3/266 31/18337 0.010052087 0.16435042 0.144985345 BAD/PI4KB/RBM7 3

MF GO:0097493 structural molecule activity conferring elasticity 2/266 11/18337 0.010576324 0.16435042 0.144985345 FBLN2/FBN1 2

MF GO:0102545 phosphatidyl phospholipase B activity 2/266 11/18337 0.010576324 0.16435042 0.144985345 PLA2G6/PLA2G4A 2

MF GO:0031625 ubiquitin protein ligase binding 10/266 293/18337 0.010742413 0.16435042 0.144985345 CUL4B/TXNIP/PRKAR1A/PCBP2/SMAD2/TNK2/HSP90AA1/NFKBIA/LRPPRC/TRIM37 10

MF GO:0030291 protein serine/threonine kinase inhibitor activity 3/266 32/18337 0.010974986 0.16435042 0.144985345 PRKAR1A/CDKN1B/SFN 3

MF GO:0016298 lipase activity 6/266 130/18337 0.011788166 0.166400364 0.146793749 ENPP2/EDNRA/LYPLA1/PLA2G6/PLA2G4A/PLD3 6

MF GO:0004714 transmembrane receptor protein tyrosine kinase activity 4/266 61/18337 0.011833414 0.166400364 0.146793749 IGF1R/FGFR3/DDR1/EFEMP1 4

MF GO:0035198 miRNA binding 3/266 33/18337 0.011945268 0.166400364 0.146793749 PUM2/MATR3/HNRNPA1 3

MF GO:0035256 G protein-coupled glutamate receptor binding 2/266 12/18337 0.012571056 0.167334719 0.147618011 FYN/HOMER3 2

MF GO:0043023 ribosomal large subunit binding 2/266 12/18337 0.012571056 0.167334719 0.147618011 OLA1/NPM1 2

MF GO:0003713 transcription coactivator activity 9/266 258/18337 0.013295595 0.171154896 0.150988064 HTATIP2/MTDH/DCAF6/SMARCE1/DYRK1A/SLC30A9/HMGB1/TRIM37/NPM1 9

MF GO:0016922 nuclear receptor binding 5/266 97/18337 0.013520316 0.171154896 0.150988064 PPID/SMARCE1/SLC30A9/UBA3/RARG 5

MF GO:0019955 cytokine binding 6/266 136/18337 0.014469295 0.171154896 0.150988064 LTBP1/CCR10/GREM1/IFNGR1/IL13RA1/HMGB1 6

MF GO:0005095 GTPase inhibitor activity 2/266 13/18337 0.014715777 0.171154896 0.150988064 IPO5/TNK2 2

MF GO:0031994 insulin-like growth factor I binding 2/266 13/18337 0.014715777 0.171154896 0.150988064 IGFBP6/IGF1R 2

MF GO:0072542 protein phosphatase activator activity 2/266 13/18337 0.014715777 0.171154896 0.150988064 PPP2R5E/PPP2R5C 2

MF GO:0019838 growth factor binding 6/266 137/18337 0.014954136 0.171154896 0.150988064 COL3A1/IGFBP6/IGF1R/FGFR3/LTBP1/FGFBP1 6

MF GO:0000217 DNA secondary structure binding 3/266 36/18337 0.015143922 0.171154896 0.150988064 MSH6/RAD51C/HMGB1 3

MF GO:0044389 ubiquitin-like protein ligase binding 10/266 312/18337 0.016057362 0.178117774 0.157130521 CUL4B/TXNIP/PRKAR1A/PCBP2/SMAD2/TNK2/HSP90AA1/NFKBIA/LRPPRC/TRIM37 10

MF GO:0035257 nuclear hormone receptor binding 6/266 140/18337 0.01647619 0.178716792 0.157658957 PPID/SMARCE1/TRIP12/SLC30A9/UBA3/RARG 6

MF GO:0015562 efflux transmembrane transporter activity 2/266 14/18337 0.01700575 0.178716792 0.157658957 ABCC1/GJA1 2

MF GO:0070851 growth factor receptor binding 6/266 141/18337 0.017006439 0.178716792 0.157658957 PDGFC/FYN/GREM1/FGF18/EFEMP1/TNK2 6

MF GO:0008022 protein C-terminus binding 7/266 185/18337 0.018556159 0.191640334 0.169059744 SIAH1/MYO1C/FN1/GPSM2/VPS4B/TOP2A/ID1 7

MF GO:0051371 muscle alpha-actinin binding 2/266 15/18337 0.019436343 0.197328292 0.174077502 PKD2/PDLIM4 2

MF GO:0004520 endodeoxyribonuclease activity 3/266 40/18337 0.020089589 0.200561068 0.176929367 MBD4/RAD51C/DICER1 3

MF GO:0019903 protein phosphatase binding 6/266 148/18337 0.021050221 0.205934565 0.181669715 CDKN1B/BAD/CDC27/CDC5L/HSP90B1/PDLIM4 6

MF GO:0030020 extracellular matrix structural constituent conferring tensile strength 3/266 41/18337 0.021448585 0.205934565 0.181669715 COL3A1/COL8A1/COL15A1 3

MF GO:0005092 GDP-dissociation inhibitor activity 2/266 16/18337 0.022003025 0.205934565 0.181669715 GPSM2/GDI2 2

MF GO:0019211 phosphatase activator activity 2/266 16/18337 0.022003025 0.205934565 0.181669715 PPP2R5E/PPP2R5C 2

MF GO:0003725 double-stranded RNA binding 4/266 76/18337 0.024613863 0.20839606 0.183841178 MTDH/DHX15/HMGB1/DICER1 4

MF GO:0005001 transmembrane receptor protein tyrosine phosphatase activity 2/266 17/18337 0.024701369 0.20839606 0.183841178 PTPRG/PTPRU 2

MF GO:0019198 transmembrane receptor protein phosphatase activity 2/266 17/18337 0.024701369 0.20839606 0.183841178 PTPRG/PTPRU 2

MF GO:0023026 MHC class II protein complex binding 2/266 17/18337 0.024701369 0.20839606 0.183841178 HSP90AA1/HLA-DMA 2

MF GO:0030275 LRR domain binding 2/266 17/18337 0.024701369 0.20839606 0.183841178 CDC5L/ROBO1 2

MF GO:0043024 ribosomal small subunit binding 2/266 17/18337 0.024701369 0.20839606 0.183841178 MTIF2/NPM1 2

MF GO:0052742 phosphatidylinositol kinase activity 2/266 17/18337 0.024701369 0.20839606 0.183841178 PIK3C3/PI4KB 2

MF GO:0004712 protein serine/threonine/tyrosine kinase activity 3/266 44/18337 0.025820457 0.214811854 0.189501012 DYRK2/DYRK1A/TNK2 3

MF GO:0016755 "transferase activity, transferring amino-acyl groups" 2/266 18/18337 0.027527045 0.222820272 0.196565814 QPCT/F13A1 2

MF GO:0140416 transcription regulator inhibitor activity 2/266 18/18337 0.027527045 0.222820272 0.196565814 ID4/ID1 2

MF GO:0004715 non-membrane spanning protein tyrosine kinase activity 3/266 46/18337 0.028980326 0.231456203 0.204184191 FYN/DYRK1A/TNK2 3

MF GO:0017116 single-stranded DNA helicase activity 2/266 19/18337 0.03047582 0.238067431 0.210016431 RFC4/MCM6 2

MF GO:0008083 growth factor activity 6/266 162/18337 0.030989381 0.238067431 0.210016431 PDGFC/FGF18/JAG1/EFEMP1/GDF15/CXCL12 6

MF GO:0004518 nuclease activity 7/266 206/18337 0.031000433 0.238067431 0.210016431 MBD4/TSN/ENPP2/RAD51C/ISG20/PLD3/DICER1 7

MF GO:0061608 nuclear import signal receptor activity 2/266 20/18337 0.033543557 0.251157387 0.221564024 IPO5/TNPO1 2

MF GO:0070182 DNA polymerase binding 2/266 20/18337 0.033543557 0.251157387 0.221564024 HSP90AA1/HMGB1 2

MF GO:0016538 cyclin-dependent protein serine/threonine kinase regulator activity 3/266 50/18337 0.035884284 0.265366494 0.234098901 CCND2/CDKN1B/CCNG2 3

MF GO:0051428 peptide hormone receptor binding 2/266 21/18337 0.036726213 0.268280504 0.23666956 FYN/PTPN11 2

MF GO:0000287 magnesium ion binding 7/266 216/18337 0.038532248 0.269346966 0.237610363 MSH6/DYRK2/PAPOLA/TOP2A/MTHFD2/SUCLG2/PSPH 7

MF GO:0003727 single-stranded RNA binding 4/266 88/18337 0.039176324 0.269346966 0.237610363 DLX2/HMGB1/HNRNPA1/RBM7 4

MF GO:0004529 exodeoxyribonuclease activity 2/266 22/18337 0.040019833 0.269346966 0.237610363 ISG20/PLD3 2

MF GO:0005158 insulin receptor binding 2/266 22/18337 0.040019833 0.269346966 0.237610363 IGF1R/PTPN11 2

MF GO:0005540 hyaluronic acid binding 2/266 22/18337 0.040019833 0.269346966 0.237610363 TNFAIP6/CEMIP 2

MF GO:0016895 "exodeoxyribonuclease activity, producing 5'-phosphomonoesters" 2/266 22/18337 0.040019833 0.269346966 0.237610363 ISG20/PLD3 2

MF GO:0050321 tau-protein kinase activity 2/266 22/18337 0.040019833 0.269346966 0.237610363 FYN/DYRK1A 2

MF GO:0008081 phosphoric diester hydrolase activity 4/266 89/18337 0.040570348 0.270018206 0.238202512 ENPP2/EDNRA/SMPDL3A/PLD3 4

MF GO:0016779 nucleotidyltransferase activity 5/266 131/18337 0.042257382 0.276690566 0.244088681 PAPOLA/POLA1/POLD3/PRIM1/POLR2B 5

MF GO:0003688 DNA replication origin binding 2/266 23/18337 0.043420556 0.276690566 0.244088681 POLA1/MCM6 2

MF GO:0017134 fibroblast growth factor binding 2/266 23/18337 0.043420556 0.276690566 0.244088681 FGFR3/FGFBP1 2

MF GO:0030552 cAMP binding 2/266 23/18337 0.043420556 0.276690566 0.244088681 PRKAR1A/POPDC3 2

MF GO:0043021 ribonucleoprotein complex binding 5/266 134/18337 0.045813213 0.288864367 0.254828068 SRP72/MTIF2/SRP9/OLA1/NPM1 5

MF GO:0005160 transforming growth factor beta receptor binding 2/266 24/18337 0.046924607 0.292789998 0.25829115 USP15/SMAD2 2

MF GO:0005539 glycosaminoglycan binding 7/266 228/18337 0.049041851 0.302846069 0.267162334 SULF1/POSTN/FN1/TNFAIP6/CEMIP/FBN1/FGFBP1 7
