# Supplementary material for: Rebalance of the Polyamine Metabolism Suppresses Oxidative Stress and Delays Senescence in Nucleus Pulposus Cells
Source: Oxid Med Cell Longev. 2022 Feb 7;2022:8033353. doi: 10.1155/2022/8033353 (PMC8844099; doi:10.1155/2022/8033353)
Supplement: Supplementary Materials — The raw data of the bioinformatics analysis containing DEGs, GO, KEGG, and PPI are provided in the supplementary file. [file 8033353.f1.zip › 8033353.f1/KEGG.docx]

**ID Description GeneRatio BgRatio pvalue p.adjust qvalue geneID Count**

hsa03030 DNA replication 5/151 36/8093 0.000500218 0.103998642 0.093967601 RFC4/POLA1/MCM6/POLD3/PRIM1 5

hsa04151 PI3K-Akt signaling pathway 16/151 354/8093 0.000892692 0.103998642 0.093967601 PDGFC/FN1/IGF1R/FGFR3/PPP2R5E/LAMB2/CCND2/BCL2L1/FGF18/CDKN1B/PPP2R5C/BAD/ANGPT1/HSP90AA1/HSP90B1/ATF2 16

hsa05215 Prostate cancer 7/151 97/8093 0.002164858 0.129959142 0.117424118 PDGFC/IGF1R/CDKN1B/BAD/HSP90AA1/HSP90B1/NFKBIA 7

hsa04110 Cell cycle 8/151 126/8093 0.002392681 0.129959142 0.117424118 CCND2/MCM6/CDKN1B/STAG2/SMAD2/CDC27/SFN/PRKDC 8

hsa04136 Autophagy - other 4/151 32/8093 0.002788823 0.129959142 0.117424118 ATG12/PIK3R4/PIK3C3/ATG3 4

hsa04140 Autophagy - animal 8/151 141/8093 0.004773362 0.185365546 0.167486375 ATG12/IGF1R/BCL2L1/PIK3R4/PIK3C3/BAD/ATG3/HMGB1 8

hsa04658 Th1 and Th2 cell differentiation 6/151 92/8093 0.007297997 0.222019925 0.200605308 RUNX3/JAG1/NFKBIA/IFNGR1/RBPJ/HLA-DMA 6

hsa04350 TGF-beta signaling pathway 6/151 94/8093 0.008084911 0.222019925 0.200605308 LTBP1/ID4/GREM1/ID1/SMAD2/FBN1 6

hsa03430 Mismatch repair 3/151 23/8093 0.008575877 0.222019925 0.200605308 RFC4/MSH6/POLD3 3

hsa04014 Ras signaling pathway 10/151 232/8093 0.01138784 0.242389791 0.219010428 PDGFC/IGF1R/FGFR3/BCL2L1/FGF18/BAD/ANGPT1/PLA2G6/PLA2G4A/PTPN11 10

hsa04115 p53 signaling pathway 5/151 73/8093 0.011492577 0.242389791 0.219010428 SIAH1/CCND2/BCL2L1/SFN/CCNG2 5

hsa00565 Ether lipid metabolism 4/151 49/8093 0.012874413 0.242389791 0.219010428 ENPP2/PLA2G6/PLA2G4A/PLD3 4

hsa05220 Chronic myeloid leukemia 5/151 76/8093 0.013523894 0.242389791 0.219010428 BCL2L1/CDKN1B/BAD/NFKBIA/PTPN11 5

hsa01521 EGFR tyrosine kinase inhibitor resistance 5/151 79/8093 0.015784732 0.260637302 0.235497901 PDGFC/IGF1R/FGFR3/BCL2L1/BAD 5

hsa04120 Ubiquitin mediated proteolysis 7/151 142/8093 0.016779225 0.260637302 0.235497901 CUL4B/SIAH1/TRIP12/CDC27/UBA3/FBXO2/TRIM37 7

hsa05145 Toxoplasmosis 6/151 112/8093 0.018111052 0.263742191 0.238303313 LAMB2/BCL2L1/BAD/NFKBIA/IFNGR1/HLA-DMA 6

hsa03410 Base excision repair 3/151 33/8093 0.023079105 0.310499685 0.280550879 MBD4/POLD3/HMGB1 3

hsa04330 Notch signaling pathway 4/151 59/8093 0.0239871 0.310499685 0.280550879 RFNG/TLE2/JAG1/RBPJ 4

hsa05222 Small cell lung cancer 5/151 92/8093 0.02846145 0.3285902 0.296896498 FN1/LAMB2/BCL2L1/CDKN1B/NFKBIA 5

hsa04510 Focal adhesion 8/151 201/8093 0.034219833 0.3285902 0.296896498 PDGFC/FN1/IGF1R/FYN/LAMB2/CCND2/BAD/MYL12A 8

hsa04068 FoxO signaling pathway 6/151 131/8093 0.035598858 0.3285902 0.296896498 ATG12/IGF1R/HOMER3/CCND2/CDKN1B/CCNG2 6

hsa00564 Glycerophospholipid metabolism 5/151 98/8093 0.036006472 0.3285902 0.296896498 LPCAT3/LYPLA1/PLA2G6/PLA2G4A/PLD3 5

hsa01522 Endocrine resistance 5/151 98/8093 0.036006472 0.3285902 0.296896498 IGF1R/CDKN1B/BAD/JAG1/MMP2 5

hsa03015 mRNA surveillance pathway 5/151 98/8093 0.036006472 0.3285902 0.296896498 HBS1L/PPP2R5E/PAPOLA/UPF3A/PPP2R5C 5

hsa00260 "Glycine, serine and threonine metabolism" 3/151 40/8093 0.038048017 0.3285902 0.296896498 DLD/CTH/PSPH 3

hsa04933 AGE-RAGE signaling pathway in diabetic complications 5/151 100/8093 0.03877092 0.3285902 0.296896498 COL3A1/FN1/CDKN1B/SMAD2/MMP2 5

hsa04141 Protein processing in endoplasmic reticulum 7/151 171/8093 0.040685373 0.3285902 0.296896498 DNAJC10/SEC24D/CANX/HSP90AA1/HSP90B1/UBXN4/FBXO2 7

hsa05131 Shigellosis 9/151 247/8093 0.041320777 0.3285902 0.296896498 ATG12/CASP4/PPID/BCL2L1/PIK3R4/PIK3C3/ACTR2/NFKBIA/MYL12A 9

hsa04371 Apelin signaling pathway 6/151 138/8093 0.044095889 0.3285902 0.296896498 MEF2A/PIK3R4/SMAD2/PIK3C3/JAG1/TFAM 6

hsa05207 Chemical carcinogenesis - receptor activation 8/151 212/8093 0.044604005 0.3285902 0.296896498 FGF18/EPHX1/BAD/JAG1/HSP90AA1/HSP90B1/CYP1B1/ATF2 8

hsa05165 Human papillomavirus infection 11/151 331/8093 0.045054492 0.3285902 0.296896498 FN1/CSNK1A1/RFNG/PPP2R5E/LAMB2/CCND2/CDKN1B/PPP2R5C/BAD/JAG1/RBPJ 11

hsa05218 Melanoma 4/151 72/8093 0.045128268 0.3285902 0.296896498 PDGFC/IGF1R/FGF18/BAD 4

hsa01524 Platinum drug resistance 4/151 73/8093 0.047078163 0.330838967 0.298928364 MSH6/BCL2L1/TOP2A/BAD 4
