# Supplementary material for: Rebalance of the Polyamine Metabolism Suppresses Oxidative Stress and Delays Senescence in Nucleus Pulposus Cells
Source: Oxid Med Cell Longev. 2022 Feb 7;2022:8033353. doi: 10.1155/2022/8033353 (PMC8844099; doi:10.1155/2022/8033353)
Supplement: Supplementary Materials — The raw data of the bioinformatics analysis containing DEGs, GO, KEGG, and PPI are provided in the supplementary file. [file 8033353.f1.zip › 8033353.f1/string_interactions.docx]

**#node1 node2 node1_string_id node2_string_id neighborhood_on_chromosome gene_fusion phylogenetic_cooccurrence homology coexpression experimentally_determined_interaction database_annotated automated_textmining combined_score**

ACAN COL1A1 9606.ENSP00000387356 9606.ENSP00000225964 0 0 0 0 0.062 0 0 0.762 0.767

ACAN MMP8 9606.ENSP00000387356 9606.ENSP00000236826 0 0 0 0 0 0.228 0 0.638 0.709

ACAN ADAMTS2 9606.ENSP00000387356 9606.ENSP00000251582 0 0 0 0 0.065 0 0 0.481 0.494

ACAN MMP13 9606.ENSP00000387356 9606.ENSP00000260302 0 0 0 0 0 0.06 0.9 0.883 0.988

ACAN TIMP2 9606.ENSP00000387356 9606.ENSP00000262768 0 0 0 0 0.062 0 0 0.555 0.565

ACAN TIMP3 9606.ENSP00000387356 9606.ENSP00000266085 0 0 0 0 0.062 0 0 0.63 0.638

ACAN MMP10 9606.ENSP00000387356 9606.ENSP00000279441 0 0 0 0 0 0.06 0 0.621 0.629

ACAN MMP3 9606.ENSP00000387356 9606.ENSP00000299855 0 0 0 0 0 0.228 0.9 0.835 0.986

ACAN MMP14 9606.ENSP00000387356 9606.ENSP00000308208 0 0 0 0 0 0.279 0 0.567 0.675

ACAN MMP19 9606.ENSP00000387356 9606.ENSP00000313437 0 0 0 0 0 0.262 0 0.348 0.498

ACAN MMP1 9606.ENSP00000387356 9606.ENSP00000322788 0 0 0 0 0 0.228 0.9 0.697 0.974

ACAN RUNX2 9606.ENSP00000387356 9606.ENSP00000360493 0 0 0 0 0 0 0 0.837 0.837

ACAN MMP9 9606.ENSP00000387356 9606.ENSP00000361405 0 0 0 0 0 0.06 0.9 0.664 0.965

ACAN COL2A1 9606.ENSP00000387356 9606.ENSP00000369889 0 0 0 0.541 0.063 0 0 0.9 0.448

ACOX1 DAO 9606.ENSP00000293217 9606.ENSP00000228476 0 0 0 0 0.062 0 0.9 0.373 0.936

ACOX1 CAT 9606.ENSP00000293217 9606.ENSP00000241052 0.069 0 0 0 0.112 0.744 0.9 0.645 0.991

ACOX1 NOX4 9606.ENSP00000293217 9606.ENSP00000263317 0 0 0 0 0 0 0 0.499 0.499

ACOX1 PPARGC1A 9606.ENSP00000293217 9606.ENSP00000264867 0 0 0 0 0.062 0 0.9 0.647 0.964

ACOX1 PAOX 9606.ENSP00000293217 9606.ENSP00000278060 0 0 0 0 0.049 0 0.9 0.202 0.917

ACOX1 UCP2 9606.ENSP00000293217 9606.ENSP00000312029 0 0 0 0 0 0 0 0.572 0.572

ACOX1 AOX1 9606.ENSP00000293217 9606.ENSP00000363832 0 0 0 0 0.063 0 0.8 0.408 0.879

ACOX1 SP1 9606.ENSP00000293217 9606.ENSP00000329357 0 0 0 0 0 0.057 0.9 0.048 0.902

ACOX1 NOS2 9606.ENSP00000293217 9606.ENSP00000327251 0 0 0 0 0.063 0 0.9 0.095 0.907

ACOX1 DDO 9606.ENSP00000293217 9606.ENSP00000357920 0 0 0 0 0.062 0 0.9 0.252 0.923

ACOX1 ACOX3 9606.ENSP00000293217 9606.ENSP00000348775 0 0 0.415 0.765 0.076 0 0.9 0.863 0.93

ACOX1 HAO1 9606.ENSP00000293217 9606.ENSP00000368066 0.045 0 0 0 0.085 0 0.9 0.391 0.939

ACOX1 PIPOX 9606.ENSP00000293217 9606.ENSP00000317721 0 0 0 0 0.063 0 0.9 0.549 0.954

ACOX1 HAO2 9606.ENSP00000293217 9606.ENSP00000483507 0.045 0 0 0 0.085 0 0.9 0.567 0.957

ACOX3 DAO 9606.ENSP00000348775 9606.ENSP00000228476 0 0 0 0 0.049 0 0.9 0.189 0.916

ACOX3 CAT 9606.ENSP00000348775 9606.ENSP00000241052 0.069 0 0 0 0.122 0.303 0.9 0.543 0.969

ACOX3 PAOX 9606.ENSP00000348775 9606.ENSP00000278060 0 0 0 0 0.058 0 0.9 0.156 0.913

ACOX3 ACOX1 9606.ENSP00000348775 9606.ENSP00000293217 0 0 0.415 0.765 0.076 0 0.9 0.863 0.93

ACOX3 PIPOX 9606.ENSP00000348775 9606.ENSP00000317721 0 0 0 0 0.062 0 0.9 0.596 0.958

ACOX3 NOS2 9606.ENSP00000348775 9606.ENSP00000327251 0 0 0 0 0.063 0 0.9 0.063 0.904

ACOX3 AOX1 9606.ENSP00000348775 9606.ENSP00000363832 0 0 0 0 0.064 0 0.8 0.162 0.829

ACOX3 DDO 9606.ENSP00000348775 9606.ENSP00000357920 0 0 0 0 0.052 0 0.9 0.101 0.907

ACOX3 HAO2 9606.ENSP00000348775 9606.ENSP00000483507 0.045 0 0 0 0.062 0 0.9 0.518 0.951

ACOX3 HAO1 9606.ENSP00000348775 9606.ENSP00000368066 0.045 0 0 0 0.064 0 0.9 0.542 0.953

ADAMTS2 COL1A1 9606.ENSP00000251582 9606.ENSP00000225964 0 0 0 0 0.334 0.153 0.9 0.536 0.97

ADAMTS2 LOX 9606.ENSP00000251582 9606.ENSP00000231004 0 0 0 0 0.17 0.064 0 0.439 0.526

ADAMTS2 TIMP2 9606.ENSP00000251582 9606.ENSP00000262768 0 0 0 0 0.096 0 0 0.397 0.432

ADAMTS2 TIMP3 9606.ENSP00000251582 9606.ENSP00000266085 0 0 0 0 0.107 0 0 0.422 0.463

ADAMTS2 ACAN 9606.ENSP00000251582 9606.ENSP00000387356 0 0 0 0 0.065 0 0 0.481 0.494

ADAMTS2 COL2A1 9606.ENSP00000251582 9606.ENSP00000369889 0 0 0 0 0.063 0.153 0.9 0.391 0.945

AOC1 DAO 9606.ENSP00000411613 9606.ENSP00000228476 0 0 0 0 0.063 0.213 0 0.496 0.596

AOC1 CAT 9606.ENSP00000411613 9606.ENSP00000241052 0 0 0 0 0.062 0 0 0.458 0.469

AOC1 PAOX 9606.ENSP00000411613 9606.ENSP00000278060 0 0 0 0 0 0 0 0.749 0.749

AOC1 MAOA 9606.ENSP00000411613 9606.ENSP00000340684 0 0 0 0 0.057 0 0.8 0.502 0.897

AOC1 MAOB 9606.ENSP00000411613 9606.ENSP00000367309 0 0 0 0 0.057 0 0.8 0.548 0.907

AOC1 ATOX1 9606.ENSP00000411613 9606.ENSP00000430598 0 0 0 0 0 0.144 0 0.373 0.44

AOC1 SMOX 9606.ENSP00000411613 9606.ENSP00000478305 0 0 0 0 0 0 0 0.536 0.536

AOC1 IL4I1 9606.ENSP00000411613 9606.ENSP00000472474 0 0 0 0 0.055 0 0.9 0.063 0.903

AOC2 DAO 9606.ENSP00000253799 9606.ENSP00000228476 0 0 0 0 0.063 0 0 0.432 0.445

AOC2 LOX 9606.ENSP00000253799 9606.ENSP00000231004 0 0 0 0 0 0 0 0.423 0.422

AOC2 CAT 9606.ENSP00000253799 9606.ENSP00000241052 0 0 0 0 0.062 0 0 0.395 0.408

AOC2 ATOX1 9606.ENSP00000253799 9606.ENSP00000430598 0 0 0 0 0 0.144 0 0.416 0.478

AOC2 SMOX 9606.ENSP00000253799 9606.ENSP00000478305 0 0 0 0 0 0 0 0.48 0.48

AOC2 TIMP2 9606.ENSP00000253799 9606.ENSP00000262768 0 0 0 0 0 0 0 0.63 0.63

AOC2 AOC3 9606.ENSP00000253799 9606.ENSP00000312326 0 0 0 0.969 0.139 0.229 0.8 0.909 0.859

AOC2 MAOB 9606.ENSP00000253799 9606.ENSP00000367309 0 0 0 0 0.055 0 0.8 0.757 0.95

AOC2 MAOA 9606.ENSP00000253799 9606.ENSP00000340684 0 0 0 0 0.055 0 0.8 0.763 0.951

AOC3 AOC2 9606.ENSP00000312326 9606.ENSP00000253799 0 0 0 0.969 0.139 0.229 0.8 0.909 0.859

AOC3 TIMP3 9606.ENSP00000312326 9606.ENSP00000266085 0 0 0 0 0.061 0 0 0.427 0.439

AOC3 CYP1A1 9606.ENSP00000312326 9606.ENSP00000369050 0 0 0 0 0.063 0 0 0.412 0.425

AOC3 PIPOX 9606.ENSP00000312326 9606.ENSP00000317721 0 0 0 0 0.051 0 0 0.436 0.442

AOC3 ATOX1 9606.ENSP00000312326 9606.ENSP00000430598 0 0 0 0 0 0.144 0 0.394 0.459

AOC3 MAOA 9606.ENSP00000312326 9606.ENSP00000340684 0 0 0 0 0.082 0 0.8 0.557 0.911

AOC3 MAOB 9606.ENSP00000312326 9606.ENSP00000367309 0 0 0 0 0.089 0 0.8 0.556 0.912

AOX1 PNPO 9606.ENSP00000363832 9606.ENSP00000225573 0 0 0 0 0 0 0.9 0.269 0.923

AOX1 CAT 9606.ENSP00000363832 9606.ENSP00000241052 0 0 0 0 0.062 0 0 0.428 0.44

AOX1 ACOX1 9606.ENSP00000363832 9606.ENSP00000293217 0 0 0 0 0.063 0 0.8 0.408 0.879

AOX1 MAOA 9606.ENSP00000363832 9606.ENSP00000340684 0 0 0 0 0.096 0 0.9 0.336 0.934

AOX1 ACOX3 9606.ENSP00000363832 9606.ENSP00000348775 0 0 0 0 0.064 0 0.8 0.162 0.829

AOX1 SRXN1 9606.ENSP00000363832 9606.ENSP00000371388 0 0 0 0 0 0 0 0.434 0.434

AOX1 TXNRD1 9606.ENSP00000363832 9606.ENSP00000434516 0 0 0 0 0 0 0 0.531 0.531

AOX1 SUOX 9606.ENSP00000363832 9606.ENSP00000377668 0 0 0 0 0 0 0 0.876 0.876

AOX1 CYP1A1 9606.ENSP00000363832 9606.ENSP00000369050 0 0 0 0 0.065 0 0.9 0.277 0.926

AOX1 MAOB 9606.ENSP00000363832 9606.ENSP00000367309 0 0 0 0 0.062 0 0.9 0.316 0.93

APOA4 PON1 9606.ENSP00000350425 9606.ENSP00000222381 0 0 0 0 0.076 0 0.72 0.576 0.88

APOA4 MMP3 9606.ENSP00000350425 9606.ENSP00000299855 0 0 0 0 0.062 0 0 0.418 0.43

APOA4 PIPOX 9606.ENSP00000350425 9606.ENSP00000317721 0 0 0 0 0.521 0 0 0 0.521

APOA4 HAO2 9606.ENSP00000350425 9606.ENSP00000483507 0 0 0 0 0.536 0 0 0.114 0.572

APOA4 VIMP 9606.ENSP00000350425 9606.ENSP00000381282 0 0 0 0 0 0 0.72 0 0.72

APOA4 PCYOX1 9606.ENSP00000350425 9606.ENSP00000387654 0 0 0 0 0 0 0.72 0.135 0.747

APTX ERCC1 9606.ENSP00000400806 9606.ENSP00000013807 0 0 0 0 0.062 0.121 0 0.555 0.601

APTX ERCC3 9606.ENSP00000400806 9606.ENSP00000285398 0 0 0 0 0.06 0 0 0.482 0.492

APTX PNKP 9606.ENSP00000400806 9606.ENSP00000323511 0 0 0 0.608 0 0.115 0 0.907 0.428

APTX ERCC6 9606.ENSP00000400806 9606.ENSP00000348089 0 0 0 0 0.062 0.058 0 0.476 0.497

APTX ERCC2 9606.ENSP00000400806 9606.ENSP00000375809 0 0 0 0 0.073 0 0 0.501 0.518

ATOX1 LOX 9606.ENSP00000430598 9606.ENSP00000231004 0 0 0 0 0 0.168 0 0.43 0.506

ATOX1 GFER 9606.ENSP00000430598 9606.ENSP00000248114 0 0 0 0 0.062 0 0 0.431 0.443

ATOX1 AOC2 9606.ENSP00000430598 9606.ENSP00000253799 0 0 0 0 0 0.144 0 0.416 0.478

ATOX1 PRDX5 9606.ENSP00000430598 9606.ENSP00000265462 0 0 0 0 0.082 0 0 0.494 0.516

ATOX1 SOD1 9606.ENSP00000430598 9606.ENSP00000270142 0 0 0 0 0.075 0.495 0 0.772 0.884

ATOX1 AOC3 9606.ENSP00000430598 9606.ENSP00000312326 0 0 0 0 0 0.144 0 0.394 0.459

ATOX1 SOD3 9606.ENSP00000430598 9606.ENSP00000371554 0 0 0 0 0.062 0.336 0.9 0.722 0.98

ATOX1 SUOX 9606.ENSP00000430598 9606.ENSP00000377668 0 0 0 0 0 0 0 0.648 0.648

ATOX1 NUDT1 9606.ENSP00000430598 9606.ENSP00000380241 0 0 0 0 0.082 0 0 0.664 0.679

ATOX1 AOC1 9606.ENSP00000430598 9606.ENSP00000411613 0 0 0 0 0 0.144 0 0.373 0.44

ATOX1 SOD2 9606.ENSP00000430598 9606.ENSP00000446252 0 0 0 0 0.107 0.243 0 0.266 0.46

CAT NOX3 9606.ENSP00000241052 9606.ENSP00000159060 0 0 0 0 0 0 0 0.593 0.593

CAT HMOX1 9606.ENSP00000241052 9606.ENSP00000216117 0 0 0 0 0.062 0.101 0 0.882 0.892

CAT TXN2 9606.ENSP00000241052 9606.ENSP00000216185 0 0 0 0 0.122 0.151 0 0.656 0.721

CAT GSS 9606.ENSP00000241052 9606.ENSP00000216951 0 0 0 0 0.063 0.26 0 0.477 0.606

CAT GSR 9606.ENSP00000241052 9606.ENSP00000221130 0 0 0 0 0.218 0.26 0 0.953 0.97

CAT PON1 9606.ENSP00000241052 9606.ENSP00000222381 0 0 0 0 0.062 0 0 0.596 0.604

CAT PON2 9606.ENSP00000241052 9606.ENSP00000222572 0 0 0 0 0.063 0 0 0.391 0.404

CAT MPO 9606.ENSP00000241052 9606.ENSP00000225275 0 0 0 0 0.061 0.241 0 0.702 0.769

CAT DAO 9606.ENSP00000241052 9606.ENSP00000228476 0.088 0 0 0 0.062 0 0.9 0.512 0.952

CAT GCLC 9606.ENSP00000241052 9606.ENSP00000229416 0 0 0 0 0.143 0.26 0 0.859 0.903

CAT MAPK14 9606.ENSP00000241052 9606.ENSP00000229795 0 0 0 0 0 0.117 0 0.507 0.546

CAT LOX 9606.ENSP00000241052 9606.ENSP00000231004 0 0 0 0 0 0 0 0.469 0.469

CAT AOC2 9606.ENSP00000241052 9606.ENSP00000253799 0 0 0 0 0.062 0 0 0.395 0.408

CAT GSTT2B 9606.ENSP00000241052 9606.ENSP00000290765 0 0 0 0 0.123 0 0 0.363 0.418

CAT CDKN2A 9606.ENSP00000241052 9606.ENSP00000418915 0.049 0 0 0 0.065 0.063 0 0.388 0.422

CAT AOX1 9606.ENSP00000241052 9606.ENSP00000363832 0 0 0 0 0.062 0 0 0.428 0.44

CAT MAOA 9606.ENSP00000241052 9606.ENSP00000340684 0 0 0 0 0.062 0 0 0.446 0.459

CAT AOC1 9606.ENSP00000241052 9606.ENSP00000411613 0 0 0 0 0.062 0 0 0.458 0.469

CAT IL4I1 9606.ENSP00000241052 9606.ENSP00000472474 0 0 0 0 0.062 0 0 0.46 0.472

CAT CYP1A1 9606.ENSP00000241052 9606.ENSP00000369050 0.047 0 0 0 0.062 0 0 0.492 0.507

CAT MAOB 9606.ENSP00000241052 9606.ENSP00000367309 0 0 0 0 0.062 0 0 0.503 0.514

CAT FOS 9606.ENSP00000241052 9606.ENSP00000306245 0 0 0 0 0.062 0 0 0.528 0.539

CAT MTHFR 9606.ENSP00000241052 9606.ENSP00000365777 0.199 0 0 0 0.049 0 0 0.456 0.549

CAT CTH 9606.ENSP00000241052 9606.ENSP00000359976 0 0 0 0 0.097 0 0 0.525 0.552

CAT MMP9 9606.ENSP00000241052 9606.ENSP00000361405 0 0 0 0 0 0 0 0.566 0.566

CAT GSTA1 9606.ENSP00000241052 9606.ENSP00000335620 0 0 0 0 0.062 0.178 0 0.506 0.586

CAT NOS1 9606.ENSP00000241052 9606.ENSP00000477999 0 0 0 0 0.049 0 0 0.598 0.602

CAT GSTP1 9606.ENSP00000241052 9606.ENSP00000381607 0 0 0 0 0.053 0.178 0 0.535 0.606

CAT UCP2 9606.ENSP00000241052 9606.ENSP00000312029 0 0 0 0 0.062 0 0 0.598 0.607

CAT SUOX 9606.ENSP00000241052 9606.ENSP00000377668 0 0.01 0 0 0.065 0 0 0.604 0.614

CAT NCF2 9606.ENSP00000241052 9606.ENSP00000356505 0 0 0 0 0.069 0 0 0.612 0.623

CAT GSTM1 9606.ENSP00000241052 9606.ENSP00000311469 0 0 0 0 0.056 0.178 0 0.556 0.625

CAT NOX5 9606.ENSP00000241052 9606.ENSP00000373518 0 0 0 0 0 0 0 0.654 0.654

CAT CYBA 9606.ENSP00000241052 9606.ENSP00000261623 0 0 0 0 0 0 0 0.658 0.658

CAT GSTM3 9606.ENSP00000241052 9606.ENSP00000256594 0 0 0 0 0.053 0.178 0 0.604 0.665

CAT HMOX2 9606.ENSP00000241052 9606.ENSP00000477572 0 0 0 0 0.062 0.101 0 0.636 0.666

CAT GCLM 9606.ENSP00000241052 9606.ENSP00000359258 0.053 0 0 0 0.133 0.058 0 0.633 0.678

CAT DUOX2 9606.ENSP00000241052 9606.ENSP00000475084 0 0 0 0 0 0 0 0.706 0.706

CAT MSRA 9606.ENSP00000241052 9606.ENSP00000313921 0 0 0 0 0.062 0.219 0 0.646 0.718

CAT PPARGC1A 9606.ENSP00000241052 9606.ENSP00000264867 0 0 0 0 0 0 0 0.718 0.718

CAT TXNRD2 9606.ENSP00000241052 9606.ENSP00000383365 0 0 0 0 0.109 0.222 0 0.634 0.724

CAT NOS3 9606.ENSP00000241052 9606.ENSP00000297494 0 0 0 0 0.049 0 0 0.744 0.746

CAT NOX1 9606.ENSP00000241052 9606.ENSP00000362057 0 0 0 0 0 0 0 0.749 0.749

CAT DUOX1 9606.ENSP00000241052 9606.ENSP00000317997 0 0 0 0 0 0 0 0.749 0.749

CAT NQO1 9606.ENSP00000241052 9606.ENSP00000319788 0 0 0 0 0.062 0 0 0.755 0.76

CAT PARK7 9606.ENSP00000241052 9606.ENSP00000418770 0.12 0 0 0 0.168 0.253 0 0.641 0.777

CAT GLRX2 9606.ENSP00000241052 9606.ENSP00000356410 0.06 0 0 0 0.138 0.221 0 0.692 0.779

CAT NOX4 9606.ENSP00000241052 9606.ENSP00000263317 0 0 0 0 0.062 0 0 0.789 0.793

CAT CYBB 9606.ENSP00000241052 9606.ENSP00000367851 0 0 0 0 0.067 0 0 0.79 0.795

CAT NFE2L2 9606.ENSP00000241052 9606.ENSP00000380252 0 0 0 0 0 0 0 0.812 0.812

CAT SRXN1 9606.ENSP00000241052 9606.ENSP00000371388 0 0 0 0 0.344 0 0 0.736 0.819

CAT PRDX6 9606.ENSP00000241052 9606.ENSP00000342026 0.051 0 0 0 0.268 0.213 0 0.795 0.873

CAT TXNRD1 9606.ENSP00000241052 9606.ENSP00000434516 0.142 0 0 0 0.171 0.363 0 0.804 0.899

CAT PRDX2 9606.ENSP00000241052 9606.ENSP00000301522 0.051 0 0 0 0.268 0.436 0 0.797 0.91

CAT GPX3 9606.ENSP00000241052 9606.ENSP00000373477 0 0 0 0 0.142 0.349 0 0.865 0.918

CAT GPX4 9606.ENSP00000241052 9606.ENSP00000346103 0 0 0 0 0.142 0.349 0 0.869 0.92

CAT PIPOX 9606.ENSP00000241052 9606.ENSP00000317721 0.088 0 0 0 0.062 0 0.9 0.253 0.927

CAT PRDX5 9606.ENSP00000241052 9606.ENSP00000265462 0.107 0 0 0 0 0.619 0 0.809 0.929

CAT DDO 9606.ENSP00000241052 9606.ENSP00000357920 0.088 0 0 0 0.062 0 0.9 0.29 0.931

CAT HAO2 9606.ENSP00000241052 9606.ENSP00000483507 0 0 0 0 0.112 0 0.9 0.32 0.934

CAT TXN 9606.ENSP00000241052 9606.ENSP00000363641 0 0 0 0 0.138 0.137 0 0.92 0.935

CAT PAOX 9606.ENSP00000241052 9606.ENSP00000278060 0 0 0 0 0 0 0.9 0.455 0.943

CAT GPX1 9606.ENSP00000241052 9606.ENSP00000407375 0 0 0 0 0.142 0.349 0 0.919 0.951

CAT NOS2 9606.ENSP00000241052 9606.ENSP00000327251 0 0 0 0 0.049 0 0.9 0.558 0.954

CAT ACOX3 9606.ENSP00000241052 9606.ENSP00000348775 0.069 0 0 0 0.122 0.303 0.9 0.543 0.969

CAT HAO1 9606.ENSP00000241052 9606.ENSP00000368066 0 0 0 0 0.112 0 0.9 0.811 0.981

CAT SOD3 9606.ENSP00000241052 9606.ENSP00000371554 0.111 0 0 0 0.124 0.339 0.9 0.845 0.99

CAT ACOX1 9606.ENSP00000241052 9606.ENSP00000293217 0.069 0 0 0 0.112 0.744 0.9 0.645 0.991

CAT SOD2 9606.ENSP00000241052 9606.ENSP00000446252 0.07 0 0 0 0.079 0.401 0.9 0.943 0.996

CAT SOD1 9606.ENSP00000241052 9606.ENSP00000270142 0.111 0 0 0 0.124 0.52 0.9 0.923 0.996

CDKN2A ERCC1 9606.ENSP00000418915 9606.ENSP00000013807 0 0 0 0 0 0 0 0.401 0.4

CDKN2A MAPK14 9606.ENSP00000418915 9606.ENSP00000229795 0 0 0 0 0 0.259 0.8 0.324 0.891

CDKN2A LOX 9606.ENSP00000418915 9606.ENSP00000231004 0 0 0 0 0.089 0 0 0.466 0.492

CDKN2A CAT 9606.ENSP00000418915 9606.ENSP00000241052 0.049 0 0 0 0.065 0.063 0 0.388 0.422

CDKN2A TIMP3 9606.ENSP00000418915 9606.ENSP00000266085 0 0 0 0 0 0 0 0.632 0.632

CDKN2A FOS 9606.ENSP00000418915 9606.ENSP00000306245 0 0 0 0 0 0.056 0 0.517 0.525

CDKN2A GSTM1 9606.ENSP00000418915 9606.ENSP00000311469 0 0 0 0 0.062 0.091 0 0.362 0.409

CDKN2A SP1 9606.ENSP00000418915 9606.ENSP00000329357 0 0 0 0 0.049 0.298 0 0.643 0.741

CDKN2A MAPK10 9606.ENSP00000418915 9606.ENSP00000352157 0 0 0 0 0 0.316 0 0.7 0.786

CDKN2A RUNX2 9606.ENSP00000418915 9606.ENSP00000360493 0 0 0 0 0 0 0 0.421 0.42

CDKN2A MMP9 9606.ENSP00000418915 9606.ENSP00000361405 0 0 0 0 0 0 0 0.505 0.505

CDKN2A MTHFR 9606.ENSP00000418915 9606.ENSP00000365777 0 0 0 0 0 0.103 0 0.402 0.441

CDKN2A NFE2L2 9606.ENSP00000418915 9606.ENSP00000380252 0 0 0 0 0 0 0 0.492 0.491

CDKN2A GSTP1 9606.ENSP00000418915 9606.ENSP00000381607 0 0 0 0 0.062 0.091 0 0.681 0.704

CDKN2A IPCEF1 9606.ENSP00000418915 9606.ENSP00000394751 0 0 0 0 0 0.078 0 0.428 0.451

COL1A1 MMP10 9606.ENSP00000225964 9606.ENSP00000279441 0 0 0 0 0.065 0.059 0 0.402 0.428

COL1A1 MMP8 9606.ENSP00000225964 9606.ENSP00000236826 0 0 0 0 0 0.059 0 0.455 0.466

COL1A1 MMP14 9606.ENSP00000225964 9606.ENSP00000308208 0 0 0 0 0.287 0.059 0 0.448 0.597

COL1A1 TIMP2 9606.ENSP00000225964 9606.ENSP00000262768 0 0 0 0 0.118 0 0 0.575 0.61

COL1A1 TIMP3 9606.ENSP00000225964 9606.ENSP00000266085 0 0 0 0 0.219 0 0 0.538 0.624

COL1A1 MMP1 9606.ENSP00000225964 9606.ENSP00000322788 0 0 0 0 0.128 0.059 0 0.611 0.653

COL1A1 MMP3 9606.ENSP00000225964 9606.ENSP00000299855 0 0 0 0 0.159 0.059 0 0.617 0.671

COL1A1 MMP13 9606.ENSP00000225964 9606.ENSP00000260302 0 0 0 0 0.062 0.059 0 0.686 0.699

COL1A1 MMP9 9606.ENSP00000225964 9606.ENSP00000361405 0 0 0 0 0.077 0.227 0 0.639 0.72

COL1A1 COL2A1 9606.ENSP00000225964 9606.ENSP00000369889 0 0 0.445 0.971 0.085 0.043 0.72 0.858 0.743

COL1A1 SP1 9606.ENSP00000225964 9606.ENSP00000329357 0 0 0 0 0 0.282 0 0.676 0.757

COL1A1 ACAN 9606.ENSP00000225964 9606.ENSP00000387356 0 0 0 0 0.062 0 0 0.762 0.767

COL1A1 LOX 9606.ENSP00000225964 9606.ENSP00000231004 0 0 0 0 0.409 0.192 0 0.632 0.809

COL1A1 ADAMTS2 9606.ENSP00000225964 9606.ENSP00000251582 0 0 0 0 0.334 0.153 0.9 0.536 0.97

COL1A1 RUNX2 9606.ENSP00000225964 9606.ENSP00000360493 0 0 0 0 0 0 0.9 0.857 0.985

COL2A1 COL1A1 9606.ENSP00000369889 9606.ENSP00000225964 0 0 0.445 0.971 0.085 0.043 0.72 0.858 0.743

COL2A1 ADAMTS2 9606.ENSP00000369889 9606.ENSP00000251582 0 0 0 0 0.063 0.153 0.9 0.391 0.945

COL2A1 MMP13 9606.ENSP00000369889 9606.ENSP00000260302 0 0 0 0 0 0.227 0 0.833 0.865

COL2A1 MMP3 9606.ENSP00000369889 9606.ENSP00000299855 0 0 0 0 0 0.059 0 0.651 0.657

COL2A1 MMP1 9606.ENSP00000369889 9606.ENSP00000322788 0 0 0 0 0 0.227 0 0.477 0.579

COL2A1 RUNX2 9606.ENSP00000369889 9606.ENSP00000360493 0 0 0 0 0 0 0 0.822 0.822

COL2A1 MMP9 9606.ENSP00000369889 9606.ENSP00000361405 0 0 0 0 0 0.146 0 0.479 0.536

COL2A1 ACAN 9606.ENSP00000369889 9606.ENSP00000387356 0 0 0 0.541 0.063 0 0 0.9 0.448

CTH HMOX1 9606.ENSP00000359976 9606.ENSP00000216117 0 0 0 0 0 0 0 0.518 0.518

CTH GSS 9606.ENSP00000359976 9606.ENSP00000216951 0 0 0 0 0.062 0 0 0.631 0.639

CTH GSR 9606.ENSP00000359976 9606.ENSP00000221130 0 0 0 0 0.084 0 0 0.456 0.481

CTH DAO 9606.ENSP00000359976 9606.ENSP00000228476 0.055 0 0 0 0.105 0 0 0.647 0.675

CTH GCLC 9606.ENSP00000359976 9606.ENSP00000229416 0 0 0 0 0.161 0.727 0.8 0.627 0.98

CTH CAT 9606.ENSP00000359976 9606.ENSP00000241052 0 0 0 0 0.097 0 0 0.525 0.552

CTH NOX4 9606.ENSP00000359976 9606.ENSP00000263317 0 0 0 0 0.062 0 0 0.399 0.412

CTH NOS3 9606.ENSP00000359976 9606.ENSP00000297494 0.047 0 0 0 0.084 0 0 0.64 0.658

CTH MSRA 9606.ENSP00000359976 9606.ENSP00000313921 0.282 0 0 0 0.185 0.13 0 0.617 0.779

CTH GCLM 9606.ENSP00000359976 9606.ENSP00000359258 0.042 0 0 0 0.064 0 0.8 0.347 0.867

CTH NOS1 9606.ENSP00000359976 9606.ENSP00000477999 0.047 0 0 0 0.084 0 0 0.433 0.461

CTH TXN 9606.ENSP00000359976 9606.ENSP00000363641 0 0 0 0 0.062 0.06 0 0.464 0.486

CTH HMOX2 9606.ENSP00000359976 9606.ENSP00000477572 0 0 0 0 0 0 0 0.574 0.574

CTH SUOX 9606.ENSP00000359976 9606.ENSP00000377668 0 0 0 0 0.064 0 0 0.639 0.648

CTH MTHFR 9606.ENSP00000359976 9606.ENSP00000365777 0.295 0 0 0 0.145 0.143 0 0.818 0.893

CTH TXNRD2 9606.ENSP00000359976 9606.ENSP00000383365 0 0 0 0 0.062 0 0.9 0.188 0.917

CTH TXNRD1 9606.ENSP00000359976 9606.ENSP00000434516 0.076 0 0 0 0.065 0 0.9 0.41 0.942

CYBA NOX3 9606.ENSP00000261623 9606.ENSP00000159060 0 0 0 0 0.084 0.139 0.8 0.979 0.996

CYBA HMOX1 9606.ENSP00000261623 9606.ENSP00000216117 0 0 0 0 0.091 0 0 0.468 0.495

CYBA MPO 9606.ENSP00000261623 9606.ENSP00000225275 0 0 0 0 0 0 0 0.512 0.512

CYBA MAPK14 9606.ENSP00000261623 9606.ENSP00000229795 0 0 0 0 0 0 0.65 0.335 0.757

CYBA CAT 9606.ENSP00000261623 9606.ENSP00000241052 0 0 0 0 0 0 0 0.658 0.658

CYBA NOS1 9606.ENSP00000261623 9606.ENSP00000477999 0 0 0 0 0 0 0 0.407 0.407

CYBA GPX4 9606.ENSP00000261623 9606.ENSP00000346103 0 0 0 0 0 0 0 0.427 0.427

CYBA NQO1 9606.ENSP00000261623 9606.ENSP00000319788 0 0 0 0 0 0 0 0.443 0.443

CYBA SOD1 9606.ENSP00000261623 9606.ENSP00000270142 0 0 0 0 0 0 0 0.444 0.444

CYBA PRDX6 9606.ENSP00000261623 9606.ENSP00000342026 0 0 0 0 0 0 0 0.444 0.444

CYBA SOD2 9606.ENSP00000261623 9606.ENSP00000446252 0 0 0 0 0 0 0 0.45 0.45

CYBA TXN 9606.ENSP00000261623 9606.ENSP00000363641 0 0 0 0 0.064 0 0 0.449 0.462

CYBA SOD3 9606.ENSP00000261623 9606.ENSP00000371554 0 0 0 0 0 0 0 0.508 0.508

CYBA GPX1 9606.ENSP00000261623 9606.ENSP00000407375 0 0 0 0 0.096 0 0 0.506 0.535

CYBA S100A8 9606.ENSP00000261623 9606.ENSP00000357722 0 0 0 0 0.087 0 0.6 0.042 0.619

CYBA S100A9 9606.ENSP00000261623 9606.ENSP00000357727 0 0 0 0 0.087 0 0.6 0.072 0.632

CYBA NOS3 9606.ENSP00000261623 9606.ENSP00000297494 0 0 0 0 0 0 0 0.681 0.681

CYBA MMP9 9606.ENSP00000261623 9606.ENSP00000361405 0 0 0 0 0.108 0 0.65 0.342 0.776

CYBA DUOX2 9606.ENSP00000261623 9606.ENSP00000475084 0 0 0 0 0.063 0.106 0.54 0.762 0.896

CYBA DUOX1 9606.ENSP00000261623 9606.ENSP00000317997 0 0 0 0 0.063 0.106 0.54 0.951 0.978

CYBA NOX5 9606.ENSP00000261623 9606.ENSP00000373518 0 0 0 0 0.062 0.078 0.54 0.974 0.988

CYBA NOX1 9606.ENSP00000261623 9606.ENSP00000362057 0 0 0 0 0.083 0.292 0.8 0.99 0.998

CYBA NOX4 9606.ENSP00000261623 9606.ENSP00000263317 0 0 0 0 0.062 0.078 0.8 0.991 0.998

CYBA CYBB 9606.ENSP00000261623 9606.ENSP00000367851 0 0 0 0 0.143 0.302 0.9 0.993 0.999

CYBA NCF2 9606.ENSP00000261623 9606.ENSP00000356505 0 0 0 0 0.156 0.462 0.9 0.993 0.999

CYBB NOX3 9606.ENSP00000367851 9606.ENSP00000159060 0 0 0 0.963 0 0 0.54 0.963 0.556

CYBB HMOX1 9606.ENSP00000367851 9606.ENSP00000216117 0 0 0 0 0.069 0 0 0.677 0.687

CYBB GSR 9606.ENSP00000367851 9606.ENSP00000221130 0 0 0 0 0 0 0 0.481 0.481

CYBB MPO 9606.ENSP00000367851 9606.ENSP00000225275 0 0 0 0 0.143 0.062 0 0.717 0.753

CYBB MAPK14 9606.ENSP00000367851 9606.ENSP00000229795 0 0 0 0 0 0.078 0.65 0.43 0.8

CYBB CAT 9606.ENSP00000367851 9606.ENSP00000241052 0 0 0 0 0.067 0 0 0.79 0.795

CYBB CYBA 9606.ENSP00000367851 9606.ENSP00000261623 0 0 0 0 0.143 0.302 0.9 0.993 0.999

CYBB NOX4 9606.ENSP00000367851 9606.ENSP00000263317 0 0 0 0.874 0 0.391 0.8 0.923 0.887

CYBB SOD1 9606.ENSP00000367851 9606.ENSP00000270142 0 0 0 0 0 0.179 0 0.658 0.707

CYBB NOS3 9606.ENSP00000367851 9606.ENSP00000297494 0 0 0 0 0.062 0 0 0.761 0.767

CYBB UCP2 9606.ENSP00000367851 9606.ENSP00000312029 0 0 0 0 0.096 0 0 0.374 0.41

CYBB DUOX1 9606.ENSP00000367851 9606.ENSP00000317997 0 0 0 0.736 0 0 0.54 0.89 0.647

CYBB NQO1 9606.ENSP00000367851 9606.ENSP00000319788 0 0 0 0 0 0 0 0.462 0.462

CYBB NOS2 9606.ENSP00000367851 9606.ENSP00000327251 0 0 0 0 0.062 0 0 0.57 0.58

CYBB PRDX6 9606.ENSP00000367851 9606.ENSP00000342026 0 0 0 0 0 0 0 0.466 0.466

CYBB GPX4 9606.ENSP00000367851 9606.ENSP00000346103 0 0 0 0 0 0 0 0.514 0.514

CYBB NCF2 9606.ENSP00000367851 9606.ENSP00000356505 0 0 0 0 0.382 0.462 0.9 0.992 0.999

CYBB S100A8 9606.ENSP00000367851 9606.ENSP00000357722 0 0 0 0 0.347 0 0.6 0.15 0.758

CYBB S100A9 9606.ENSP00000367851 9606.ENSP00000357727 0 0 0 0 0.351 0 0.6 0.154 0.761

CYBB MMP9 9606.ENSP00000367851 9606.ENSP00000361405 0 0 0 0 0.17 0 0.65 0.559 0.86

CYBB NOX1 9606.ENSP00000367851 9606.ENSP00000362057 0 0 0 0.959 0 0 0.8 0.99 0.808

CYBB TXN 9606.ENSP00000367851 9606.ENSP00000363641 0 0 0 0 0.062 0.057 0 0.634 0.648

CYBB HMOX2 9606.ENSP00000367851 9606.ENSP00000477572 0 0 0 0 0 0 0 0.417 0.417

CYBB GPX3 9606.ENSP00000367851 9606.ENSP00000373477 0 0 0 0 0 0 0 0.437 0.437

CYBB NFE2L2 9606.ENSP00000367851 9606.ENSP00000380252 0 0 0 0 0 0 0 0.533 0.533

CYBB NOS1 9606.ENSP00000367851 9606.ENSP00000477999 0 0 0 0 0.062 0 0 0.568 0.577

CYBB SOD2 9606.ENSP00000367851 9606.ENSP00000446252 0 0 0 0 0.093 0 0 0.601 0.623

CYBB GPX1 9606.ENSP00000367851 9606.ENSP00000407375 0 0 0 0 0.062 0 0 0.623 0.631

CYBB DUOX2 9606.ENSP00000367851 9606.ENSP00000475084 0 0 0 0.743 0 0 0.54 0.826 0.636

CYBB SOD3 9606.ENSP00000367851 9606.ENSP00000371554 0 0 0 0 0 0.104 0 0.62 0.645

CYBB NOX5 9606.ENSP00000367851 9606.ENSP00000373518 0 0 0 0.731 0 0 0.54 0.908 0.651

CYP1A1 HMOX1 9606.ENSP00000369050 9606.ENSP00000216117 0 0 0 0 0.066 0 0 0.567 0.578

CYP1A1 GSR 9606.ENSP00000369050 9606.ENSP00000221130 0 0 0 0 0.062 0 0 0.402 0.415

CYP1A1 PON1 9606.ENSP00000369050 9606.ENSP00000222381 0 0 0 0 0.054 0 0 0.451 0.458

CYP1A1 CAT 9606.ENSP00000369050 9606.ENSP00000241052 0.047 0 0 0 0.062 0 0 0.492 0.507

CYP1A1 GSTM3 9606.ENSP00000369050 9606.ENSP00000256594 0 0 0 0 0 0.057 0.65 0.561 0.842

CYP1A1 GSTT2B 9606.ENSP00000369050 9606.ENSP00000290765 0 0 0 0 0.062 0 0.65 0.416 0.791

CYP1A1 GSTM1 9606.ENSP00000369050 9606.ENSP00000311469 0 0 0 0 0.062 0.057 0.65 0.87 0.954

CYP1A1 AOC3 9606.ENSP00000369050 9606.ENSP00000312326 0 0 0 0 0.063 0 0 0.412 0.425

CYP1A1 NQO1 9606.ENSP00000369050 9606.ENSP00000319788 0 0 0 0 0.085 0 0 0.749 0.761

CYP1A1 GSTA1 9606.ENSP00000369050 9606.ENSP00000335620 0 0 0 0 0.062 0.057 0.65 0.613 0.864

CYP1A1 GPX4 9606.ENSP00000369050 9606.ENSP00000346103 0 0 0 0 0 0 0 0.606 0.606

CYP1A1 AOX1 9606.ENSP00000369050 9606.ENSP00000363832 0 0 0 0 0.065 0 0.9 0.277 0.926

CYP1A1 MTHFR 9606.ENSP00000369050 9606.ENSP00000365777 0 0 0 0 0.064 0 0 0.523 0.534

CYP1A1 GPX1 9606.ENSP00000369050 9606.ENSP00000407375 0 0 0 0 0 0 0 0.458 0.458

CYP1A1 NFE2L2 9606.ENSP00000369050 9606.ENSP00000380252 0 0 0 0 0 0 0 0.52 0.52

CYP1A1 MGST1 9606.ENSP00000369050 9606.ENSP00000379512 0 0 0 0 0.062 0 0.65 0.281 0.743

CYP1A1 GSTP1 9606.ENSP00000369050 9606.ENSP00000381607 0 0 0 0 0 0.057 0.65 0.763 0.915

DAO MPO 9606.ENSP00000228476 9606.ENSP00000225275 0 0 0 0 0.072 0.236 0 0.463 0.586

DAO TXNRD1 9606.ENSP00000228476 9606.ENSP00000434516 0.185 0 0 0 0.062 0.175 0 0.174 0.409

DAO DRD3 9606.ENSP00000228476 9606.ENSP00000373169 0 0 0 0 0 0 0 0.412 0.412

DAO MTHFR 9606.ENSP00000228476 9606.ENSP00000365777 0.049 0 0 0 0 0 0 0.415 0.42

DAO SUOX 9606.ENSP00000228476 9606.ENSP00000377668 0 0 0 0 0.062 0 0 0.422 0.434

DAO AOC2 9606.ENSP00000228476 9606.ENSP00000253799 0 0 0 0 0.063 0 0 0.432 0.445

DAO DRD1 9606.ENSP00000228476 9606.ENSP00000377353 0 0 0 0 0.139 0 0 0.428 0.487

DAO IL4I1 9606.ENSP00000228476 9606.ENSP00000472474 0.07 0 0 0 0.062 0 0 0.55 0.573

DAO AOC1 9606.ENSP00000228476 9606.ENSP00000411613 0 0 0 0 0.063 0.213 0 0.496 0.596

DAO CTH 9606.ENSP00000228476 9606.ENSP00000359976 0.055 0 0 0 0.105 0 0 0.647 0.675

DAO NOS2 9606.ENSP00000228476 9606.ENSP00000327251 0 0 0 0 0 0 0.9 0.131 0.909

DAO DDO 9606.ENSP00000228476 9606.ENSP00000357920 0 0 0.418 0.88 0.062 0 0.9 0.905 0.916

DAO ACOX3 9606.ENSP00000228476 9606.ENSP00000348775 0 0 0 0 0.049 0 0.9 0.189 0.916

DAO ACOX1 9606.ENSP00000228476 9606.ENSP00000293217 0 0 0 0 0.062 0 0.9 0.373 0.936

DAO HAO2 9606.ENSP00000228476 9606.ENSP00000483507 0 0 0 0 0.167 0 0.9 0.45 0.95

DAO CAT 9606.ENSP00000228476 9606.ENSP00000241052 0.088 0 0 0 0.062 0 0.9 0.512 0.952

DAO PAOX 9606.ENSP00000228476 9606.ENSP00000278060 0 0 0 0 0.152 0 0.9 0.499 0.953

DAO HAO1 9606.ENSP00000228476 9606.ENSP00000368066 0 0 0 0 0.14 0 0.9 0.505 0.953

DAO PIPOX 9606.ENSP00000228476 9606.ENSP00000317721 0 0 0 0 0.065 0 0.9 0.563 0.955

DDO DAO 9606.ENSP00000357920 9606.ENSP00000228476 0 0 0.418 0.88 0.062 0 0.9 0.905 0.916

DDO CAT 9606.ENSP00000357920 9606.ENSP00000241052 0.088 0 0 0 0.062 0 0.9 0.29 0.931

DDO GFER 9606.ENSP00000357920 9606.ENSP00000248114 0 0 0 0 0 0 0 0.423 0.423

DDO PAOX 9606.ENSP00000357920 9606.ENSP00000278060 0 0 0 0 0.152 0 0.9 0.515 0.955

DDO ACOX1 9606.ENSP00000357920 9606.ENSP00000293217 0 0 0 0 0.062 0 0.9 0.252 0.923

DDO PIPOX 9606.ENSP00000357920 9606.ENSP00000317721 0 0 0 0 0.065 0 0.9 0.605 0.959

DDO NOS2 9606.ENSP00000357920 9606.ENSP00000327251 0 0 0 0 0 0 0.9 0.1 0.906

DDO ACOX3 9606.ENSP00000357920 9606.ENSP00000348775 0 0 0 0 0.052 0 0.9 0.101 0.907

DDO TXNRD2 9606.ENSP00000357920 9606.ENSP00000383365 0.072 0 0 0 0.062 0.194 0 0.297 0.441

DDO IL4I1 9606.ENSP00000357920 9606.ENSP00000472474 0.07 0 0 0 0.062 0 0 0.466 0.493

DDO HAO1 9606.ENSP00000357920 9606.ENSP00000368066 0 0 0 0 0.108 0 0.9 0.276 0.929

DDO HAO2 9606.ENSP00000357920 9606.ENSP00000483507 0 0 0 0 0.108 0 0.9 0.348 0.936

DGKK MTHFR 9606.ENSP00000477515 9606.ENSP00000365777 0 0 0 0 0 0 0 0.66 0.66

DHCR24 SUOX 9606.ENSP00000360316 9606.ENSP00000377668 0 0 0 0 0 0.709 0 0.044 0.709

DRD1 DAO 9606.ENSP00000377353 9606.ENSP00000228476 0 0 0 0 0.139 0 0 0.428 0.487

DRD1 FOS 9606.ENSP00000377353 9606.ENSP00000306245 0 0 0 0 0 0 0 0.604 0.604

DRD1 MAOA 9606.ENSP00000377353 9606.ENSP00000340684 0 0 0 0 0 0 0 0.614 0.614

DRD1 DRD2 9606.ENSP00000377353 9606.ENSP00000354859 0 0 0 0.727 0.116 0.176 0 0.965 0.44

DRD1 MAOB 9606.ENSP00000377353 9606.ENSP00000367309 0 0 0 0 0.123 0 0 0.525 0.566

DRD1 DRD3 9606.ENSP00000377353 9606.ENSP00000373169 0 0 0 0.758 0 0.312 0 0.895 0.46

DRD2 DRD4 9606.ENSP00000354859 9606.ENSP00000176183 0 0 0 0.855 0 0.312 0 0.962 0.407

DRD2 DRD5 9606.ENSP00000354859 9606.ENSP00000306129 0 0 0 0.713 0.09 0.159 0 0.944 0.417

DRD2 FOS 9606.ENSP00000354859 9606.ENSP00000306245 0 0 0 0 0 0 0 0.607 0.607

DRD2 MAOA 9606.ENSP00000354859 9606.ENSP00000340684 0 0 0 0 0 0 0 0.756 0.757

DRD2 DRD1 9606.ENSP00000354859 9606.ENSP00000377353 0 0 0 0.727 0.116 0.176 0 0.965 0.44

DRD2 MTHFR 9606.ENSP00000354859 9606.ENSP00000365777 0 0 0 0 0 0 0 0.455 0.455

DRD2 MAOB 9606.ENSP00000354859 9606.ENSP00000367309 0 0 0 0 0.063 0 0 0.611 0.62

DRD3 DRD4 9606.ENSP00000373169 9606.ENSP00000176183 0 0 0 0.869 0 0 0.8 0.888 0.823

DRD3 DAO 9606.ENSP00000373169 9606.ENSP00000228476 0 0 0 0 0 0 0 0.412 0.412

DRD3 FOS 9606.ENSP00000373169 9606.ENSP00000306245 0 0 0 0 0 0 0 0.408 0.408

DRD3 MAOA 9606.ENSP00000373169 9606.ENSP00000340684 0 0 0 0 0 0 0 0.665 0.665

DRD3 MAOB 9606.ENSP00000373169 9606.ENSP00000367309 0 0 0 0 0 0 0 0.56 0.56

DRD3 GSTP1 9606.ENSP00000373169 9606.ENSP00000381607 0 0 0 0 0 0.063 0 0.426 0.439

DRD3 DRD1 9606.ENSP00000373169 9606.ENSP00000377353 0 0 0 0.758 0 0.312 0 0.895 0.46

DRD4 DRD2 9606.ENSP00000176183 9606.ENSP00000354859 0 0 0 0.855 0 0.312 0 0.962 0.407

DRD4 MTHFR 9606.ENSP00000176183 9606.ENSP00000365777 0 0 0 0 0 0 0 0.45 0.45

DRD4 MAOB 9606.ENSP00000176183 9606.ENSP00000367309 0 0 0 0 0 0 0 0.575 0.575

DRD4 DRD3 9606.ENSP00000176183 9606.ENSP00000373169 0 0 0 0.869 0 0 0.8 0.888 0.823

DRD4 MAOA 9606.ENSP00000176183 9606.ENSP00000340684 0 0 0 0 0 0 0 0.831 0.831

DRD5 DRD2 9606.ENSP00000306129 9606.ENSP00000354859 0 0 0 0.713 0.09 0.159 0 0.944 0.417

DRD5 MAOB 9606.ENSP00000306129 9606.ENSP00000367309 0 0 0 0 0.067 0 0 0.504 0.517

DRD5 MAOA 9606.ENSP00000306129 9606.ENSP00000340684 0 0 0 0 0 0 0 0.61 0.61

DUOX1 NOX3 9606.ENSP00000317997 9606.ENSP00000159060 0 0 0 0.754 0 0 0.54 0.883 0.639

DUOX1 HMOX1 9606.ENSP00000317997 9606.ENSP00000216117 0 0 0 0 0 0 0 0.5 0.5

DUOX1 GSR 9606.ENSP00000317997 9606.ENSP00000221130 0 0 0 0 0 0 0 0.442 0.442

DUOX1 CAT 9606.ENSP00000317997 9606.ENSP00000241052 0 0 0 0 0 0 0 0.749 0.749

DUOX1 CYBA 9606.ENSP00000317997 9606.ENSP00000261623 0 0 0 0 0.063 0.106 0.54 0.951 0.978

DUOX1 NOX4 9606.ENSP00000317997 9606.ENSP00000263317 0 0 0 0.709 0 0.244 0.54 0.914 0.733

DUOX1 SOD1 9606.ENSP00000317997 9606.ENSP00000270142 0 0 0 0 0 0.141 0 0.465 0.521

DUOX1 NOS3 9606.ENSP00000317997 9606.ENSP00000297494 0 0 0 0 0.062 0 0 0.618 0.626

DUOX1 NOS1 9606.ENSP00000317997 9606.ENSP00000477999 0 0 0 0 0.082 0 0 0.383 0.41

DUOX1 GPX3 9606.ENSP00000317997 9606.ENSP00000373477 0 0 0 0 0 0 0 0.417 0.417

DUOX1 SOD2 9606.ENSP00000317997 9606.ENSP00000446252 0 0 0 0 0.065 0 0 0.424 0.438

DUOX1 GPX1 9606.ENSP00000317997 9606.ENSP00000407375 0 0 0 0 0 0 0 0.465 0.465

DUOX1 NFE2L2 9606.ENSP00000317997 9606.ENSP00000380252 0 0 0 0 0 0 0 0.467 0.467

DUOX1 SOD3 9606.ENSP00000317997 9606.ENSP00000371554 0 0 0 0 0 0.104 0 0.431 0.469

DUOX1 GPX4 9606.ENSP00000317997 9606.ENSP00000346103 0 0 0 0 0 0 0 0.538 0.538

DUOX1 TXN 9606.ENSP00000317997 9606.ENSP00000363641 0 0 0 0 0.062 0.057 0 0.557 0.575

DUOX1 NOX1 9606.ENSP00000317997 9606.ENSP00000362057 0 0 0 0.756 0 0 0.54 0.944 0.645

DUOX1 CYBB 9606.ENSP00000317997 9606.ENSP00000367851 0 0 0 0.736 0 0 0.54 0.89 0.647

DUOX1 NOX5 9606.ENSP00000317997 9606.ENSP00000373518 0 0 0 0.706 0 0 0.54 0.975 0.671

DUOX1 DUOX2 9606.ENSP00000317997 9606.ENSP00000475084 0 0 0 0.979 0.082 0 0.8 0.967 0.812

DUOX1 NCF2 9606.ENSP00000317997 9606.ENSP00000356505 0 0 0 0 0.065 0 0.54 0.873 0.94

DUOX2 NOX3 9606.ENSP00000475084 9606.ENSP00000159060 0 0 0 0.751 0 0 0.54 0.82 0.633

DUOX2 HMOX1 9606.ENSP00000475084 9606.ENSP00000216117 0 0 0 0 0 0 0 0.489 0.489

DUOX2 CAT 9606.ENSP00000475084 9606.ENSP00000241052 0 0 0 0 0 0 0 0.706 0.706

DUOX2 CYBA 9606.ENSP00000475084 9606.ENSP00000261623 0 0 0 0 0.063 0.106 0.54 0.762 0.896

DUOX2 NOX4 9606.ENSP00000475084 9606.ENSP00000263317 0 0 0 0.7 0 0.244 0.54 0.863 0.73

DUOX2 SOD1 9606.ENSP00000475084 9606.ENSP00000270142 0 0 0 0 0 0.141 0 0.404 0.467

DUOX2 NOS3 9606.ENSP00000475084 9606.ENSP00000297494 0 0 0 0 0.062 0 0 0.609 0.618

DUOX2 DUOX1 9606.ENSP00000475084 9606.ENSP00000317997 0 0 0 0.979 0.082 0 0.8 0.967 0.812

DUOX2 GPX4 9606.ENSP00000475084 9606.ENSP00000346103 0 0 0 0 0 0 0 0.48 0.48

DUOX2 NCF2 9606.ENSP00000475084 9606.ENSP00000356505 0 0 0 0 0.065 0 0.54 0.713 0.866

DUOX2 NOX1 9606.ENSP00000475084 9606.ENSP00000362057 0 0 0 0.744 0 0 0.54 0.857 0.64

DUOX2 TXN 9606.ENSP00000475084 9606.ENSP00000363641 0 0 0 0 0.062 0.057 0 0.387 0.41

DUOX2 CYBB 9606.ENSP00000475084 9606.ENSP00000367851 0 0 0 0.743 0 0 0.54 0.826 0.636

DUOX2 SOD3 9606.ENSP00000475084 9606.ENSP00000371554 0 0 0 0 0 0.104 0 0.361 0.403

DUOX2 NOX5 9606.ENSP00000475084 9606.ENSP00000373518 0 0 0 0.7 0 0 0.54 0.908 0.664

DUOX2 NFE2L2 9606.ENSP00000475084 9606.ENSP00000380252 0 0 0 0 0 0 0 0.411 0.411

DUOX2 GPX1 9606.ENSP00000475084 9606.ENSP00000407375 0 0 0 0 0 0 0 0.454 0.454

DUSP1 MAPK14 9606.ENSP00000239223 9606.ENSP00000229795 0 0 0 0 0 0.9 0.9 0.887 0.998

DUSP1 NFE2L2 9606.ENSP00000239223 9606.ENSP00000380252 0 0 0 0 0 0.27 0 0.218 0.405

DUSP1 JUNB 9606.ENSP00000239223 9606.ENSP00000303315 0 0 0 0 0.658 0.145 0 0.299 0.777

DUSP1 MAPK10 9606.ENSP00000239223 9606.ENSP00000352157 0 0 0 0 0.062 0.356 0.8 0.374 0.914

DUSP1 FOS 9606.ENSP00000239223 9606.ENSP00000306245 0 0 0 0 0.872 0.139 0 0.686 0.962

ERCC1 CDKN2A 9606.ENSP00000013807 9606.ENSP00000418915 0 0 0 0 0 0 0 0.401 0.4

ERCC1 GSTM1 9606.ENSP00000013807 9606.ENSP00000311469 0 0 0 0 0 0 0 0.519 0.519

ERCC1 MTHFR 9606.ENSP00000013807 9606.ENSP00000365777 0 0 0 0 0 0 0 0.52 0.52

ERCC1 APTX 9606.ENSP00000013807 9606.ENSP00000400806 0 0 0 0 0.062 0.121 0 0.555 0.601

ERCC1 GSTP1 9606.ENSP00000013807 9606.ENSP00000381607 0 0 0 0 0.062 0 0 0.616 0.624

ERCC1 PNKP 9606.ENSP00000013807 9606.ENSP00000323511 0 0 0 0 0.12 0.144 0 0.679 0.737

ERCC1 ERCC6 9606.ENSP00000013807 9606.ENSP00000348089 0 0 0 0 0 0.058 0 0.812 0.815

ERCC1 ERCC3 9606.ENSP00000013807 9606.ENSP00000285398 0 0 0 0 0.062 0 0.6 0.875 0.949

ERCC1 ERCC8 9606.ENSP00000013807 9606.ENSP00000265038 0 0 0 0 0 0 0.9 0.71 0.969

ERCC1 ERCC2 9606.ENSP00000013807 9606.ENSP00000375809 0 0 0 0 0.062 0 0.6 0.949 0.979

ERCC2 ERCC1 9606.ENSP00000375809 9606.ENSP00000013807 0 0 0 0 0.062 0 0.6 0.949 0.979

ERCC2 ERCC8 9606.ENSP00000375809 9606.ENSP00000265038 0 0 0 0 0.062 0 0.6 0.754 0.899

ERCC2 ERCC3 9606.ENSP00000375809 9606.ENSP00000285398 0.051 0 0 0 0.117 0.998 0.9 0.98 0.999

ERCC2 GSTM1 9606.ENSP00000375809 9606.ENSP00000311469 0 0 0 0 0 0 0 0.601 0.601

ERCC2 PNKP 9606.ENSP00000375809 9606.ENSP00000323511 0 0 0 0 0.107 0 0 0.439 0.478

ERCC2 ERCC6 9606.ENSP00000375809 9606.ENSP00000348089 0 0 0 0 0.065 0.296 0 0.839 0.885

ERCC2 MTHFR 9606.ENSP00000375809 9606.ENSP00000365777 0.069 0 0 0 0.062 0 0 0.519 0.543

ERCC2 APTX 9606.ENSP00000375809 9606.ENSP00000400806 0 0 0 0 0.073 0 0 0.501 0.518

ERCC2 GSTP1 9606.ENSP00000375809 9606.ENSP00000381607 0 0 0 0 0 0 0 0.609 0.609

ERCC3 ERCC1 9606.ENSP00000285398 9606.ENSP00000013807 0 0 0 0 0.062 0 0.6 0.875 0.949

ERCC3 ERCC8 9606.ENSP00000285398 9606.ENSP00000265038 0 0 0 0 0.063 0 0.6 0.756 0.9

ERCC3 APTX 9606.ENSP00000285398 9606.ENSP00000400806 0 0 0 0 0.06 0 0 0.482 0.492

ERCC3 ERCC6 9606.ENSP00000285398 9606.ENSP00000348089 0.068 0 0 0 0.063 0.346 0 0.799 0.87

ERCC3 ERCC2 9606.ENSP00000285398 9606.ENSP00000375809 0.051 0 0 0 0.117 0.998 0.9 0.98 0.999

ERCC6 ERCC1 9606.ENSP00000348089 9606.ENSP00000013807 0 0 0 0 0 0.058 0 0.812 0.815

ERCC6 ERCC8 9606.ENSP00000348089 9606.ENSP00000265038 0 0 0 0 0.054 0.87 0 0.914 0.988

ERCC6 ERCC3 9606.ENSP00000348089 9606.ENSP00000285398 0.068 0 0 0 0.063 0.346 0 0.799 0.87

ERCC6 APTX 9606.ENSP00000348089 9606.ENSP00000400806 0 0 0 0 0.062 0.058 0 0.476 0.497

ERCC6 ERCC2 9606.ENSP00000348089 9606.ENSP00000375809 0 0 0 0 0.065 0.296 0 0.839 0.885

ERCC8 ERCC1 9606.ENSP00000265038 9606.ENSP00000013807 0 0 0 0 0 0 0.9 0.71 0.969

ERCC8 ERCC2 9606.ENSP00000265038 9606.ENSP00000375809 0 0 0 0 0.062 0 0.6 0.754 0.899

ERCC8 ERCC3 9606.ENSP00000265038 9606.ENSP00000285398 0 0 0 0 0.063 0 0.6 0.756 0.9

ERCC8 ERCC6 9606.ENSP00000265038 9606.ENSP00000348089 0 0 0 0 0.054 0.87 0 0.914 0.988

FOS HMOX1 9606.ENSP00000306245 9606.ENSP00000216117 0 0 0 0 0 0 0.9 0.517 0.949

FOS MPO 9606.ENSP00000306245 9606.ENSP00000225275 0 0 0 0 0 0 0 0.414 0.414

FOS NFKB1 9606.ENSP00000306245 9606.ENSP00000226574 0 0 0 0 0.049 0.225 0 0.516 0.612

FOS MAPK14 9606.ENSP00000306245 9606.ENSP00000229795 0 0 0 0 0 0.248 0.9 0.592 0.966

FOS LOX 9606.ENSP00000306245 9606.ENSP00000231004 0 0 0 0 0 0 0 0.406 0.406

FOS DUSP1 9606.ENSP00000306245 9606.ENSP00000239223 0 0 0 0 0.872 0.139 0 0.686 0.962

FOS CAT 9606.ENSP00000306245 9606.ENSP00000241052 0 0 0 0 0.062 0 0 0.528 0.539

FOS MMP13 9606.ENSP00000306245 9606.ENSP00000260302 0 0 0 0 0 0 0 0.468 0.468

FOS NOS3 9606.ENSP00000306245 9606.ENSP00000297494 0 0 0 0 0 0 0 0.467 0.467

FOS MMP3 9606.ENSP00000306245 9606.ENSP00000299855 0 0 0 0 0 0 0 0.473 0.473

FOS JUNB 9606.ENSP00000306245 9606.ENSP00000303315 0 0 0 0 0.826 0.899 0.9 0.986 0.999

FOS DRD3 9606.ENSP00000306245 9606.ENSP00000373169 0 0 0 0 0 0 0 0.408 0.408

FOS CDKN2A 9606.ENSP00000306245 9606.ENSP00000418915 0 0 0 0 0 0.056 0 0.517 0.525

FOS NQO1 9606.ENSP00000306245 9606.ENSP00000319788 0 0 0 0 0 0.27 0 0.391 0.536

FOS NOS1 9606.ENSP00000306245 9606.ENSP00000477999 0 0 0 0 0 0 0 0.54 0.54

FOS DRD1 9606.ENSP00000306245 9606.ENSP00000377353 0 0 0 0 0 0 0 0.604 0.604

FOS DRD2 9606.ENSP00000306245 9606.ENSP00000354859 0 0 0 0 0 0 0 0.607 0.607

FOS MMP9 9606.ENSP00000306245 9606.ENSP00000361405 0 0 0 0 0.062 0 0 0.656 0.664

FOS MMP1 9606.ENSP00000306245 9606.ENSP00000322788 0 0 0 0 0 0.27 0 0.561 0.666

FOS MAPK10 9606.ENSP00000306245 9606.ENSP00000352157 0 0 0 0 0 0.243 0.8 0.479 0.914

FOS HMOX2 9606.ENSP00000306245 9606.ENSP00000477572 0 0 0 0 0 0 0.9 0.185 0.915

FOS NFE2L2 9606.ENSP00000306245 9606.ENSP00000380252 0 0 0 0 0 0.056 0.9 0.468 0.945

FOS SP1 9606.ENSP00000306245 9606.ENSP00000329357 0 0 0 0 0 0.062 0.9 0.501 0.949

FOS RUNX2 9606.ENSP00000306245 9606.ENSP00000360493 0 0 0 0 0 0.486 0.8 0.898 0.988

GCLC HMOX1 9606.ENSP00000229416 9606.ENSP00000216117 0 0 0 0 0 0.104 0 0.889 0.896

GCLC TXN2 9606.ENSP00000229416 9606.ENSP00000216185 0 0 0 0 0.062 0.06 0 0.376 0.402

GCLC GSS 9606.ENSP00000229416 9606.ENSP00000216951 0 0 0 0 0.108 0.348 0.9 0.847 0.989

GCLC GSR 9606.ENSP00000229416 9606.ENSP00000221130 0 0 0 0 0.184 0 0 0.911 0.924

GCLC NOX4 9606.ENSP00000229416 9606.ENSP00000263317 0 0 0 0 0.062 0 0 0.399 0.413

GCLC PRDX6 9606.ENSP00000229416 9606.ENSP00000342026 0 0 0 0 0 0 0 0.416 0.416

GCLC MGST1 9606.ENSP00000229416 9606.ENSP00000379512 0 0 0 0 0.062 0 0 0.414 0.427

GCLC PRDX5 9606.ENSP00000229416 9606.ENSP00000265462 0 0 0 0 0 0 0 0.431 0.431

GCLC PRDX2 9606.ENSP00000229416 9606.ENSP00000301522 0 0 0 0 0.053 0 0 0.51 0.516

GCLC GSTT2B 9606.ENSP00000229416 9606.ENSP00000290765 0 0 0 0 0.082 0.152 0 0.431 0.518

GCLC GSTM3 9606.ENSP00000229416 9606.ENSP00000256594 0 0 0 0 0 0 0 0.554 0.554

GCLC GPX3 9606.ENSP00000229416 9606.ENSP00000373477 0 0 0 0 0.062 0 0 0.558 0.568

GCLC GSTM1 9606.ENSP00000229416 9606.ENSP00000311469 0 0 0 0 0 0 0 0.605 0.605

GCLC SOD3 9606.ENSP00000229416 9606.ENSP00000371554 0 0 0 0 0.052 0.271 0 0.521 0.64

GCLC TXNRD2 9606.ENSP00000229416 9606.ENSP00000383365 0 0 0 0 0.112 0 0 0.616 0.645

GCLC GSTA1 9606.ENSP00000229416 9606.ENSP00000335620 0 0 0 0 0.052 0 0 0.641 0.646

GCLC HMOX2 9606.ENSP00000229416 9606.ENSP00000477572 0 0 0 0 0 0.104 0 0.645 0.668

GCLC GLRX2 9606.ENSP00000229416 9606.ENSP00000356410 0 0 0 0 0.123 0 0 0.689 0.716

GCLC GSTP1 9606.ENSP00000229416 9606.ENSP00000381607 0 0 0 0 0 0 0 0.732 0.732

GCLC SRXN1 9606.ENSP00000229416 9606.ENSP00000371388 0 0 0 0 0.233 0 0 0.7 0.76

GCLC GPX1 9606.ENSP00000229416 9606.ENSP00000407375 0 0 0 0 0.062 0 0 0.765 0.77

GCLC GPX4 9606.ENSP00000229416 9606.ENSP00000346103 0 0 0 0 0.062 0 0 0.78 0.785

GCLC TXN 9606.ENSP00000229416 9606.ENSP00000363641 0 0 0 0 0.088 0.103 0 0.825 0.845

GCLC SOD1 9606.ENSP00000229416 9606.ENSP00000270142 0 0 0 0 0.062 0.348 0 0.776 0.851

GCLC NFE2L2 9606.ENSP00000229416 9606.ENSP00000380252 0 0 0 0 0 0 0 0.859 0.859

GCLC NQO1 9606.ENSP00000229416 9606.ENSP00000319788 0 0 0 0 0.063 0 0 0.892 0.895

GCLC CAT 9606.ENSP00000229416 9606.ENSP00000241052 0 0 0 0 0.143 0.26 0 0.859 0.903

GCLC TXNRD1 9606.ENSP00000229416 9606.ENSP00000434516 0 0 0 0 0.186 0 0 0.894 0.91

GCLC SOD2 9606.ENSP00000229416 9606.ENSP00000446252 0 0 0 0 0.067 0.731 0 0.816 0.949

GCLC CTH 9606.ENSP00000229416 9606.ENSP00000359976 0 0 0 0 0.161 0.727 0.8 0.627 0.98

GCLC GCLM 9606.ENSP00000229416 9606.ENSP00000359258 0 0 0 0 0.202 0.921 0.9 0.995 0.999

GCLM HMOX1 9606.ENSP00000359258 9606.ENSP00000216117 0 0 0 0 0.053 0 0 0.855 0.857

GCLM TXN2 9606.ENSP00000359258 9606.ENSP00000216185 0 0 0 0 0.063 0.056 0 0.401 0.424

GCLM GSS 9606.ENSP00000359258 9606.ENSP00000216951 0 0 0 0 0.067 0.223 0.9 0.75 0.979

GCLM GSR 9606.ENSP00000359258 9606.ENSP00000221130 0 0 0 0 0.064 0.057 0 0.809 0.816

GCLM GCLC 9606.ENSP00000359258 9606.ENSP00000229416 0 0 0 0 0.202 0.921 0.9 0.995 0.999

GCLM CAT 9606.ENSP00000359258 9606.ENSP00000241052 0.053 0 0 0 0.133 0.058 0 0.633 0.678

GCLM GSTM3 9606.ENSP00000359258 9606.ENSP00000256594 0 0 0 0 0 0.114 0 0.47 0.51

GCLM SOD1 9606.ENSP00000359258 9606.ENSP00000270142 0 0 0 0 0.11 0.056 0 0.547 0.586

GCLM GSTT2B 9606.ENSP00000359258 9606.ENSP00000290765 0 0 0 0 0.063 0.156 0 0.327 0.422

GCLM GSTM1 9606.ENSP00000359258 9606.ENSP00000311469 0 0 0 0 0 0.114 0 0.588 0.62

GCLM NQO1 9606.ENSP00000359258 9606.ENSP00000319788 0.072 0 0 0 0.16 0 0 0.876 0.895

GCLM GSTA1 9606.ENSP00000359258 9606.ENSP00000335620 0 0 0 0 0.052 0.114 0 0.529 0.569

GCLM GPX4 9606.ENSP00000359258 9606.ENSP00000346103 0 0 0 0 0.095 0.056 0 0.563 0.594

GCLM GLRX2 9606.ENSP00000359258 9606.ENSP00000356410 0 0 0 0 0.064 0.056 0 0.443 0.465

GCLM MGST1 9606.ENSP00000359258 9606.ENSP00000379512 0 0 0 0 0.062 0 0 0.415 0.427

GCLM TXNRD2 9606.ENSP00000359258 9606.ENSP00000383365 0 0 0 0 0.062 0.057 0 0.457 0.477

GCLM PARK7 9606.ENSP00000359258 9606.ENSP00000418770 0.081 0 0 0 0.096 0 0 0.48 0.53

GCLM GPX3 9606.ENSP00000359258 9606.ENSP00000373477 0 0 0 0 0.095 0.056 0 0.506 0.541

GCLM SOD2 9606.ENSP00000359258 9606.ENSP00000446252 0 0 0 0 0.066 0.066 0 0.535 0.559

GCLM HMOX2 9606.ENSP00000359258 9606.ENSP00000477572 0 0 0 0 0.053 0 0 0.555 0.561

GCLM GSTP1 9606.ENSP00000359258 9606.ENSP00000381607 0 0 0 0 0.085 0.114 0 0.604 0.651

GCLM GPX1 9606.ENSP00000359258 9606.ENSP00000407375 0 0 0 0 0.095 0.056 0 0.66 0.685

GCLM TXN 9606.ENSP00000359258 9606.ENSP00000363641 0 0 0 0 0.063 0.056 0 0.692 0.704

GCLM SRXN1 9606.ENSP00000359258 9606.ENSP00000371388 0 0 0 0 0.101 0 0 0.707 0.725

GCLM NFE2L2 9606.ENSP00000359258 9606.ENSP00000380252 0 0 0 0 0 0 0 0.811 0.811

GCLM TXNRD1 9606.ENSP00000359258 9606.ENSP00000434516 0.048 0 0 0 0.135 0.06 0 0.792 0.817

GCLM CTH 9606.ENSP00000359258 9606.ENSP00000359976 0.042 0 0 0 0.064 0 0.8 0.347 0.867

GFER DDO 9606.ENSP00000248114 9606.ENSP00000357920 0 0 0 0 0 0 0 0.423 0.423

GFER GLRX2 9606.ENSP00000248114 9606.ENSP00000356410 0 0 0 0 0.065 0 0 0.427 0.441

GFER ATOX1 9606.ENSP00000248114 9606.ENSP00000430598 0 0 0 0 0.062 0 0 0.431 0.443

GFER SOD1 9606.ENSP00000248114 9606.ENSP00000270142 0 0 0 0 0.053 0 0 0.508 0.514

GFER TXN 9606.ENSP00000248114 9606.ENSP00000363641 0 0 0 0 0.062 0.274 0 0.632 0.727

GFER QSOX1 9606.ENSP00000248114 9606.ENSP00000356574 0 0 0 0 0 0 0 0.75 0.75

GFER QSOX2 9606.ENSP00000248114 9606.ENSP00000351536 0 0 0 0 0.065 0 0 0.767 0.773

GLRX2 TXN2 9606.ENSP00000356410 9606.ENSP00000216185 0.048 0 0 0 0.1 0.057 0 0.712 0.737

GLRX2 GSR 9606.ENSP00000356410 9606.ENSP00000221130 0.07 0 0 0 0.144 0.15 0 0.842 0.879

GLRX2 GCLC 9606.ENSP00000356410 9606.ENSP00000229416 0 0 0 0 0.123 0 0 0.689 0.716

GLRX2 CAT 9606.ENSP00000356410 9606.ENSP00000241052 0.06 0 0 0 0.138 0.221 0 0.692 0.779

GLRX2 GFER 9606.ENSP00000356410 9606.ENSP00000248114 0 0 0 0 0.065 0 0 0.427 0.441

GLRX2 PRDX5 9606.ENSP00000356410 9606.ENSP00000265462 0.044 0 0 0 0.073 0 0 0.675 0.687

GLRX2 SOD1 9606.ENSP00000356410 9606.ENSP00000270142 0 0 0 0 0.082 0 0 0.618 0.634

GLRX2 PRDX2 9606.ENSP00000356410 9606.ENSP00000301522 0.074 0 0 0 0.074 0.112 0 0.48 0.551

GLRX2 MSRA 9606.ENSP00000356410 9606.ENSP00000313921 0.05 0 0 0 0 0.291 0 0.332 0.511

GLRX2 PRDX6 9606.ENSP00000356410 9606.ENSP00000342026 0.074 0 0 0 0.171 0.217 0 0.567 0.704

GLRX2 GPX4 9606.ENSP00000356410 9606.ENSP00000346103 0.071 0 0 0 0.296 0.319 0 0.703 0.85

GLRX2 GCLM 9606.ENSP00000356410 9606.ENSP00000359258 0 0 0 0 0.064 0.056 0 0.443 0.465

GLRX2 SRXN1 9606.ENSP00000356410 9606.ENSP00000371388 0 0 0 0 0.166 0 0 0.438 0.511

GLRX2 GPX3 9606.ENSP00000356410 9606.ENSP00000373477 0.071 0 0 0 0.125 0.078 0 0.475 0.554

GLRX2 SOD2 9606.ENSP00000356410 9606.ENSP00000446252 0.058 0 0 0 0.152 0 0 0.491 0.558

GLRX2 TXNRD1 9606.ENSP00000356410 9606.ENSP00000434516 0.07 0 0 0.73 0.083 0.427 0 0.655 0.561

GLRX2 GPX1 9606.ENSP00000356410 9606.ENSP00000407375 0.071 0 0 0 0.125 0.078 0 0.567 0.632

GLRX2 TXNRD2 9606.ENSP00000356410 9606.ENSP00000383365 0.07 0 0 0 0.083 0.248 0 0.692 0.776

GLRX2 TXN 9606.ENSP00000356410 9606.ENSP00000363641 0.048 0 0 0 0.265 0.094 0 0.873 0.908

GPX1 NOX3 9606.ENSP00000407375 9606.ENSP00000159060 0 0 0 0 0.062 0 0 0.417 0.429

GPX1 HMOX1 9606.ENSP00000407375 9606.ENSP00000216117 0 0 0 0 0.082 0.074 0 0.729 0.75

GPX1 TXN2 9606.ENSP00000407375 9606.ENSP00000216185 0 0 0 0 0.062 0.141 0 0.66 0.702

GPX1 GSS 9606.ENSP00000407375 9606.ENSP00000216951 0 0 0 0 0.062 0 0.9 0.367 0.935

GPX1 GSR 9606.ENSP00000407375 9606.ENSP00000221130 0.05 0 0 0 0.065 0.112 0.9 0.889 0.989

GPX1 PON1 9606.ENSP00000407375 9606.ENSP00000222381 0 0 0 0 0.064 0 0 0.46 0.473

GPX1 MPO 9606.ENSP00000407375 9606.ENSP00000225275 0 0 0 0 0 0 0 0.53 0.53

GPX1 GCLC 9606.ENSP00000407375 9606.ENSP00000229416 0 0 0 0 0.062 0 0 0.765 0.77

GPX1 CAT 9606.ENSP00000407375 9606.ENSP00000241052 0 0 0 0 0.142 0.349 0 0.919 0.951

GPX1 SESN2 9606.ENSP00000407375 9606.ENSP00000253063 0 0 0 0 0 0 0 0.403 0.403

GPX1 GSTM3 9606.ENSP00000407375 9606.ENSP00000256594 0 0 0 0 0.062 0 0.65 0.671 0.882

GPX1 CYBA 9606.ENSP00000407375 9606.ENSP00000261623 0 0 0 0 0.096 0 0 0.506 0.535

GPX1 NOX4 9606.ENSP00000407375 9606.ENSP00000263317 0 0 0 0 0.062 0 0 0.623 0.631

GPX1 PPARGC1A 9606.ENSP00000407375 9606.ENSP00000264867 0 0 0 0 0 0 0.8 0.602 0.917

GPX1 PRDX5 9606.ENSP00000407375 9606.ENSP00000265462 0 0 0 0 0.093 0.13 0 0.65 0.7

GPX1 SOD1 9606.ENSP00000407375 9606.ENSP00000270142 0.044 0 0 0 0.094 0.178 0.9 0.838 0.986

GPX1 GSTT2B 9606.ENSP00000407375 9606.ENSP00000290765 0 0 0 0 0.065 0 0.65 0.335 0.763

GPX1 NOS3 9606.ENSP00000407375 9606.ENSP00000297494 0 0 0 0 0 0 0 0.529 0.529

GPX1 PRDX2 9606.ENSP00000407375 9606.ENSP00000301522 0 0 0 0 0.062 0.185 0 0.63 0.692

GPX1 GSTM1 9606.ENSP00000407375 9606.ENSP00000311469 0 0 0 0 0.062 0 0.65 0.702 0.893

GPX1 UCP2 9606.ENSP00000407375 9606.ENSP00000312029 0 0 0 0 0.064 0 0 0.516 0.528

GPX1 MSRA 9606.ENSP00000407375 9606.ENSP00000313921 0.072 0 0 0 0.062 0.247 0 0.416 0.566

GPX1 DUOX1 9606.ENSP00000407375 9606.ENSP00000317997 0 0 0 0 0 0 0 0.465 0.465

GPX1 NQO1 9606.ENSP00000407375 9606.ENSP00000319788 0 0 0 0 0 0 0 0.684 0.684

GPX1 GSTA1 9606.ENSP00000407375 9606.ENSP00000335620 0 0 0 0 0.062 0 0.65 0.536 0.834

GPX1 PRDX6 9606.ENSP00000407375 9606.ENSP00000342026 0 0 0 0 0.063 0.174 0 0.626 0.685

GPX1 GPX4 9606.ENSP00000407375 9606.ENSP00000346103 0 0 0.448 0.804 0.13 0 0.5 0.875 0.655

GPX1 GLRX2 9606.ENSP00000407375 9606.ENSP00000356410 0.071 0 0 0 0.125 0.078 0 0.567 0.632

GPX1 NCF2 9606.ENSP00000407375 9606.ENSP00000356505 0 0 0 0 0 0 0 0.4 0.4

GPX1 GCLM 9606.ENSP00000407375 9606.ENSP00000359258 0 0 0 0 0.095 0.056 0 0.66 0.685

GPX1 NOX1 9606.ENSP00000407375 9606.ENSP00000362057 0 0 0 0 0.062 0 0 0.552 0.562

GPX1 TXN 9606.ENSP00000407375 9606.ENSP00000363641 0 0 0 0 0.063 0.284 0 0.728 0.802

GPX1 CYBB 9606.ENSP00000407375 9606.ENSP00000367851 0 0 0 0 0.062 0 0 0.623 0.631

GPX1 CYP1A1 9606.ENSP00000407375 9606.ENSP00000369050 0 0 0 0 0 0 0 0.458 0.458

GPX1 SRXN1 9606.ENSP00000407375 9606.ENSP00000371388 0 0 0 0 0.06 0 0 0.552 0.56

GPX1 SOD3 9606.ENSP00000407375 9606.ENSP00000371554 0.044 0 0 0 0.094 0.178 0.9 0.64 0.969

GPX1 SUOX 9606.ENSP00000407375 9606.ENSP00000377668 0.102 0 0 0 0 0 0 0.461 0.495

GPX1 MGST1 9606.ENSP00000407375 9606.ENSP00000379512 0 0 0 0 0.064 0 0.65 0.392 0.783

GPX1 NFE2L2 9606.ENSP00000407375 9606.ENSP00000380252 0 0 0 0 0 0 0 0.667 0.667

GPX1 VIMP 9606.ENSP00000407375 9606.ENSP00000381282 0 0 0 0 0 0 0 0.698 0.698

GPX1 GSTP1 9606.ENSP00000407375 9606.ENSP00000381607 0 0 0 0 0.156 0 0.65 0.626 0.88

GPX1 TXNRD2 9606.ENSP00000407375 9606.ENSP00000383365 0.05 0 0 0 0.063 0.15 0 0.743 0.779

GPX1 DUOX2 9606.ENSP00000407375 9606.ENSP00000475084 0 0 0 0 0 0 0 0.454 0.454

GPX1 PARK7 9606.ENSP00000407375 9606.ENSP00000418770 0 0 0 0 0.066 0.141 0 0.423 0.496

GPX1 SEPP1 9606.ENSP00000407375 9606.ENSP00000420939 0 0 0 0 0.062 0 0 0.811 0.815

GPX1 TXNRD1 9606.ENSP00000407375 9606.ENSP00000434516 0.23 0 0 0 0.144 0.167 0 0.831 0.895

GPX1 SOD2 9606.ENSP00000407375 9606.ENSP00000446252 0 0 0 0 0.062 0.254 0.9 0.856 0.988

GPX3 HMOX1 9606.ENSP00000373477 9606.ENSP00000216117 0 0 0 0 0 0.074 0 0.68 0.691

GPX3 TXN2 9606.ENSP00000373477 9606.ENSP00000216185 0 0 0 0 0.062 0.141 0 0.489 0.552

GPX3 GSS 9606.ENSP00000373477 9606.ENSP00000216951 0 0 0 0 0 0 0.9 0.41 0.938

GPX3 GSR 9606.ENSP00000373477 9606.ENSP00000221130 0.05 0 0 0 0.065 0.112 0.9 0.863 0.987

GPX3 MPO 9606.ENSP00000373477 9606.ENSP00000225275 0 0 0 0 0 0 0 0.4 0.4

GPX3 GCLC 9606.ENSP00000373477 9606.ENSP00000229416 0 0 0 0 0.062 0 0 0.558 0.568

GPX3 CAT 9606.ENSP00000373477 9606.ENSP00000241052 0 0 0 0 0.142 0.349 0 0.865 0.918

GPX3 GSTM3 9606.ENSP00000373477 9606.ENSP00000256594 0 0 0 0 0.062 0 0.65 0.658 0.878

GPX3 NOX4 9606.ENSP00000373477 9606.ENSP00000263317 0 0 0 0 0 0 0 0.515 0.515

GPX3 PPARGC1A 9606.ENSP00000373477 9606.ENSP00000264867 0 0 0 0 0 0 0.8 0.218 0.836

GPX3 PRDX5 9606.ENSP00000373477 9606.ENSP00000265462 0 0 0 0 0.066 0.13 0 0.576 0.626

GPX3 SOD1 9606.ENSP00000373477 9606.ENSP00000270142 0.044 0 0 0 0.094 0.178 0.9 0.627 0.968

GPX3 GSTT2B 9606.ENSP00000373477 9606.ENSP00000290765 0 0 0 0 0.065 0 0.65 0.334 0.763

GPX3 PRDX2 9606.ENSP00000373477 9606.ENSP00000301522 0 0 0 0 0.062 0.185 0 0.58 0.651

GPX3 GSTM1 9606.ENSP00000373477 9606.ENSP00000311469 0 0 0 0 0.062 0 0.65 0.493 0.819

GPX3 MSRA 9606.ENSP00000373477 9606.ENSP00000313921 0.072 0 0 0 0.062 0.247 0 0.372 0.533

GPX3 DUOX1 9606.ENSP00000373477 9606.ENSP00000317997 0 0 0 0 0 0 0 0.417 0.417

GPX3 NQO1 9606.ENSP00000373477 9606.ENSP00000319788 0 0 0 0 0 0 0 0.52 0.52

GPX3 GSTA1 9606.ENSP00000373477 9606.ENSP00000335620 0 0 0 0 0.083 0 0.65 0.38 0.783

GPX3 PRDX6 9606.ENSP00000373477 9606.ENSP00000342026 0 0 0 0 0.063 0.174 0 0.536 0.61

GPX3 GLRX2 9606.ENSP00000373477 9606.ENSP00000356410 0.071 0 0 0 0.125 0.078 0 0.475 0.554

GPX3 GCLM 9606.ENSP00000373477 9606.ENSP00000359258 0 0 0 0 0.095 0.056 0 0.506 0.541

GPX3 NOX1 9606.ENSP00000373477 9606.ENSP00000362057 0 0 0 0 0 0 0 0.407 0.407

GPX3 TXN 9606.ENSP00000373477 9606.ENSP00000363641 0 0 0 0 0.062 0.212 0 0.732 0.785

GPX3 CYBB 9606.ENSP00000373477 9606.ENSP00000367851 0 0 0 0 0 0 0 0.437 0.437

GPX3 SRXN1 9606.ENSP00000373477 9606.ENSP00000371388 0 0 0 0 0.06 0 0 0.47 0.48

GPX3 SOD3 9606.ENSP00000373477 9606.ENSP00000371554 0.044 0 0 0 0.161 0.178 0.9 0.609 0.969

GPX3 PARK7 9606.ENSP00000373477 9606.ENSP00000418770 0 0 0 0 0.066 0.141 0 0.32 0.407

GPX3 NFE2L2 9606.ENSP00000373477 9606.ENSP00000380252 0 0 0 0 0 0 0 0.533 0.533

GPX3 TXNRD2 9606.ENSP00000373477 9606.ENSP00000383365 0.05 0 0 0 0.063 0.15 0 0.694 0.738

GPX3 MGST1 9606.ENSP00000373477 9606.ENSP00000379512 0 0 0 0 0.062 0 0.65 0.293 0.747

GPX3 VIMP 9606.ENSP00000373477 9606.ENSP00000381282 0 0 0 0 0 0 0 0.768 0.768

GPX3 GSTP1 9606.ENSP00000373477 9606.ENSP00000381607 0 0 0 0 0.062 0 0.65 0.46 0.807

GPX3 TXNRD1 9606.ENSP00000373477 9606.ENSP00000434516 0.23 0 0 0 0.144 0.167 0 0.767 0.855

GPX3 SEPP1 9606.ENSP00000373477 9606.ENSP00000420939 0 0 0 0 0.117 0 0 0.892 0.901

GPX3 SOD2 9606.ENSP00000373477 9606.ENSP00000446252 0 0 0 0 0.062 0.254 0.9 0.646 0.971

GPX4 HMOX1 9606.ENSP00000346103 9606.ENSP00000216117 0 0 0 0 0.062 0.074 0 0.775 0.788

GPX4 TXN2 9606.ENSP00000346103 9606.ENSP00000216185 0 0 0 0 0.065 0.141 0 0.626 0.673

GPX4 GSS 9606.ENSP00000346103 9606.ENSP00000216951 0 0 0 0 0 0 0.9 0.465 0.944

GPX4 GSR 9606.ENSP00000346103 9606.ENSP00000221130 0.05 0 0 0 0.065 0.112 0.9 0.87 0.987

GPX4 MPO 9606.ENSP00000346103 9606.ENSP00000225275 0 0 0 0 0 0 0 0.433 0.433

GPX4 GCLC 9606.ENSP00000346103 9606.ENSP00000229416 0 0 0 0 0.062 0 0 0.78 0.785

GPX4 LOX 9606.ENSP00000346103 9606.ENSP00000231004 0 0 0 0 0.062 0 0 0.395 0.408

GPX4 CAT 9606.ENSP00000346103 9606.ENSP00000241052 0 0 0 0 0.142 0.349 0 0.869 0.92

GPX4 GSTM3 9606.ENSP00000346103 9606.ENSP00000256594 0 0 0 0 0.062 0 0.65 0.714 0.897

GPX4 CYBA 9606.ENSP00000346103 9606.ENSP00000261623 0 0 0 0 0 0 0 0.427 0.427

GPX4 NOX4 9606.ENSP00000346103 9606.ENSP00000263317 0 0 0 0 0 0 0 0.541 0.541

GPX4 PRDX5 9606.ENSP00000346103 9606.ENSP00000265462 0 0 0 0 0.111 0.13 0 0.639 0.696

GPX4 SOD1 9606.ENSP00000346103 9606.ENSP00000270142 0.044 0 0 0 0.138 0.376 0 0.86 0.918

GPX4 GSTT2B 9606.ENSP00000346103 9606.ENSP00000290765 0 0 0 0 0.065 0 0.65 0.297 0.749

GPX4 PRDX2 9606.ENSP00000346103 9606.ENSP00000301522 0 0 0 0 0.097 0.47 0 0.692 0.84

GPX4 GSTM1 9606.ENSP00000346103 9606.ENSP00000311469 0 0 0 0 0.062 0 0.65 0.459 0.807

GPX4 UCP2 9606.ENSP00000346103 9606.ENSP00000312029 0 0 0 0 0.064 0 0 0.397 0.411

GPX4 MSRA 9606.ENSP00000346103 9606.ENSP00000313921 0.072 0 0 0 0.062 0.247 0 0.49 0.621

GPX4 DUOX1 9606.ENSP00000346103 9606.ENSP00000317997 0 0 0 0 0 0 0 0.538 0.538

GPX4 NQO1 9606.ENSP00000346103 9606.ENSP00000319788 0 0 0 0 0 0 0 0.583 0.583

GPX4 GSTA1 9606.ENSP00000346103 9606.ENSP00000335620 0 0 0 0 0.062 0 0.65 0.477 0.813

GPX4 PRDX6 9606.ENSP00000346103 9606.ENSP00000342026 0 0 0 0 0.064 0.378 0 0.739 0.834

GPX4 NOX5 9606.ENSP00000346103 9606.ENSP00000373518 0 0 0 0 0 0 0 0.426 0.426

GPX4 DUOX2 9606.ENSP00000346103 9606.ENSP00000475084 0 0 0 0 0 0 0 0.48 0.48

GPX4 CYBB 9606.ENSP00000346103 9606.ENSP00000367851 0 0 0 0 0 0 0 0.514 0.514

GPX4 HMOX2 9606.ENSP00000346103 9606.ENSP00000477572 0 0 0 0 0.062 0.074 0 0.488 0.516

GPX4 NOX1 9606.ENSP00000346103 9606.ENSP00000362057 0 0 0 0 0 0 0 0.539 0.539

GPX4 GCLM 9606.ENSP00000346103 9606.ENSP00000359258 0 0 0 0 0.095 0.056 0 0.563 0.594

GPX4 CYP1A1 9606.ENSP00000346103 9606.ENSP00000369050 0 0 0 0 0 0 0 0.606 0.606

GPX4 SOD3 9606.ENSP00000346103 9606.ENSP00000371554 0.044 0 0 0 0.094 0.178 0 0.547 0.634

GPX4 PARK7 9606.ENSP00000346103 9606.ENSP00000418770 0 0 0 0 0.094 0.346 0 0.45 0.646

GPX4 GPX1 9606.ENSP00000346103 9606.ENSP00000407375 0 0 0.448 0.804 0.13 0 0.5 0.875 0.655

GPX4 SRXN1 9606.ENSP00000346103 9606.ENSP00000371388 0 0 0 0 0.062 0 0 0.658 0.666

GPX4 NFE2L2 9606.ENSP00000346103 9606.ENSP00000380252 0 0 0 0 0 0 0 0.7 0.7

GPX4 VIMP 9606.ENSP00000346103 9606.ENSP00000381282 0 0 0 0 0.062 0 0 0.715 0.722

GPX4 MGST1 9606.ENSP00000346103 9606.ENSP00000379512 0 0 0 0 0.062 0 0.65 0.258 0.735

GPX4 SEPP1 9606.ENSP00000346103 9606.ENSP00000420939 0 0 0 0 0.062 0 0 0.812 0.816

GPX4 GSTP1 9606.ENSP00000346103 9606.ENSP00000381607 0 0 0 0 0.089 0 0.65 0.488 0.822

GPX4 GLRX2 9606.ENSP00000346103 9606.ENSP00000356410 0.071 0 0 0 0.296 0.319 0 0.703 0.85

GPX4 TXNRD2 9606.ENSP00000346103 9606.ENSP00000383365 0.05 0 0 0 0.063 0.15 0 0.828 0.852

GPX4 TXN 9606.ENSP00000346103 9606.ENSP00000363641 0 0 0 0 0.087 0.212 0 0.828 0.865

GPX4 TXNRD1 9606.ENSP00000346103 9606.ENSP00000434516 0.23 0 0 0 0.144 0.167 0 0.847 0.904

GPX4 SOD2 9606.ENSP00000346103 9606.ENSP00000446252 0 0 0 0 0.064 0.539 0 0.843 0.926

GSR HMOX1 9606.ENSP00000221130 9606.ENSP00000216117 0 0 0 0 0.083 0.098 0 0.742 0.768

GSR TXN2 9606.ENSP00000221130 9606.ENSP00000216185 0.051 0 0 0 0.078 0.22 0 0.575 0.671

GSR GSS 9606.ENSP00000221130 9606.ENSP00000216951 0 0 0 0 0.086 0.261 0.9 0.711 0.977

GSR PPARGC1A 9606.ENSP00000221130 9606.ENSP00000264867 0 0 0 0 0 0 0 0.401 0.401

GSR PON1 9606.ENSP00000221130 9606.ENSP00000222381 0 0 0 0 0 0 0 0.404 0.404

GSR NOS1 9606.ENSP00000221130 9606.ENSP00000477999 0 0 0 0 0 0 0 0.405 0.405

GSR CYP1A1 9606.ENSP00000221130 9606.ENSP00000369050 0 0 0 0 0.062 0 0 0.402 0.415

GSR NDUFS2 9606.ENSP00000221130 9606.ENSP00000356972 0 0 0 0 0.157 0.247 0 0.152 0.415

GSR UCP2 9606.ENSP00000221130 9606.ENSP00000312029 0 0 0 0 0.064 0.126 0 0.37 0.439

GSR MSRA 9606.ENSP00000221130 9606.ENSP00000313921 0.057 0 0 0 0.083 0 0 0.404 0.44

GSR DUOX1 9606.ENSP00000221130 9606.ENSP00000317997 0 0 0 0 0 0 0 0.442 0.442

GSR MTHFR 9606.ENSP00000221130 9606.ENSP00000365777 0.163 0 0 0 0.062 0 0 0.358 0.452

GSR PARK7 9606.ENSP00000221130 9606.ENSP00000418770 0.077 0 0 0 0 0 0 0.431 0.453

GSR NOX1 9606.ENSP00000221130 9606.ENSP00000362057 0 0 0 0 0 0 0 0.478 0.478

GSR TXNRD1 9606.ENSP00000221130 9606.ENSP00000434516 0.07 0 0.428 0.809 0.203 0.15 0 0.916 0.478

GSR CTH 9606.ENSP00000221130 9606.ENSP00000359976 0 0 0 0 0.084 0 0 0.456 0.481

GSR CYBB 9606.ENSP00000221130 9606.ENSP00000367851 0 0 0 0 0 0 0 0.481 0.481

GSR NOX4 9606.ENSP00000221130 9606.ENSP00000263317 0 0 0 0 0 0 0 0.491 0.491

GSR MT1X 9606.ENSP00000221130 9606.ENSP00000377995 0 0 0 0 0 0 0 0.52 0.52

GSR NOS3 9606.ENSP00000221130 9606.ENSP00000297494 0 0 0 0 0 0 0 0.525 0.525

GSR MPO 9606.ENSP00000221130 9606.ENSP00000225275 0 0 0 0 0 0.166 0 0.49 0.556

GSR SOD3 9606.ENSP00000221130 9606.ENSP00000371554 0 0 0 0 0.055 0.117 0 0.669 0.7

GSR NFE2L2 9606.ENSP00000221130 9606.ENSP00000380252 0 0 0 0 0 0 0 0.716 0.716

GSR PRDX2 9606.ENSP00000221130 9606.ENSP00000301522 0 0 0 0 0.159 0.067 0 0.682 0.729

GSR PRDX6 9606.ENSP00000221130 9606.ENSP00000342026 0 0 0 0 0.083 0.115 0 0.695 0.731

GSR NQO1 9606.ENSP00000221130 9606.ENSP00000319788 0 0 0 0 0.064 0 0 0.73 0.736

GSR SRXN1 9606.ENSP00000221130 9606.ENSP00000371388 0 0 0 0 0.256 0 0 0.728 0.789

GSR SOD2 9606.ENSP00000221130 9606.ENSP00000446252 0.07 0 0 0 0.11 0 0 0.768 0.791

GSR GSTT2B 9606.ENSP00000221130 9606.ENSP00000290765 0 0 0 0 0.112 0.061 0.65 0.436 0.813

GSR MGST1 9606.ENSP00000221130 9606.ENSP00000379512 0 0 0 0 0.062 0 0.65 0.483 0.815

GSR GCLM 9606.ENSP00000221130 9606.ENSP00000359258 0 0 0 0 0.064 0.057 0 0.809 0.816

GSR PRDX5 9606.ENSP00000221130 9606.ENSP00000265462 0.166 0 0 0 0.138 0 0 0.774 0.823

GSR GSTM1 9606.ENSP00000221130 9606.ENSP00000311469 0 0 0 0 0.064 0 0.65 0.544 0.837

GSR GSTA1 9606.ENSP00000221130 9606.ENSP00000335620 0 0 0 0 0.064 0 0.65 0.576 0.849

GSR GSTP1 9606.ENSP00000221130 9606.ENSP00000381607 0 0 0 0 0.064 0 0.65 0.58 0.85

GSR SOD1 9606.ENSP00000221130 9606.ENSP00000270142 0 0 0 0 0.073 0.117 0 0.84 0.858

GSR GSTM3 9606.ENSP00000221130 9606.ENSP00000256594 0 0 0 0 0.064 0 0.65 0.619 0.864

GSR GLRX2 9606.ENSP00000221130 9606.ENSP00000356410 0.07 0 0 0 0.144 0.15 0 0.842 0.879

GSR GCLC 9606.ENSP00000221130 9606.ENSP00000229416 0 0 0 0 0.184 0 0 0.911 0.924

GSR TXN 9606.ENSP00000221130 9606.ENSP00000363641 0.051 0 0 0 0.246 0.338 0 0.905 0.949

GSR CAT 9606.ENSP00000221130 9606.ENSP00000241052 0 0 0 0 0.218 0.26 0 0.953 0.97

GSR GPX3 9606.ENSP00000221130 9606.ENSP00000373477 0.05 0 0 0 0.065 0.112 0.9 0.863 0.987

GSR GPX4 9606.ENSP00000221130 9606.ENSP00000346103 0.05 0 0 0 0.065 0.112 0.9 0.87 0.987

GSR GPX1 9606.ENSP00000221130 9606.ENSP00000407375 0.05 0 0 0 0.065 0.112 0.9 0.889 0.989

GSS TXNRD2 9606.ENSP00000216951 9606.ENSP00000383365 0 0 0 0 0.065 0.261 0 0.207 0.404

GSS MSRA 9606.ENSP00000216951 9606.ENSP00000313921 0 0 0 0 0 0 0 0.418 0.418

GSS TXNRD1 9606.ENSP00000216951 9606.ENSP00000434516 0 0 0 0 0.096 0.261 0 0.251 0.456

GSS TXN 9606.ENSP00000216951 9606.ENSP00000363641 0 0 0 0 0.09 0.119 0 0.47 0.538

GSS CAT 9606.ENSP00000216951 9606.ENSP00000241052 0 0 0 0 0.063 0.26 0 0.477 0.606

GSS CTH 9606.ENSP00000216951 9606.ENSP00000359976 0 0 0 0 0.062 0 0 0.631 0.639

GSS MGST1 9606.ENSP00000216951 9606.ENSP00000379512 0 0 0 0 0.062 0 0.65 0.181 0.707

GSS GSTA1 9606.ENSP00000216951 9606.ENSP00000335620 0 0 0 0 0.065 0.16 0.65 0.124 0.727

GSS GSTM1 9606.ENSP00000216951 9606.ENSP00000311469 0 0 0 0 0.065 0.16 0.65 0.125 0.727

GSS GSTM3 9606.ENSP00000216951 9606.ENSP00000256594 0 0 0 0 0.065 0.16 0.65 0.144 0.733

GSS GSTP1 9606.ENSP00000216951 9606.ENSP00000381607 0 0 0 0 0.065 0.16 0.65 0.215 0.755

GSS GSTT2B 9606.ENSP00000216951 9606.ENSP00000290765 0 0 0 0 0 0.065 0.65 0.354 0.77

GSS PRDX6 9606.ENSP00000216951 9606.ENSP00000342026 0 0 0 0 0.081 0 0.9 0.11 0.911

GSS GPX1 9606.ENSP00000216951 9606.ENSP00000407375 0 0 0 0 0.062 0 0.9 0.367 0.935

GSS GPX3 9606.ENSP00000216951 9606.ENSP00000373477 0 0 0 0 0 0 0.9 0.41 0.938

GSS GPX4 9606.ENSP00000216951 9606.ENSP00000346103 0 0 0 0 0 0 0.9 0.465 0.944

GSS GSR 9606.ENSP00000216951 9606.ENSP00000221130 0 0 0 0 0.086 0.261 0.9 0.711 0.977

GSS GCLM 9606.ENSP00000216951 9606.ENSP00000359258 0 0 0 0 0.067 0.223 0.9 0.75 0.979

GSS GCLC 9606.ENSP00000216951 9606.ENSP00000229416 0 0 0 0 0.108 0.348 0.9 0.847 0.989

GSTA1 HMOX1 9606.ENSP00000335620 9606.ENSP00000216117 0 0 0 0 0 0 0 0.589 0.589

GSTA1 GSS 9606.ENSP00000335620 9606.ENSP00000216951 0 0 0 0 0.065 0.16 0.65 0.124 0.727

GSTA1 GSR 9606.ENSP00000335620 9606.ENSP00000221130 0 0 0 0 0.064 0 0.65 0.576 0.849

GSTA1 GCLC 9606.ENSP00000335620 9606.ENSP00000229416 0 0 0 0 0.052 0 0 0.641 0.646

GSTA1 CAT 9606.ENSP00000335620 9606.ENSP00000241052 0 0 0 0 0.062 0.178 0 0.506 0.586

GSTA1 GSTM3 9606.ENSP00000335620 9606.ENSP00000256594 0 0 0 0.648 0 0 0.65 0.681 0.732

GSTA1 GSTT2B 9606.ENSP00000335620 9606.ENSP00000290765 0 0 0 0 0.065 0.147 0.65 0.667 0.894

GSTA1 GSTM1 9606.ENSP00000335620 9606.ENSP00000311469 0 0 0 0.628 0 0 0.65 0.846 0.759

GSTA1 NQO1 9606.ENSP00000335620 9606.ENSP00000319788 0 0 0 0 0.049 0 0 0.693 0.696

GSTA1 SOD2 9606.ENSP00000335620 9606.ENSP00000446252 0 0 0 0 0.064 0 0 0.401 0.415

GSTA1 TXNRD1 9606.ENSP00000335620 9606.ENSP00000434516 0 0 0 0 0.081 0 0 0.493 0.514

GSTA1 NFE2L2 9606.ENSP00000335620 9606.ENSP00000380252 0 0 0 0 0 0 0 0.528 0.528

GSTA1 GCLM 9606.ENSP00000335620 9606.ENSP00000359258 0 0 0 0 0.052 0.114 0 0.529 0.569

GSTA1 PRDX6 9606.ENSP00000335620 9606.ENSP00000342026 0 0 0 0 0.062 0.078 0.65 0.131 0.701

GSTA1 GSTP1 9606.ENSP00000335620 9606.ENSP00000381607 0 0 0 0.744 0.049 0 0.65 0.851 0.728

GSTA1 GPX3 9606.ENSP00000335620 9606.ENSP00000373477 0 0 0 0 0.083 0 0.65 0.38 0.783

GSTA1 GPX4 9606.ENSP00000335620 9606.ENSP00000346103 0 0 0 0 0.062 0 0.65 0.477 0.813

GSTA1 MGST1 9606.ENSP00000335620 9606.ENSP00000379512 0 0 0 0 0.064 0 0.65 0.515 0.827

GSTA1 GPX1 9606.ENSP00000335620 9606.ENSP00000407375 0 0 0 0 0.062 0 0.65 0.536 0.834

GSTA1 CYP1A1 9606.ENSP00000335620 9606.ENSP00000369050 0 0 0 0 0.062 0.057 0.65 0.613 0.864

GSTM1 ERCC1 9606.ENSP00000311469 9606.ENSP00000013807 0 0 0 0 0 0 0 0.519 0.519

GSTM1 HMOX1 9606.ENSP00000311469 9606.ENSP00000216117 0 0 0 0 0 0 0 0.552 0.552

GSTM1 GSS 9606.ENSP00000311469 9606.ENSP00000216951 0 0 0 0 0.065 0.16 0.65 0.125 0.727

GSTM1 GSR 9606.ENSP00000311469 9606.ENSP00000221130 0 0 0 0 0.064 0 0.65 0.544 0.837

GSTM1 PON1 9606.ENSP00000311469 9606.ENSP00000222381 0 0 0 0 0.062 0 0 0.517 0.527

GSTM1 MPO 9606.ENSP00000311469 9606.ENSP00000225275 0 0 0 0 0 0 0 0.467 0.467

GSTM1 GCLC 9606.ENSP00000311469 9606.ENSP00000229416 0 0 0 0 0 0 0 0.605 0.605

GSTM1 CAT 9606.ENSP00000311469 9606.ENSP00000241052 0 0 0 0 0.056 0.178 0 0.556 0.625

GSTM1 GSTM3 9606.ENSP00000311469 9606.ENSP00000256594 0 0 0.447 0.974 0.065 0.232 0.65 0.821 0.735

GSTM1 GSTT2B 9606.ENSP00000311469 9606.ENSP00000290765 0 0 0 0 0.065 0.147 0.65 0.666 0.894

GSTM1 CDKN2A 9606.ENSP00000311469 9606.ENSP00000418915 0 0 0 0 0.062 0.091 0 0.362 0.409

GSTM1 NFE2L2 9606.ENSP00000311469 9606.ENSP00000380252 0 0 0 0 0 0 0 0.503 0.503

GSTM1 TXNRD1 9606.ENSP00000311469 9606.ENSP00000434516 0 0 0 0 0.081 0 0 0.491 0.513

GSTM1 SOD2 9606.ENSP00000311469 9606.ENSP00000446252 0 0 0 0 0.064 0 0 0.54 0.551

GSTM1 ERCC2 9606.ENSP00000311469 9606.ENSP00000375809 0 0 0 0 0 0 0 0.601 0.601

GSTM1 GCLM 9606.ENSP00000311469 9606.ENSP00000359258 0 0 0 0 0 0.114 0 0.588 0.62

GSTM1 MTHFR 9606.ENSP00000311469 9606.ENSP00000365777 0 0 0 0 0 0 0 0.699 0.699

GSTM1 GSTP1 9606.ENSP00000311469 9606.ENSP00000381607 0 0 0 0.782 0 0 0.65 0.927 0.72

GSTM1 NQO1 9606.ENSP00000311469 9606.ENSP00000319788 0 0 0 0 0.06 0 0 0.735 0.74

GSTM1 GSTA1 9606.ENSP00000311469 9606.ENSP00000335620 0 0 0 0.628 0 0 0.65 0.846 0.759

GSTM1 PRDX6 9606.ENSP00000311469 9606.ENSP00000342026 0 0 0 0 0.062 0.078 0.65 0.317 0.765

GSTM1 GPX4 9606.ENSP00000311469 9606.ENSP00000346103 0 0 0 0 0.062 0 0.65 0.459 0.807

GSTM1 MGST1 9606.ENSP00000311469 9606.ENSP00000379512 0 0 0 0 0.062 0 0.65 0.465 0.809

GSTM1 GPX3 9606.ENSP00000311469 9606.ENSP00000373477 0 0 0 0 0.062 0 0.65 0.493 0.819

GSTM1 GPX1 9606.ENSP00000311469 9606.ENSP00000407375 0 0 0 0 0.062 0 0.65 0.702 0.893

GSTM1 CYP1A1 9606.ENSP00000311469 9606.ENSP00000369050 0 0 0 0 0.062 0.057 0.65 0.87 0.954

GSTM3 HMOX1 9606.ENSP00000256594 9606.ENSP00000216117 0 0 0 0 0 0 0 0.597 0.597

GSTM3 GSS 9606.ENSP00000256594 9606.ENSP00000216951 0 0 0 0 0.065 0.16 0.65 0.144 0.733

GSTM3 GSR 9606.ENSP00000256594 9606.ENSP00000221130 0 0 0 0 0.064 0 0.65 0.619 0.864

GSTM3 GCLC 9606.ENSP00000256594 9606.ENSP00000229416 0 0 0 0 0 0 0 0.554 0.554

GSTM3 CAT 9606.ENSP00000256594 9606.ENSP00000241052 0 0 0 0 0.053 0.178 0 0.604 0.665

GSTM3 TXN 9606.ENSP00000256594 9606.ENSP00000363641 0 0 0 0 0 0 0 0.47 0.47

GSTM3 NFE2L2 9606.ENSP00000256594 9606.ENSP00000380252 0 0 0 0 0 0 0 0.499 0.499

GSTM3 GCLM 9606.ENSP00000256594 9606.ENSP00000359258 0 0 0 0 0 0.114 0 0.47 0.51

GSTM3 NQO1 9606.ENSP00000256594 9606.ENSP00000319788 0 0 0 0 0.049 0 0 0.567 0.571

GSTM3 GSTP1 9606.ENSP00000256594 9606.ENSP00000381607 0 0 0 0.828 0 0 0.65 0.813 0.698

GSTM3 GSTA1 9606.ENSP00000256594 9606.ENSP00000335620 0 0 0 0.648 0 0 0.65 0.681 0.732

GSTM3 GSTM1 9606.ENSP00000256594 9606.ENSP00000311469 0 0 0.447 0.974 0.065 0.232 0.65 0.821 0.735

GSTM3 PRDX6 9606.ENSP00000256594 9606.ENSP00000342026 0 0 0 0 0.062 0.078 0.65 0.281 0.753

GSTM3 MGST1 9606.ENSP00000256594 9606.ENSP00000379512 0 0 0 0 0.062 0 0.65 0.535 0.834

GSTM3 CYP1A1 9606.ENSP00000256594 9606.ENSP00000369050 0 0 0 0 0 0.057 0.65 0.561 0.842

GSTM3 GPX3 9606.ENSP00000256594 9606.ENSP00000373477 0 0 0 0 0.062 0 0.65 0.658 0.878

GSTM3 GPX1 9606.ENSP00000256594 9606.ENSP00000407375 0 0 0 0 0.062 0 0.65 0.671 0.882

GSTM3 GSTT2B 9606.ENSP00000256594 9606.ENSP00000290765 0 0 0 0 0.065 0.147 0.65 0.671 0.896

GSTM3 GPX4 9606.ENSP00000256594 9606.ENSP00000346103 0 0 0 0 0.062 0 0.65 0.714 0.897

GSTP1 ERCC1 9606.ENSP00000381607 9606.ENSP00000013807 0 0 0 0 0.062 0 0 0.616 0.624

GSTP1 HMOX1 9606.ENSP00000381607 9606.ENSP00000216117 0 0 0 0 0 0 0 0.556 0.556

GSTP1 GSS 9606.ENSP00000381607 9606.ENSP00000216951 0 0 0 0 0.065 0.16 0.65 0.215 0.755

GSTP1 GSR 9606.ENSP00000381607 9606.ENSP00000221130 0 0 0 0 0.064 0 0.65 0.58 0.85

GSTP1 PON1 9606.ENSP00000381607 9606.ENSP00000222381 0 0 0 0 0.062 0 0 0.402 0.415

GSTP1 MPO 9606.ENSP00000381607 9606.ENSP00000225275 0 0 0 0 0 0 0 0.421 0.42

GSTP1 GCLC 9606.ENSP00000381607 9606.ENSP00000229416 0 0 0 0 0 0 0 0.732 0.732

GSTP1 CAT 9606.ENSP00000381607 9606.ENSP00000241052 0 0 0 0 0.053 0.178 0 0.535 0.606

GSTP1 GSTM3 9606.ENSP00000381607 9606.ENSP00000256594 0 0 0 0.828 0 0 0.65 0.813 0.698

GSTP1 TIMP3 9606.ENSP00000381607 9606.ENSP00000266085 0 0 0 0 0 0 0 0.617 0.617

GSTP1 SOD1 9606.ENSP00000381607 9606.ENSP00000270142 0 0 0 0 0.063 0.129 0 0.47 0.53

GSTP1 GSTT2B 9606.ENSP00000381607 9606.ENSP00000290765 0 0 0 0 0.065 0.352 0.65 0.634 0.912

GSTP1 PRDX2 9606.ENSP00000381607 9606.ENSP00000301522 0 0 0 0 0.108 0.156 0 0.55 0.632

GSTP1 GSTM1 9606.ENSP00000381607 9606.ENSP00000311469 0 0 0 0.782 0 0 0.65 0.927 0.72

GSTP1 NQO1 9606.ENSP00000381607 9606.ENSP00000319788 0 0 0 0 0.065 0 0 0.714 0.721

GSTP1 GSTA1 9606.ENSP00000381607 9606.ENSP00000335620 0 0 0 0.744 0.049 0 0.65 0.851 0.728

GSTP1 PRDX6 9606.ENSP00000381607 9606.ENSP00000342026 0 0 0 0 0.083 0.558 0.8 0.475 0.951

GSTP1 GPX4 9606.ENSP00000381607 9606.ENSP00000346103 0 0 0 0 0.089 0 0.65 0.488 0.822

GSTP1 GCLM 9606.ENSP00000381607 9606.ENSP00000359258 0 0 0 0 0.085 0.114 0 0.604 0.651

GSTP1 TXN 9606.ENSP00000381607 9606.ENSP00000363641 0 0 0 0 0.07 0.117 0 0.472 0.529

GSTP1 MTHFR 9606.ENSP00000381607 9606.ENSP00000365777 0 0 0 0 0 0 0 0.664 0.664

GSTP1 CYP1A1 9606.ENSP00000381607 9606.ENSP00000369050 0 0 0 0 0 0.057 0.65 0.763 0.915

GSTP1 DRD3 9606.ENSP00000381607 9606.ENSP00000373169 0 0 0 0 0 0.063 0 0.426 0.439

GSTP1 GPX3 9606.ENSP00000381607 9606.ENSP00000373477 0 0 0 0 0.062 0 0.65 0.46 0.807

GSTP1 ERCC2 9606.ENSP00000381607 9606.ENSP00000375809 0 0 0 0 0 0 0 0.609 0.609

GSTP1 MGST1 9606.ENSP00000381607 9606.ENSP00000379512 0 0 0 0 0.062 0 0.65 0.509 0.824

GSTP1 NFE2L2 9606.ENSP00000381607 9606.ENSP00000380252 0 0 0 0 0 0 0 0.573 0.573

GSTP1 PARK7 9606.ENSP00000381607 9606.ENSP00000418770 0 0 0 0 0.09 0.129 0 0.317 0.411

GSTP1 TXNRD1 9606.ENSP00000381607 9606.ENSP00000434516 0 0 0 0 0.081 0 0 0.495 0.516

GSTP1 SOD2 9606.ENSP00000381607 9606.ENSP00000446252 0 0 0 0 0.065 0 0 0.575 0.586

GSTP1 CDKN2A 9606.ENSP00000381607 9606.ENSP00000418915 0 0 0 0 0.062 0.091 0 0.681 0.704

GSTP1 GPX1 9606.ENSP00000381607 9606.ENSP00000407375 0 0 0 0 0.156 0 0.65 0.626 0.88

GSTT2B GSS 9606.ENSP00000290765 9606.ENSP00000216951 0 0 0 0 0 0.065 0.65 0.354 0.77

GSTT2B GSR 9606.ENSP00000290765 9606.ENSP00000221130 0 0 0 0 0.112 0.061 0.65 0.436 0.813

GSTT2B GCLC 9606.ENSP00000290765 9606.ENSP00000229416 0 0 0 0 0.082 0.152 0 0.431 0.518

GSTT2B CAT 9606.ENSP00000290765 9606.ENSP00000241052 0 0 0 0 0.123 0 0 0.363 0.418

GSTT2B GSTM3 9606.ENSP00000290765 9606.ENSP00000256594 0 0 0 0 0.065 0.147 0.65 0.671 0.896

GSTT2B NQO1 9606.ENSP00000290765 9606.ENSP00000319788 0 0 0 0 0 0 0 0.405 0.405

GSTT2B GCLM 9606.ENSP00000290765 9606.ENSP00000359258 0 0 0 0 0.063 0.156 0 0.327 0.422

GSTT2B TXNRD1 9606.ENSP00000290765 9606.ENSP00000434516 0 0 0 0 0.112 0.174 0 0.375 0.502

GSTT2B GPX4 9606.ENSP00000290765 9606.ENSP00000346103 0 0 0 0 0.065 0 0.65 0.297 0.749

GSTT2B PRDX6 9606.ENSP00000290765 9606.ENSP00000342026 0 0 0 0 0.108 0.078 0.65 0.267 0.761

GSTT2B GPX1 9606.ENSP00000290765 9606.ENSP00000407375 0 0 0 0 0.065 0 0.65 0.335 0.763

GSTT2B GPX3 9606.ENSP00000290765 9606.ENSP00000373477 0 0 0 0 0.065 0 0.65 0.334 0.763

GSTT2B CYP1A1 9606.ENSP00000290765 9606.ENSP00000369050 0 0 0 0 0.062 0 0.65 0.416 0.791

GSTT2B MGST1 9606.ENSP00000290765 9606.ENSP00000379512 0 0 0 0 0.062 0 0.65 0.484 0.816

GSTT2B GSTA1 9606.ENSP00000290765 9606.ENSP00000335620 0 0 0 0 0.065 0.147 0.65 0.667 0.894

GSTT2B GSTM1 9606.ENSP00000290765 9606.ENSP00000311469 0 0 0 0 0.065 0.147 0.65 0.666 0.894

GSTT2B GSTP1 9606.ENSP00000290765 9606.ENSP00000381607 0 0 0 0 0.065 0.352 0.65 0.634 0.912

HAO1 DAO 9606.ENSP00000368066 9606.ENSP00000228476 0 0 0 0 0.14 0 0.9 0.505 0.953

HAO1 LOX 9606.ENSP00000368066 9606.ENSP00000231004 0 0 0 0 0 0 0 0.46 0.459

HAO1 CAT 9606.ENSP00000368066 9606.ENSP00000241052 0 0 0 0 0.112 0 0.9 0.811 0.981

HAO1 PAOX 9606.ENSP00000368066 9606.ENSP00000278060 0 0 0 0 0.06 0 0.9 0.289 0.927

HAO1 ACOX1 9606.ENSP00000368066 9606.ENSP00000293217 0.045 0 0 0 0.085 0 0.9 0.391 0.939

HAO1 PIPOX 9606.ENSP00000368066 9606.ENSP00000317721 0 0 0 0 0.185 0 0.9 0.689 0.972

HAO1 NOS2 9606.ENSP00000368066 9606.ENSP00000327251 0.05 0 0 0 0.065 0 0.9 0.062 0.905

HAO1 ACOX3 9606.ENSP00000368066 9606.ENSP00000348775 0.045 0 0 0 0.064 0 0.9 0.542 0.953

HAO1 DDO 9606.ENSP00000368066 9606.ENSP00000357920 0 0 0 0 0.108 0 0.9 0.276 0.929

HAO1 SMOX 9606.ENSP00000368066 9606.ENSP00000478305 0 0 0 0 0 0 0 0.608 0.608

HAO1 HAO2 9606.ENSP00000368066 9606.ENSP00000483507 0 0 0.449 0.939 0.299 0 0.9 0.955 0.932

HAO2 DAO 9606.ENSP00000483507 9606.ENSP00000228476 0 0 0 0 0.167 0 0.9 0.45 0.95

HAO2 CAT 9606.ENSP00000483507 9606.ENSP00000241052 0 0 0 0 0.112 0 0.9 0.32 0.934

HAO2 PAOX 9606.ENSP00000483507 9606.ENSP00000278060 0 0 0 0 0 0 0.9 0.147 0.911

HAO2 ACOX1 9606.ENSP00000483507 9606.ENSP00000293217 0.045 0 0 0 0.085 0 0.9 0.567 0.957

HAO2 PIPOX 9606.ENSP00000483507 9606.ENSP00000317721 0 0 0 0 0.515 0 0.9 0.7 0.984

HAO2 NOS2 9606.ENSP00000483507 9606.ENSP00000327251 0.05 0 0 0 0.065 0 0.9 0.105 0.909

HAO2 ACOX3 9606.ENSP00000483507 9606.ENSP00000348775 0.045 0 0 0 0.062 0 0.9 0.518 0.951

HAO2 APOA4 9606.ENSP00000483507 9606.ENSP00000350425 0 0 0 0 0.536 0 0 0.114 0.572

HAO2 DDO 9606.ENSP00000483507 9606.ENSP00000357920 0 0 0 0 0.108 0 0.9 0.348 0.936

HAO2 HAO1 9606.ENSP00000483507 9606.ENSP00000368066 0 0 0.449 0.939 0.299 0 0.9 0.955 0.932

HMOX1 LOX 9606.ENSP00000216117 9606.ENSP00000231004 0 0 0 0 0.062 0 0 0.39 0.403

HMOX1 MMP3 9606.ENSP00000216117 9606.ENSP00000299855 0 0 0 0 0 0 0 0.413 0.413

HMOX1 MMP1 9606.ENSP00000216117 9606.ENSP00000322788 0 0 0 0 0.068 0 0 0.415 0.431

HMOX1 NCF2 9606.ENSP00000216117 9606.ENSP00000356505 0 0 0 0 0.088 0 0 0.413 0.441

HMOX1 UCP2 9606.ENSP00000216117 9606.ENSP00000312029 0 0 0 0 0 0 0 0.45 0.45

HMOX1 PARK7 9606.ENSP00000216117 9606.ENSP00000418770 0 0 0 0 0.062 0 0 0.459 0.47

HMOX1 DUOX2 9606.ENSP00000216117 9606.ENSP00000475084 0 0 0 0 0 0 0 0.489 0.489

HMOX1 CYBA 9606.ENSP00000216117 9606.ENSP00000261623 0 0 0 0 0.091 0 0 0.468 0.495

HMOX1 DUOX1 9606.ENSP00000216117 9606.ENSP00000317997 0 0 0 0 0 0 0 0.5 0.5

HMOX1 CTH 9606.ENSP00000216117 9606.ENSP00000359976 0 0 0 0 0 0 0 0.518 0.518

HMOX1 GSTM1 9606.ENSP00000216117 9606.ENSP00000311469 0 0 0 0 0 0 0 0.552 0.552

HMOX1 GSTP1 9606.ENSP00000216117 9606.ENSP00000381607 0 0 0 0 0 0 0 0.556 0.556

HMOX1 CYP1A1 9606.ENSP00000216117 9606.ENSP00000369050 0 0 0 0 0.066 0 0 0.567 0.578

HMOX1 NOX1 9606.ENSP00000216117 9606.ENSP00000362057 0 0 0 0 0 0 0 0.586 0.586

HMOX1 GSTA1 9606.ENSP00000216117 9606.ENSP00000335620 0 0 0 0 0 0 0 0.589 0.589

HMOX1 GSTM3 9606.ENSP00000216117 9606.ENSP00000256594 0 0 0 0 0 0 0 0.597 0.597

HMOX1 MMP9 9606.ENSP00000216117 9606.ENSP00000361405 0 0 0 0 0.088 0 0 0.619 0.637

HMOX1 NOX4 9606.ENSP00000216117 9606.ENSP00000263317 0 0 0 0 0 0 0 0.645 0.645

HMOX1 MPO 9606.ENSP00000216117 9606.ENSP00000225275 0 0 0 0 0 0 0 0.652 0.652

HMOX1 SRXN1 9606.ENSP00000216117 9606.ENSP00000371388 0 0 0 0 0.107 0 0 0.631 0.657

HMOX1 CYBB 9606.ENSP00000216117 9606.ENSP00000367851 0 0 0 0 0.069 0 0 0.677 0.687

HMOX1 GPX3 9606.ENSP00000216117 9606.ENSP00000373477 0 0 0 0 0 0.074 0 0.68 0.691

HMOX1 SOD1 9606.ENSP00000216117 9606.ENSP00000270142 0 0 0 0 0 0 0 0.708 0.708

HMOX1 TXN 9606.ENSP00000216117 9606.ENSP00000363641 0 0 0 0 0 0 0 0.709 0.709

HMOX1 NOS2 9606.ENSP00000216117 9606.ENSP00000327251 0 0 0 0 0 0.177 0 0.667 0.714

HMOX1 PPARGC1A 9606.ENSP00000216117 9606.ENSP00000264867 0 0 0 0 0 0 0 0.719 0.719

HMOX1 SOD2 9606.ENSP00000216117 9606.ENSP00000446252 0.071 0 0 0 0.064 0 0 0.722 0.737

HMOX1 TXNRD1 9606.ENSP00000216117 9606.ENSP00000434516 0 0 0 0 0.066 0.128 0 0.706 0.739

HMOX1 GPX1 9606.ENSP00000216117 9606.ENSP00000407375 0 0 0 0 0.082 0.074 0 0.729 0.75

HMOX1 SOD3 9606.ENSP00000216117 9606.ENSP00000371554 0 0 0 0 0 0 0 0.759 0.76

HMOX1 GSR 9606.ENSP00000216117 9606.ENSP00000221130 0 0 0 0 0.083 0.098 0 0.742 0.768

HMOX1 NOS1 9606.ENSP00000216117 9606.ENSP00000477999 0 0 0 0 0 0.324 0 0.672 0.769

HMOX1 NOS3 9606.ENSP00000216117 9606.ENSP00000297494 0 0 0 0 0 0.177 0 0.752 0.787

HMOX1 GPX4 9606.ENSP00000216117 9606.ENSP00000346103 0 0 0 0 0.062 0.074 0 0.775 0.788

HMOX1 GCLM 9606.ENSP00000216117 9606.ENSP00000359258 0 0 0 0 0.053 0 0 0.855 0.857

HMOX1 CAT 9606.ENSP00000216117 9606.ENSP00000241052 0 0 0 0 0.062 0.101 0 0.882 0.892

HMOX1 GCLC 9606.ENSP00000216117 9606.ENSP00000229416 0 0 0 0 0 0.104 0 0.889 0.896

HMOX1 HMOX2 9606.ENSP00000216117 9606.ENSP00000477572 0 0 0.448 0.927 0 0 0.9 0.933 0.909

HMOX1 NQO1 9606.ENSP00000216117 9606.ENSP00000319788 0 0 0 0 0.058 0 0 0.915 0.916

HMOX1 MAPK14 9606.ENSP00000216117 9606.ENSP00000229795 0 0 0 0 0 0 0.9 0.407 0.938

HMOX1 FOS 9606.ENSP00000216117 9606.ENSP00000306245 0 0 0 0 0 0 0.9 0.517 0.949

HMOX1 NFE2L2 9606.ENSP00000216117 9606.ENSP00000380252 0 0 0 0 0 0 0.9 0.916 0.991

HMOX2 HMOX1 9606.ENSP00000477572 9606.ENSP00000216117 0 0 0.448 0.927 0 0 0.9 0.933 0.909

HMOX2 PON2 9606.ENSP00000477572 9606.ENSP00000222572 0 0 0 0 0 0 0 0.494 0.494

HMOX2 GCLC 9606.ENSP00000477572 9606.ENSP00000229416 0 0 0 0 0 0.104 0 0.645 0.668

HMOX2 CAT 9606.ENSP00000477572 9606.ENSP00000241052 0 0 0 0 0.062 0.101 0 0.636 0.666

HMOX2 NOS3 9606.ENSP00000477572 9606.ENSP00000297494 0 0 0 0 0 0.177 0 0.581 0.64

HMOX2 FOS 9606.ENSP00000477572 9606.ENSP00000306245 0 0 0 0 0 0 0.9 0.185 0.915

HMOX2 NQO1 9606.ENSP00000477572 9606.ENSP00000319788 0 0 0 0 0.058 0 0 0.5 0.509

HMOX2 NOS2 9606.ENSP00000477572 9606.ENSP00000327251 0 0 0 0 0 0.177 0 0.393 0.479

HMOX2 GPX4 9606.ENSP00000477572 9606.ENSP00000346103 0 0 0 0 0.062 0.074 0 0.488 0.516

HMOX2 GCLM 9606.ENSP00000477572 9606.ENSP00000359258 0 0 0 0 0.053 0 0 0.555 0.561

HMOX2 CTH 9606.ENSP00000477572 9606.ENSP00000359976 0 0 0 0 0 0 0 0.574 0.574

HMOX2 CYBB 9606.ENSP00000477572 9606.ENSP00000367851 0 0 0 0 0 0 0 0.417 0.417

HMOX2 SOD3 9606.ENSP00000477572 9606.ENSP00000371554 0 0 0 0 0 0 0 0.488 0.488

HMOX2 NFE2L2 9606.ENSP00000477572 9606.ENSP00000380252 0 0 0 0 0 0 0.9 0.524 0.95

HMOX2 NOS1 9606.ENSP00000477572 9606.ENSP00000477999 0 0 0 0 0 0.177 0 0.583 0.642

IL4I1 DAO 9606.ENSP00000472474 9606.ENSP00000228476 0.07 0 0 0 0.062 0 0 0.55 0.573

IL4I1 CAT 9606.ENSP00000472474 9606.ENSP00000241052 0 0 0 0 0.062 0 0 0.46 0.472

IL4I1 NQO1 9606.ENSP00000472474 9606.ENSP00000319788 0.048 0 0 0 0 0 0 0.562 0.565

IL4I1 MAOA 9606.ENSP00000472474 9606.ENSP00000340684 0 0 0 0.589 0 0 0.9 0.186 0.906

IL4I1 DDO 9606.ENSP00000472474 9606.ENSP00000357920 0.07 0 0 0 0.062 0 0 0.466 0.493

IL4I1 MAOB 9606.ENSP00000472474 9606.ENSP00000367309 0 0 0 0.603 0 0 0.9 0.156 0.904

IL4I1 AOC1 9606.ENSP00000472474 9606.ENSP00000411613 0 0 0 0 0.055 0 0.9 0.063 0.903

IPCEF1 CDKN2A 9606.ENSP00000394751 9606.ENSP00000418915 0 0 0 0 0 0.078 0 0.428 0.451

JUNB MAPK14 9606.ENSP00000303315 9606.ENSP00000229795 0 0 0 0 0 0.304 0.6 0.169 0.748

JUNB DUSP1 9606.ENSP00000303315 9606.ENSP00000239223 0 0 0 0 0.658 0.145 0 0.299 0.777

JUNB NCF2 9606.ENSP00000303315 9606.ENSP00000356505 0 0 0 0 0.099 0 0 0.394 0.43

JUNB MAPK10 9606.ENSP00000303315 9606.ENSP00000352157 0 0 0 0 0.062 0.344 0.6 0.282 0.799

JUNB SP1 9606.ENSP00000303315 9606.ENSP00000329357 0 0 0 0 0.046 0.101 0.9 0.29 0.93

JUNB FOS 9606.ENSP00000303315 9606.ENSP00000306245 0 0 0 0 0.826 0.899 0.9 0.986 0.999

LOX HMOX1 9606.ENSP00000231004 9606.ENSP00000216117 0 0 0 0 0.062 0 0 0.39 0.403

LOX COL1A1 9606.ENSP00000231004 9606.ENSP00000225964 0 0 0 0 0.409 0.192 0 0.632 0.809

LOX FOS 9606.ENSP00000231004 9606.ENSP00000306245 0 0 0 0 0 0 0 0.406 0.406

LOX GPX4 9606.ENSP00000231004 9606.ENSP00000346103 0 0 0 0 0.062 0 0 0.395 0.408

LOX MMP13 9606.ENSP00000231004 9606.ENSP00000260302 0 0 0 0 0.063 0 0 0.397 0.411

LOX AOC2 9606.ENSP00000231004 9606.ENSP00000253799 0 0 0 0 0 0 0 0.423 0.422

LOX NOS3 9606.ENSP00000231004 9606.ENSP00000297494 0 0 0 0 0 0 0 0.424 0.424

LOX TIMP2 9606.ENSP00000231004 9606.ENSP00000262768 0 0 0 0 0.131 0 0 0.399 0.456

LOX HAO1 9606.ENSP00000231004 9606.ENSP00000368066 0 0 0 0 0 0 0 0.46 0.459

LOX CAT 9606.ENSP00000231004 9606.ENSP00000241052 0 0 0 0 0 0 0 0.469 0.469

LOX CDKN2A 9606.ENSP00000231004 9606.ENSP00000418915 0 0 0 0 0.089 0 0 0.466 0.492

LOX TIMP3 9606.ENSP00000231004 9606.ENSP00000266085 0 0 0 0 0.214 0 0 0.394 0.503

LOX ATOX1 9606.ENSP00000231004 9606.ENSP00000430598 0 0 0 0 0 0.168 0 0.43 0.506

LOX MMP14 9606.ENSP00000231004 9606.ENSP00000308208 0 0 0 0 0.232 0 0 0.394 0.515

LOX MMP1 9606.ENSP00000231004 9606.ENSP00000322788 0 0 0 0 0.194 0 0 0.435 0.525

LOX ADAMTS2 9606.ENSP00000231004 9606.ENSP00000251582 0 0 0 0 0.17 0.064 0 0.439 0.526

LOX MMP3 9606.ENSP00000231004 9606.ENSP00000299855 0 0 0 0 0.17 0 0 0.475 0.546

LOX MMP9 9606.ENSP00000231004 9606.ENSP00000361405 0 0 0 0 0.062 0 0 0.607 0.616

MAOA DRD4 9606.ENSP00000340684 9606.ENSP00000176183 0 0 0 0 0 0 0 0.831 0.831

MAOA CAT 9606.ENSP00000340684 9606.ENSP00000241052 0 0 0 0 0.062 0 0 0.446 0.459

MAOA AOC2 9606.ENSP00000340684 9606.ENSP00000253799 0 0 0 0 0.055 0 0.8 0.763 0.951

MAOA DRD5 9606.ENSP00000340684 9606.ENSP00000306129 0 0 0 0 0 0 0 0.61 0.61

MAOA AOC3 9606.ENSP00000340684 9606.ENSP00000312326 0 0 0 0 0.082 0 0.8 0.557 0.911

MAOA SMOX 9606.ENSP00000340684 9606.ENSP00000478305 0 0 0 0 0 0 0 0.463 0.464

MAOA DRD1 9606.ENSP00000340684 9606.ENSP00000377353 0 0 0 0 0 0 0 0.614 0.614

MAOA DRD3 9606.ENSP00000340684 9606.ENSP00000373169 0 0 0 0 0 0 0 0.665 0.665

MAOA DRD2 9606.ENSP00000340684 9606.ENSP00000354859 0 0 0 0 0 0 0 0.756 0.757

MAOA MAOB 9606.ENSP00000340684 9606.ENSP00000367309 0 0 0.448 0.976 0.18 0.307 0.8 0.918 0.88

MAOA AOC1 9606.ENSP00000340684 9606.ENSP00000411613 0 0 0 0 0.057 0 0.8 0.502 0.897

MAOA IL4I1 9606.ENSP00000340684 9606.ENSP00000472474 0 0 0 0.589 0 0 0.9 0.186 0.906

MAOA AOX1 9606.ENSP00000340684 9606.ENSP00000363832 0 0 0 0 0.096 0 0.9 0.336 0.934

MAOB DRD4 9606.ENSP00000367309 9606.ENSP00000176183 0 0 0 0 0 0 0 0.575 0.575

MAOB CAT 9606.ENSP00000367309 9606.ENSP00000241052 0 0 0 0 0.062 0 0 0.503 0.514

MAOB AOC2 9606.ENSP00000367309 9606.ENSP00000253799 0 0 0 0 0.055 0 0.8 0.757 0.95

MAOB DRD5 9606.ENSP00000367309 9606.ENSP00000306129 0 0 0 0 0.067 0 0 0.504 0.517

MAOB AOC3 9606.ENSP00000367309 9606.ENSP00000312326 0 0 0 0 0.089 0 0.8 0.556 0.912

MAOB MAOA 9606.ENSP00000367309 9606.ENSP00000340684 0 0 0.448 0.976 0.18 0.307 0.8 0.918 0.88

MAOB DRD2 9606.ENSP00000367309 9606.ENSP00000354859 0 0 0 0 0.063 0 0 0.611 0.62

MAOB AOX1 9606.ENSP00000367309 9606.ENSP00000363832 0 0 0 0 0.062 0 0.9 0.316 0.93

MAOB PARK7 9606.ENSP00000367309 9606.ENSP00000418770 0.06 0 0 0 0 0 0 0.525 0.534

MAOB DRD3 9606.ENSP00000367309 9606.ENSP00000373169 0 0 0 0 0 0 0 0.56 0.56

MAOB DRD1 9606.ENSP00000367309 9606.ENSP00000377353 0 0 0 0 0.123 0 0 0.525 0.566

MAOB IL4I1 9606.ENSP00000367309 9606.ENSP00000472474 0 0 0 0.603 0 0 0.9 0.156 0.904

MAOB AOC1 9606.ENSP00000367309 9606.ENSP00000411613 0 0 0 0 0.057 0 0.8 0.548 0.907

MAPK10 NFKB1 9606.ENSP00000352157 9606.ENSP00000226574 0 0 0 0 0 0.102 0.8 0.28 0.859

MAPK10 MAPK14 9606.ENSP00000352157 9606.ENSP00000229795 0 0 0.347 0.921 0.066 0.056 0.6 0.711 0.646

MAPK10 DUSP1 9606.ENSP00000352157 9606.ENSP00000239223 0 0 0 0 0.062 0.356 0.8 0.374 0.914

MAPK10 JUNB 9606.ENSP00000352157 9606.ENSP00000303315 0 0 0 0 0.062 0.344 0.6 0.282 0.799

MAPK10 FOS 9606.ENSP00000352157 9606.ENSP00000306245 0 0 0 0 0 0.243 0.8 0.479 0.914

MAPK10 SP1 9606.ENSP00000352157 9606.ENSP00000329357 0 0 0 0 0 0.062 0.9 0.094 0.907

MAPK10 CDKN2A 9606.ENSP00000352157 9606.ENSP00000418915 0 0 0 0 0 0.316 0 0.7 0.786

MAPK14 HMOX1 9606.ENSP00000229795 9606.ENSP00000216117 0 0 0 0 0 0 0.9 0.407 0.938

MAPK14 NFKB1 9606.ENSP00000229795 9606.ENSP00000226574 0 0 0 0 0.065 0.134 0.9 0.463 0.95

MAPK14 RUNX2 9606.ENSP00000229795 9606.ENSP00000360493 0 0 0 0 0 0.106 0 0.37 0.412

MAPK14 MMP13 9606.ENSP00000229795 9606.ENSP00000260302 0 0 0 0 0.049 0 0 0.427 0.432

MAPK14 TXN 9606.ENSP00000229795 9606.ENSP00000363641 0 0 0 0 0.053 0.076 0 0.416 0.445

MAPK14 NOX4 9606.ENSP00000229795 9606.ENSP00000263317 0 0 0 0 0 0.07 0 0.431 0.448

MAPK14 NOS3 9606.ENSP00000229795 9606.ENSP00000297494 0 0 0 0 0.049 0 0 0.46 0.465

MAPK14 SOD2 9606.ENSP00000229795 9606.ENSP00000446252 0 0 0 0 0.062 0.132 0 0.412 0.48

MAPK14 NOS2 9606.ENSP00000229795 9606.ENSP00000327251 0 0 0 0 0.049 0 0 0.496 0.5

MAPK14 CAT 9606.ENSP00000229795 9606.ENSP00000241052 0 0 0 0 0 0.117 0 0.507 0.546

MAPK14 MMP9 9606.ENSP00000229795 9606.ENSP00000361405 0 0 0 0 0.049 0 0 0.567 0.571

MAPK14 MAPK10 9606.ENSP00000229795 9606.ENSP00000352157 0 0 0.347 0.921 0.066 0.056 0.6 0.711 0.646

MAPK14 JUNB 9606.ENSP00000229795 9606.ENSP00000303315 0 0 0 0 0 0.304 0.6 0.169 0.748

MAPK14 CYBA 9606.ENSP00000229795 9606.ENSP00000261623 0 0 0 0 0 0 0.65 0.335 0.757

MAPK14 CYBB 9606.ENSP00000229795 9606.ENSP00000367851 0 0 0 0 0 0.078 0.65 0.43 0.8

MAPK14 CDKN2A 9606.ENSP00000229795 9606.ENSP00000418915 0 0 0 0 0 0.259 0.8 0.324 0.891

MAPK14 NCF2 9606.ENSP00000229795 9606.ENSP00000356505 0 0 0 0 0.062 0 0.9 0.329 0.931

MAPK14 SP1 9606.ENSP00000229795 9606.ENSP00000329357 0 0 0 0 0 0.34 0.9 0.204 0.942

MAPK14 PPARGC1A 9606.ENSP00000229795 9606.ENSP00000264867 0 0 0 0 0 0.213 0.9 0.457 0.953

MAPK14 FOS 9606.ENSP00000229795 9606.ENSP00000306245 0 0 0 0 0 0.248 0.9 0.592 0.966

MAPK14 DUSP1 9606.ENSP00000229795 9606.ENSP00000239223 0 0 0 0 0 0.9 0.9 0.887 0.998

MGST1 GSS 9606.ENSP00000379512 9606.ENSP00000216951 0 0 0 0 0.062 0 0.65 0.181 0.707

MGST1 GSR 9606.ENSP00000379512 9606.ENSP00000221130 0 0 0 0 0.062 0 0.65 0.483 0.815

MGST1 GCLC 9606.ENSP00000379512 9606.ENSP00000229416 0 0 0 0 0.062 0 0 0.414 0.427

MGST1 GSTM3 9606.ENSP00000379512 9606.ENSP00000256594 0 0 0 0 0.062 0 0.65 0.535 0.834

MGST1 SOD1 9606.ENSP00000379512 9606.ENSP00000270142 0 0 0 0 0.089 0 0 0.424 0.453

MGST1 GSTT2B 9606.ENSP00000379512 9606.ENSP00000290765 0 0 0 0 0.062 0 0.65 0.484 0.816

MGST1 GSTM1 9606.ENSP00000379512 9606.ENSP00000311469 0 0 0 0 0.062 0 0.65 0.465 0.809

MGST1 NQO1 9606.ENSP00000379512 9606.ENSP00000319788 0 0 0 0 0.088 0 0 0.37 0.4

MGST1 GSTA1 9606.ENSP00000379512 9606.ENSP00000335620 0 0 0 0 0.064 0 0.65 0.515 0.827

MGST1 PRDX6 9606.ENSP00000379512 9606.ENSP00000342026 0 0 0 0 0.049 0 0.65 0.308 0.749

MGST1 GPX4 9606.ENSP00000379512 9606.ENSP00000346103 0 0 0 0 0.062 0 0.65 0.258 0.735

MGST1 GCLM 9606.ENSP00000379512 9606.ENSP00000359258 0 0 0 0 0.062 0 0 0.415 0.427

MGST1 CYP1A1 9606.ENSP00000379512 9606.ENSP00000369050 0 0 0 0 0.062 0 0.65 0.281 0.743

MGST1 GPX3 9606.ENSP00000379512 9606.ENSP00000373477 0 0 0 0 0.062 0 0.65 0.293 0.747

MGST1 TXNRD1 9606.ENSP00000379512 9606.ENSP00000434516 0 0 0 0 0 0 0 0.448 0.448

MGST1 GPX1 9606.ENSP00000379512 9606.ENSP00000407375 0 0 0 0 0.064 0 0.65 0.392 0.783

MGST1 GSTP1 9606.ENSP00000379512 9606.ENSP00000381607 0 0 0 0 0.062 0 0.65 0.509 0.824

MMP1 HMOX1 9606.ENSP00000322788 9606.ENSP00000216117 0 0 0 0 0.068 0 0 0.415 0.431

MMP1 MPO 9606.ENSP00000322788 9606.ENSP00000225275 0 0 0 0 0 0 0 0.405 0.405

MMP1 COL1A1 9606.ENSP00000322788 9606.ENSP00000225964 0 0 0 0 0.128 0.059 0 0.611 0.653

MMP1 LOX 9606.ENSP00000322788 9606.ENSP00000231004 0 0 0 0 0.194 0 0 0.435 0.525

MMP1 MMP13 9606.ENSP00000322788 9606.ENSP00000260302 0 0 0 0.935 0.078 0 0.65 0.871 0.682

MMP1 TIMP2 9606.ENSP00000322788 9606.ENSP00000262768 0 0 0 0 0.098 0.18 0 0.937 0.95

MMP1 TIMP3 9606.ENSP00000322788 9606.ENSP00000266085 0 0 0 0 0.088 0.18 0 0.818 0.852

MMP1 MMP10 9606.ENSP00000322788 9606.ENSP00000279441 0 0 0.28 0.948 0.361 0 0.9 0.764 0.936

MMP1 TIMP4 9606.ENSP00000322788 9606.ENSP00000287814 0 0 0 0 0 0.18 0 0.659 0.708

MMP1 MMP3 9606.ENSP00000322788 9606.ENSP00000299855 0 0 0.298 0.953 0.529 0 0.9 0.912 0.953

MMP1 FOS 9606.ENSP00000322788 9606.ENSP00000306245 0 0 0 0 0 0.27 0 0.561 0.666

MMP1 COL2A1 9606.ENSP00000322788 9606.ENSP00000369889 0 0 0 0 0 0.227 0 0.477 0.579

MMP1 MMP9 9606.ENSP00000322788 9606.ENSP00000361405 0 0 0 0.75 0.518 0 0.9 0.901 0.961

MMP1 ACAN 9606.ENSP00000322788 9606.ENSP00000387356 0 0 0 0 0 0.228 0.9 0.697 0.974

MMP10 COL1A1 9606.ENSP00000279441 9606.ENSP00000225964 0 0 0 0 0.065 0.059 0 0.402 0.428

MMP10 MMP13 9606.ENSP00000279441 9606.ENSP00000260302 0 0 0.236 0.941 0.086 0 0.65 0.766 0.685

MMP10 TIMP2 9606.ENSP00000279441 9606.ENSP00000262768 0 0 0 0 0.064 0.828 0 0.816 0.968

MMP10 TIMP3 9606.ENSP00000279441 9606.ENSP00000266085 0 0 0 0 0 0.18 0 0.629 0.682

MMP10 SP1 9606.ENSP00000279441 9606.ENSP00000329357 0 0 0 0 0 0 0 0.416 0.416

MMP10 ACAN 9606.ENSP00000279441 9606.ENSP00000387356 0 0 0 0 0 0.06 0 0.621 0.629

MMP10 TIMP4 9606.ENSP00000279441 9606.ENSP00000287814 0 0 0 0 0 0.18 0 0.587 0.647

MMP10 MMP3 9606.ENSP00000279441 9606.ENSP00000299855 0 0 0.448 0.98 0.311 0.738 0.65 0.825 0.933

MMP10 MMP1 9606.ENSP00000279441 9606.ENSP00000322788 0 0 0.28 0.948 0.361 0 0.9 0.764 0.936

MMP10 MMP9 9606.ENSP00000279441 9606.ENSP00000361405 0 0 0 0.786 0.53 0.213 0.9 0.836 0.966

MMP13 COL1A1 9606.ENSP00000260302 9606.ENSP00000225964 0 0 0 0 0.062 0.059 0 0.686 0.699

MMP13 MAPK14 9606.ENSP00000260302 9606.ENSP00000229795 0 0 0 0 0.049 0 0 0.427 0.432

MMP13 LOX 9606.ENSP00000260302 9606.ENSP00000231004 0 0 0 0 0.063 0 0 0.397 0.411

MMP13 NOS2 9606.ENSP00000260302 9606.ENSP00000327251 0 0 0 0 0.062 0 0 0.398 0.411

MMP13 MMP9 9606.ENSP00000260302 9606.ENSP00000361405 0 0 0 0.797 0.065 0 0.3 0.865 0.436

MMP13 FOS 9606.ENSP00000260302 9606.ENSP00000306245 0 0 0 0 0 0 0 0.468 0.468

MMP13 MMP1 9606.ENSP00000260302 9606.ENSP00000322788 0 0 0 0.935 0.078 0 0.65 0.871 0.682

MMP13 MMP10 9606.ENSP00000260302 9606.ENSP00000279441 0 0 0.236 0.941 0.086 0 0.65 0.766 0.685

MMP13 TIMP4 9606.ENSP00000260302 9606.ENSP00000287814 0 0 0 0 0 0.18 0 0.64 0.692

MMP13 TIMP3 9606.ENSP00000260302 9606.ENSP00000266085 0 0 0 0 0 0.327 0 0.7 0.79

MMP13 COL2A1 9606.ENSP00000260302 9606.ENSP00000369889 0 0 0 0 0 0.227 0 0.833 0.865

MMP13 MMP14 9606.ENSP00000260302 9606.ENSP00000308208 0 0 0.241 0.854 0.062 0.213 0.9 0.798 0.931

MMP13 TIMP2 9606.ENSP00000260302 9606.ENSP00000262768 0 0 0 0 0 0.702 0 0.925 0.976

MMP13 RUNX2 9606.ENSP00000260302 9606.ENSP00000360493 0 0 0 0 0 0 0.9 0.779 0.976

MMP13 ACAN 9606.ENSP00000260302 9606.ENSP00000387356 0 0 0 0 0 0.06 0.9 0.883 0.988

MMP14 COL1A1 9606.ENSP00000308208 9606.ENSP00000225964 0 0 0 0 0.287 0.059 0 0.448 0.597

MMP14 LOX 9606.ENSP00000308208 9606.ENSP00000231004 0 0 0 0 0.232 0 0 0.394 0.515

MMP14 MMP13 9606.ENSP00000308208 9606.ENSP00000260302 0 0 0.241 0.854 0.062 0.213 0.9 0.798 0.931

MMP14 TIMP2 9606.ENSP00000308208 9606.ENSP00000262768 0 0 0 0 0.153 0.884 0.9 0.99 0.999

MMP14 TIMP3 9606.ENSP00000308208 9606.ENSP00000266085 0 0 0 0 0.183 0.383 0 0.784 0.881

MMP14 TIMP4 9606.ENSP00000308208 9606.ENSP00000287814 0 0 0 0 0 0.327 0 0.755 0.829

MMP14 MMP3 9606.ENSP00000308208 9606.ENSP00000299855 0 0 0.203 0.853 0.152 0.27 0 0.824 0.446

MMP14 NCF2 9606.ENSP00000308208 9606.ENSP00000356505 0 0 0 0 0 0 0 0.559 0.559

MMP14 ACAN 9606.ENSP00000308208 9606.ENSP00000387356 0 0 0 0 0 0.279 0 0.567 0.675

MMP19 TIMP2 9606.ENSP00000313437 9606.ENSP00000262768 0 0 0 0 0.074 0.18 0 0.481 0.571

MMP19 TIMP3 9606.ENSP00000313437 9606.ENSP00000266085 0 0 0 0 0.076 0.249 0 0.508 0.629

MMP19 TIMP4 9606.ENSP00000313437 9606.ENSP00000287814 0 0 0 0 0 0.18 0 0.43 0.512

MMP19 MMP3 9606.ENSP00000313437 9606.ENSP00000299855 0 0 0 0.776 0.088 0 0.3 0.563 0.415

MMP19 ACAN 9606.ENSP00000313437 9606.ENSP00000387356 0 0 0 0 0 0.262 0 0.348 0.498

MMP3 HMOX1 9606.ENSP00000299855 9606.ENSP00000216117 0 0 0 0 0 0 0 0.413 0.413

MMP3 MPO 9606.ENSP00000299855 9606.ENSP00000225275 0 0 0 0 0 0 0 0.455 0.455

MMP3 COL1A1 9606.ENSP00000299855 9606.ENSP00000225964 0 0 0 0 0.159 0.059 0 0.617 0.671

MMP3 LOX 9606.ENSP00000299855 9606.ENSP00000231004 0 0 0 0 0.17 0 0 0.475 0.546

MMP3 TIMP2 9606.ENSP00000299855 9606.ENSP00000262768 0 0 0 0 0.091 0.18 0 0.958 0.966

MMP3 TIMP3 9606.ENSP00000299855 9606.ENSP00000266085 0 0 0 0 0.088 0.568 0 0.837 0.93

MMP3 MMP10 9606.ENSP00000299855 9606.ENSP00000279441 0 0 0.448 0.98 0.311 0.738 0.65 0.825 0.933

MMP3 TIMP4 9606.ENSP00000299855 9606.ENSP00000287814 0 0 0 0 0 0.18 0 0.691 0.736

MMP3 MMP19 9606.ENSP00000299855 9606.ENSP00000313437 0 0 0 0.776 0.088 0 0.3 0.563 0.415

MMP3 APOA4 9606.ENSP00000299855 9606.ENSP00000350425 0 0 0 0 0.062 0 0 0.418 0.43

MMP3 MMP14 9606.ENSP00000299855 9606.ENSP00000308208 0 0 0.203 0.853 0.152 0.27 0 0.824 0.446

MMP3 FOS 9606.ENSP00000299855 9606.ENSP00000306245 0 0 0 0 0 0 0 0.473 0.473

MMP3 RUNX2 9606.ENSP00000299855 9606.ENSP00000360493 0 0 0 0 0 0 0 0.482 0.482

MMP3 COL2A1 9606.ENSP00000299855 9606.ENSP00000369889 0 0 0 0 0 0.059 0 0.651 0.657

MMP3 MMP9 9606.ENSP00000299855 9606.ENSP00000361405 0 0 0 0.791 0.518 0 0.8 0.903 0.918

MMP3 MMP1 9606.ENSP00000299855 9606.ENSP00000322788 0 0 0.298 0.953 0.529 0 0.9 0.912 0.953

MMP3 ACAN 9606.ENSP00000299855 9606.ENSP00000387356 0 0 0 0 0 0.228 0.9 0.835 0.986

MMP8 MPO 9606.ENSP00000236826 9606.ENSP00000225275 0 0 0 0 0.142 0 0 0.603 0.645

MMP8 COL1A1 9606.ENSP00000236826 9606.ENSP00000225964 0 0 0 0 0 0.059 0 0.455 0.466

MMP8 MMP9 9606.ENSP00000236826 9606.ENSP00000361405 0 0 0 0.738 0.546 0 0 0.888 0.651

MMP8 TIMP3 9606.ENSP00000236826 9606.ENSP00000266085 0 0 0 0 0 0.18 0 0.642 0.693

MMP8 ACAN 9606.ENSP00000236826 9606.ENSP00000387356 0 0 0 0 0 0.228 0 0.638 0.709

MMP8 TIMP4 9606.ENSP00000236826 9606.ENSP00000287814 0 0 0 0 0 0.18 0 0.688 0.733

MMP8 TIMP2 9606.ENSP00000236826 9606.ENSP00000262768 0 0 0 0 0.064 0.327 0 0.79 0.857

MMP9 HMOX1 9606.ENSP00000361405 9606.ENSP00000216117 0 0 0 0 0.088 0 0 0.619 0.637

MMP9 MPO 9606.ENSP00000361405 9606.ENSP00000225275 0 0 0 0 0.076 0 0 0.716 0.727

MMP9 COL1A1 9606.ENSP00000361405 9606.ENSP00000225964 0 0 0 0 0.077 0.227 0 0.639 0.72

MMP9 NFKB1 9606.ENSP00000361405 9606.ENSP00000226574 0 0 0 0 0.085 0 0 0.412 0.439

MMP9 MAPK14 9606.ENSP00000361405 9606.ENSP00000229795 0 0 0 0 0.049 0 0 0.567 0.571

MMP9 LOX 9606.ENSP00000361405 9606.ENSP00000231004 0 0 0 0 0.062 0 0 0.607 0.616

MMP9 MMP8 9606.ENSP00000361405 9606.ENSP00000236826 0 0 0 0.738 0.546 0 0 0.888 0.651

MMP9 CAT 9606.ENSP00000361405 9606.ENSP00000241052 0 0 0 0 0 0 0 0.566 0.566

MMP9 MMP13 9606.ENSP00000361405 9606.ENSP00000260302 0 0 0 0.797 0.065 0 0.3 0.865 0.436

MMP9 CYBA 9606.ENSP00000361405 9606.ENSP00000261623 0 0 0 0 0.108 0 0.65 0.342 0.776

MMP9 TIMP2 9606.ENSP00000361405 9606.ENSP00000262768 0 0 0 0 0.068 0.18 0 0.923 0.936

MMP9 NOX4 9606.ENSP00000361405 9606.ENSP00000263317 0 0 0 0 0 0 0 0.489 0.489

MMP9 TIMP3 9606.ENSP00000361405 9606.ENSP00000266085 0 0 0 0 0 0.327 0.8 0.914 0.987

MMP9 MMP10 9606.ENSP00000361405 9606.ENSP00000279441 0 0 0 0.786 0.53 0.213 0.9 0.836 0.966

MMP9 TIMP4 9606.ENSP00000361405 9606.ENSP00000287814 0 0 0 0 0 0.18 0 0.793 0.823

MMP9 NOS3 9606.ENSP00000361405 9606.ENSP00000297494 0 0 0 0 0.062 0 0 0.658 0.665

MMP9 MMP3 9606.ENSP00000361405 9606.ENSP00000299855 0 0 0 0.791 0.518 0 0.8 0.903 0.918

MMP9 FOS 9606.ENSP00000361405 9606.ENSP00000306245 0 0 0 0 0.062 0 0 0.656 0.664

MMP9 MMP1 9606.ENSP00000361405 9606.ENSP00000322788 0 0 0 0.75 0.518 0 0.9 0.901 0.961

MMP9 NOS2 9606.ENSP00000361405 9606.ENSP00000327251 0 0 0 0 0.065 0 0 0.535 0.547

MMP9 SP1 9606.ENSP00000361405 9606.ENSP00000329357 0 0 0 0 0 0 0 0.406 0.406

MMP9 NCF2 9606.ENSP00000361405 9606.ENSP00000356505 0 0 0 0 0.229 0 0.65 0.295 0.793

MMP9 S100A8 9606.ENSP00000361405 9606.ENSP00000357722 0 0 0 0 0.23 0 0 0.341 0.471

MMP9 S100A9 9606.ENSP00000361405 9606.ENSP00000357727 0 0 0 0 0.314 0 0 0.34 0.528

MMP9 RUNX2 9606.ENSP00000361405 9606.ENSP00000360493 0 0 0 0 0.062 0 0 0.581 0.59

MMP9 SOD2 9606.ENSP00000361405 9606.ENSP00000446252 0 0 0 0 0.069 0 0 0.395 0.413

MMP9 NFE2L2 9606.ENSP00000361405 9606.ENSP00000380252 0 0 0 0 0 0 0 0.425 0.424

MMP9 CDKN2A 9606.ENSP00000361405 9606.ENSP00000418915 0 0 0 0 0 0 0 0.505 0.505

MMP9 NOX1 9606.ENSP00000361405 9606.ENSP00000362057 0 0 0 0 0.062 0 0 0.521 0.532

MMP9 COL2A1 9606.ENSP00000361405 9606.ENSP00000369889 0 0 0 0 0 0.146 0 0.479 0.536

MMP9 CYBB 9606.ENSP00000361405 9606.ENSP00000367851 0 0 0 0 0.17 0 0.65 0.559 0.86

MMP9 ACAN 9606.ENSP00000361405 9606.ENSP00000387356 0 0 0 0 0 0.06 0.9 0.664 0.965

MPO HMOX1 9606.ENSP00000225275 9606.ENSP00000216117 0 0 0 0 0 0 0 0.652 0.652

MPO GSR 9606.ENSP00000225275 9606.ENSP00000221130 0 0 0 0 0 0.166 0 0.49 0.556

MPO PON1 9606.ENSP00000225275 9606.ENSP00000222381 0 0 0 0 0 0 0 0.923 0.923

MPO GPX3 9606.ENSP00000225275 9606.ENSP00000373477 0 0 0 0 0 0 0 0.4 0.4

MPO MMP1 9606.ENSP00000225275 9606.ENSP00000322788 0 0 0 0 0 0 0 0.405 0.405

MPO FOS 9606.ENSP00000225275 9606.ENSP00000306245 0 0 0 0 0 0 0 0.414 0.414

MPO GSTP1 9606.ENSP00000225275 9606.ENSP00000381607 0 0 0 0 0 0 0 0.421 0.42

MPO GPX4 9606.ENSP00000225275 9606.ENSP00000346103 0 0 0 0 0 0 0 0.433 0.433

MPO MMP3 9606.ENSP00000225275 9606.ENSP00000299855 0 0 0 0 0 0 0 0.455 0.455

MPO NOX4 9606.ENSP00000225275 9606.ENSP00000263317 0 0 0 0 0.049 0.062 0 0.441 0.457

MPO PRDX2 9606.ENSP00000225275 9606.ENSP00000301522 0 0 0 0 0 0 0 0.458 0.459

MPO GSTM1 9606.ENSP00000225275 9606.ENSP00000311469 0 0 0 0 0 0 0 0.467 0.467

MPO NFE2L2 9606.ENSP00000225275 9606.ENSP00000380252 0 0 0 0 0 0 0 0.47 0.47

MPO NOX1 9606.ENSP00000225275 9606.ENSP00000362057 0 0 0 0 0.049 0.062 0 0.471 0.487

MPO NQO1 9606.ENSP00000225275 9606.ENSP00000319788 0 0 0 0 0 0 0 0.505 0.505

MPO CYBA 9606.ENSP00000225275 9606.ENSP00000261623 0 0 0 0 0 0 0 0.512 0.512

MPO S100A9 9606.ENSP00000225275 9606.ENSP00000357727 0 0 0 0 0.231 0 0 0.394 0.514

MPO S100A8 9606.ENSP00000225275 9606.ENSP00000357722 0 0 0 0 0.232 0.128 0 0.341 0.52

MPO GPX1 9606.ENSP00000225275 9606.ENSP00000407375 0 0 0 0 0 0 0 0.53 0.53

MPO NCF2 9606.ENSP00000225275 9606.ENSP00000356505 0 0 0 0 0.147 0 0 0.488 0.545

MPO NOS2 9606.ENSP00000225275 9606.ENSP00000327251 0 0 0 0 0 0.09 0 0.535 0.559

MPO DAO 9606.ENSP00000225275 9606.ENSP00000228476 0 0 0 0 0.072 0.236 0 0.463 0.586

MPO NOS3 9606.ENSP00000225275 9606.ENSP00000297494 0 0 0 0 0 0.09 0 0.565 0.587

MPO MMP8 9606.ENSP00000225275 9606.ENSP00000236826 0 0 0 0 0.142 0 0 0.603 0.645

MPO MMP9 9606.ENSP00000225275 9606.ENSP00000361405 0 0 0 0 0.076 0 0 0.716 0.727

MPO CYBB 9606.ENSP00000225275 9606.ENSP00000367851 0 0 0 0 0.143 0.062 0 0.717 0.753

MPO CAT 9606.ENSP00000225275 9606.ENSP00000241052 0 0 0 0 0.061 0.241 0 0.702 0.769

MSRA TXN2 9606.ENSP00000313921 9606.ENSP00000216185 0.071 0 0 0 0.055 0.248 0 0.686 0.765

MSRA GSS 9606.ENSP00000313921 9606.ENSP00000216951 0 0 0 0 0 0 0 0.418 0.418

MSRA GSR 9606.ENSP00000313921 9606.ENSP00000221130 0.057 0 0 0 0.083 0 0 0.404 0.44

MSRA CAT 9606.ENSP00000313921 9606.ENSP00000241052 0 0 0 0 0.062 0.219 0 0.646 0.718

MSRA PRDX5 9606.ENSP00000313921 9606.ENSP00000265462 0.124 0 0 0 0.066 0 0 0.358 0.428

MSRA PRDX6 9606.ENSP00000313921 9606.ENSP00000342026 0.049 0 0 0 0 0 0 0.4 0.405

MSRA SUOX 9606.ENSP00000313921 9606.ENSP00000377668 0.186 0 0 0 0 0 0 0.338 0.438

MSRA MTHFR 9606.ENSP00000313921 9606.ENSP00000365777 0.237 0 0 0 0.06 0 0 0.355 0.497

MSRA TXNRD2 9606.ENSP00000313921 9606.ENSP00000383365 0.057 0 0 0 0 0 0 0.499 0.507

MSRA GLRX2 9606.ENSP00000313921 9606.ENSP00000356410 0.05 0 0 0 0 0.291 0 0.332 0.511

MSRA GPX3 9606.ENSP00000313921 9606.ENSP00000373477 0.072 0 0 0 0.062 0.247 0 0.372 0.533

MSRA GPX1 9606.ENSP00000313921 9606.ENSP00000407375 0.072 0 0 0 0.062 0.247 0 0.416 0.566

MSRA GPX4 9606.ENSP00000313921 9606.ENSP00000346103 0.072 0 0 0 0.062 0.247 0 0.49 0.621

MSRA VIMP 9606.ENSP00000313921 9606.ENSP00000381282 0 0 0 0 0 0 0 0.63 0.63

MSRA SOD2 9606.ENSP00000313921 9606.ENSP00000446252 0.295 0 0 0 0.064 0 0 0.499 0.64

MSRA CTH 9606.ENSP00000313921 9606.ENSP00000359976 0.282 0 0 0 0.185 0.13 0 0.617 0.779

MSRA TXNRD1 9606.ENSP00000313921 9606.ENSP00000434516 0.207 0 0 0 0 0.291 0 0.678 0.803

MSRA TXN 9606.ENSP00000313921 9606.ENSP00000363641 0.071 0 0 0 0.055 0.774 0.9 0.613 0.99

MT1X GSR 9606.ENSP00000377995 9606.ENSP00000221130 0 0 0 0 0 0 0 0.52 0.52

MT1X NQO1 9606.ENSP00000377995 9606.ENSP00000319788 0 0 0 0 0.054 0 0 0.5 0.506

MTHFR ERCC1 9606.ENSP00000365777 9606.ENSP00000013807 0 0 0 0 0 0 0 0.52 0.52

MTHFR DRD4 9606.ENSP00000365777 9606.ENSP00000176183 0 0 0 0 0 0 0 0.45 0.45

MTHFR GSR 9606.ENSP00000365777 9606.ENSP00000221130 0.163 0 0 0 0.062 0 0 0.358 0.452

MTHFR PON1 9606.ENSP00000365777 9606.ENSP00000222381 0 0 0 0 0 0 0 0.651 0.651

MTHFR PNPO 9606.ENSP00000365777 9606.ENSP00000225573 0.111 0 0 0 0.067 0 0 0.565 0.608

MTHFR DAO 9606.ENSP00000365777 9606.ENSP00000228476 0.049 0 0 0 0 0 0 0.415 0.42

MTHFR CAT 9606.ENSP00000365777 9606.ENSP00000241052 0.199 0 0 0 0.049 0 0 0.456 0.549

MTHFR SOD1 9606.ENSP00000365777 9606.ENSP00000270142 0.115 0 0 0 0 0.082 0 0.33 0.408

MTHFR NOS3 9606.ENSP00000365777 9606.ENSP00000297494 0.123 0 0 0 0.066 0.181 0 0.681 0.757

MTHFR GSTM1 9606.ENSP00000365777 9606.ENSP00000311469 0 0 0 0 0 0 0 0.699 0.699

MTHFR MSRA 9606.ENSP00000365777 9606.ENSP00000313921 0.237 0 0 0 0.06 0 0 0.355 0.497

MTHFR NQO1 9606.ENSP00000365777 9606.ENSP00000319788 0.056 0 0 0 0 0 0 0.454 0.462

MTHFR NOS2 9606.ENSP00000365777 9606.ENSP00000327251 0.123 0 0 0 0.066 0.181 0 0.256 0.434

MTHFR DRD2 9606.ENSP00000365777 9606.ENSP00000354859 0 0 0 0 0 0 0 0.455 0.455

MTHFR CTH 9606.ENSP00000365777 9606.ENSP00000359976 0.295 0 0 0 0.145 0.143 0 0.818 0.893

MTHFR SOD3 9606.ENSP00000365777 9606.ENSP00000371554 0.115 0 0 0 0 0.082 0 0.354 0.43

MTHFR NOS1 9606.ENSP00000365777 9606.ENSP00000477999 0.123 0 0 0 0.066 0.181 0 0.26 0.437

MTHFR CDKN2A 9606.ENSP00000365777 9606.ENSP00000418915 0 0 0 0 0 0.103 0 0.402 0.441

MTHFR TXNRD1 9606.ENSP00000365777 9606.ENSP00000434516 0.183 0 0 0 0.062 0 0 0.432 0.527

MTHFR CYP1A1 9606.ENSP00000365777 9606.ENSP00000369050 0 0 0 0 0.064 0 0 0.523 0.534

MTHFR SOD2 9606.ENSP00000365777 9606.ENSP00000446252 0.05 0 0 0 0.062 0.078 0 0.51 0.543

MTHFR ERCC2 9606.ENSP00000365777 9606.ENSP00000375809 0.069 0 0 0 0.062 0 0 0.519 0.543

MTHFR DGKK 9606.ENSP00000365777 9606.ENSP00000477515 0 0 0 0 0 0 0 0.66 0.66

MTHFR GSTP1 9606.ENSP00000365777 9606.ENSP00000381607 0 0 0 0 0 0 0 0.664 0.664

NCF2 NOX3 9606.ENSP00000356505 9606.ENSP00000159060 0 0 0 0 0.104 0 0.6 0.827 0.932

NCF2 HMOX1 9606.ENSP00000356505 9606.ENSP00000216117 0 0 0 0 0.088 0 0 0.413 0.441

NCF2 MPO 9606.ENSP00000356505 9606.ENSP00000225275 0 0 0 0 0.147 0 0 0.488 0.545

NCF2 MAPK14 9606.ENSP00000356505 9606.ENSP00000229795 0 0 0 0 0.062 0 0.9 0.329 0.931

NCF2 CAT 9606.ENSP00000356505 9606.ENSP00000241052 0 0 0 0 0.069 0 0 0.612 0.623

NCF2 CYBA 9606.ENSP00000356505 9606.ENSP00000261623 0 0 0 0 0.156 0.462 0.9 0.993 0.999

NCF2 NOX4 9606.ENSP00000356505 9606.ENSP00000263317 0 0 0 0 0.063 0 0.54 0.879 0.943

NCF2 NOS3 9606.ENSP00000356505 9606.ENSP00000297494 0 0 0 0 0 0 0 0.595 0.595

NCF2 JUNB 9606.ENSP00000356505 9606.ENSP00000303315 0 0 0 0 0.099 0 0 0.394 0.43

NCF2 MMP14 9606.ENSP00000356505 9606.ENSP00000308208 0 0 0 0 0 0 0 0.559 0.559

NCF2 DUOX1 9606.ENSP00000356505 9606.ENSP00000317997 0 0 0 0 0.065 0 0.54 0.873 0.94

NCF2 PRDX6 9606.ENSP00000356505 9606.ENSP00000342026 0 0 0 0 0 0.345 0 0.783 0.852

NCF2 GPX1 9606.ENSP00000356505 9606.ENSP00000407375 0 0 0 0 0 0 0 0.4 0.4

NCF2 SOD2 9606.ENSP00000356505 9606.ENSP00000446252 0 0 0 0 0.076 0 0 0.391 0.414

NCF2 SOD3 9606.ENSP00000356505 9606.ENSP00000371554 0 0 0 0 0 0 0 0.454 0.454

NCF2 MMP9 9606.ENSP00000356505 9606.ENSP00000361405 0 0 0 0 0.229 0 0.65 0.295 0.793

NCF2 DUOX2 9606.ENSP00000356505 9606.ENSP00000475084 0 0 0 0 0.065 0 0.54 0.713 0.866

NCF2 S100A9 9606.ENSP00000356505 9606.ENSP00000357727 0 0 0 0 0.564 0.27 0.6 0.145 0.876

NCF2 S100A8 9606.ENSP00000356505 9606.ENSP00000357722 0 0 0 0 0.563 0.27 0.6 0.451 0.92

NCF2 NOX5 9606.ENSP00000356505 9606.ENSP00000373518 0 0 0 0 0.063 0 0.54 0.871 0.939

NCF2 NOX1 9606.ENSP00000356505 9606.ENSP00000362057 0 0 0 0 0.104 0 0.8 0.966 0.993

NCF2 CYBB 9606.ENSP00000356505 9606.ENSP00000367851 0 0 0 0 0.382 0.462 0.9 0.992 0.999

NDUFA12 NDUFB4 9606.ENSP00000330737 9606.ENSP00000184266 0 0 0 0 0.879 0.987 0.9 0.532 0.999

NDUFA12 NDUFS8 9606.ENSP00000330737 9606.ENSP00000315774 0 0 0 0 0.82 0.998 0.9 0.723 0.999

NDUFA12 PARK7 9606.ENSP00000330737 9606.ENSP00000418770 0 0 0 0 0.133 0.897 0 0 0.906

NDUFA12 NDUFS2 9606.ENSP00000330737 9606.ENSP00000356972 0 0 0 0 0.308 0.995 0.9 0.696 0.999

NDUFA12 NDUFA6 9606.ENSP00000330737 9606.ENSP00000418842 0 0 0 0 0.945 0.998 0.9 0.718 0.999

NDUFA6 NDUFB4 9606.ENSP00000418842 9606.ENSP00000184266 0 0 0 0 0.751 0.993 0.9 0.763 0.999

NDUFA6 NDUFS8 9606.ENSP00000418842 9606.ENSP00000315774 0 0 0 0 0.889 0.998 0.9 0.644 0.999

NDUFA6 NDUFA12 9606.ENSP00000418842 9606.ENSP00000330737 0 0 0 0 0.945 0.998 0.9 0.718 0.999

NDUFA6 NDUFS2 9606.ENSP00000418842 9606.ENSP00000356972 0 0 0 0 0.288 0.995 0.9 0.73 0.999

NDUFA6 PARK7 9606.ENSP00000418842 9606.ENSP00000418770 0 0 0 0 0.218 0.879 0 0.068 0.904

NDUFB4 NDUFS8 9606.ENSP00000184266 9606.ENSP00000315774 0 0 0 0 0.283 0.842 0.9 0.39 0.992

NDUFB4 NDUFA6 9606.ENSP00000184266 9606.ENSP00000418842 0 0 0 0 0.751 0.993 0.9 0.763 0.999

NDUFB4 NDUFS2 9606.ENSP00000184266 9606.ENSP00000356972 0 0 0 0 0.175 0.987 0.9 0.5 0.999

NDUFB4 NDUFA12 9606.ENSP00000184266 9606.ENSP00000330737 0 0 0 0 0.879 0.987 0.9 0.532 0.999

NDUFS2 NDUFB4 9606.ENSP00000356972 9606.ENSP00000184266 0 0 0 0 0.175 0.987 0.9 0.5 0.999

NDUFS2 GSR 9606.ENSP00000356972 9606.ENSP00000221130 0 0 0 0 0.157 0.247 0 0.152 0.415

NDUFS2 NDUFS8 9606.ENSP00000356972 9606.ENSP00000315774 0.295 0 0.435 0 0.775 0.995 0.9 0.934 0.999

NDUFS2 NDUFA12 9606.ENSP00000356972 9606.ENSP00000330737 0 0 0 0 0.308 0.995 0.9 0.696 0.999

NDUFS2 PARK7 9606.ENSP00000356972 9606.ENSP00000418770 0 0 0 0 0.359 0 0 0.338 0.558

NDUFS2 NDUFA6 9606.ENSP00000356972 9606.ENSP00000418842 0 0 0 0 0.288 0.995 0.9 0.73 0.999

NDUFS8 NDUFB4 9606.ENSP00000315774 9606.ENSP00000184266 0 0 0 0 0.283 0.842 0.9 0.39 0.992

NDUFS8 PARK7 9606.ENSP00000315774 9606.ENSP00000418770 0 0 0 0 0.445 0 0 0.096 0.477

NDUFS8 SOD2 9606.ENSP00000315774 9606.ENSP00000446252 0.046 0 0 0 0.483 0 0 0.166 0.553

NDUFS8 NDUFA6 9606.ENSP00000315774 9606.ENSP00000418842 0 0 0 0 0.889 0.998 0.9 0.644 0.999

NDUFS8 NDUFS2 9606.ENSP00000315774 9606.ENSP00000356972 0.295 0 0.435 0 0.775 0.995 0.9 0.934 0.999

NDUFS8 NDUFA12 9606.ENSP00000315774 9606.ENSP00000330737 0 0 0 0 0.82 0.998 0.9 0.723 0.999

NFE2L2 NOX3 9606.ENSP00000380252 9606.ENSP00000159060 0 0 0 0 0 0 0 0.467 0.467

NFE2L2 HMOX1 9606.ENSP00000380252 9606.ENSP00000216117 0 0 0 0 0 0 0.9 0.916 0.991

NFE2L2 GSR 9606.ENSP00000380252 9606.ENSP00000221130 0 0 0 0 0 0 0 0.716 0.716

NFE2L2 MPO 9606.ENSP00000380252 9606.ENSP00000225275 0 0 0 0 0 0 0 0.47 0.47

NFE2L2 GCLC 9606.ENSP00000380252 9606.ENSP00000229416 0 0 0 0 0 0 0 0.859 0.859

NFE2L2 DUSP1 9606.ENSP00000380252 9606.ENSP00000239223 0 0 0 0 0 0.27 0 0.218 0.405

NFE2L2 CAT 9606.ENSP00000380252 9606.ENSP00000241052 0 0 0 0 0 0 0 0.812 0.812

NFE2L2 GSTM3 9606.ENSP00000380252 9606.ENSP00000256594 0 0 0 0 0 0 0 0.499 0.499

NFE2L2 NOX4 9606.ENSP00000380252 9606.ENSP00000263317 0 0 0 0 0 0 0 0.575 0.575

NFE2L2 PPARGC1A 9606.ENSP00000380252 9606.ENSP00000264867 0 0 0 0 0 0 0 0.675 0.675

NFE2L2 SOD1 9606.ENSP00000380252 9606.ENSP00000270142 0 0 0 0 0 0 0 0.704 0.704

NFE2L2 NOS3 9606.ENSP00000380252 9606.ENSP00000297494 0 0 0 0 0 0 0 0.556 0.556

NFE2L2 FOS 9606.ENSP00000380252 9606.ENSP00000306245 0 0 0 0 0 0.056 0.9 0.468 0.945

NFE2L2 GSTM1 9606.ENSP00000380252 9606.ENSP00000311469 0 0 0 0 0 0 0 0.503 0.503

NFE2L2 DUOX1 9606.ENSP00000380252 9606.ENSP00000317997 0 0 0 0 0 0 0 0.467 0.467

NFE2L2 NQO1 9606.ENSP00000380252 9606.ENSP00000319788 0 0 0 0 0 0.27 0 0.876 0.905

NFE2L2 SP1 9606.ENSP00000380252 9606.ENSP00000329357 0 0 0 0 0 0.056 0 0.478 0.486

NFE2L2 GSTA1 9606.ENSP00000380252 9606.ENSP00000335620 0 0 0 0 0 0 0 0.528 0.528

NFE2L2 PRDX6 9606.ENSP00000380252 9606.ENSP00000342026 0 0 0 0 0 0 0 0.485 0.485

NFE2L2 GPX4 9606.ENSP00000380252 9606.ENSP00000346103 0 0 0 0 0 0 0 0.7 0.7

NFE2L2 GCLM 9606.ENSP00000380252 9606.ENSP00000359258 0 0 0 0 0 0 0 0.811 0.811

NFE2L2 MMP9 9606.ENSP00000380252 9606.ENSP00000361405 0 0 0 0 0 0 0 0.425 0.424

NFE2L2 NOX1 9606.ENSP00000380252 9606.ENSP00000362057 0 0 0 0 0 0 0 0.475 0.475

NFE2L2 TXN 9606.ENSP00000380252 9606.ENSP00000363641 0 0 0 0 0 0 0 0.681 0.681

NFE2L2 CYBB 9606.ENSP00000380252 9606.ENSP00000367851 0 0 0 0 0 0 0 0.533 0.533

NFE2L2 CYP1A1 9606.ENSP00000380252 9606.ENSP00000369050 0 0 0 0 0 0 0 0.52 0.52

NFE2L2 SRXN1 9606.ENSP00000380252 9606.ENSP00000371388 0 0 0 0 0 0 0 0.575 0.575

NFE2L2 SOD3 9606.ENSP00000380252 9606.ENSP00000371554 0 0 0 0 0 0 0 0.419 0.419

NFE2L2 GPX3 9606.ENSP00000380252 9606.ENSP00000373477 0 0 0 0 0 0 0 0.533 0.533

NFE2L2 DUOX2 9606.ENSP00000380252 9606.ENSP00000475084 0 0 0 0 0 0 0 0.411 0.411

NFE2L2 CDKN2A 9606.ENSP00000380252 9606.ENSP00000418915 0 0 0 0 0 0 0 0.492 0.491

NFE2L2 GSTP1 9606.ENSP00000380252 9606.ENSP00000381607 0 0 0 0 0 0 0 0.573 0.573

NFE2L2 SOD2 9606.ENSP00000380252 9606.ENSP00000446252 0 0 0 0 0 0 0 0.646 0.646

NFE2L2 TXNRD1 9606.ENSP00000380252 9606.ENSP00000434516 0 0 0 0 0 0 0 0.661 0.661

NFE2L2 GPX1 9606.ENSP00000380252 9606.ENSP00000407375 0 0 0 0 0 0 0 0.667 0.667

NFE2L2 PARK7 9606.ENSP00000380252 9606.ENSP00000418770 0 0 0 0 0 0 0.8 0.576 0.911

NFE2L2 HMOX2 9606.ENSP00000380252 9606.ENSP00000477572 0 0 0 0 0 0 0.9 0.524 0.95

NFKB1 NOS2 9606.ENSP00000226574 9606.ENSP00000327251 0 0 0 0 0.062 0.06 0 0.377 0.402

NFKB1 PPARGC1A 9606.ENSP00000226574 9606.ENSP00000264867 0 0 0 0 0 0.27 0 0.222 0.408

NFKB1 MMP9 9606.ENSP00000226574 9606.ENSP00000361405 0 0 0 0 0.085 0 0 0.412 0.439

NFKB1 FOS 9606.ENSP00000226574 9606.ENSP00000306245 0 0 0 0 0.049 0.225 0 0.516 0.612

NFKB1 SP1 9606.ENSP00000226574 9606.ENSP00000329357 0 0 0 0 0.079 0.687 0 0.259 0.768

NFKB1 MAPK10 9606.ENSP00000226574 9606.ENSP00000352157 0 0 0 0 0 0.102 0.8 0.28 0.859

NFKB1 TXN 9606.ENSP00000226574 9606.ENSP00000363641 0 0 0 0 0.052 0.934 0 0.178 0.944

NFKB1 MAPK14 9606.ENSP00000226574 9606.ENSP00000229795 0 0 0 0 0.065 0.134 0.9 0.463 0.95

NOS1 HMOX1 9606.ENSP00000477999 9606.ENSP00000216117 0 0 0 0 0 0.324 0 0.672 0.769

NOS1 GSR 9606.ENSP00000477999 9606.ENSP00000221130 0 0 0 0 0 0 0 0.405 0.405

NOS1 CAT 9606.ENSP00000477999 9606.ENSP00000241052 0 0 0 0 0.049 0 0 0.598 0.602

NOS1 CYBA 9606.ENSP00000477999 9606.ENSP00000261623 0 0 0 0 0 0 0 0.407 0.407

NOS1 NOX4 9606.ENSP00000477999 9606.ENSP00000263317 0 0 0 0 0.062 0 0 0.471 0.483

NOS1 SOD1 9606.ENSP00000477999 9606.ENSP00000270142 0 0 0 0 0 0 0 0.525 0.525

NOS1 NOS3 9606.ENSP00000477999 9606.ENSP00000297494 0 0 0.442 0.959 0 0 0.8 0.916 0.81

NOS1 FOS 9606.ENSP00000477999 9606.ENSP00000306245 0 0 0 0 0 0 0 0.54 0.54

NOS1 DUOX1 9606.ENSP00000477999 9606.ENSP00000317997 0 0 0 0 0.082 0 0 0.383 0.41

NOS1 NOS2 9606.ENSP00000477999 9606.ENSP00000327251 0 0 0.438 0.95 0 0.147 0.8 0.843 0.833

NOS1 CTH 9606.ENSP00000477999 9606.ENSP00000359976 0.047 0 0 0 0.084 0 0 0.433 0.461

NOS1 NOX1 9606.ENSP00000477999 9606.ENSP00000362057 0 0 0 0 0.062 0 0 0.469 0.48

NOS1 TXN 9606.ENSP00000477999 9606.ENSP00000363641 0 0 0 0 0.062 0 0 0.421 0.434

NOS1 MTHFR 9606.ENSP00000477999 9606.ENSP00000365777 0.123 0 0 0 0.066 0.181 0 0.26 0.437

NOS1 CYBB 9606.ENSP00000477999 9606.ENSP00000367851 0 0 0 0 0.062 0 0 0.568 0.577

NOS1 PRNP 9606.ENSP00000477999 9606.ENSP00000368752 0 0 0 0 0 0.27 0 0.45 0.581

NOS1 SOD3 9606.ENSP00000477999 9606.ENSP00000371554 0 0 0 0 0.049 0 0 0.402 0.407

NOS1 SOD2 9606.ENSP00000477999 9606.ENSP00000446252 0 0 0 0 0.062 0 0 0.438 0.45

NOS1 HMOX2 9606.ENSP00000477999 9606.ENSP00000477572 0 0 0 0 0 0.177 0 0.583 0.642

NOS2 HMOX1 9606.ENSP00000327251 9606.ENSP00000216117 0 0 0 0 0 0.177 0 0.667 0.714

NOS2 MPO 9606.ENSP00000327251 9606.ENSP00000225275 0 0 0 0 0 0.09 0 0.535 0.559

NOS2 NFKB1 9606.ENSP00000327251 9606.ENSP00000226574 0 0 0 0 0.062 0.06 0 0.377 0.402

NOS2 DAO 9606.ENSP00000327251 9606.ENSP00000228476 0 0 0 0 0 0 0.9 0.131 0.909

NOS2 MAPK14 9606.ENSP00000327251 9606.ENSP00000229795 0 0 0 0 0.049 0 0 0.496 0.5

NOS2 CAT 9606.ENSP00000327251 9606.ENSP00000241052 0 0 0 0 0.049 0 0.9 0.558 0.954

NOS2 MMP13 9606.ENSP00000327251 9606.ENSP00000260302 0 0 0 0 0.062 0 0 0.398 0.411

NOS2 SOD1 9606.ENSP00000327251 9606.ENSP00000270142 0 0 0 0 0 0.115 0 0.406 0.452

NOS2 PAOX 9606.ENSP00000327251 9606.ENSP00000278060 0 0 0 0 0 0 0.9 0.089 0.905

NOS2 ACOX1 9606.ENSP00000327251 9606.ENSP00000293217 0 0 0 0 0.063 0 0.9 0.095 0.907

NOS2 NOS3 9606.ENSP00000327251 9606.ENSP00000297494 0 0 0.447 0.944 0 0.164 0.8 0.82 0.837

NOS2 PIPOX 9606.ENSP00000327251 9606.ENSP00000317721 0 0 0 0 0 0 0.9 0.141 0.91

NOS2 NOX1 9606.ENSP00000327251 9606.ENSP00000362057 0 0 0 0 0.062 0 0 0.388 0.401

NOS2 MTHFR 9606.ENSP00000327251 9606.ENSP00000365777 0.123 0 0 0 0.066 0.181 0 0.256 0.434

NOS2 SOD2 9606.ENSP00000327251 9606.ENSP00000446252 0 0 0 0 0.062 0 0 0.442 0.454

NOS2 HMOX2 9606.ENSP00000327251 9606.ENSP00000477572 0 0 0 0 0 0.177 0 0.393 0.479

NOS2 MMP9 9606.ENSP00000327251 9606.ENSP00000361405 0 0 0 0 0.065 0 0 0.535 0.547

NOS2 CYBB 9606.ENSP00000327251 9606.ENSP00000367851 0 0 0 0 0.062 0 0 0.57 0.58

NOS2 NOS1 9606.ENSP00000327251 9606.ENSP00000477999 0 0 0.438 0.95 0 0.147 0.8 0.843 0.833

NOS2 ACOX3 9606.ENSP00000327251 9606.ENSP00000348775 0 0 0 0 0.063 0 0.9 0.063 0.904

NOS2 HAO1 9606.ENSP00000327251 9606.ENSP00000368066 0.05 0 0 0 0.065 0 0.9 0.062 0.905

NOS2 SP1 9606.ENSP00000327251 9606.ENSP00000329357 0 0 0 0 0 0 0.9 0.09 0.905

NOS2 DDO 9606.ENSP00000327251 9606.ENSP00000357920 0 0 0 0 0 0 0.9 0.1 0.906

NOS2 HAO2 9606.ENSP00000327251 9606.ENSP00000483507 0.05 0 0 0 0.065 0 0.9 0.105 0.909

NOS3 NOX3 9606.ENSP00000297494 9606.ENSP00000159060 0 0 0 0 0.062 0 0 0.402 0.415

NOS3 HMOX1 9606.ENSP00000297494 9606.ENSP00000216117 0 0 0 0 0 0.177 0 0.752 0.787

NOS3 GSR 9606.ENSP00000297494 9606.ENSP00000221130 0 0 0 0 0 0 0 0.525 0.525

NOS3 PON1 9606.ENSP00000297494 9606.ENSP00000222381 0 0 0 0 0 0 0 0.553 0.553

NOS3 MPO 9606.ENSP00000297494 9606.ENSP00000225275 0 0 0 0 0 0.09 0 0.565 0.587

NOS3 MAPK14 9606.ENSP00000297494 9606.ENSP00000229795 0 0 0 0 0.049 0 0 0.46 0.465

NOS3 LOX 9606.ENSP00000297494 9606.ENSP00000231004 0 0 0 0 0 0 0 0.424 0.424

NOS3 CAT 9606.ENSP00000297494 9606.ENSP00000241052 0 0 0 0 0.049 0 0 0.744 0.746

NOS3 CYBA 9606.ENSP00000297494 9606.ENSP00000261623 0 0 0 0 0 0 0 0.681 0.681

NOS3 NOX4 9606.ENSP00000297494 9606.ENSP00000263317 0 0 0 0 0.062 0 0 0.736 0.741

NOS3 PPARGC1A 9606.ENSP00000297494 9606.ENSP00000264867 0 0 0 0 0 0 0 0.637 0.637

NOS3 SOD1 9606.ENSP00000297494 9606.ENSP00000270142 0 0 0 0 0 0 0 0.523 0.523

NOS3 FOS 9606.ENSP00000297494 9606.ENSP00000306245 0 0 0 0 0 0 0 0.467 0.467

NOS3 UCP2 9606.ENSP00000297494 9606.ENSP00000312029 0 0 0 0 0 0 0 0.468 0.468

NOS3 SP1 9606.ENSP00000297494 9606.ENSP00000329357 0 0 0 0 0 0.213 0 0.358 0.473

NOS3 GPX1 9606.ENSP00000297494 9606.ENSP00000407375 0 0 0 0 0 0 0 0.529 0.529

NOS3 TXN 9606.ENSP00000297494 9606.ENSP00000363641 0 0 0 0 0.062 0 0 0.531 0.541

NOS3 NOX5 9606.ENSP00000297494 9606.ENSP00000373518 0 0 0 0 0.062 0 0 0.533 0.543

NOS3 NFE2L2 9606.ENSP00000297494 9606.ENSP00000380252 0 0 0 0 0 0 0 0.556 0.556

NOS3 NCF2 9606.ENSP00000297494 9606.ENSP00000356505 0 0 0 0 0 0 0 0.595 0.595

NOS3 DUOX2 9606.ENSP00000297494 9606.ENSP00000475084 0 0 0 0 0.062 0 0 0.609 0.618

NOS3 SOD3 9606.ENSP00000297494 9606.ENSP00000371554 0 0 0 0 0.062 0 0 0.613 0.621

NOS3 DUOX1 9606.ENSP00000297494 9606.ENSP00000317997 0 0 0 0 0.062 0 0 0.618 0.626

NOS3 HMOX2 9606.ENSP00000297494 9606.ENSP00000477572 0 0 0 0 0 0.177 0 0.581 0.64

NOS3 SOD2 9606.ENSP00000297494 9606.ENSP00000446252 0 0 0 0 0.062 0 0 0.636 0.644

NOS3 CTH 9606.ENSP00000297494 9606.ENSP00000359976 0.047 0 0 0 0.084 0 0 0.64 0.658

NOS3 MMP9 9606.ENSP00000297494 9606.ENSP00000361405 0 0 0 0 0.062 0 0 0.658 0.665

NOS3 NOX1 9606.ENSP00000297494 9606.ENSP00000362057 0 0 0 0 0.062 0 0 0.686 0.693

NOS3 MTHFR 9606.ENSP00000297494 9606.ENSP00000365777 0.123 0 0 0 0.066 0.181 0 0.681 0.757

NOS3 CYBB 9606.ENSP00000297494 9606.ENSP00000367851 0 0 0 0 0.062 0 0 0.761 0.767

NOS3 NOS1 9606.ENSP00000297494 9606.ENSP00000477999 0 0 0.442 0.959 0 0 0.8 0.916 0.81

NOS3 NOS2 9606.ENSP00000297494 9606.ENSP00000327251 0 0 0.447 0.944 0 0.164 0.8 0.82 0.837

NOX1 NOX3 9606.ENSP00000362057 9606.ENSP00000159060 0 0 0 0.957 0 0 0.54 0.945 0.558

NOX1 HMOX1 9606.ENSP00000362057 9606.ENSP00000216117 0 0 0 0 0 0 0 0.586 0.586

NOX1 GSR 9606.ENSP00000362057 9606.ENSP00000221130 0 0 0 0 0 0 0 0.478 0.478

NOX1 MPO 9606.ENSP00000362057 9606.ENSP00000225275 0 0 0 0 0.049 0.062 0 0.471 0.487

NOX1 CAT 9606.ENSP00000362057 9606.ENSP00000241052 0 0 0 0 0 0 0 0.749 0.749

NOX1 CYBA 9606.ENSP00000362057 9606.ENSP00000261623 0 0 0 0 0.083 0.292 0.8 0.99 0.998

NOX1 NOX4 9606.ENSP00000362057 9606.ENSP00000263317 0 0 0 0.859 0 0.282 0.8 0.989 0.871

NOX1 SOD1 9606.ENSP00000362057 9606.ENSP00000270142 0 0 0 0 0 0.153 0 0.542 0.595

NOX1 NOS3 9606.ENSP00000362057 9606.ENSP00000297494 0 0 0 0 0.062 0 0 0.686 0.693

NOX1 DUOX1 9606.ENSP00000362057 9606.ENSP00000317997 0 0 0 0.756 0 0 0.54 0.944 0.645

NOX1 NQO1 9606.ENSP00000362057 9606.ENSP00000319788 0 0 0 0 0 0 0 0.409 0.409

NOX1 NOS2 9606.ENSP00000362057 9606.ENSP00000327251 0 0 0 0 0.062 0 0 0.388 0.401

NOX1 GPX4 9606.ENSP00000362057 9606.ENSP00000346103 0 0 0 0 0 0 0 0.539 0.539

NOX1 NCF2 9606.ENSP00000362057 9606.ENSP00000356505 0 0 0 0 0.104 0 0.8 0.966 0.993

NOX1 MMP9 9606.ENSP00000362057 9606.ENSP00000361405 0 0 0 0 0.062 0 0 0.521 0.532

NOX1 GPX3 9606.ENSP00000362057 9606.ENSP00000373477 0 0 0 0 0 0 0 0.407 0.407

NOX1 NFE2L2 9606.ENSP00000362057 9606.ENSP00000380252 0 0 0 0 0 0 0 0.475 0.475

NOX1 NOS1 9606.ENSP00000362057 9606.ENSP00000477999 0 0 0 0 0.062 0 0 0.469 0.48

NOX1 SOD2 9606.ENSP00000362057 9606.ENSP00000446252 0 0 0 0 0.065 0 0 0.508 0.521

NOX1 TXN 9606.ENSP00000362057 9606.ENSP00000363641 0 0 0 0 0.062 0.057 0 0.514 0.532

NOX1 GPX1 9606.ENSP00000362057 9606.ENSP00000407375 0 0 0 0 0.062 0 0 0.552 0.562

NOX1 SOD3 9606.ENSP00000362057 9606.ENSP00000371554 0 0 0 0 0.062 0.104 0 0.572 0.609

NOX1 DUOX2 9606.ENSP00000362057 9606.ENSP00000475084 0 0 0 0.744 0 0 0.54 0.857 0.64

NOX1 NOX5 9606.ENSP00000362057 9606.ENSP00000373518 0 0 0 0.75 0 0 0.54 0.992 0.654

NOX1 CYBB 9606.ENSP00000362057 9606.ENSP00000367851 0 0 0 0.959 0 0 0.8 0.99 0.808

NOX3 S100A9 9606.ENSP00000159060 9606.ENSP00000357727 0 0 0 0 0.062 0 0 0.398 0.411

NOX3 NOS3 9606.ENSP00000159060 9606.ENSP00000297494 0 0 0 0 0.062 0 0 0.402 0.415

NOX3 GPX1 9606.ENSP00000159060 9606.ENSP00000407375 0 0 0 0 0.062 0 0 0.417 0.429

NOX3 SOD1 9606.ENSP00000159060 9606.ENSP00000270142 0 0 0 0 0 0.162 0 0.348 0.43

NOX3 SOD3 9606.ENSP00000159060 9606.ENSP00000371554 0 0 0 0 0 0.104 0 0.408 0.447

NOX3 NFE2L2 9606.ENSP00000159060 9606.ENSP00000380252 0 0 0 0 0 0 0 0.467 0.467

NOX3 CYBB 9606.ENSP00000159060 9606.ENSP00000367851 0 0 0 0.963 0 0 0.54 0.963 0.556

NOX3 NOX1 9606.ENSP00000159060 9606.ENSP00000362057 0 0 0 0.957 0 0 0.54 0.945 0.558

NOX3 CAT 9606.ENSP00000159060 9606.ENSP00000241052 0 0 0 0 0 0 0 0.593 0.593

NOX3 DUOX2 9606.ENSP00000159060 9606.ENSP00000475084 0 0 0 0.751 0 0 0.54 0.82 0.633

NOX3 DUOX1 9606.ENSP00000159060 9606.ENSP00000317997 0 0 0 0.754 0 0 0.54 0.883 0.639

NOX3 NOX5 9606.ENSP00000159060 9606.ENSP00000373518 0 0 0 0.723 0 0 0.54 0.932 0.658

NOX3 NOX4 9606.ENSP00000159060 9606.ENSP00000263317 0 0 0 0.859 0 0.344 0.54 0.954 0.727

NOX3 NCF2 9606.ENSP00000159060 9606.ENSP00000356505 0 0 0 0 0.104 0 0.6 0.827 0.932

NOX3 CYBA 9606.ENSP00000159060 9606.ENSP00000261623 0 0 0 0 0.084 0.139 0.8 0.979 0.996

NOX4 NOX3 9606.ENSP00000263317 9606.ENSP00000159060 0 0 0 0.859 0 0.344 0.54 0.954 0.727

NOX4 HMOX1 9606.ENSP00000263317 9606.ENSP00000216117 0 0 0 0 0 0 0 0.645 0.645

NOX4 TXN2 9606.ENSP00000263317 9606.ENSP00000216185 0 0 0 0 0.062 0.057 0 0.425 0.447

NOX4 GSR 9606.ENSP00000263317 9606.ENSP00000221130 0 0 0 0 0 0 0 0.491 0.491

NOX4 MPO 9606.ENSP00000263317 9606.ENSP00000225275 0 0 0 0 0.049 0.062 0 0.441 0.457

NOX4 GCLC 9606.ENSP00000263317 9606.ENSP00000229416 0 0 0 0 0.062 0 0 0.399 0.413

NOX4 MAPK14 9606.ENSP00000263317 9606.ENSP00000229795 0 0 0 0 0 0.07 0 0.431 0.448

NOX4 CAT 9606.ENSP00000263317 9606.ENSP00000241052 0 0 0 0 0.062 0 0 0.789 0.793

NOX4 CYBA 9606.ENSP00000263317 9606.ENSP00000261623 0 0 0 0 0.062 0.078 0.8 0.991 0.998

NOX4 UCP2 9606.ENSP00000263317 9606.ENSP00000312029 0 0 0 0 0 0 0 0.407 0.407

NOX4 CTH 9606.ENSP00000263317 9606.ENSP00000359976 0 0 0 0 0.062 0 0 0.399 0.412

NOX4 TXNRD1 9606.ENSP00000263317 9606.ENSP00000434516 0 0 0 0 0 0 0 0.435 0.435

NOX4 NQO1 9606.ENSP00000263317 9606.ENSP00000319788 0 0 0 0 0 0 0 0.462 0.462

NOX4 PPARGC1A 9606.ENSP00000263317 9606.ENSP00000264867 0 0 0 0 0 0 0 0.464 0.463

NOX4 NOS1 9606.ENSP00000263317 9606.ENSP00000477999 0 0 0 0 0.062 0 0 0.471 0.483

NOX4 MMP9 9606.ENSP00000263317 9606.ENSP00000361405 0 0 0 0 0 0 0 0.489 0.489

NOX4 ACOX1 9606.ENSP00000263317 9606.ENSP00000293217 0 0 0 0 0 0 0 0.499 0.499

NOX4 GPX3 9606.ENSP00000263317 9606.ENSP00000373477 0 0 0 0 0 0 0 0.515 0.515

NOX4 GPX4 9606.ENSP00000263317 9606.ENSP00000346103 0 0 0 0 0 0 0 0.541 0.541

NOX4 TXN 9606.ENSP00000263317 9606.ENSP00000363641 0 0 0 0 0.062 0.057 0 0.551 0.568

NOX4 NFE2L2 9606.ENSP00000263317 9606.ENSP00000380252 0 0 0 0 0 0 0 0.575 0.575

NOX4 SOD1 9606.ENSP00000263317 9606.ENSP00000270142 0 0 0 0 0 0.104 0 0.573 0.601

NOX4 SOD3 9606.ENSP00000263317 9606.ENSP00000371554 0 0 0 0 0.062 0.104 0 0.594 0.629

NOX4 GPX1 9606.ENSP00000263317 9606.ENSP00000407375 0 0 0 0 0.062 0 0 0.623 0.631

NOX4 SOD2 9606.ENSP00000263317 9606.ENSP00000446252 0 0 0 0 0.065 0 0 0.631 0.64

NOX4 NOX5 9606.ENSP00000263317 9606.ENSP00000373518 0 0 0 0.65 0 0 0.54 0.976 0.697

NOX4 DUOX2 9606.ENSP00000263317 9606.ENSP00000475084 0 0 0 0.7 0 0.244 0.54 0.863 0.73

NOX4 DUOX1 9606.ENSP00000263317 9606.ENSP00000317997 0 0 0 0.709 0 0.244 0.54 0.914 0.733

NOX4 NOS3 9606.ENSP00000263317 9606.ENSP00000297494 0 0 0 0 0.062 0 0 0.736 0.741

NOX4 NOX1 9606.ENSP00000263317 9606.ENSP00000362057 0 0 0 0.859 0 0.282 0.8 0.989 0.871

NOX4 CYBB 9606.ENSP00000263317 9606.ENSP00000367851 0 0 0 0.874 0 0.391 0.8 0.923 0.887

NOX4 NCF2 9606.ENSP00000263317 9606.ENSP00000356505 0 0 0 0 0.063 0 0.54 0.879 0.943

NOX5 NOX3 9606.ENSP00000373518 9606.ENSP00000159060 0 0 0 0.723 0 0 0.54 0.932 0.658

NOX5 CAT 9606.ENSP00000373518 9606.ENSP00000241052 0 0 0 0 0 0 0 0.654 0.654

NOX5 CYBA 9606.ENSP00000373518 9606.ENSP00000261623 0 0 0 0 0.062 0.078 0.54 0.974 0.988

NOX5 NOX4 9606.ENSP00000373518 9606.ENSP00000263317 0 0 0 0.65 0 0 0.54 0.976 0.697

NOX5 SOD1 9606.ENSP00000373518 9606.ENSP00000270142 0 0 0 0 0 0.104 0 0.4 0.439

NOX5 NOS3 9606.ENSP00000373518 9606.ENSP00000297494 0 0 0 0 0.062 0 0 0.533 0.543

NOX5 DUOX1 9606.ENSP00000373518 9606.ENSP00000317997 0 0 0 0.706 0 0 0.54 0.975 0.671

NOX5 GPX4 9606.ENSP00000373518 9606.ENSP00000346103 0 0 0 0 0 0 0 0.426 0.426

NOX5 NCF2 9606.ENSP00000373518 9606.ENSP00000356505 0 0 0 0 0.063 0 0.54 0.871 0.939

NOX5 NOX1 9606.ENSP00000373518 9606.ENSP00000362057 0 0 0 0.75 0 0 0.54 0.992 0.654

NOX5 TXN 9606.ENSP00000373518 9606.ENSP00000363641 0 0 0 0 0.062 0.057 0 0.429 0.45

NOX5 CYBB 9606.ENSP00000373518 9606.ENSP00000367851 0 0 0 0.731 0 0 0.54 0.908 0.651

NOX5 SOD3 9606.ENSP00000373518 9606.ENSP00000371554 0 0 0 0 0 0.104 0 0.417 0.455

NOX5 DUOX2 9606.ENSP00000373518 9606.ENSP00000475084 0 0 0 0.7 0 0 0.54 0.908 0.664

NQO1 HMOX1 9606.ENSP00000319788 9606.ENSP00000216117 0 0 0 0 0.058 0 0 0.915 0.916

NQO1 GSR 9606.ENSP00000319788 9606.ENSP00000221130 0 0 0 0 0.064 0 0 0.73 0.736

NQO1 MPO 9606.ENSP00000319788 9606.ENSP00000225275 0 0 0 0 0 0 0 0.505 0.505

NQO1 GCLC 9606.ENSP00000319788 9606.ENSP00000229416 0 0 0 0 0.063 0 0 0.892 0.895

NQO1 CAT 9606.ENSP00000319788 9606.ENSP00000241052 0 0 0 0 0.062 0 0 0.755 0.76

NQO1 GSTM3 9606.ENSP00000319788 9606.ENSP00000256594 0 0 0 0 0.049 0 0 0.567 0.571

NQO1 CYBA 9606.ENSP00000319788 9606.ENSP00000261623 0 0 0 0 0 0 0 0.443 0.443

NQO1 NOX4 9606.ENSP00000319788 9606.ENSP00000263317 0 0 0 0 0 0 0 0.462 0.462

NQO1 PPARGC1A 9606.ENSP00000319788 9606.ENSP00000264867 0 0 0 0 0 0 0 0.709 0.709

NQO1 SOD1 9606.ENSP00000319788 9606.ENSP00000270142 0 0 0 0 0.062 0 0 0.638 0.646

NQO1 GSTT2B 9606.ENSP00000319788 9606.ENSP00000290765 0 0 0 0 0 0 0 0.405 0.405

NQO1 PRDX2 9606.ENSP00000319788 9606.ENSP00000301522 0 0 0 0 0 0 0 0.404 0.404

NQO1 FOS 9606.ENSP00000319788 9606.ENSP00000306245 0 0 0 0 0 0.27 0 0.391 0.536

NQO1 GSTM1 9606.ENSP00000319788 9606.ENSP00000311469 0 0 0 0 0.06 0 0 0.735 0.74

NQO1 MGST1 9606.ENSP00000319788 9606.ENSP00000379512 0 0 0 0 0.088 0 0 0.37 0.4

NQO1 NOX1 9606.ENSP00000319788 9606.ENSP00000362057 0 0 0 0 0 0 0 0.409 0.409

NQO1 MTHFR 9606.ENSP00000319788 9606.ENSP00000365777 0.056 0 0 0 0 0 0 0.454 0.462

NQO1 CYBB 9606.ENSP00000319788 9606.ENSP00000367851 0 0 0 0 0 0 0 0.462 0.462

NQO1 MT1X 9606.ENSP00000319788 9606.ENSP00000377995 0 0 0 0 0.054 0 0 0.5 0.506

NQO1 HMOX2 9606.ENSP00000319788 9606.ENSP00000477572 0 0 0 0 0.058 0 0 0.5 0.509

NQO1 GPX3 9606.ENSP00000319788 9606.ENSP00000373477 0 0 0 0 0 0 0 0.52 0.52

NQO1 PARK7 9606.ENSP00000319788 9606.ENSP00000418770 0.194 0 0 0 0.062 0 0 0.455 0.552

NQO1 IL4I1 9606.ENSP00000319788 9606.ENSP00000472474 0.048 0 0 0 0 0 0 0.562 0.565

NQO1 GPX4 9606.ENSP00000319788 9606.ENSP00000346103 0 0 0 0 0 0 0 0.583 0.583

NQO1 SOD2 9606.ENSP00000319788 9606.ENSP00000446252 0 0 0 0 0 0 0 0.647 0.647

NQO1 TXN 9606.ENSP00000319788 9606.ENSP00000363641 0 0 0 0 0.098 0 0 0.661 0.682

NQO1 GPX1 9606.ENSP00000319788 9606.ENSP00000407375 0 0 0 0 0 0 0 0.684 0.684

NQO1 GSTA1 9606.ENSP00000319788 9606.ENSP00000335620 0 0 0 0 0.049 0 0 0.693 0.696

NQO1 SRXN1 9606.ENSP00000319788 9606.ENSP00000371388 0 0 0 0 0.092 0 0 0.686 0.703

NQO1 GSTP1 9606.ENSP00000319788 9606.ENSP00000381607 0 0 0 0 0.065 0 0 0.714 0.721

NQO1 CYP1A1 9606.ENSP00000319788 9606.ENSP00000369050 0 0 0 0 0.085 0 0 0.749 0.761

NQO1 TXNRD1 9606.ENSP00000319788 9606.ENSP00000434516 0 0 0 0 0.152 0 0 0.729 0.761

NQO1 GCLM 9606.ENSP00000319788 9606.ENSP00000359258 0.072 0 0 0 0.16 0 0 0.876 0.895

NQO1 NFE2L2 9606.ENSP00000319788 9606.ENSP00000380252 0 0 0 0 0 0.27 0 0.876 0.905

NUDT1 PRDX5 9606.ENSP00000380241 9606.ENSP00000265462 0 0 0 0 0.062 0 0 0.453 0.465

NUDT1 ATOX1 9606.ENSP00000380241 9606.ENSP00000430598 0 0 0 0 0.082 0 0 0.664 0.679

PAOX DAO 9606.ENSP00000278060 9606.ENSP00000228476 0 0 0 0 0.152 0 0.9 0.499 0.953

PAOX CAT 9606.ENSP00000278060 9606.ENSP00000241052 0 0 0 0 0 0 0.9 0.455 0.943

PAOX AOC1 9606.ENSP00000278060 9606.ENSP00000411613 0 0 0 0 0 0 0 0.749 0.749

PAOX NOS2 9606.ENSP00000278060 9606.ENSP00000327251 0 0 0 0 0 0 0.9 0.089 0.905

PAOX HAO2 9606.ENSP00000278060 9606.ENSP00000483507 0 0 0 0 0 0 0.9 0.147 0.911

PAOX ACOX3 9606.ENSP00000278060 9606.ENSP00000348775 0 0 0 0 0.058 0 0.9 0.156 0.913

PAOX ACOX1 9606.ENSP00000278060 9606.ENSP00000293217 0 0 0 0 0.049 0 0.9 0.202 0.917

PAOX HAO1 9606.ENSP00000278060 9606.ENSP00000368066 0 0 0 0 0.06 0 0.9 0.289 0.927

PAOX PIPOX 9606.ENSP00000278060 9606.ENSP00000317721 0 0 0 0 0.094 0 0.9 0.42 0.942

PAOX DDO 9606.ENSP00000278060 9606.ENSP00000357920 0 0 0 0 0.152 0 0.9 0.515 0.955

PARK7 HMOX1 9606.ENSP00000418770 9606.ENSP00000216117 0 0 0 0 0.062 0 0 0.459 0.47

PARK7 GSR 9606.ENSP00000418770 9606.ENSP00000221130 0.077 0 0 0 0 0 0 0.431 0.453

PARK7 PON2 9606.ENSP00000418770 9606.ENSP00000222572 0 0 0 0 0.062 0 0 0.652 0.661

PARK7 CAT 9606.ENSP00000418770 9606.ENSP00000241052 0.12 0 0 0 0.168 0.253 0 0.641 0.777

PARK7 PPARGC1A 9606.ENSP00000418770 9606.ENSP00000264867 0 0 0 0 0 0.209 0 0.52 0.605

PARK7 PRDX5 9606.ENSP00000418770 9606.ENSP00000265462 0 0 0 0 0.133 0.825 0 0.36 0.894

PARK7 SOD1 9606.ENSP00000418770 9606.ENSP00000270142 0.046 0 0 0 0.372 0.704 0 0.847 0.969

PARK7 PRDX2 9606.ENSP00000418770 9606.ENSP00000301522 0.07 0 0 0 0.14 0.672 0 0.765 0.93

PARK7 NDUFS8 9606.ENSP00000418770 9606.ENSP00000315774 0 0 0 0 0.445 0 0 0.096 0.477

PARK7 NQO1 9606.ENSP00000418770 9606.ENSP00000319788 0.194 0 0 0 0.062 0 0 0.455 0.552

PARK7 NDUFA12 9606.ENSP00000418770 9606.ENSP00000330737 0 0 0 0 0.133 0.897 0 0 0.906

PARK7 PRDX6 9606.ENSP00000418770 9606.ENSP00000342026 0.07 0 0 0 0.171 0.256 0 0.586 0.731

PARK7 GPX4 9606.ENSP00000418770 9606.ENSP00000346103 0 0 0 0 0.094 0.346 0 0.45 0.646

PARK7 NDUFS2 9606.ENSP00000418770 9606.ENSP00000356972 0 0 0 0 0.359 0 0 0.338 0.558

PARK7 GCLM 9606.ENSP00000418770 9606.ENSP00000359258 0.081 0 0 0 0.096 0 0 0.48 0.53

PARK7 TXN 9606.ENSP00000418770 9606.ENSP00000363641 0 0 0 0 0.183 0.079 0 0.76 0.804

PARK7 MAOB 9606.ENSP00000418770 9606.ENSP00000367309 0.06 0 0 0 0 0 0 0.525 0.534

PARK7 SOD3 9606.ENSP00000418770 9606.ENSP00000371554 0.046 0 0 0 0.093 0.17 0 0.396 0.508

PARK7 GPX3 9606.ENSP00000418770 9606.ENSP00000373477 0 0 0 0 0.066 0.141 0 0.32 0.407

PARK7 NFE2L2 9606.ENSP00000418770 9606.ENSP00000380252 0 0 0 0 0 0 0.8 0.576 0.911

PARK7 GSTP1 9606.ENSP00000418770 9606.ENSP00000381607 0 0 0 0 0.09 0.129 0 0.317 0.411

PARK7 GPX1 9606.ENSP00000418770 9606.ENSP00000407375 0 0 0 0 0.066 0.141 0 0.423 0.496

PARK7 TXNRD1 9606.ENSP00000418770 9606.ENSP00000434516 0.212 0 0 0 0.069 0 0 0.442 0.555

PARK7 SOD2 9606.ENSP00000418770 9606.ENSP00000446252 0.165 0 0 0 0.494 0 0 0.621 0.825

PARK7 NDUFA6 9606.ENSP00000418770 9606.ENSP00000418842 0 0 0 0 0.218 0.879 0 0.068 0.904

PCYOX1 PON1 9606.ENSP00000387654 9606.ENSP00000222381 0 0 0 0 0 0 0.72 0.461 0.842

PCYOX1 PON2 9606.ENSP00000387654 9606.ENSP00000222572 0 0 0 0 0 0 0 0.44 0.44

PCYOX1 APOA4 9606.ENSP00000387654 9606.ENSP00000350425 0 0 0 0 0 0 0.72 0.135 0.747

PCYOX1 VIMP 9606.ENSP00000387654 9606.ENSP00000381282 0 0 0 0 0 0 0.72 0 0.72

PIPOX DAO 9606.ENSP00000317721 9606.ENSP00000228476 0 0 0 0 0.065 0 0.9 0.563 0.955

PIPOX CAT 9606.ENSP00000317721 9606.ENSP00000241052 0.088 0 0 0 0.062 0 0.9 0.253 0.927

PIPOX PAOX 9606.ENSP00000317721 9606.ENSP00000278060 0 0 0 0 0.094 0 0.9 0.42 0.942

PIPOX ACOX1 9606.ENSP00000317721 9606.ENSP00000293217 0 0 0 0 0.063 0 0.9 0.549 0.954

PIPOX AOC3 9606.ENSP00000317721 9606.ENSP00000312326 0 0 0 0 0.051 0 0 0.436 0.442

PIPOX APOA4 9606.ENSP00000317721 9606.ENSP00000350425 0 0 0 0 0.521 0 0 0 0.521

PIPOX NOS2 9606.ENSP00000317721 9606.ENSP00000327251 0 0 0 0 0 0 0.9 0.141 0.91

PIPOX ACOX3 9606.ENSP00000317721 9606.ENSP00000348775 0 0 0 0 0.062 0 0.9 0.596 0.958

PIPOX DDO 9606.ENSP00000317721 9606.ENSP00000357920 0 0 0 0 0.065 0 0.9 0.605 0.959

PIPOX HAO1 9606.ENSP00000317721 9606.ENSP00000368066 0 0 0 0 0.185 0 0.9 0.689 0.972

PIPOX HAO2 9606.ENSP00000317721 9606.ENSP00000483507 0 0 0 0 0.515 0 0.9 0.7 0.984

PNKP ERCC1 9606.ENSP00000323511 9606.ENSP00000013807 0 0 0 0 0.12 0.144 0 0.679 0.737

PNKP PNPO 9606.ENSP00000323511 9606.ENSP00000225573 0 0 0 0 0 0 0 0.535 0.535

PNKP APTX 9606.ENSP00000323511 9606.ENSP00000400806 0 0 0 0.608 0 0.115 0 0.907 0.428

PNKP ERCC2 9606.ENSP00000323511 9606.ENSP00000375809 0 0 0 0 0.107 0 0 0.439 0.478

PNPO PNKP 9606.ENSP00000225573 9606.ENSP00000323511 0 0 0 0 0 0 0 0.535 0.535

PNPO MTHFR 9606.ENSP00000225573 9606.ENSP00000365777 0.111 0 0 0 0.067 0 0 0.565 0.608

PNPO AOX1 9606.ENSP00000225573 9606.ENSP00000363832 0 0 0 0 0 0 0.9 0.269 0.923

PON1 GSR 9606.ENSP00000222381 9606.ENSP00000221130 0 0 0 0 0 0 0 0.404 0.404

PON1 GSTP1 9606.ENSP00000222381 9606.ENSP00000381607 0 0 0 0 0.062 0 0 0.402 0.415

PON1 CYP1A1 9606.ENSP00000222381 9606.ENSP00000369050 0 0 0 0 0.054 0 0 0.451 0.458

PON1 GPX1 9606.ENSP00000222381 9606.ENSP00000407375 0 0 0 0 0.064 0 0 0.46 0.473

PON1 GSTM1 9606.ENSP00000222381 9606.ENSP00000311469 0 0 0 0 0.062 0 0 0.517 0.527

PON1 NOS3 9606.ENSP00000222381 9606.ENSP00000297494 0 0 0 0 0 0 0 0.553 0.553

PON1 CAT 9606.ENSP00000222381 9606.ENSP00000241052 0 0 0 0 0.062 0 0 0.596 0.604

PON1 PON2 9606.ENSP00000222381 9606.ENSP00000222572 0 0 0.44 0.971 0 0 0.6 0.877 0.614

PON1 MTHFR 9606.ENSP00000222381 9606.ENSP00000365777 0 0 0 0 0 0 0 0.651 0.651

PON1 VIMP 9606.ENSP00000222381 9606.ENSP00000381282 0 0 0 0 0 0 0.72 0 0.72

PON1 PCYOX1 9606.ENSP00000222381 9606.ENSP00000387654 0 0 0 0 0 0 0.72 0.461 0.842

PON1 APOA4 9606.ENSP00000222381 9606.ENSP00000350425 0 0 0 0 0.076 0 0.72 0.576 0.88

PON1 MPO 9606.ENSP00000222381 9606.ENSP00000225275 0 0 0 0 0 0 0 0.923 0.923

PON2 PON1 9606.ENSP00000222572 9606.ENSP00000222381 0 0 0.44 0.971 0 0 0.6 0.877 0.614

PON2 CAT 9606.ENSP00000222572 9606.ENSP00000241052 0 0 0 0 0.063 0 0 0.391 0.404

PON2 PCYOX1 9606.ENSP00000222572 9606.ENSP00000387654 0 0 0 0 0 0 0 0.44 0.44

PON2 HMOX2 9606.ENSP00000222572 9606.ENSP00000477572 0 0 0 0 0 0 0 0.494 0.494

PON2 PARK7 9606.ENSP00000222572 9606.ENSP00000418770 0 0 0 0 0.062 0 0 0.652 0.661

PPARGC1A HMOX1 9606.ENSP00000264867 9606.ENSP00000216117 0 0 0 0 0 0 0 0.719 0.719

PPARGC1A TXN2 9606.ENSP00000264867 9606.ENSP00000216185 0 0 0 0 0 0 0 0.406 0.406

PPARGC1A GSR 9606.ENSP00000264867 9606.ENSP00000221130 0 0 0 0 0 0 0 0.401 0.401

PPARGC1A NFKB1 9606.ENSP00000264867 9606.ENSP00000226574 0 0 0 0 0 0.27 0 0.222 0.408

PPARGC1A MAPK14 9606.ENSP00000264867 9606.ENSP00000229795 0 0 0 0 0 0.213 0.9 0.457 0.953

PPARGC1A CAT 9606.ENSP00000264867 9606.ENSP00000241052 0 0 0 0 0 0 0 0.718 0.718

PPARGC1A NOX4 9606.ENSP00000264867 9606.ENSP00000263317 0 0 0 0 0 0 0 0.464 0.463

PPARGC1A TXN 9606.ENSP00000264867 9606.ENSP00000363641 0 0 0 0 0 0 0 0.422 0.422

PPARGC1A TXNRD2 9606.ENSP00000264867 9606.ENSP00000383365 0 0 0 0 0 0 0 0.494 0.494

PPARGC1A PARK7 9606.ENSP00000264867 9606.ENSP00000418770 0 0 0 0 0 0.209 0 0.52 0.605

PPARGC1A NOS3 9606.ENSP00000264867 9606.ENSP00000297494 0 0 0 0 0 0 0 0.637 0.637

PPARGC1A NFE2L2 9606.ENSP00000264867 9606.ENSP00000380252 0 0 0 0 0 0 0 0.675 0.675

PPARGC1A NQO1 9606.ENSP00000264867 9606.ENSP00000319788 0 0 0 0 0 0 0 0.709 0.709

PPARGC1A SOD3 9606.ENSP00000264867 9606.ENSP00000371554 0 0 0 0 0.07 0 0 0.722 0.73

PPARGC1A UCP2 9606.ENSP00000264867 9606.ENSP00000312029 0 0 0 0 0 0 0 0.766 0.766

PPARGC1A GPX3 9606.ENSP00000264867 9606.ENSP00000373477 0 0 0 0 0 0 0.8 0.218 0.836

PPARGC1A GPX1 9606.ENSP00000264867 9606.ENSP00000407375 0 0 0 0 0 0 0.8 0.602 0.917

PPARGC1A RUNX2 9606.ENSP00000264867 9606.ENSP00000360493 0 0 0 0 0 0 0.9 0.254 0.922

PPARGC1A SOD1 9606.ENSP00000264867 9606.ENSP00000270142 0 0 0 0 0 0 0.8 0.628 0.922

PPARGC1A SOD2 9606.ENSP00000264867 9606.ENSP00000446252 0 0 0 0 0 0 0.8 0.736 0.945

PPARGC1A ACOX1 9606.ENSP00000264867 9606.ENSP00000293217 0 0 0 0 0.062 0 0.9 0.647 0.964

PRDX2 TXN2 9606.ENSP00000301522 9606.ENSP00000216185 0.045 0 0 0 0.112 0.464 0 0.558 0.772

PRDX2 GSR 9606.ENSP00000301522 9606.ENSP00000221130 0 0 0 0 0.159 0.067 0 0.682 0.729

PRDX2 MPO 9606.ENSP00000301522 9606.ENSP00000225275 0 0 0 0 0 0 0 0.458 0.459

PRDX2 GCLC 9606.ENSP00000301522 9606.ENSP00000229416 0 0 0 0 0.053 0 0 0.51 0.516

PRDX2 CAT 9606.ENSP00000301522 9606.ENSP00000241052 0.051 0 0 0 0.268 0.436 0 0.797 0.91

PRDX2 PRDX5 9606.ENSP00000301522 9606.ENSP00000265462 0 0 0 0 0.158 0.811 0.5 0.819 0.983

PRDX2 SOD1 9606.ENSP00000301522 9606.ENSP00000270142 0 0 0 0 0.274 0.708 0 0.65 0.919

PRDX2 NQO1 9606.ENSP00000301522 9606.ENSP00000319788 0 0 0 0 0 0 0 0.404 0.404

PRDX2 SOD3 9606.ENSP00000301522 9606.ENSP00000371554 0 0 0 0 0.111 0.068 0 0.39 0.451

PRDX2 SEPP1 9606.ENSP00000301522 9606.ENSP00000420939 0 0 0 0 0 0 0 0.492 0.492

PRDX2 GLRX2 9606.ENSP00000301522 9606.ENSP00000356410 0.074 0 0 0 0.074 0.112 0 0.48 0.551

PRDX2 TXNRD2 9606.ENSP00000301522 9606.ENSP00000383365 0 0 0 0 0.068 0.067 0 0.585 0.608

PRDX2 GSTP1 9606.ENSP00000301522 9606.ENSP00000381607 0 0 0 0 0.108 0.156 0 0.55 0.632

PRDX2 GPX3 9606.ENSP00000301522 9606.ENSP00000373477 0 0 0 0 0.062 0.185 0 0.58 0.651

PRDX2 GPX1 9606.ENSP00000301522 9606.ENSP00000407375 0 0 0 0 0.062 0.185 0 0.63 0.692

PRDX2 SOD2 9606.ENSP00000301522 9606.ENSP00000446252 0.154 0 0 0 0.112 0 0 0.628 0.696

PRDX2 SRXN1 9606.ENSP00000301522 9606.ENSP00000371388 0 0 0 0 0.067 0.255 0 0.682 0.759

PRDX2 TXNRD1 9606.ENSP00000301522 9606.ENSP00000434516 0.18 0 0 0 0.109 0.154 0 0.686 0.78

PRDX2 PRDX6 9606.ENSP00000301522 9606.ENSP00000342026 0 0 0.292 0.73 0.119 0.748 0 0.784 0.83

PRDX2 GPX4 9606.ENSP00000301522 9606.ENSP00000346103 0 0 0 0 0.097 0.47 0 0.692 0.84

PRDX2 PARK7 9606.ENSP00000301522 9606.ENSP00000418770 0.07 0 0 0 0.14 0.672 0 0.765 0.93

PRDX2 TXN 9606.ENSP00000301522 9606.ENSP00000363641 0.045 0 0 0 0.079 0.78 0.9 0.896 0.997

PRDX5 TXN2 9606.ENSP00000265462 9606.ENSP00000216185 0 0 0 0 0.067 0.301 0.9 0.776 0.983

PRDX5 GSR 9606.ENSP00000265462 9606.ENSP00000221130 0.166 0 0 0 0.138 0 0 0.774 0.823

PRDX5 GCLC 9606.ENSP00000265462 9606.ENSP00000229416 0 0 0 0 0 0 0 0.431 0.431

PRDX5 CAT 9606.ENSP00000265462 9606.ENSP00000241052 0.107 0 0 0 0 0.619 0 0.809 0.929

PRDX5 SESN2 9606.ENSP00000265462 9606.ENSP00000253063 0 0 0 0 0 0 0 0.62 0.62

PRDX5 MSRA 9606.ENSP00000265462 9606.ENSP00000313921 0.124 0 0 0 0.066 0 0 0.358 0.428

PRDX5 NUDT1 9606.ENSP00000265462 9606.ENSP00000380241 0 0 0 0 0.062 0 0 0.453 0.465

PRDX5 ATOX1 9606.ENSP00000265462 9606.ENSP00000430598 0 0 0 0 0.082 0 0 0.494 0.516

PRDX5 SOD3 9606.ENSP00000265462 9606.ENSP00000371554 0.048 0 0 0 0.132 0 0 0.505 0.555

PRDX5 TXNRD2 9606.ENSP00000265462 9606.ENSP00000383365 0.166 0 0 0 0.091 0 0 0.527 0.61

PRDX5 GPX3 9606.ENSP00000265462 9606.ENSP00000373477 0 0 0 0 0.066 0.13 0 0.576 0.626

PRDX5 GLRX2 9606.ENSP00000265462 9606.ENSP00000356410 0.044 0 0 0 0.073 0 0 0.675 0.687

PRDX5 GPX4 9606.ENSP00000265462 9606.ENSP00000346103 0 0 0 0 0.111 0.13 0 0.639 0.696

PRDX5 GPX1 9606.ENSP00000265462 9606.ENSP00000407375 0 0 0 0 0.093 0.13 0 0.65 0.7

PRDX5 SRXN1 9606.ENSP00000265462 9606.ENSP00000371388 0 0 0 0 0.062 0 0 0.783 0.788

PRDX5 TXNRD1 9606.ENSP00000265462 9606.ENSP00000434516 0.288 0 0 0 0.125 0 0 0.751 0.831

PRDX5 SOD1 9606.ENSP00000265462 9606.ENSP00000270142 0.048 0 0 0 0.158 0.493 0 0.708 0.865

PRDX5 SOD2 9606.ENSP00000265462 9606.ENSP00000446252 0 0 0 0 0.264 0.31 0 0.802 0.89

PRDX5 PARK7 9606.ENSP00000265462 9606.ENSP00000418770 0 0 0 0 0.133 0.825 0 0.36 0.894

PRDX5 PRDX6 9606.ENSP00000265462 9606.ENSP00000342026 0 0 0 0 0.172 0.586 0 0.792 0.922

PRDX5 PRDX2 9606.ENSP00000265462 9606.ENSP00000301522 0 0 0 0 0.158 0.811 0.5 0.819 0.983

PRDX5 TXN 9606.ENSP00000265462 9606.ENSP00000363641 0 0 0 0 0.107 0.451 0.9 0.718 0.984

PRDX6 TXN2 9606.ENSP00000342026 9606.ENSP00000216185 0.045 0 0 0 0.062 0.226 0 0.629 0.708

PRDX6 GSS 9606.ENSP00000342026 9606.ENSP00000216951 0 0 0 0 0.081 0 0.9 0.11 0.911

PRDX6 GSR 9606.ENSP00000342026 9606.ENSP00000221130 0 0 0 0 0.083 0.115 0 0.695 0.731

PRDX6 GCLC 9606.ENSP00000342026 9606.ENSP00000229416 0 0 0 0 0 0 0 0.416 0.416

PRDX6 CAT 9606.ENSP00000342026 9606.ENSP00000241052 0.051 0 0 0 0.268 0.213 0 0.795 0.873

PRDX6 GSTM3 9606.ENSP00000342026 9606.ENSP00000256594 0 0 0 0 0.062 0.078 0.65 0.281 0.753

PRDX6 CYBA 9606.ENSP00000342026 9606.ENSP00000261623 0 0 0 0 0 0 0 0.444 0.444

PRDX6 PRDX5 9606.ENSP00000342026 9606.ENSP00000265462 0 0 0 0 0.172 0.586 0 0.792 0.922

PRDX6 SOD1 9606.ENSP00000342026 9606.ENSP00000270142 0 0 0 0 0.136 0.152 0 0.636 0.711

PRDX6 GSTT2B 9606.ENSP00000342026 9606.ENSP00000290765 0 0 0 0 0.108 0.078 0.65 0.267 0.761

PRDX6 PRDX2 9606.ENSP00000342026 9606.ENSP00000301522 0 0 0.292 0.73 0.119 0.748 0 0.784 0.83

PRDX6 GSTM1 9606.ENSP00000342026 9606.ENSP00000311469 0 0 0 0 0.062 0.078 0.65 0.317 0.765

PRDX6 MSRA 9606.ENSP00000342026 9606.ENSP00000313921 0.049 0 0 0 0 0 0 0.4 0.405

PRDX6 GSTA1 9606.ENSP00000342026 9606.ENSP00000335620 0 0 0 0 0.062 0.078 0.65 0.131 0.701

PRDX6 CYBB 9606.ENSP00000342026 9606.ENSP00000367851 0 0 0 0 0 0 0 0.466 0.466

PRDX6 TXNRD2 9606.ENSP00000342026 9606.ENSP00000383365 0 0 0 0 0.068 0.067 0 0.452 0.482

PRDX6 NFE2L2 9606.ENSP00000342026 9606.ENSP00000380252 0 0 0 0 0 0 0 0.485 0.485

PRDX6 SOD3 9606.ENSP00000342026 9606.ENSP00000371554 0 0 0 0 0.111 0.068 0 0.545 0.59

PRDX6 GPX3 9606.ENSP00000342026 9606.ENSP00000373477 0 0 0 0 0.063 0.174 0 0.536 0.61

PRDX6 SRXN1 9606.ENSP00000342026 9606.ENSP00000371388 0 0 0 0 0.083 0.239 0 0.558 0.665

PRDX6 GPX1 9606.ENSP00000342026 9606.ENSP00000407375 0 0 0 0 0.063 0.174 0 0.626 0.685

PRDX6 GLRX2 9606.ENSP00000342026 9606.ENSP00000356410 0.074 0 0 0 0.171 0.217 0 0.567 0.704

PRDX6 PARK7 9606.ENSP00000342026 9606.ENSP00000418770 0.07 0 0 0 0.171 0.256 0 0.586 0.731

PRDX6 MGST1 9606.ENSP00000342026 9606.ENSP00000379512 0 0 0 0 0.049 0 0.65 0.308 0.749

PRDX6 SOD2 9606.ENSP00000342026 9606.ENSP00000446252 0.154 0 0 0 0.233 0 0 0.686 0.778

PRDX6 TXNRD1 9606.ENSP00000342026 9606.ENSP00000434516 0.18 0 0 0 0.131 0.18 0 0.719 0.814

PRDX6 GPX4 9606.ENSP00000342026 9606.ENSP00000346103 0 0 0 0 0.064 0.378 0 0.739 0.834

PRDX6 NCF2 9606.ENSP00000342026 9606.ENSP00000356505 0 0 0 0 0 0.345 0 0.783 0.852

PRDX6 TXN 9606.ENSP00000342026 9606.ENSP00000363641 0.045 0 0 0 0.126 0.226 0 0.807 0.858

PRDX6 GSTP1 9606.ENSP00000342026 9606.ENSP00000381607 0 0 0 0 0.083 0.558 0.8 0.475 0.951

PRNP SOD1 9606.ENSP00000368752 9606.ENSP00000270142 0 0 0 0 0 0 0 0.483 0.483

PRNP SUOX 9606.ENSP00000368752 9606.ENSP00000377668 0 0 0 0 0 0 0 0.489 0.489

PRNP NOS1 9606.ENSP00000368752 9606.ENSP00000477999 0 0 0 0 0 0.27 0 0.45 0.581

QSOX1 GFER 9606.ENSP00000356574 9606.ENSP00000248114 0 0 0 0 0 0 0 0.75 0.75

QSOX1 TXN 9606.ENSP00000356574 9606.ENSP00000363641 0 0 0 0 0 0 0 0.651 0.651

QSOX2 GFER 9606.ENSP00000351536 9606.ENSP00000248114 0 0 0 0 0.065 0 0 0.767 0.773

QSOX2 TXN 9606.ENSP00000351536 9606.ENSP00000363641 0 0 0 0 0 0 0 0.669 0.669

RUNX2 COL1A1 9606.ENSP00000360493 9606.ENSP00000225964 0 0 0 0 0 0 0.9 0.857 0.985

RUNX2 MAPK14 9606.ENSP00000360493 9606.ENSP00000229795 0 0 0 0 0 0.106 0 0.37 0.412

RUNX2 MMP13 9606.ENSP00000360493 9606.ENSP00000260302 0 0 0 0 0 0 0.9 0.779 0.976

RUNX2 TIMP2 9606.ENSP00000360493 9606.ENSP00000262768 0 0 0 0 0 0 0 0.45 0.45

RUNX2 PPARGC1A 9606.ENSP00000360493 9606.ENSP00000264867 0 0 0 0 0 0 0.9 0.254 0.922

RUNX2 MMP3 9606.ENSP00000360493 9606.ENSP00000299855 0 0 0 0 0 0 0 0.482 0.482

RUNX2 FOS 9606.ENSP00000360493 9606.ENSP00000306245 0 0 0 0 0 0.486 0.8 0.898 0.988

RUNX2 CDKN2A 9606.ENSP00000360493 9606.ENSP00000418915 0 0 0 0 0 0 0 0.421 0.42

RUNX2 MMP9 9606.ENSP00000360493 9606.ENSP00000361405 0 0 0 0 0.062 0 0 0.581 0.59

RUNX2 COL2A1 9606.ENSP00000360493 9606.ENSP00000369889 0 0 0 0 0 0 0 0.822 0.822

RUNX2 ACAN 9606.ENSP00000360493 9606.ENSP00000387356 0 0 0 0 0 0 0 0.837 0.837

S100A7 S100A8 9606.ENSP00000357712 9606.ENSP00000357722 0 0 0 0 0.142 0 0 0.518 0.569

S100A7 S100A9 9606.ENSP00000357712 9606.ENSP00000357727 0 0 0 0 0.153 0 0 0.603 0.65

S100A8 MPO 9606.ENSP00000357722 9606.ENSP00000225275 0 0 0 0 0.232 0.128 0 0.341 0.52

S100A8 CYBA 9606.ENSP00000357722 9606.ENSP00000261623 0 0 0 0 0.087 0 0.6 0.042 0.619

S100A8 NCF2 9606.ENSP00000357722 9606.ENSP00000356505 0 0 0 0 0.563 0.27 0.6 0.451 0.92

S100A8 S100A7 9606.ENSP00000357722 9606.ENSP00000357712 0 0 0 0 0.142 0 0 0.518 0.569

S100A8 MMP9 9606.ENSP00000357722 9606.ENSP00000361405 0 0 0 0 0.23 0 0 0.341 0.471

S100A8 CYBB 9606.ENSP00000357722 9606.ENSP00000367851 0 0 0 0 0.347 0 0.6 0.15 0.758

S100A8 S100A9 9606.ENSP00000357722 9606.ENSP00000357727 0 0 0 0.801 0.995 0.95 0.9 0.987 0.999

S100A9 NOX3 9606.ENSP00000357727 9606.ENSP00000159060 0 0 0 0 0.062 0 0 0.398 0.411

S100A9 MPO 9606.ENSP00000357727 9606.ENSP00000225275 0 0 0 0 0.231 0 0 0.394 0.514

S100A9 CYBA 9606.ENSP00000357727 9606.ENSP00000261623 0 0 0 0 0.087 0 0.6 0.072 0.632

S100A9 NCF2 9606.ENSP00000357727 9606.ENSP00000356505 0 0 0 0 0.564 0.27 0.6 0.145 0.876

S100A9 S100A7 9606.ENSP00000357727 9606.ENSP00000357712 0 0 0 0 0.153 0 0 0.603 0.65

S100A9 S100A8 9606.ENSP00000357727 9606.ENSP00000357722 0 0 0 0.801 0.995 0.95 0.9 0.987 0.999

S100A9 MMP9 9606.ENSP00000357727 9606.ENSP00000361405 0 0 0 0 0.314 0 0 0.34 0.528

S100A9 CYBB 9606.ENSP00000357727 9606.ENSP00000367851 0 0 0 0 0.351 0 0.6 0.154 0.761

SEPP1 PRDX2 9606.ENSP00000420939 9606.ENSP00000301522 0 0 0 0 0 0 0 0.492 0.492

SEPP1 GPX4 9606.ENSP00000420939 9606.ENSP00000346103 0 0 0 0 0.062 0 0 0.812 0.816

SEPP1 TXN 9606.ENSP00000420939 9606.ENSP00000363641 0 0 0 0 0 0 0 0.511 0.511

SEPP1 GPX3 9606.ENSP00000420939 9606.ENSP00000373477 0 0 0 0 0.117 0 0 0.892 0.901

SEPP1 VIMP 9606.ENSP00000420939 9606.ENSP00000381282 0 0 0 0 0 0 0 0.811 0.811

SEPP1 TXNRD2 9606.ENSP00000420939 9606.ENSP00000383365 0 0 0 0 0 0 0 0.792 0.792

SEPP1 GPX1 9606.ENSP00000420939 9606.ENSP00000407375 0 0 0 0 0.062 0 0 0.811 0.815

SEPP1 TXNRD1 9606.ENSP00000420939 9606.ENSP00000434516 0 0 0 0 0 0 0 0.756 0.756

SESN2 GPX1 9606.ENSP00000253063 9606.ENSP00000407375 0 0 0 0 0 0 0 0.403 0.403

SESN2 SRXN1 9606.ENSP00000253063 9606.ENSP00000371388 0 0 0 0 0.069 0 0 0.415 0.432

SESN2 PRDX5 9606.ENSP00000253063 9606.ENSP00000265462 0 0 0 0 0 0 0 0.62 0.62

SGK2 SGK3 9606.ENSP00000340608 9606.ENSP00000379842 0 0 0.435 0.959 0.063 0 0.8 0.763 0.813

SGK3 SGK2 9606.ENSP00000379842 9606.ENSP00000340608 0 0 0.435 0.959 0.063 0 0.8 0.763 0.813

SMOX AOC2 9606.ENSP00000478305 9606.ENSP00000253799 0 0 0 0 0 0 0 0.48 0.48

SMOX MAOA 9606.ENSP00000478305 9606.ENSP00000340684 0 0 0 0 0 0 0 0.463 0.464

SMOX HAO1 9606.ENSP00000478305 9606.ENSP00000368066 0 0 0 0 0 0 0 0.608 0.608

SMOX AOC1 9606.ENSP00000478305 9606.ENSP00000411613 0 0 0 0 0 0 0 0.536 0.536

SOD1 NOX3 9606.ENSP00000270142 9606.ENSP00000159060 0 0 0 0 0 0.162 0 0.348 0.43

SOD1 HMOX1 9606.ENSP00000270142 9606.ENSP00000216117 0 0 0 0 0 0 0 0.708 0.708

SOD1 TXN2 9606.ENSP00000270142 9606.ENSP00000216185 0 0 0 0 0.104 0.36 0 0.488 0.68

SOD1 GSR 9606.ENSP00000270142 9606.ENSP00000221130 0 0 0 0 0.073 0.117 0 0.84 0.858

SOD1 GCLC 9606.ENSP00000270142 9606.ENSP00000229416 0 0 0 0 0.062 0.348 0 0.776 0.851

SOD1 CAT 9606.ENSP00000270142 9606.ENSP00000241052 0.111 0 0 0 0.124 0.52 0.9 0.923 0.996

SOD1 GFER 9606.ENSP00000270142 9606.ENSP00000248114 0 0 0 0 0.053 0 0 0.508 0.514

SOD1 CYBA 9606.ENSP00000270142 9606.ENSP00000261623 0 0 0 0 0 0 0 0.444 0.444

SOD1 NOX4 9606.ENSP00000270142 9606.ENSP00000263317 0 0 0 0 0 0.104 0 0.573 0.601

SOD1 PPARGC1A 9606.ENSP00000270142 9606.ENSP00000264867 0 0 0 0 0 0 0.8 0.628 0.922

SOD1 PRDX5 9606.ENSP00000270142 9606.ENSP00000265462 0.048 0 0 0 0.158 0.493 0 0.708 0.865

SOD1 MTHFR 9606.ENSP00000270142 9606.ENSP00000365777 0.115 0 0 0 0 0.082 0 0.33 0.408

SOD1 NOX5 9606.ENSP00000270142 9606.ENSP00000373518 0 0 0 0 0 0.104 0 0.4 0.439

SOD1 SRXN1 9606.ENSP00000270142 9606.ENSP00000371388 0 0 0 0 0.112 0 0 0.403 0.447

SOD1 NOS2 9606.ENSP00000270142 9606.ENSP00000327251 0 0 0 0 0 0.115 0 0.406 0.452

SOD1 MGST1 9606.ENSP00000270142 9606.ENSP00000379512 0 0 0 0 0.089 0 0 0.424 0.453

SOD1 DUOX2 9606.ENSP00000270142 9606.ENSP00000475084 0 0 0 0 0 0.141 0 0.404 0.467

SOD1 UCP2 9606.ENSP00000270142 9606.ENSP00000312029 0 0 0 0 0 0 0 0.481 0.481

SOD1 PRNP 9606.ENSP00000270142 9606.ENSP00000368752 0 0 0 0 0 0 0 0.483 0.483

SOD1 DUOX1 9606.ENSP00000270142 9606.ENSP00000317997 0 0 0 0 0 0.141 0 0.465 0.521

SOD1 NOS3 9606.ENSP00000270142 9606.ENSP00000297494 0 0 0 0 0 0 0 0.523 0.523

SOD1 NOS1 9606.ENSP00000270142 9606.ENSP00000477999 0 0 0 0 0 0 0 0.525 0.525

SOD1 GSTP1 9606.ENSP00000270142 9606.ENSP00000381607 0 0 0 0 0.063 0.129 0 0.47 0.53

SOD1 TXNRD2 9606.ENSP00000270142 9606.ENSP00000383365 0 0 0 0 0.062 0.17 0 0.446 0.531

SOD1 GCLM 9606.ENSP00000270142 9606.ENSP00000359258 0 0 0 0 0.11 0.056 0 0.547 0.586

SOD1 NOX1 9606.ENSP00000270142 9606.ENSP00000362057 0 0 0 0 0 0.153 0 0.542 0.595

SOD1 GLRX2 9606.ENSP00000270142 9606.ENSP00000356410 0 0 0 0 0.082 0 0 0.618 0.634

SOD1 NQO1 9606.ENSP00000270142 9606.ENSP00000319788 0 0 0 0 0.062 0 0 0.638 0.646

SOD1 NFE2L2 9606.ENSP00000270142 9606.ENSP00000380252 0 0 0 0 0 0 0 0.704 0.704

SOD1 CYBB 9606.ENSP00000270142 9606.ENSP00000367851 0 0 0 0 0 0.179 0 0.658 0.707

SOD1 PRDX6 9606.ENSP00000270142 9606.ENSP00000342026 0 0 0 0 0.136 0.152 0 0.636 0.711

SOD1 TXNRD1 9606.ENSP00000270142 9606.ENSP00000434516 0 0 0 0 0.068 0.17 0 0.722 0.766

SOD1 ATOX1 9606.ENSP00000270142 9606.ENSP00000430598 0 0 0 0 0.075 0.495 0 0.772 0.884

SOD1 SOD3 9606.ENSP00000270142 9606.ENSP00000371554 0 0 0.281 0.865 0 0 0.9 0.753 0.913

SOD1 GPX4 9606.ENSP00000270142 9606.ENSP00000346103 0.044 0 0 0 0.138 0.376 0 0.86 0.918

SOD1 PRDX2 9606.ENSP00000270142 9606.ENSP00000301522 0 0 0 0 0.274 0.708 0 0.65 0.919

SOD1 TXN 9606.ENSP00000270142 9606.ENSP00000363641 0 0 0 0 0.431 0.353 0.65 0.74 0.962

SOD1 GPX3 9606.ENSP00000270142 9606.ENSP00000373477 0.044 0 0 0 0.094 0.178 0.9 0.627 0.968

SOD1 PARK7 9606.ENSP00000270142 9606.ENSP00000418770 0.046 0 0 0 0.372 0.704 0 0.847 0.969

SOD1 GPX1 9606.ENSP00000270142 9606.ENSP00000407375 0.044 0 0 0 0.094 0.178 0.9 0.838 0.986

SOD1 SOD2 9606.ENSP00000270142 9606.ENSP00000446252 0 0 0 0 0.168 0.943 0.9 0.901 0.999

SOD2 HMOX1 9606.ENSP00000446252 9606.ENSP00000216117 0.071 0 0 0 0.064 0 0 0.722 0.737

SOD2 TXN2 9606.ENSP00000446252 9606.ENSP00000216185 0 0 0 0 0.073 0 0 0.641 0.653

SOD2 GSR 9606.ENSP00000446252 9606.ENSP00000221130 0.07 0 0 0 0.11 0 0 0.768 0.791

SOD2 GCLC 9606.ENSP00000446252 9606.ENSP00000229416 0 0 0 0 0.067 0.731 0 0.816 0.949

SOD2 MAPK14 9606.ENSP00000446252 9606.ENSP00000229795 0 0 0 0 0.062 0.132 0 0.412 0.48

SOD2 CAT 9606.ENSP00000446252 9606.ENSP00000241052 0.07 0 0 0 0.079 0.401 0.9 0.943 0.996

SOD2 CYBA 9606.ENSP00000446252 9606.ENSP00000261623 0 0 0 0 0 0 0 0.45 0.45

SOD2 NOX4 9606.ENSP00000446252 9606.ENSP00000263317 0 0 0 0 0.065 0 0 0.631 0.64

SOD2 PPARGC1A 9606.ENSP00000446252 9606.ENSP00000264867 0 0 0 0 0 0 0.8 0.736 0.945

SOD2 PRDX5 9606.ENSP00000446252 9606.ENSP00000265462 0 0 0 0 0.264 0.31 0 0.802 0.89

SOD2 SOD1 9606.ENSP00000446252 9606.ENSP00000270142 0 0 0 0 0.168 0.943 0.9 0.901 0.999

SOD2 NOS3 9606.ENSP00000446252 9606.ENSP00000297494 0 0 0 0 0.062 0 0 0.636 0.644

SOD2 PRDX2 9606.ENSP00000446252 9606.ENSP00000301522 0.154 0 0 0 0.112 0 0 0.628 0.696

SOD2 GSTM1 9606.ENSP00000446252 9606.ENSP00000311469 0 0 0 0 0.064 0 0 0.54 0.551

SOD2 UCP2 9606.ENSP00000446252 9606.ENSP00000312029 0 0 0 0 0.062 0 0 0.649 0.657

SOD2 MSRA 9606.ENSP00000446252 9606.ENSP00000313921 0.295 0 0 0 0.064 0 0 0.499 0.64

SOD2 NDUFS8 9606.ENSP00000446252 9606.ENSP00000315774 0.046 0 0 0 0.483 0 0 0.166 0.553

SOD2 DUOX1 9606.ENSP00000446252 9606.ENSP00000317997 0 0 0 0 0.065 0 0 0.424 0.438

SOD2 NQO1 9606.ENSP00000446252 9606.ENSP00000319788 0 0 0 0 0 0 0 0.647 0.647

SOD2 NOS2 9606.ENSP00000446252 9606.ENSP00000327251 0 0 0 0 0.062 0 0 0.442 0.454

SOD2 GSTA1 9606.ENSP00000446252 9606.ENSP00000335620 0 0 0 0 0.064 0 0 0.401 0.415

SOD2 PRDX6 9606.ENSP00000446252 9606.ENSP00000342026 0.154 0 0 0 0.233 0 0 0.686 0.778

SOD2 GPX4 9606.ENSP00000446252 9606.ENSP00000346103 0 0 0 0 0.064 0.539 0 0.843 0.926

SOD2 GLRX2 9606.ENSP00000446252 9606.ENSP00000356410 0.058 0 0 0 0.152 0 0 0.491 0.558

SOD2 NCF2 9606.ENSP00000446252 9606.ENSP00000356505 0 0 0 0 0.076 0 0 0.391 0.414

SOD2 GCLM 9606.ENSP00000446252 9606.ENSP00000359258 0 0 0 0 0.066 0.066 0 0.535 0.559

SOD2 MMP9 9606.ENSP00000446252 9606.ENSP00000361405 0 0 0 0 0.069 0 0 0.395 0.413

SOD2 NOX1 9606.ENSP00000446252 9606.ENSP00000362057 0 0 0 0 0.065 0 0 0.508 0.521

SOD2 TXN 9606.ENSP00000446252 9606.ENSP00000363641 0 0 0 0 0.409 0 0 0.714 0.823

SOD2 MTHFR 9606.ENSP00000446252 9606.ENSP00000365777 0.05 0 0 0 0.062 0.078 0 0.51 0.543

SOD2 CYBB 9606.ENSP00000446252 9606.ENSP00000367851 0 0 0 0 0.093 0 0 0.601 0.623

SOD2 SRXN1 9606.ENSP00000446252 9606.ENSP00000371388 0 0 0 0 0.064 0 0 0.408 0.422

SOD2 SOD3 9606.ENSP00000446252 9606.ENSP00000371554 0 0 0 0 0.112 0.744 0.9 0.785 0.994

SOD2 GPX3 9606.ENSP00000446252 9606.ENSP00000373477 0 0 0 0 0.062 0.254 0.9 0.646 0.971

SOD2 NFE2L2 9606.ENSP00000446252 9606.ENSP00000380252 0 0 0 0 0 0 0 0.646 0.646

SOD2 GSTP1 9606.ENSP00000446252 9606.ENSP00000381607 0 0 0 0 0.065 0 0 0.575 0.586

SOD2 TXNRD2 9606.ENSP00000446252 9606.ENSP00000383365 0.07 0 0 0 0.108 0 0 0.597 0.636

SOD2 GPX1 9606.ENSP00000446252 9606.ENSP00000407375 0 0 0 0 0.062 0.254 0.9 0.856 0.988

SOD2 PARK7 9606.ENSP00000446252 9606.ENSP00000418770 0.165 0 0 0 0.494 0 0 0.621 0.825

SOD2 ATOX1 9606.ENSP00000446252 9606.ENSP00000430598 0 0 0 0 0.107 0.243 0 0.266 0.46

SOD2 TXNRD1 9606.ENSP00000446252 9606.ENSP00000434516 0.247 0 0 0 0.146 0 0 0.75 0.825

SOD2 NOS1 9606.ENSP00000446252 9606.ENSP00000477999 0 0 0 0 0.062 0 0 0.438 0.45

SOD3 NOX3 9606.ENSP00000371554 9606.ENSP00000159060 0 0 0 0 0 0.104 0 0.408 0.447

SOD3 HMOX1 9606.ENSP00000371554 9606.ENSP00000216117 0 0 0 0 0 0 0 0.759 0.76

SOD3 TXN2 9606.ENSP00000371554 9606.ENSP00000216185 0 0 0 0 0.064 0.157 0 0.34 0.433

SOD3 GSR 9606.ENSP00000371554 9606.ENSP00000221130 0 0 0 0 0.055 0.117 0 0.669 0.7

SOD3 GCLC 9606.ENSP00000371554 9606.ENSP00000229416 0 0 0 0 0.052 0.271 0 0.521 0.64

SOD3 CAT 9606.ENSP00000371554 9606.ENSP00000241052 0.111 0 0 0 0.124 0.339 0.9 0.845 0.99

SOD3 CYBA 9606.ENSP00000371554 9606.ENSP00000261623 0 0 0 0 0 0 0 0.508 0.508

SOD3 NOX4 9606.ENSP00000371554 9606.ENSP00000263317 0 0 0 0 0.062 0.104 0 0.594 0.629

SOD3 PPARGC1A 9606.ENSP00000371554 9606.ENSP00000264867 0 0 0 0 0.07 0 0 0.722 0.73

SOD3 PRDX5 9606.ENSP00000371554 9606.ENSP00000265462 0.048 0 0 0 0.132 0 0 0.505 0.555

SOD3 SOD1 9606.ENSP00000371554 9606.ENSP00000270142 0 0 0.281 0.865 0 0 0.9 0.753 0.913

SOD3 NOS3 9606.ENSP00000371554 9606.ENSP00000297494 0 0 0 0 0.062 0 0 0.613 0.621

SOD3 PRDX2 9606.ENSP00000371554 9606.ENSP00000301522 0 0 0 0 0.111 0.068 0 0.39 0.451

SOD3 DUOX1 9606.ENSP00000371554 9606.ENSP00000317997 0 0 0 0 0 0.104 0 0.431 0.469

SOD3 PRDX6 9606.ENSP00000371554 9606.ENSP00000342026 0 0 0 0 0.111 0.068 0 0.545 0.59

SOD3 GPX4 9606.ENSP00000371554 9606.ENSP00000346103 0.044 0 0 0 0.094 0.178 0 0.547 0.634

SOD3 NCF2 9606.ENSP00000371554 9606.ENSP00000356505 0 0 0 0 0 0 0 0.454 0.454

SOD3 NOX1 9606.ENSP00000371554 9606.ENSP00000362057 0 0 0 0 0.062 0.104 0 0.572 0.609

SOD3 TXN 9606.ENSP00000371554 9606.ENSP00000363641 0 0 0 0 0.097 0.149 0 0.518 0.597

SOD3 MTHFR 9606.ENSP00000371554 9606.ENSP00000365777 0.115 0 0 0 0 0.082 0 0.354 0.43

SOD3 CYBB 9606.ENSP00000371554 9606.ENSP00000367851 0 0 0 0 0 0.104 0 0.62 0.645

SOD3 DUOX2 9606.ENSP00000371554 9606.ENSP00000475084 0 0 0 0 0 0.104 0 0.361 0.403

SOD3 NOS1 9606.ENSP00000371554 9606.ENSP00000477999 0 0 0 0 0.049 0 0 0.402 0.407

SOD3 NFE2L2 9606.ENSP00000371554 9606.ENSP00000380252 0 0 0 0 0 0 0 0.419 0.419

SOD3 TXNRD2 9606.ENSP00000371554 9606.ENSP00000383365 0 0 0 0 0.055 0.149 0 0.374 0.452

SOD3 NOX5 9606.ENSP00000371554 9606.ENSP00000373518 0 0 0 0 0 0.104 0 0.417 0.455

SOD3 HMOX2 9606.ENSP00000371554 9606.ENSP00000477572 0 0 0 0 0 0 0 0.488 0.488

SOD3 PARK7 9606.ENSP00000371554 9606.ENSP00000418770 0.046 0 0 0 0.093 0.17 0 0.396 0.508

SOD3 TXNRD1 9606.ENSP00000371554 9606.ENSP00000434516 0 0 0 0 0.063 0.149 0 0.577 0.633

SOD3 GPX3 9606.ENSP00000371554 9606.ENSP00000373477 0.044 0 0 0 0.161 0.178 0.9 0.609 0.969

SOD3 GPX1 9606.ENSP00000371554 9606.ENSP00000407375 0.044 0 0 0 0.094 0.178 0.9 0.64 0.969

SOD3 ATOX1 9606.ENSP00000371554 9606.ENSP00000430598 0 0 0 0 0.062 0.336 0.9 0.722 0.98

SOD3 SOD2 9606.ENSP00000371554 9606.ENSP00000446252 0 0 0 0 0.112 0.744 0.9 0.785 0.994

SP1 COL1A1 9606.ENSP00000329357 9606.ENSP00000225964 0 0 0 0 0 0.282 0 0.676 0.757

SP1 NFKB1 9606.ENSP00000329357 9606.ENSP00000226574 0 0 0 0 0.079 0.687 0 0.259 0.768

SP1 MAPK14 9606.ENSP00000329357 9606.ENSP00000229795 0 0 0 0 0 0.34 0.9 0.204 0.942

SP1 MMP10 9606.ENSP00000329357 9606.ENSP00000279441 0 0 0 0 0 0 0 0.416 0.416

SP1 ACOX1 9606.ENSP00000329357 9606.ENSP00000293217 0 0 0 0 0 0.057 0.9 0.048 0.902

SP1 NOS3 9606.ENSP00000329357 9606.ENSP00000297494 0 0 0 0 0 0.213 0 0.358 0.473

SP1 JUNB 9606.ENSP00000329357 9606.ENSP00000303315 0 0 0 0 0.046 0.101 0.9 0.29 0.93

SP1 FOS 9606.ENSP00000329357 9606.ENSP00000306245 0 0 0 0 0 0.062 0.9 0.501 0.949

SP1 NOS2 9606.ENSP00000329357 9606.ENSP00000327251 0 0 0 0 0 0 0.9 0.09 0.905

SP1 MMP9 9606.ENSP00000329357 9606.ENSP00000361405 0 0 0 0 0 0 0 0.406 0.406

SP1 TXN 9606.ENSP00000329357 9606.ENSP00000363641 0 0 0 0 0.049 0.057 0 0.444 0.458

SP1 NFE2L2 9606.ENSP00000329357 9606.ENSP00000380252 0 0 0 0 0 0.056 0 0.478 0.486

SP1 CDKN2A 9606.ENSP00000329357 9606.ENSP00000418915 0 0 0 0 0.049 0.298 0 0.643 0.741

SP1 MAPK10 9606.ENSP00000329357 9606.ENSP00000352157 0 0 0 0 0 0.062 0.9 0.094 0.907

SRXN1 HMOX1 9606.ENSP00000371388 9606.ENSP00000216117 0 0 0 0 0.107 0 0 0.631 0.657

SRXN1 TXN2 9606.ENSP00000371388 9606.ENSP00000216185 0 0 0 0 0.209 0.062 0 0.34 0.468

SRXN1 GSR 9606.ENSP00000371388 9606.ENSP00000221130 0 0 0 0 0.256 0 0 0.728 0.789

SRXN1 GCLC 9606.ENSP00000371388 9606.ENSP00000229416 0 0 0 0 0.233 0 0 0.7 0.76

SRXN1 CAT 9606.ENSP00000371388 9606.ENSP00000241052 0 0 0 0 0.344 0 0 0.736 0.819

SRXN1 SESN2 9606.ENSP00000371388 9606.ENSP00000253063 0 0 0 0 0.069 0 0 0.415 0.432

SRXN1 PRDX5 9606.ENSP00000371388 9606.ENSP00000265462 0 0 0 0 0.062 0 0 0.783 0.788

SRXN1 SOD1 9606.ENSP00000371388 9606.ENSP00000270142 0 0 0 0 0.112 0 0 0.403 0.447

SRXN1 PRDX2 9606.ENSP00000371388 9606.ENSP00000301522 0 0 0 0 0.067 0.255 0 0.682 0.759

SRXN1 NQO1 9606.ENSP00000371388 9606.ENSP00000319788 0 0 0 0 0.092 0 0 0.686 0.703

SRXN1 PRDX6 9606.ENSP00000371388 9606.ENSP00000342026 0 0 0 0 0.083 0.239 0 0.558 0.665

SRXN1 GPX4 9606.ENSP00000371388 9606.ENSP00000346103 0 0 0 0 0.062 0 0 0.658 0.666

SRXN1 GLRX2 9606.ENSP00000371388 9606.ENSP00000356410 0 0 0 0 0.166 0 0 0.438 0.511

SRXN1 GCLM 9606.ENSP00000371388 9606.ENSP00000359258 0 0 0 0 0.101 0 0 0.707 0.725

SRXN1 TXN 9606.ENSP00000371388 9606.ENSP00000363641 0 0 0 0 0.38 0.11 0 0.737 0.842

SRXN1 AOX1 9606.ENSP00000371388 9606.ENSP00000363832 0 0 0 0 0 0 0 0.434 0.434

SRXN1 SOD2 9606.ENSP00000371388 9606.ENSP00000446252 0 0 0 0 0.064 0 0 0.408 0.422

SRXN1 GPX3 9606.ENSP00000371388 9606.ENSP00000373477 0 0 0 0 0.06 0 0 0.47 0.48

SRXN1 TXNRD2 9606.ENSP00000371388 9606.ENSP00000383365 0 0 0 0 0.122 0 0 0.444 0.491

SRXN1 GPX1 9606.ENSP00000371388 9606.ENSP00000407375 0 0 0 0 0.06 0 0 0.552 0.56

SRXN1 NFE2L2 9606.ENSP00000371388 9606.ENSP00000380252 0 0 0 0 0 0 0 0.575 0.575

SRXN1 TXNRD1 9606.ENSP00000371388 9606.ENSP00000434516 0 0 0 0 0.27 0 0 0.79 0.84

SUOX DAO 9606.ENSP00000377668 9606.ENSP00000228476 0 0 0 0 0.062 0 0 0.422 0.434

SUOX CAT 9606.ENSP00000377668 9606.ENSP00000241052 0 0.01 0 0 0.065 0 0 0.604 0.614

SUOX MSRA 9606.ENSP00000377668 9606.ENSP00000313921 0.186 0 0 0 0 0 0 0.338 0.438

SUOX CTH 9606.ENSP00000377668 9606.ENSP00000359976 0 0 0 0 0.064 0 0 0.639 0.648

SUOX DHCR24 9606.ENSP00000377668 9606.ENSP00000360316 0 0 0 0 0 0.709 0 0.044 0.709

SUOX TXN 9606.ENSP00000377668 9606.ENSP00000363641 0.045 0 0 0 0 0.368 0 0.144 0.438

SUOX AOX1 9606.ENSP00000377668 9606.ENSP00000363832 0 0 0 0 0 0 0 0.876 0.876

SUOX PRNP 9606.ENSP00000377668 9606.ENSP00000368752 0 0 0 0 0 0 0 0.489 0.489

SUOX GPX1 9606.ENSP00000377668 9606.ENSP00000407375 0.102 0 0 0 0 0 0 0.461 0.495

SUOX ATOX1 9606.ENSP00000377668 9606.ENSP00000430598 0 0 0 0 0 0 0 0.648 0.648

SUOX TXNRD2 9606.ENSP00000377668 9606.ENSP00000383365 0.07 0 0 0 0 0 0 0.643 0.654

SUOX TXNRD1 9606.ENSP00000377668 9606.ENSP00000434516 0.254 0 0 0 0 0 0 0.672 0.745

TIMP2 COL1A1 9606.ENSP00000262768 9606.ENSP00000225964 0 0 0 0 0.118 0 0 0.575 0.61

TIMP2 LOX 9606.ENSP00000262768 9606.ENSP00000231004 0 0 0 0 0.131 0 0 0.399 0.456

TIMP2 MMP8 9606.ENSP00000262768 9606.ENSP00000236826 0 0 0 0 0.064 0.327 0 0.79 0.857

TIMP2 ADAMTS2 9606.ENSP00000262768 9606.ENSP00000251582 0 0 0 0 0.096 0 0 0.397 0.432

TIMP2 AOC2 9606.ENSP00000262768 9606.ENSP00000253799 0 0 0 0 0 0 0 0.63 0.63

TIMP2 MMP13 9606.ENSP00000262768 9606.ENSP00000260302 0 0 0 0 0 0.702 0 0.925 0.976

TIMP2 RUNX2 9606.ENSP00000262768 9606.ENSP00000360493 0 0 0 0 0 0 0 0.45 0.45

TIMP2 ACAN 9606.ENSP00000262768 9606.ENSP00000387356 0 0 0 0 0.062 0 0 0.555 0.565

TIMP2 MMP19 9606.ENSP00000262768 9606.ENSP00000313437 0 0 0 0 0.074 0.18 0 0.481 0.571

TIMP2 TIMP3 9606.ENSP00000262768 9606.ENSP00000266085 0 0 0 0.925 0.133 0.684 0 0.87 0.732

TIMP2 MMP9 9606.ENSP00000262768 9606.ENSP00000361405 0 0 0 0 0.068 0.18 0 0.923 0.936

TIMP2 MMP1 9606.ENSP00000262768 9606.ENSP00000322788 0 0 0 0 0.098 0.18 0 0.937 0.95

TIMP2 MMP3 9606.ENSP00000262768 9606.ENSP00000299855 0 0 0 0 0.091 0.18 0 0.958 0.966

TIMP2 MMP10 9606.ENSP00000262768 9606.ENSP00000279441 0 0 0 0 0.064 0.828 0 0.816 0.968

TIMP2 MMP14 9606.ENSP00000262768 9606.ENSP00000308208 0 0 0 0 0.153 0.884 0.9 0.99 0.999

TIMP3 COL1A1 9606.ENSP00000266085 9606.ENSP00000225964 0 0 0 0 0.219 0 0 0.538 0.624

TIMP3 LOX 9606.ENSP00000266085 9606.ENSP00000231004 0 0 0 0 0.214 0 0 0.394 0.503

TIMP3 MMP8 9606.ENSP00000266085 9606.ENSP00000236826 0 0 0 0 0 0.18 0 0.642 0.693

TIMP3 ADAMTS2 9606.ENSP00000266085 9606.ENSP00000251582 0 0 0 0 0.107 0 0 0.422 0.463

TIMP3 MMP13 9606.ENSP00000266085 9606.ENSP00000260302 0 0 0 0 0 0.327 0 0.7 0.79

TIMP3 TIMP2 9606.ENSP00000266085 9606.ENSP00000262768 0 0 0 0.925 0.133 0.684 0 0.87 0.732

TIMP3 AOC3 9606.ENSP00000266085 9606.ENSP00000312326 0 0 0 0 0.061 0 0 0.427 0.439

TIMP3 GSTP1 9606.ENSP00000266085 9606.ENSP00000381607 0 0 0 0 0 0 0 0.617 0.617

TIMP3 MMP19 9606.ENSP00000266085 9606.ENSP00000313437 0 0 0 0 0.076 0.249 0 0.508 0.629

TIMP3 CDKN2A 9606.ENSP00000266085 9606.ENSP00000418915 0 0 0 0 0 0 0 0.632 0.632

TIMP3 ACAN 9606.ENSP00000266085 9606.ENSP00000387356 0 0 0 0 0.062 0 0 0.63 0.638

TIMP3 MMP10 9606.ENSP00000266085 9606.ENSP00000279441 0 0 0 0 0 0.18 0 0.629 0.682

TIMP3 MMP1 9606.ENSP00000266085 9606.ENSP00000322788 0 0 0 0 0.088 0.18 0 0.818 0.852

TIMP3 MMP14 9606.ENSP00000266085 9606.ENSP00000308208 0 0 0 0 0.183 0.383 0 0.784 0.881

TIMP3 MMP3 9606.ENSP00000266085 9606.ENSP00000299855 0 0 0 0 0.088 0.568 0 0.837 0.93

TIMP3 MMP9 9606.ENSP00000266085 9606.ENSP00000361405 0 0 0 0 0 0.327 0.8 0.914 0.987

TIMP4 MMP8 9606.ENSP00000287814 9606.ENSP00000236826 0 0 0 0 0 0.18 0 0.688 0.733

TIMP4 MMP13 9606.ENSP00000287814 9606.ENSP00000260302 0 0 0 0 0 0.18 0 0.64 0.692

TIMP4 MMP10 9606.ENSP00000287814 9606.ENSP00000279441 0 0 0 0 0 0.18 0 0.587 0.647

TIMP4 MMP19 9606.ENSP00000287814 9606.ENSP00000313437 0 0 0 0 0 0.18 0 0.43 0.512

TIMP4 MMP1 9606.ENSP00000287814 9606.ENSP00000322788 0 0 0 0 0 0.18 0 0.659 0.708

TIMP4 MMP3 9606.ENSP00000287814 9606.ENSP00000299855 0 0 0 0 0 0.18 0 0.691 0.736

TIMP4 MMP9 9606.ENSP00000287814 9606.ENSP00000361405 0 0 0 0 0 0.18 0 0.793 0.823

TIMP4 MMP14 9606.ENSP00000287814 9606.ENSP00000308208 0 0 0 0 0 0.327 0 0.755 0.829

TXN HMOX1 9606.ENSP00000363641 9606.ENSP00000216117 0 0 0 0 0 0 0 0.709 0.709

TXN TXN2 9606.ENSP00000363641 9606.ENSP00000216185 0 0 0.359 0.846 0.241 0.477 0.8 0.842 0.928

TXN GSS 9606.ENSP00000363641 9606.ENSP00000216951 0 0 0 0 0.09 0.119 0 0.47 0.538

TXN GSR 9606.ENSP00000363641 9606.ENSP00000221130 0.051 0 0 0 0.246 0.338 0 0.905 0.949

TXN NFKB1 9606.ENSP00000363641 9606.ENSP00000226574 0 0 0 0 0.052 0.934 0 0.178 0.944

TXN GCLC 9606.ENSP00000363641 9606.ENSP00000229416 0 0 0 0 0.088 0.103 0 0.825 0.845

TXN MAPK14 9606.ENSP00000363641 9606.ENSP00000229795 0 0 0 0 0.053 0.076 0 0.416 0.445

TXN CAT 9606.ENSP00000363641 9606.ENSP00000241052 0 0 0 0 0.138 0.137 0 0.92 0.935

TXN GFER 9606.ENSP00000363641 9606.ENSP00000248114 0 0 0 0 0.062 0.274 0 0.632 0.727

TXN GSTM3 9606.ENSP00000363641 9606.ENSP00000256594 0 0 0 0 0 0 0 0.47 0.47

TXN CYBA 9606.ENSP00000363641 9606.ENSP00000261623 0 0 0 0 0.064 0 0 0.449 0.462

TXN NOX4 9606.ENSP00000363641 9606.ENSP00000263317 0 0 0 0 0.062 0.057 0 0.551 0.568

TXN PPARGC1A 9606.ENSP00000363641 9606.ENSP00000264867 0 0 0 0 0 0 0 0.422 0.422

TXN PRDX5 9606.ENSP00000363641 9606.ENSP00000265462 0 0 0 0 0.107 0.451 0.9 0.718 0.984

TXN SOD1 9606.ENSP00000363641 9606.ENSP00000270142 0 0 0 0 0.431 0.353 0.65 0.74 0.962

TXN NOS3 9606.ENSP00000363641 9606.ENSP00000297494 0 0 0 0 0.062 0 0 0.531 0.541

TXN PRDX2 9606.ENSP00000363641 9606.ENSP00000301522 0.045 0 0 0 0.079 0.78 0.9 0.896 0.997

TXN UCP2 9606.ENSP00000363641 9606.ENSP00000312029 0 0 0 0 0 0.062 0 0.391 0.405

TXN MSRA 9606.ENSP00000363641 9606.ENSP00000313921 0.071 0 0 0 0.055 0.774 0.9 0.613 0.99

TXN DUOX1 9606.ENSP00000363641 9606.ENSP00000317997 0 0 0 0 0.062 0.057 0 0.557 0.575

TXN NQO1 9606.ENSP00000363641 9606.ENSP00000319788 0 0 0 0 0.098 0 0 0.661 0.682

TXN SP1 9606.ENSP00000363641 9606.ENSP00000329357 0 0 0 0 0.049 0.057 0 0.444 0.458

TXN PRDX6 9606.ENSP00000363641 9606.ENSP00000342026 0.045 0 0 0 0.126 0.226 0 0.807 0.858

TXN GPX4 9606.ENSP00000363641 9606.ENSP00000346103 0 0 0 0 0.087 0.212 0 0.828 0.865

TXN QSOX2 9606.ENSP00000363641 9606.ENSP00000351536 0 0 0 0 0 0 0 0.669 0.669

TXN GLRX2 9606.ENSP00000363641 9606.ENSP00000356410 0.048 0 0 0 0.265 0.094 0 0.873 0.908

TXN QSOX1 9606.ENSP00000363641 9606.ENSP00000356574 0 0 0 0 0 0 0 0.651 0.651

TXN GCLM 9606.ENSP00000363641 9606.ENSP00000359258 0 0 0 0 0.063 0.056 0 0.692 0.704

TXN CTH 9606.ENSP00000363641 9606.ENSP00000359976 0 0 0 0 0.062 0.06 0 0.464 0.486

TXN NOX1 9606.ENSP00000363641 9606.ENSP00000362057 0 0 0 0 0.062 0.057 0 0.514 0.532

TXN VIMP 9606.ENSP00000363641 9606.ENSP00000381282 0 0 0 0 0.092 0 0 0.374 0.408

TXN DUOX2 9606.ENSP00000363641 9606.ENSP00000475084 0 0 0 0 0.062 0.057 0 0.387 0.41

TXN NOS1 9606.ENSP00000363641 9606.ENSP00000477999 0 0 0 0 0.062 0 0 0.421 0.434

TXN SUOX 9606.ENSP00000363641 9606.ENSP00000377668 0.045 0 0 0 0 0.368 0 0.144 0.438

TXN NOX5 9606.ENSP00000363641 9606.ENSP00000373518 0 0 0 0 0.062 0.057 0 0.429 0.45

TXN SEPP1 9606.ENSP00000363641 9606.ENSP00000420939 0 0 0 0 0 0 0 0.511 0.511

TXN GSTP1 9606.ENSP00000363641 9606.ENSP00000381607 0 0 0 0 0.07 0.117 0 0.472 0.529

TXN SOD3 9606.ENSP00000363641 9606.ENSP00000371554 0 0 0 0 0.097 0.149 0 0.518 0.597

TXN CYBB 9606.ENSP00000363641 9606.ENSP00000367851 0 0 0 0 0.062 0.057 0 0.634 0.648

TXN NFE2L2 9606.ENSP00000363641 9606.ENSP00000380252 0 0 0 0 0 0 0 0.681 0.681

TXN GPX3 9606.ENSP00000363641 9606.ENSP00000373477 0 0 0 0 0.062 0.212 0 0.732 0.785

TXN GPX1 9606.ENSP00000363641 9606.ENSP00000407375 0 0 0 0 0.063 0.284 0 0.728 0.802

TXN PARK7 9606.ENSP00000363641 9606.ENSP00000418770 0 0 0 0 0.183 0.079 0 0.76 0.804

TXN SOD2 9606.ENSP00000363641 9606.ENSP00000446252 0 0 0 0 0.409 0 0 0.714 0.823

TXN SRXN1 9606.ENSP00000363641 9606.ENSP00000371388 0 0 0 0 0.38 0.11 0 0.737 0.842

TXN TXNRD2 9606.ENSP00000363641 9606.ENSP00000383365 0.051 0 0 0 0.078 0.243 0 0.861 0.895

TXN TXNRD1 9606.ENSP00000363641 9606.ENSP00000434516 0.179 0 0 0 0.167 0.842 0.9 0.963 0.999

TXN2 GCLC 9606.ENSP00000216185 9606.ENSP00000229416 0 0 0 0 0.062 0.06 0 0.376 0.402

TXN2 PPARGC1A 9606.ENSP00000216185 9606.ENSP00000264867 0 0 0 0 0 0 0 0.406 0.406

TXN2 GCLM 9606.ENSP00000216185 9606.ENSP00000359258 0 0 0 0 0.063 0.056 0 0.401 0.424

TXN2 SOD3 9606.ENSP00000216185 9606.ENSP00000371554 0 0 0 0 0.064 0.157 0 0.34 0.433

TXN2 NOX4 9606.ENSP00000216185 9606.ENSP00000263317 0 0 0 0 0.062 0.057 0 0.425 0.447

TXN2 SRXN1 9606.ENSP00000216185 9606.ENSP00000371388 0 0 0 0 0.209 0.062 0 0.34 0.468

TXN2 GPX3 9606.ENSP00000216185 9606.ENSP00000373477 0 0 0 0 0.062 0.141 0 0.489 0.552

TXN2 SOD2 9606.ENSP00000216185 9606.ENSP00000446252 0 0 0 0 0.073 0 0 0.641 0.653

TXN2 GSR 9606.ENSP00000216185 9606.ENSP00000221130 0.051 0 0 0 0.078 0.22 0 0.575 0.671

TXN2 GPX4 9606.ENSP00000216185 9606.ENSP00000346103 0 0 0 0 0.065 0.141 0 0.626 0.673

TXN2 SOD1 9606.ENSP00000216185 9606.ENSP00000270142 0 0 0 0 0.104 0.36 0 0.488 0.68

TXN2 GPX1 9606.ENSP00000216185 9606.ENSP00000407375 0 0 0 0 0.062 0.141 0 0.66 0.702

TXN2 PRDX6 9606.ENSP00000216185 9606.ENSP00000342026 0.045 0 0 0 0.062 0.226 0 0.629 0.708

TXN2 CAT 9606.ENSP00000216185 9606.ENSP00000241052 0 0 0 0 0.122 0.151 0 0.656 0.721

TXN2 GLRX2 9606.ENSP00000216185 9606.ENSP00000356410 0.048 0 0 0 0.1 0.057 0 0.712 0.737

TXN2 MSRA 9606.ENSP00000216185 9606.ENSP00000313921 0.071 0 0 0 0.055 0.248 0 0.686 0.765

TXN2 PRDX2 9606.ENSP00000216185 9606.ENSP00000301522 0.045 0 0 0 0.112 0.464 0 0.558 0.772

TXN2 TXNRD1 9606.ENSP00000216185 9606.ENSP00000434516 0.179 0 0 0 0.134 0.245 0 0.69 0.811

TXN2 TXN 9606.ENSP00000216185 9606.ENSP00000363641 0 0 0.359 0.846 0.241 0.477 0.8 0.842 0.928

TXN2 PRDX5 9606.ENSP00000216185 9606.ENSP00000265462 0 0 0 0 0.067 0.301 0.9 0.776 0.983

TXN2 TXNRD2 9606.ENSP00000216185 9606.ENSP00000383365 0.051 0 0 0 0.078 0.22 0.9 0.926 0.994

TXNRD1 HMOX1 9606.ENSP00000434516 9606.ENSP00000216117 0 0 0 0 0.066 0.128 0 0.706 0.739

TXNRD1 TXN2 9606.ENSP00000434516 9606.ENSP00000216185 0.179 0 0 0 0.134 0.245 0 0.69 0.811

TXNRD1 GSS 9606.ENSP00000434516 9606.ENSP00000216951 0 0 0 0 0.096 0.261 0 0.251 0.456

TXNRD1 GSR 9606.ENSP00000434516 9606.ENSP00000221130 0.07 0 0.428 0.809 0.203 0.15 0 0.916 0.478

TXNRD1 DAO 9606.ENSP00000434516 9606.ENSP00000228476 0.185 0 0 0 0.062 0.175 0 0.174 0.409

TXNRD1 GCLC 9606.ENSP00000434516 9606.ENSP00000229416 0 0 0 0 0.186 0 0 0.894 0.91

TXNRD1 CAT 9606.ENSP00000434516 9606.ENSP00000241052 0.142 0 0 0 0.171 0.363 0 0.804 0.899

TXNRD1 NOX4 9606.ENSP00000434516 9606.ENSP00000263317 0 0 0 0 0 0 0 0.435 0.435

TXNRD1 PRDX5 9606.ENSP00000434516 9606.ENSP00000265462 0.288 0 0 0 0.125 0 0 0.751 0.831

TXNRD1 SOD1 9606.ENSP00000434516 9606.ENSP00000270142 0 0 0 0 0.068 0.17 0 0.722 0.766

TXNRD1 GSTT2B 9606.ENSP00000434516 9606.ENSP00000290765 0 0 0 0 0.112 0.174 0 0.375 0.502

TXNRD1 PRDX2 9606.ENSP00000434516 9606.ENSP00000301522 0.18 0 0 0 0.109 0.154 0 0.686 0.78

TXNRD1 GSTM1 9606.ENSP00000434516 9606.ENSP00000311469 0 0 0 0 0.081 0 0 0.491 0.513

TXNRD1 MSRA 9606.ENSP00000434516 9606.ENSP00000313921 0.207 0 0 0 0 0.291 0 0.678 0.803

TXNRD1 NQO1 9606.ENSP00000434516 9606.ENSP00000319788 0 0 0 0 0.152 0 0 0.729 0.761

TXNRD1 GSTA1 9606.ENSP00000434516 9606.ENSP00000335620 0 0 0 0 0.081 0 0 0.493 0.514

TXNRD1 PRDX6 9606.ENSP00000434516 9606.ENSP00000342026 0.18 0 0 0 0.131 0.18 0 0.719 0.814

TXNRD1 GPX4 9606.ENSP00000434516 9606.ENSP00000346103 0.23 0 0 0 0.144 0.167 0 0.847 0.904

TXNRD1 GLRX2 9606.ENSP00000434516 9606.ENSP00000356410 0.07 0 0 0.73 0.083 0.427 0 0.655 0.561

TXNRD1 GCLM 9606.ENSP00000434516 9606.ENSP00000359258 0.048 0 0 0 0.135 0.06 0 0.792 0.817

TXNRD1 CTH 9606.ENSP00000434516 9606.ENSP00000359976 0.076 0 0 0 0.065 0 0.9 0.41 0.942

TXNRD1 TXN 9606.ENSP00000434516 9606.ENSP00000363641 0.179 0 0 0 0.167 0.842 0.9 0.963 0.999

TXNRD1 AOX1 9606.ENSP00000434516 9606.ENSP00000363832 0 0 0 0 0 0 0 0.531 0.531

TXNRD1 MTHFR 9606.ENSP00000434516 9606.ENSP00000365777 0.183 0 0 0 0.062 0 0 0.432 0.527

TXNRD1 SRXN1 9606.ENSP00000434516 9606.ENSP00000371388 0 0 0 0 0.27 0 0 0.79 0.84

TXNRD1 SOD3 9606.ENSP00000434516 9606.ENSP00000371554 0 0 0 0 0.063 0.149 0 0.577 0.633

TXNRD1 GPX3 9606.ENSP00000434516 9606.ENSP00000373477 0.23 0 0 0 0.144 0.167 0 0.767 0.855

TXNRD1 SUOX 9606.ENSP00000434516 9606.ENSP00000377668 0.254 0 0 0 0 0 0 0.672 0.745

TXNRD1 MGST1 9606.ENSP00000434516 9606.ENSP00000379512 0 0 0 0 0 0 0 0.448 0.448

TXNRD1 NFE2L2 9606.ENSP00000434516 9606.ENSP00000380252 0 0 0 0 0 0 0 0.661 0.661

TXNRD1 VIMP 9606.ENSP00000434516 9606.ENSP00000381282 0 0 0 0 0.06 0 0 0.716 0.721

TXNRD1 GSTP1 9606.ENSP00000434516 9606.ENSP00000381607 0 0 0 0 0.081 0 0 0.495 0.516

TXNRD1 TXNRD2 9606.ENSP00000434516 9606.ENSP00000383365 0.07 0 0.444 0.95 0.083 0.267 0.8 0.888 0.867

TXNRD1 GPX1 9606.ENSP00000434516 9606.ENSP00000407375 0.23 0 0 0 0.144 0.167 0 0.831 0.895

TXNRD1 PARK7 9606.ENSP00000434516 9606.ENSP00000418770 0.212 0 0 0 0.069 0 0 0.442 0.555

TXNRD1 SEPP1 9606.ENSP00000434516 9606.ENSP00000420939 0 0 0 0 0 0 0 0.756 0.756

TXNRD1 SOD2 9606.ENSP00000434516 9606.ENSP00000446252 0.247 0 0 0 0.146 0 0 0.75 0.825

TXNRD2 TXN2 9606.ENSP00000383365 9606.ENSP00000216185 0.051 0 0 0 0.078 0.22 0.9 0.926 0.994

TXNRD2 GSS 9606.ENSP00000383365 9606.ENSP00000216951 0 0 0 0 0.065 0.261 0 0.207 0.404

TXNRD2 GCLC 9606.ENSP00000383365 9606.ENSP00000229416 0 0 0 0 0.112 0 0 0.616 0.645

TXNRD2 CAT 9606.ENSP00000383365 9606.ENSP00000241052 0 0 0 0 0.109 0.222 0 0.634 0.724

TXNRD2 PPARGC1A 9606.ENSP00000383365 9606.ENSP00000264867 0 0 0 0 0 0 0 0.494 0.494

TXNRD2 PRDX5 9606.ENSP00000383365 9606.ENSP00000265462 0.166 0 0 0 0.091 0 0 0.527 0.61

TXNRD2 SOD1 9606.ENSP00000383365 9606.ENSP00000270142 0 0 0 0 0.062 0.17 0 0.446 0.531

TXNRD2 PRDX2 9606.ENSP00000383365 9606.ENSP00000301522 0 0 0 0 0.068 0.067 0 0.585 0.608

TXNRD2 MSRA 9606.ENSP00000383365 9606.ENSP00000313921 0.057 0 0 0 0 0 0 0.499 0.507

TXNRD2 PRDX6 9606.ENSP00000383365 9606.ENSP00000342026 0 0 0 0 0.068 0.067 0 0.452 0.482

TXNRD2 GPX4 9606.ENSP00000383365 9606.ENSP00000346103 0.05 0 0 0 0.063 0.15 0 0.828 0.852

TXNRD2 GLRX2 9606.ENSP00000383365 9606.ENSP00000356410 0.07 0 0 0 0.083 0.248 0 0.692 0.776

TXNRD2 DDO 9606.ENSP00000383365 9606.ENSP00000357920 0.072 0 0 0 0.062 0.194 0 0.297 0.441

TXNRD2 GCLM 9606.ENSP00000383365 9606.ENSP00000359258 0 0 0 0 0.062 0.057 0 0.457 0.477

TXNRD2 CTH 9606.ENSP00000383365 9606.ENSP00000359976 0 0 0 0 0.062 0 0.9 0.188 0.917

TXNRD2 TXN 9606.ENSP00000383365 9606.ENSP00000363641 0.051 0 0 0 0.078 0.243 0 0.861 0.895

TXNRD2 SRXN1 9606.ENSP00000383365 9606.ENSP00000371388 0 0 0 0 0.122 0 0 0.444 0.491

TXNRD2 SOD3 9606.ENSP00000383365 9606.ENSP00000371554 0 0 0 0 0.055 0.149 0 0.374 0.452

TXNRD2 GPX3 9606.ENSP00000383365 9606.ENSP00000373477 0.05 0 0 0 0.063 0.15 0 0.694 0.738

TXNRD2 SUOX 9606.ENSP00000383365 9606.ENSP00000377668 0.07 0 0 0 0 0 0 0.643 0.654

TXNRD2 VIMP 9606.ENSP00000383365 9606.ENSP00000381282 0 0 0 0 0 0 0 0.766 0.766

TXNRD2 SOD2 9606.ENSP00000383365 9606.ENSP00000446252 0.07 0 0 0 0.108 0 0 0.597 0.636

TXNRD2 GPX1 9606.ENSP00000383365 9606.ENSP00000407375 0.05 0 0 0 0.063 0.15 0 0.743 0.779

TXNRD2 SEPP1 9606.ENSP00000383365 9606.ENSP00000420939 0 0 0 0 0 0 0 0.792 0.792

TXNRD2 TXNRD1 9606.ENSP00000383365 9606.ENSP00000434516 0.07 0 0.444 0.95 0.083 0.267 0.8 0.888 0.867

UCP2 HMOX1 9606.ENSP00000312029 9606.ENSP00000216117 0 0 0 0 0 0 0 0.45 0.45

UCP2 GSR 9606.ENSP00000312029 9606.ENSP00000221130 0 0 0 0 0.064 0.126 0 0.37 0.439

UCP2 CAT 9606.ENSP00000312029 9606.ENSP00000241052 0 0 0 0 0.062 0 0 0.598 0.607

UCP2 NOX4 9606.ENSP00000312029 9606.ENSP00000263317 0 0 0 0 0 0 0 0.407 0.407

UCP2 PPARGC1A 9606.ENSP00000312029 9606.ENSP00000264867 0 0 0 0 0 0 0 0.766 0.766

UCP2 SOD1 9606.ENSP00000312029 9606.ENSP00000270142 0 0 0 0 0 0 0 0.481 0.481

UCP2 ACOX1 9606.ENSP00000312029 9606.ENSP00000293217 0 0 0 0 0 0 0 0.572 0.572

UCP2 NOS3 9606.ENSP00000312029 9606.ENSP00000297494 0 0 0 0 0 0 0 0.468 0.468

UCP2 TXN 9606.ENSP00000312029 9606.ENSP00000363641 0 0 0 0 0 0.062 0 0.391 0.405

UCP2 CYBB 9606.ENSP00000312029 9606.ENSP00000367851 0 0 0 0 0.096 0 0 0.374 0.41

UCP2 GPX4 9606.ENSP00000312029 9606.ENSP00000346103 0 0 0 0 0.064 0 0 0.397 0.411

UCP2 GPX1 9606.ENSP00000312029 9606.ENSP00000407375 0 0 0 0 0.064 0 0 0.516 0.528

UCP2 SOD2 9606.ENSP00000312029 9606.ENSP00000446252 0 0 0 0 0.062 0 0 0.649 0.657

VIMP PON1 9606.ENSP00000381282 9606.ENSP00000222381 0 0 0 0 0 0 0.72 0 0.72

VIMP MSRA 9606.ENSP00000381282 9606.ENSP00000313921 0 0 0 0 0 0 0 0.63 0.63

VIMP GPX4 9606.ENSP00000381282 9606.ENSP00000346103 0 0 0 0 0.062 0 0 0.715 0.722

VIMP APOA4 9606.ENSP00000381282 9606.ENSP00000350425 0 0 0 0 0 0 0.72 0 0.72

VIMP TXN 9606.ENSP00000381282 9606.ENSP00000363641 0 0 0 0 0.092 0 0 0.374 0.408

VIMP GPX3 9606.ENSP00000381282 9606.ENSP00000373477 0 0 0 0 0 0 0 0.768 0.768

VIMP GPX1 9606.ENSP00000381282 9606.ENSP00000407375 0 0 0 0 0 0 0 0.698 0.698

VIMP PCYOX1 9606.ENSP00000381282 9606.ENSP00000387654 0 0 0 0 0 0 0.72 0 0.72

VIMP TXNRD1 9606.ENSP00000381282 9606.ENSP00000434516 0 0 0 0 0.06 0 0 0.716 0.721

VIMP TXNRD2 9606.ENSP00000381282 9606.ENSP00000383365 0 0 0 0 0 0 0 0.766 0.766

VIMP SEPP1 9606.ENSP00000381282 9606.ENSP00000420939 0 0 0 0 0 0 0 0.811 0.811
