# Supplementary material for: Rebalance of the Polyamine Metabolism Suppresses Oxidative Stress and Delays Senescence in Nucleus Pulposus Cells
Source: Oxid Med Cell Longev. 2022 Feb 7;2022:8033353. doi: 10.1155/2022/8033353 (PMC8844099; doi:10.1155/2022/8033353)
Supplement: Supplementary Materials — The raw data of the bioinformatics analysis containing DEGs, GO, KEGG, and PPI are provided in the supplementary file. [file 8033353.f1.zip › 8033353.f1/string_network_coordinates.docx]

**#node identifier x_position y_position color annotation**

ACAN 9606.ENSP00000387356 0.912098501 0.560288675 "rgb(239,233,117)" Aggrecan core protein; This proteoglycan is a major component of extracellular matrix of cartilagenous tissues. A major function of this protein is to resist compression in cartilage. It binds avidly to hyaluronic acid via an N-terminal globular region; C-type lectin domain containing

ACOX1 9606.ENSP00000293217 0.581263383 0.595595855 "rgb(128,239,117)" "Peroxisomal acyl-coenzyme A oxidase 1; Catalyzes the desaturation of acyl-CoAs to 2-trans- enoyl-CoAs. Isoform 1 shows highest activity against medium-chain fatty acyl-CoAs and activity decreases with increasing chain length. Isoform 2 is active against a much broader range of substrates and shows activity towards very long-chain acyl-CoAs. Isoform 2 is twice as active as isoform 1 against 16-hydroxy- palmitoyl-CoA and is 25% more active against 1,16-hexadecanodioyl- CoA"

ACOX3 9606.ENSP00000348775 0.593468951 0.401739452 "rgb(117,239,176)" Peroxisomal acyl-coenzyme A oxidase 3; Oxidizes the CoA-esters of 2-methyl-branched fatty acids; Belongs to the acyl-CoA oxidase family

ADAMTS2 9606.ENSP00000251582 0.800963597 0.626905996 "rgb(117,169,239)" A disintegrin and metalloproteinase with thrombospondin motifs 2; Cleaves the propeptides of type I and II collagen prior to fibril assembly. Does not act on type III collagen. May also play a role in development that is independent of its role in collagen biosynthesis; ADAM metallopeptidases with thrombospondin type 1 motif

ANGPTL7 9606.ENSP00000366015 0.445074946 0.658216136 "rgb(170,239,117)" Angiopoietin-related protein 7; Fibrinogen C domain containing; Angiopoietin like

AOC1 9606.ENSP00000411613 0.499678801 0.627572169 "rgb(117,239,222)" "Amiloride-sensitive amine oxidase [copper-containing]; Catalyzes the degradation of compounds such as putrescine, histamine, spermine, and spermidine, substances involved in allergic and immune responses, cell proliferation, tissue differentiation, tumor formation, and possibly apoptosis. Placental DAO is thought to play a role in the regulation of the female reproductive function; Belongs to the copper/topaquinone oxidase family"

AOC2 9606.ENSP00000253799 0.613383298 0.609585492 "rgb(117,239,201)" Retina-specific copper amine oxidase; Has a monoamine oxidase activity with substrate specificity for 2-phenylethylamine and tryptamine. May play a role in adipogenesis. May be a critical modulator of signal transmission in retina

AOC3 9606.ENSP00000312326 0.641006424 0.576943005 "rgb(117,117,239)" Membrane primary amine oxidase; Cell adhesion protein that participates in lymphocyte extravasation and recirculation by mediating the binding of lymphocytes to peripheral lymph node vascular endothelial cells in an L-selectin-independent fashion. Has semicarbazide-sensitive (SSAO) monoamine oxidase activity. May play a role in adipogenesis

AOX1 9606.ENSP00000363832 0.445074946 0.403071799 "rgb(182,239,117)" "Aldehyde oxidase; Oxidase with broad substrate specificity, oxidizing aromatic azaheterocycles, such as N1-methylnicotinamide, N- methylphthalazinium and phthalazine, as well as aldehydes, such as benzaldehyde, retinal, pyridoxal, and vanillin. Plays a key role in the metabolism of xenobiotics and drugs containing aromatic azaheterocyclic substituents. Participates in the bioactivation of prodrugs such as famciclovir, catalyzing the oxidation step from 6-deoxypenciclovir to penciclovir, which is a potent antiviral agent. Is probably involved in the regulation of reactive oxygen species [...] "

APOA4 9606.ENSP00000350425 0.69111349 0.609585492 "rgb(141,239,117)" Apolipoprotein A-IV; May have a role in chylomicrons and VLDL secretion and catabolism. Required for efficient activation of lipoprotein lipase by ApoC-II; potent activator of LCAT. Apoa-IV is a major component of HDL and chylomicrons; Belongs to the apolipoprotein A1/A4/E family

APTX 9606.ENSP00000400806 0.387259101 0.253849001 "rgb(239,158,117)" "Aprataxin; DNA-binding protein involved in single-strand DNA break repair, double-strand DNA break repair and base excision repair. Resolves abortive DNA ligation intermediates formed either at base excision sites, or when DNA ligases attempt to repair non-ligatable breaks induced by reactive oxygen species. Catalyzes the release of adenylate groups covalently linked to 5'-phosphate termini, resulting in the production of 5'-phosphate termini that can be efficiently rejoined. Also able to hydrolyze adenosine 5'- monophosphoramidate (AMP-NH(2)) and diadenosine tetraphosphate (AppppA), b [...] "

ATOX1 9606.ENSP00000430598 0.434796574 0.590932642 "rgb(239,213,117)" Copper transport protein ATOX1; Binds and deliver cytosolic copper to the copper ATPase proteins. May be important in cellular antioxidant defense; Belongs to the ATX1 family

CAT 9606.ENSP00000241052 0.518308351 0.46569208 "rgb(117,239,189)" "Catalase; Occurs in almost all aerobically respiring organisms and serves to protect cells from the toxic effects of hydrogen peroxide. Promotes growth of cells including T-cells, B-cells, myeloid leukemia cells, melanoma cells, mastocytoma cells and normal and transformed fibroblast cells; Belongs to the catalase family"

CDKN2A 9606.ENSP00000418915 0.808672377 0.421724648 "rgb(239,225,117)" Cyclin-dependent kinase inhibitor 2A; Acts as a negative regulator of the proliferation of normal cells by interacting strongly with CDK4 and CDK6. This inhibits their ability to interact with cyclins D and to phosphorylate the retinoblastoma protein

COL1A1 9606.ENSP00000225964 0.906316916 0.602257587 "rgb(117,239,193)" Collagen alpha-1(I) chain; Type I collagen is a member of group I collagen (fibrillar forming collagen); Collagens

COL2A1 9606.ENSP00000369889 0.887687366 0.525647668 "rgb(255,175,101)" "Collagen alpha-1(II) chain; Type II collagen is specific for cartilaginous tissues. It is essential for the normal embryonic development of the skeleton, for linear growth and for the ability of cartilage to resist compressive forces"

CTH 9606.ENSP00000359976 0.525374732 0.331791266 "rgb(117,117,239)" "Cystathionine gamma-lyase; Catalyzes the last step in the trans-sulfuration pathway from methionine to cysteine. Has broad substrate specificity. Converts cystathionine to cysteine, ammonia and 2-oxobutanoate. Converts two cysteine molecules to lanthionine and hydrogen sulfide. Can also accept homocysteine as substrate. Specificity depends on the levels of the endogenous substrates. Generates the endogenous signaling molecule hydrogen sulfide (H2S), and so contributes to the regulation of blood pressure. Acts as a cysteine-protein sulfhydrase by mediating sulfhydration of target protei [...] "

CYBA 9606.ENSP00000261623 0.637794433 0.458364175 "rgb(117,144,239)" Cytochrome b-245 light chain; Critical component of the membrane-bound oxidase of phagocytes that generates superoxide. Associates with NOX3 to form a functional NADPH oxidase constitutively generating superoxide

CYBB 9606.ENSP00000367851 0.69111349 0.382420429 "rgb(239,150,117)" Cytochrome b-245 heavy chain; Critical component of the membrane-bound oxidase of phagocytes that generates superoxide. It is the terminal component of a respiratory chain that transfers single electrons from cytoplasmic NADPH across the plasma membrane to molecular oxygen on the exterior. Also functions as a voltage-gated proton channel that mediates the H(+) currents of resting phagocytes. It participates in the regulation of cellular pH and is blocked by zinc

CYGB 9606.ENSP00000293230 0.314025696 0.638897113 "rgb(239,229,117)" Cytoglobin; May have a protective function during conditions of oxidative stress. May be involved in intracellular oxygen storage or transfer

CYP1A1 9606.ENSP00000369050 0.405246253 0.389748335 "rgb(174,239,117)" "Cytochrome P450 1A1; Cytochromes P450 are a group of heme-thiolate monooxygenases. In liver microsomes, this enzyme is involved in an NADPH-dependent electron transport pathway. It oxidizes a variety of structurally unrelated compounds, including steroids, fatty acids, and xenobiotics"

DAO 9606.ENSP00000228476 0.529229122 0.609585492 "rgb(120,239,117)" "D-amino-acid oxidase; Regulates the level of the neuromodulator D-serine in the brain. Has high activity towards D-DOPA and contributes to dopamine synthesis. Could act as a detoxifying agent which removes D-amino acids accumulated during aging. Acts on a variety of D- amino acids with a preference for those having small hydrophobic side chains followed by those bearing polar, aromatic, and basic groups. Does not act on acidic amino acids; Belongs to the DAMOX/DASOX family"

DDO 9606.ENSP00000357920 0.575481799 0.321132494 "rgb(117,119,239)" "D-aspartate oxidase; Selectively catalyzes the oxidative deamination of D- aspartate and its N-methylated derivative, N-methyl D-aspartate"

DGKK 9606.ENSP00000477515 0.300535332 0.302479645 "rgb(117,194,239)" Diacylglycerol kinase kappa; Phosphorylates diacylglycerol (DAG) to generate phosphatidic acid (PA)

DHCR24 9606.ENSP00000360316 0.40267666 0.652886751 "rgb(191,239,117)" Delta(24)-sterol reductase; Catalyzes the reduction of the delta-24 double bond of sterol intermediates. Protects cells from oxidative stress by reducing caspase 3 activity during apoptosis induced by oxidative stress. Also protects against amyloid-beta peptide-induced apoptosis; Belongs to the FAD-binding oxidoreductase/transferase type 4 family

DRD1 9606.ENSP00000377353 0.56391863 0.622908956 "rgb(117,117,239)" D(1A) dopamine receptor; Dopamine receptor whose activity is mediated by G proteins which activate adenylyl cyclase; Belongs to the G-protein coupled receptor 1 family

DRD2 9606.ENSP00000354859 0.569057816 0.652220577 "rgb(199,239,117)" D(2) dopamine receptor; Dopamine receptor whose activity is mediated by G proteins which inhibit adenylyl cyclase; Belongs to the G-protein coupled receptor 1 family

DRD3 9606.ENSP00000373169 0.473982869 0.510991858 "rgb(239,183,117)" D(3) dopamine receptor; Dopamine receptor whose activity is mediated by G proteins which inhibit adenylyl cyclase. Promotes cell proliferation; Belongs to the G-protein coupled receptor 1 family

DRD4 9606.ENSP00000176183 0.496466809 0.554293116 "rgb(117,239,230)" Dopamine receptor D4; Belongs to the G-protein coupled receptor 1 family

DRD5 9606.ENSP00000306129 0.527944325 0.668208734 "rgb(117,239,197)" D(1B) dopamine receptor; Dopamine receptor whose activity is mediated by G proteins which activate adenylyl cyclase; Belongs to the G-protein coupled receptor 1 family

DUOX1 9606.ENSP00000317997 0.457922912 0.293819393 "rgb(212,239,117)" Dual oxidase 1; Generates hydrogen peroxide which is required for the activity of thyroid peroxidase/TPO and lactoperoxidase/LPO. Plays a role in thyroid hormones synthesis and lactoperoxidase-mediated antimicrobial defense at the surface of mucosa. May have its own peroxidase activity through its N-terminal peroxidase-like domain; EF-hand domain containing

DUOX2 9606.ENSP00000475084 0.628800857 0.301813472 "rgb(178,171,0)" Dual oxidase 2; Generates hydrogen peroxide which is required for the activity of thyroid peroxidase/TPO and lactoperoxidase/LPO. Plays a role in thyroid hormones synthesis and lactoperoxidase-mediated antimicrobial defense at the surface of mucosa. May have its own peroxidase activity through its N-terminal peroxidase-like domain

DUSP1 9606.ENSP00000239223 0.565845824 0.370429312 "rgb(117,239,185)" "Dual specificity protein phosphatase 1; Dual specificity phosphatase that dephosphorylates MAP kinase MAPK1/ERK2 on both 'Thr-183' and 'Tyr-185', regulating its activity during the meiotic cell cycle; Belongs to the protein-tyrosine phosphatase family. Non-receptor class dual specificity subfamily"

ERCC1 9606.ENSP00000013807 0.565845824 0.256513694 "rgb(162,239,117)" "DNA excision repair protein ERCC-1; Isoform 1: Non-catalytic component of a structure- specific DNA repair endonuclease responsible for the 5'-incision during DNA repair. Responsible, in conjunction with SLX4, for the first step in the repair of interstrand cross-links (ICL). Participates in the processing of anaphase bridge-generating DNA structures, which consist in incompletely processed DNA lesions arising during S or G2 phase, and can result in cytokinesis failure. Also required for homology-directed repair (HDR) of DNA double-strand breaks, in conjunction with SLX4; ERCC excision [...] "

ERCC2 9606.ENSP00000375809 0.420021413 0.292487047 "rgb(117,161,239)" "TFIIH basal transcription factor complex helicase XPD subunit; ATP-dependent 5'-3' DNA helicase, component of the core- TFIIH basal transcription factor. Involved in nucleotide excision repair (NER) of DNA by opening DNA around the damage, and in RNA transcription by RNA polymerase II by anchoring the CDK-activating kinase (CAK) complex, composed of CDK7, cyclin H and MAT1, to the core-TFIIH complex. Involved in the regulation of vitamin-D receptor activity. As part of the mitotic spindle-associated MMXD complex it plays a role in chromosome segregation. Might have a role in aging proc [...] "

ERCC3 9606.ENSP00000285398 0.472698073 0.245854922 "rgb(117,219,239)" "TFIIH basal transcription factor complex helicase XPB subunit; ATP-dependent 3'-5' DNA helicase, component of the core- TFIIH basal transcription factor, involved in nucleotide excision repair (NER) of DNA and, when complexed to CAK, in RNA transcription by RNA polymerase II. Acts by opening DNA either around the RNA transcription start site or the DNA damage"

ERCC6 9606.ENSP00000348089 0.419379015 0.249851962 "rgb(124,239,117)" "DNA excision repair protein ERCC-6; Essential factor involved in transcription-coupled nucleotide excision repair which allows RNA polymerase II-blocking lesions to be rapidly removed from the transcribed strand of active genes. Upon DNA-binding, it locally modifies DNA conformation by wrapping the DNA around itself, thereby modifying the interface between stalled RNA polymerase II and DNA. It is required for transcription-coupled repair complex formation. It recruits the CSA complex (DCX(ERCC8) complex), nucleotide excision repair proteins and EP300 to the at sites of RNA polymerase I [...] "

ERCC8 9606.ENSP00000265038 0.515738758 0.245854922 "rgb(117,117,239)" "DNA excision repair protein ERCC-8; Substrate-recognition component of the CSA complex, a DCX (DDB1-CUL4-X-box) E3 ubiquitin-protein ligase complex, involved in transcription-coupled nucleotide excision repair. The CSA complex (DCX(ERCC8) complex) promotes the ubiquitination and subsequent proteasomal degradation of ERCC6 in a UV-dependent manner; ERCC6 degradation is essential for the recovery of RNA synthesis after transcription-coupled repair. It is required for the recruitment of XAB2, HMGN1 and TCEA1/TFIIS to a transcription- coupled repair complex which removes RNA polymerase II- [...] "

FGF5 9606.ENSP00000311697 0.383404711 0.680866025 "rgb(117,136,239)" "Fibroblast growth factor 5; Plays an important role in the regulation of cell proliferation and cell differentiation. Required for normal regulation of the hair growth cycle. Functions as an inhibitor of hair elongation by promoting progression from anagen, the growth phase of the hair follicle, into catagen the apoptosis-induced regression phase (By similarity); Endogenous ligands"

FOS 9606.ENSP00000306245 0.60117773 0.538304959 "rgb(239,142,117)" "Proto-oncogene c-Fos; Nuclear phosphoprotein which forms a tight but non- covalently linked complex with the JUN/AP-1 transcription factor. In the heterodimer, FOS and JUN/AP-1 basic regions each seems to interact with symmetrical DNA half sites. On TGF-beta activation, forms a multimeric SMAD3/SMAD4/JUN/FOS complex at the AP1/SMAD- binding site to regulate TGF-beta-mediated signaling. Has a critical function in regulating the development of cells destined to form and maintain the skeleton. It is thought to have an important role in signal transduction, cell proliferation and different [...] "

GCLC 9606.ENSP00000229416 0.406531049 0.349777942 "rgb(239,204,117)" Glutamate-cysteine ligase catalytic subunit

GCLM 9606.ENSP00000359258 0.333940043 0.399074759 "rgb(255,0,0)" Glutamate-cysteine ligase modifier subunit; Belongs to the aldo/keto reductase family. Glutamate-- cysteine ligase light chain subfamily

GFER 9606.ENSP00000248114 0.405888651 0.610917839 "rgb(207,239,117)" "FAD-linked sulfhydryl oxidase ALR; Isoform 1: FAD-dependent sulfhydryl oxidase that regenerates the redox-active disulfide bonds in CHCHD4/MIA40, a chaperone essential for disulfide bond formation and protein folding in the mitochondrial intermembrane space. The reduced form of CHCHD4/MIA40 forms a transient intermolecular disulfide bridge with GFER/ERV1, resulting in regeneration of the essential disulfide bonds in CHCHD4/MIA40, while GFER/ERV1 becomes re- oxidized by donating electrons to cytochrome c or molecular oxygen"

GLRX2 9606.ENSP00000356410 0.35 0.481014064 "rgb(239,217,117)" "Glutaredoxin-2, mitochondrial; Glutathione-dependent oxidoreductase that facilitates the maintenance of mitochondrial redox homeostasis upon induction of apoptosis by oxidative stress. Involved in response to hydrogen peroxide and regulation of apoptosis caused by oxidative stress. Acts as a very efficient catalyst of monothiol reactions because of its high affinity for protein glutathione-mixed disulfides. Can receive electrons not only from glutathione (GSH), but also from thioredoxin reductase supporting both monothiol and dithiol reactions. Efficiently catalyzes both glutathionylat [...] "

GPX1 9606.ENSP00000407375 0.387901499 0.421058475 "rgb(150,101,255)" Glutathione peroxidase 1; Protects the hemoglobin in erythrocytes from oxidative breakdown; Belongs to the glutathione peroxidase family

GPX3 9606.ENSP00000373477 0.300535332 0.491006662 "rgb(239,154,117)" "Glutathione peroxidase 3; Protects cells and enzymes from oxidative damage, by catalyzing the reduction of hydrogen peroxide, lipid peroxides and organic hydroperoxide, by glutathione; Selenoproteins"

GPX4 9606.ENSP00000346103 0.373768737 0.451036269 "rgb(0,178,157)" "Phospholipid hydroperoxide glutathione peroxidase, mitochondrial; Protects cells against membrane lipid peroxidation and cell death. Required for normal sperm development and male fertility. Could play a major role in protecting mammals from the toxicity of ingested lipid hydroperoxides. Essential for embryonic development. Protects from radiation and oxidative damage. Essential for maturation and survival of photoreceptor cells. Plays a role in a primary T cell response to viral and parasitic infection by protecting T cells from ferroptosis, a cell death resulting from an iron-depende [...] "

GSR 9606.ENSP00000221130 0.295396146 0.407735011 "rgb(239,200,117)" "Glutathione reductase, mitochondrial; Maintains high levels of reduced glutathione in the cytosol"

GSS 9606.ENSP00000216951 0.370556745 0.354441155 "rgb(117,239,214)" Glutathione synthetase; Belongs to the eukaryotic GSH synthase family

GSTA1 9606.ENSP00000335620 0.306316916 0.331791266 "rgb(117,239,151)" Glutathione S-transferase A1; Conjugation of reduced glutathione to a wide number of exogenous and endogenous hydrophobic electrophiles; Belongs to the GST superfamily. Alpha family

GSTM1 9606.ENSP00000311469 0.430299786 0.327794226 "rgb(117,239,235)" Glutathione S-transferase Mu 1; Conjugation of reduced glutathione to a wide number of exogenous and endogenous hydrophobic electrophiles; Soluble glutathione S-transferases

GSTM3 9606.ENSP00000256594 0.355781585 0.301813472 "rgb(117,239,218)" Glutathione S-transferase Mu 3; Conjugation of reduced glutathione to a wide number of exogenous and endogenous hydrophobic electrophiles. May govern uptake and detoxification of both endogenous compounds and xenobiotics at the testis and brain blood barriers; Belongs to the GST superfamily. Mu family

GSTP1 9606.ENSP00000381607 0.486188437 0.320466321 "rgb(187,239,117)" Glutathione S-transferase P; Conjugation of reduced glutathione to a wide number of exogenous and endogenous hydrophobic electrophiles. Regulates negatively CDK5 activity via p25/p35 translocation to prevent neurodegeneration; Soluble glutathione S-transferases

GSTT2B 9606.ENSP00000290765 0.348715203 0.421058475 "rgb(239,146,117)" Glutathione S-transferase theta-2B; Conjugation of reduced glutathione to a wide number of exogenous and endogenous hydrophobic electrophiles. Has a sulfatase activity; Soluble glutathione S-transferases

HAO1 9606.ENSP00000368066 0.565203426 0.563619541 "rgb(237,239,117)" "Hydroxyacid oxidase 1; Has 2-hydroxyacid oxidase activity. Most active on the 2-carbon substrate glycolate, but is also active on 2-hydroxy fatty acids, with high activity towards 2-hydroxy palmitate and 2- hydroxy octanoate"

HAO2 9606.ENSP00000483507 0.520235546 0.509659511 "rgb(117,117,239)" "Hydroxyacid oxidase 2; Catalyzes the oxidation of L-alpha-hydroxy acids as well as, more slowly, that of L-alpha-amino acids; Belongs to the FMN-dependent alpha-hydroxy acid dehydrogenase family"

HMOX1 9606.ENSP00000216117 0.686616702 0.5696151 "rgb(117,206,239)" "Heme oxygenase 1; Heme oxygenase cleaves the heme ring at the alpha methene bridge to form biliverdin. Biliverdin is subsequently converted to bilirubin by biliverdin reductase. Under physiological conditions, the activity of heme oxygenase is highest in the spleen, where senescent erythrocytes are sequestrated and destroyed. Exhibits cytoprotective effects since excess of free heme sensitizes cells to undergo apoptosis"

HMOX2 9606.ENSP00000477572 0.547858672 0.453700962 "rgb(117,215,239)" "Heme oxygenase 2; Heme oxygenase cleaves the heme ring at the alpha methene bridge to form biliverdin. Biliverdin is subsequently converted to bilirubin by biliverdin reductase. Under physiological conditions, the activity of heme oxygenase is highest in the spleen, where senescent erythrocytes are sequestrated and destroyed. Heme oxygenase 2 could be implicated in the production of carbon monoxide in brain where it could act as a neurotransmitter"

IL4I1 9606.ENSP00000472474 0.471413276 0.58826795 "rgb(117,227,239)" L-amino-acid oxidase; Lysosomal L-amino-acid oxidase with highest specific activity with phenylalanine. May play a role in lysosomal antigen processing and presentation (By similarity); Belongs to the flavin monoamine oxidase family. FIG1 subfamily

IPCEF1 9606.ENSP00000394751 0.60117773 0.275832717 "rgb(101,255,163)" Interactor protein for cytohesin exchange factors 1; Enhances the promotion of guanine-nucleotide exchange by PSCD2 on ARF6 in a concentration-dependent manner; Pleckstrin homology domain containing

JUNB 9606.ENSP00000303315 0.644860814 0.392413027 "rgb(239,125,117)" Transcription factor jun-B; Transcription factor involved in regulating gene activity following the primary growth factor response. Binds to the DNA sequence 5'-TGA[CG]TCA-3'; Belongs to the bZIP family. Jun subfamily

LOX 9606.ENSP00000231004 0.662847966 0.596262028 "rgb(117,140,239)" Protein-lysine 6-oxidase; Responsible for the post-translational oxidative deamination of peptidyl lysine residues in precursors to fibrous collagen and elastin. Regulator of Ras expression. May play a role in tumor suppression. Plays a role in the aortic wall architecture (By similarity); Belongs to the lysyl oxidase family

MAOA 9606.ENSP00000340684 0.512526767 0.58826795 "rgb(117,239,239)" "Amine oxidase [flavin-containing] A; Catalyzes the oxidative deamination of biogenic and xenobiotic amines and has important functions in the metabolism of neuroactive and vasoactive amines in the central nervous system and peripheral tissues. MAOA preferentially oxidizes biogenic amines such as 5-hydroxytryptamine (5-HT), norepinephrine and epinephrine; Belongs to the flavin monoamine oxidase family"

MAOB 9606.ENSP00000367309 0.463062099 0.618245744 "rgb(195,239,117)" Amine oxidase [flavin-containing] B; Catalyzes the oxidative deamination of biogenic and xenobiotic amines and has important functions in the metabolism of neuroactive and vasoactive amines in the central nervous system and peripheral tissues. MAOB preferentially degrades benzylamine and phenylethylamine

MAPK10 9606.ENSP00000352157 0.617880086 0.34511473 "rgb(239,196,117)" "Mitogen-activated protein kinase 10; Serine/threonine-protein kinase involved in various processes such as neuronal proliferation, differentiation, migration and programmed cell death. Extracellular stimuli such as proinflammatory cytokines or physical stress stimulate the stress- activated protein kinase/c-Jun N-terminal kinase (SAP/JNK) signaling pathway. In this cascade, two dual specificity kinases MAP2K4/MKK4 and MAP2K7/MKK7 phosphorylate and activate MAPK10/JNK3. In turn, MAPK10/JNK3 phosphorylates a number of transcription factors, primarily components of AP-1 such as JUN and AT [...] "

MAPK14 9606.ENSP00000229795 0.599250535 0.44903775 "rgb(117,152,239)" "Mitogen-activated protein kinase 14; Serine/threonine kinase which acts as an essential component of the MAP kinase signal transduction pathway. MAPK14 is one of the four p38 MAPKs which play an important role in the cascades of cellular responses evoked by extracellular stimuli such as proinflammatory cytokines or physical stress leading to direct activation of transcription factors. Accordingly, p38 MAPKs phosphorylate a broad range of proteins and it has been estimated that they may have approximately 200 to 300 substrates each. Some of the targets are downstream kinases which are a [...] "

MGST1 9606.ENSP00000379512 0.367987152 0.385751295 "rgb(117,239,210)" Microsomal glutathione S-transferase 1; Conjugation of reduced glutathione to a wide number of exogenous and endogenous hydrophobic electrophiles. Has a wide substrate specificity; Microsomal glutathione S-transferases

MMP1 9606.ENSP00000322788 0.808672377 0.521650629 "rgb(157,239,117)" "Interstitial collagenase; Cleaves collagens of types I, II, and III at one site in the helical domain. Also cleaves collagens of types VII and X. In case of HIV infection, interacts and cleaves the secreted viral Tat protein, leading to a decrease in neuronal Tat's mediated neurotoxicity; Endogenous ligands"

MMP10 9606.ENSP00000279441 0.851713062 0.512324204 "rgb(137,239,117)" "Stromelysin-2; Can degrade fibronectin, gelatins of type I, III, IV, and V; weakly collagens III, IV, and V. Activates procollagenase; Belongs to the peptidase M10A family"

MMP13 9606.ENSP00000260302 0.827944325 0.563619541 "rgb(117,239,180)" "Collagenase 3; Plays a role in the degradation of extracellular matrix proteins including fibrillar collagen, fibronectin, TNC and ACAN. Cleaves triple helical collagens, including type I, type II and type III collagen, but has the highest activity with soluble type II collagen. Can also degrade collagen type IV, type XIV and type X. May also function by activating or degrading key regulatory proteins, such as TGFB1 and CTGF. Plays a role in wound healing, tissue remodeling, cartilage degradation, bone development, bone mineralization and ossification. Required for normal embryonic bon [...] "

MMP14 9606.ENSP00000308208 0.8748394 0.620910437 "rgb(117,148,239)" Matrix metalloproteinase-14; Endopeptidase that degrades various components of the extracellular matrix such as collagen. Activates progelatinase A. Essential for pericellular collagenolysis and modeling of skeletal and extraskeletal connective tissues during development (By similarity). May be involved in actin cytoskeleton reorganization by cleaving PTK7. Acts as a positive regulator of cell growth and migration via activation of MMP15. Involved in the formation of the fibrovascular tissues in association with pro-MMP2. Cleaves ADGRB1 to release vasculostatin-40 which inhibits angiog [...]

MMP19 9606.ENSP00000313437 0.883190578 0.646225019 "rgb(117,117,239)" "Matrix metalloproteinase-19; Endopeptidase that degrades various components of the extracellular matrix, such as aggrecan and cartilage oligomeric matrix protein (comp), during development, haemostasis and pathological conditions (arthritic disease). May also play a role in neovascularization or angiogenesis. Hydrolyzes collagen type IV, laminin, nidogen, nascin-C isoform, fibronectin, and type I gelatin; Belongs to the peptidase M10A family"

MMP3 9606.ENSP00000299855 0.840149893 0.648223538 "rgb(117,117,239)" "Stromelysin-1; Can degrade fibronectin, laminin, gelatins of type I, III, IV, and V; collagens III, IV, X, and IX, and cartilage proteoglycans. Activates procollagenase; Belongs to the peptidase M10A family"

MMP8 9606.ENSP00000236826 0.861349036 0.548963731 "rgb(117,190,239)" "Neutrophil collagenase; Can degrade fibrillar type I, II, and III collagens; Belongs to the peptidase M10A family"

MMP9 9606.ENSP00000361405 0.781691649 0.597594375 "rgb(239,188,117)" Matrix metalloproteinase-9; May play an essential role in local proteolysis of the extracellular matrix and in leukocyte migration. Could play a role in bone osteoclastic resorption. Cleaves KiSS1 at a Gly-|-Leu bond. Cleaves type IV and type V collagen into large C-terminal three quarter fragments and shorter N-terminal one quarter fragments. Degrades fibronectin but not laminin or Pz-peptide; M10 matrix metallopeptidases

MPO 9606.ENSP00000225275 0.603104925 0.492339008 "rgb(239,167,117)" "Myeloperoxidase; Part of the host defense system of polymorphonuclear leukocytes. It is responsible for microbicidal activity against a wide range of organisms. In the stimulated PMN, MPO catalyzes the production of hypohalous acids, primarily hypochlorous acid in physiologic situations, and other toxic intermediates that greatly enhance PMN microbicidal activity; Belongs to the peroxidase family. XPO subfamily"

MSRA 9606.ENSP00000313921 0.414882227 0.445040711 "rgb(239,133,117)" Mitochondrial peptide methionine sulfoxide reductase; Has an important function as a repair enzyme for proteins that have been inactivated by oxidation. Catalyzes the reversible oxidation-reduction of methionine sulfoxide in proteins to methionine

MT1X 9606.ENSP00000377995 0.246573876 0.413064397 "rgb(239,221,117)" Metallothionein-1X; Metallothioneins have a high content of cysteine residues that bind various heavy metals; these proteins are transcriptionally regulated by both heavy metals and glucocorticoids. May be involved in FAM168A anti-apoptotic signaling

MTHFR 9606.ENSP00000365777 0.455353319 0.363101406 "rgb(117,117,239)" "Methylenetetrahydrofolate reductase; Catalyzes the conversion of 5,10- methylenetetrahydrofolate to 5-methyltetrahydrofolate, a co- substrate for homocysteine remethylation to methionine"

NCF2 9606.ENSP00000356505 0.707173448 0.42838638 "rgb(239,238,117)" "Neutrophil cytosol factor 2; NCF2, NCF1, and a membrane bound cytochrome b558 are required for activation of the latent NADPH oxidase (necessary for superoxide production); Tetratricopeptide repeat domain containing"

NDUFA12 9606.ENSP00000330737 0.303747323 0.459030348 "rgb(0,28,178)" "NADH dehydrogenase [ubiquinone] 1 alpha subcomplex subunit 12; Accessory subunit of the mitochondrial membrane respiratory chain NADH dehydrogenase (Complex I), that is believed not to be involved in catalysis. Complex I functions in the transfer of electrons from NADH to the respiratory chain. The immediate electron acceptor for the enzyme is believed to be ubiquinone"

NDUFA6 9606.ENSP00000418842 0.24143469 0.456365655 "rgb(117,239,122)" "NADH dehydrogenase [ubiquinone] 1 alpha subcomplex subunit 6; Accessory subunit of the mitochondrial membrane respiratory chain NADH dehydrogenase (Complex I), that is believed to be not involved in catalysis. Complex I functions in the transfer of electrons from NADH to the respiratory chain. The immediate electron acceptor for the enzyme is believed to be ubiquinone; LYR motif containing"

NDUFB4 9606.ENSP00000184266 0.287687366 0.439045152 "rgb(117,239,160)" "NADH dehydrogenase [ubiquinone] 1 beta subcomplex subunit 4; Accessory subunit of the mitochondrial membrane respiratory chain NADH dehydrogenase (Complex I), that is believed not to be involved in catalysis. Complex I functions in the transfer of electrons from NADH to the respiratory chain. The immediate electron acceptor for the enzyme is believed to be ubiquinone"

NDUFS2 9606.ENSP00000356972 0.242077088 0.505662472 "rgb(239,175,117)" "NADH dehydrogenase [ubiquinone] iron-sulfur protein 2, mitochondrial; Core subunit of the mitochondrial membrane respiratory chain NADH dehydrogenase (Complex I) that is believed to belong to the minimal assembly required for catalysis. Complex I functions in the transfer of electrons from NADH to the respiratory chain. The immediate electron acceptor for the enzyme is believed to be ubiquinone"

NDUFS8 9606.ENSP00000315774 0.37633833 0.554293116 "rgb(117,239,226)" "NADH dehydrogenase [ubiquinone] iron-sulfur protein 8, mitochondrial; Core subunit of the mitochondrial membrane respiratory chain NADH dehydrogenase (Complex I) that is believed to belong to the minimal assembly required for catalysis. Complex I functions in the transfer of electrons from NADH to the respiratory chain. The immediate electron acceptor for the enzyme is believed to be ubiquinone (By similarity). May donate electrons to ubiquinone; NADH:ubiquinone oxidoreductase core subunits"

NFE2L2 9606.ENSP00000380252 0.494539615 0.369096965 "rgb(239,129,117)" Nuclear factor erythroid 2-related factor 2; Transcription activator that binds to antioxidant response (ARE) elements in the promoter regions of target genes. Important for the coordinated up-regulation of genes in response to oxidative stress. May be involved in the transcriptional activation of genes of the beta-globin cluster by mediating enhancer activity of hypersensitive site 2 of the beta-globin locus control region; Basic leucine zipper proteins

NFIX 9606.ENSP00000380781 0.267773098 0.576943005 "rgb(178,239,117)" Nuclear factor 1 X-type; Recognizes and binds the palindromic sequence 5'- TTGGCNNNNNGCCAA-3' present in viral and cellular promoters and in the origin of replication of adenovirus type 2. These proteins are individually capable of activating transcription and replication

NFKB1 9606.ENSP00000226574 0.664132762 0.540969652 "rgb(117,239,139)" "Nuclear factor NF-kappa-B p105 subunit; NF-kappa-B is a pleiotropic transcription factor present in almost all cell types and is the endpoint of a series of signal transduction events that are initiated by a vast array of stimuli related to many biological processes such as inflammation, immunity, differentiation, cell growth, tumorigenesis and apoptosis. NF-kappa-B is a homo- or heterodimeric complex formed by the Rel-like domain-containing proteins RELA/p65, RELB, NFKB1/p105, NFKB1/p50, REL and NFKB2/p52 and the heterodimeric p65-p50 complex appears to be most abundant one. The dimer [...] "

NOS1 9606.ENSP00000477999 0.534368308 0.412398224 "rgb(117,198,239)" "Nitric oxide synthase, brain; Produces nitric oxide (NO) which is a messenger molecule with diverse functions throughout the body. In the brain and peripheral nervous system, NO displays many properties of a neurotransmitter. Probably has nitrosylase activity and mediates cysteine S-nitrosylation of cytoplasmic target proteins such SRR; Belongs to the NOS family"

NOS2 9606.ENSP00000327251 0.700749465 0.543634345 "rgb(203,239,117)" "Nitric oxide synthase, inducible; Produces nitric oxide (NO) which is a messenger molecule with diverse functions throughout the body. In macrophages, NO mediates tumoricidal and bactericidal actions. Also has nitrosylase activity and mediates cysteine S-nitrosylation of cytoplasmic target proteins such PTGS2/COX2 (By similarity). As component of the iNOS-S100A8/9 transnitrosylase complex involved in the selective inflammatory stimulus-dependent S-nitrosylation of GAPDH on 'Cys-247' implicated in regulation of the GAIT complex activity and probably multiple targets including ANXA5, EZR [...] "

NOS3 9606.ENSP00000297494 0.628800857 0.550296077 "rgb(117,131,239)" "Nitric oxide synthase, endothelial; Produces nitric oxide (NO) which is implicated in vascular smooth muscle relaxation through a cGMP-mediated signal transduction pathway. NO mediates vascular endothelial growth factor (VEGF)-induced angiogenesis in coronary vessels and promotes blood clotting through the activation of platelets"

NOX1 9606.ENSP00000362057 0.712312634 0.4676906 "rgb(239,179,117)" "NADPH oxidase 1; NOH-1S is a voltage-gated proton channel that mediates the H(+) currents of resting phagocytes and other tissues. It participates in the regulation of cellular pH and is blocked by zinc. NOH-1L is a pyridine nucleotide-dependent oxidoreductase that generates superoxide and might conduct H(+) ions as part of its electron transport mechanism, whereas NOH-1S does not contain an electron transport chain"

NOX3 9606.ENSP00000159060 0.661563169 0.339785344 "rgb(117,236,239)" "NADPH oxidase 3; NADPH oxidase which constitutively produces superoxide upon formation of a complex with CYBA/p22phox. Plays a role in the biogenesis of otoconia/otolith, which are crystalline structures of the inner ear involved in the perception of gravity"

NOX4 9606.ENSP00000263317 0.554925054 0.286491488 "rgb(117,223,239)" "NADPH oxidase 4; Constitutive NADPH oxidase which generates superoxide intracellularly upon formation of a complex with CYBA/p22phox. Regulates signaling cascades probably through phosphatases inhibition. May function as an oxygen sensor regulating the KCNK3/TASK-1 potassium channel and HIF1A activity. May regulate insulin signaling cascade. May play a role in apoptosis, bone resorption and lipolysaccharide-mediated activation of NFKB. May produce superoxide in the nucleus and play a role in regulating gene expression upon cell stimulation. Isoform 3 is not functional. Isoform 5 and is [...] "

NOX5 9606.ENSP00000373518 0.511884368 0.283826795 "rgb(117,123,239)" "NADPH oxidase 5; Calcium-dependent NADPH oxidase that generates superoxide. Also functions as a calcium-dependent proton channel and may regulate redox-dependent processes in lymphocytes and spermatozoa. May play a role in cell growth and apoptosis. Isoform v2 and isoform v5 are involved in endothelial generation of reactive oxygen species (ROS), proliferation and angiogenesis and contribute to endothelial response to thrombin; EF-hand domain containing"

NQO1 9606.ENSP00000319788 0.422591006 0.525647668 "rgb(117,239,155)" NAD(P)H dehydrogenase [quinone] 1; The enzyme apparently serves as a quinone reductase in connection with conjugation reactions of hydroquinons involved in detoxification pathways as well as in biosynthetic processes such as the vitamin K-dependent gamma-carboxylation of glutamate residues in prothrombin synthesis; Belongs to the NAD(P)H dehydrogenase (quinone) family

NUDT1 9606.ENSP00000380241 0.292826552 0.562953368 "rgb(166,239,117)" "7,8-dihydro-8-oxoguanine triphosphatase; Antimutagenic. Acts as a sanitizing enzyme for oxidized nucleotide pools, thus suppressing cell dysfunction and death induced by oxidative stress. Hydrolyzes 8-oxo-dGTP, 8-oxo-dATP and 2-OH-dATP, thus preventing misincorporation of oxidized purine nucleoside triphosphates into DNA and subsequently preventing A:T to C:G and G:C to T:A transversions. Able to hydrolyze also the corresponding ribonucleotides, 2-OH-ATP, 8-oxo-GTP and 8-oxo-ATP. Does not play a role in U8 snoRNA decapping activity. Binds U8 snoRNA; Nudix hydrolase family"

OXSR1 9606.ENSP00000311713 0.283190578 0.612250185 "rgb(117,239,147)" Serine/threonine-protein kinase OSR1; Regulates downstream kinases in response to environmental stress. May also have a function in regulating the actin cytoskeleton; Belongs to the protein kinase superfamily. STE Ser/Thr protein kinase family. STE20 subfamily

PAOX 9606.ENSP00000278060 0.845289079 0.369763138 "rgb(224,239,117)" "Peroxisomal N(1)-acetyl-spermine/spermidine oxidase; Flavoenzyme which catalyzes the oxidation of N(1)- acetylspermine to spermidine and is thus involved in the polyamine back-conversion. Can also oxidize N(1)-acetylspermidine to putrescine. Substrate specificity: N(1)-acetylspermine = N(1)- acetylspermidine > N(1),N(12)-diacylspermine >> spermine. Does not oxidize spermidine. Plays an important role in the regulation of polyamine intracellular concentration and has the potential to act as a determinant of cellular sensitivity to the antitumor polyamine analogs; Belongs to the flavin m [...] "

PARK7 9606.ENSP00000418770 0.385974304 0.594263509 "rgb(239,163,117)" "Protein/nucleic acid deglycase DJ-1; Protein and nucleotide deglycase that catalyzes the deglycation of the Maillard adducts formed between amino groups of proteins or nucleotides and reactive carbonyl groups of glyoxals. Thus, functions as a protein deglycase that repairs methylglyoxal- and glyoxal-glycated proteins, and releases repaired proteins and lactate or glycolate, respectively. Deglycates cysteine, arginine and lysine residues in proteins, and thus reactivates these proteins by reversing glycation by glyoxals. Acts on early glycation intermediates (hemithioacetals and aminoca [...] "

PCYOX1 9606.ENSP00000387654 0.434796574 0.630903035 "rgb(216,239,117)" "Prenylcysteine oxidase 1; Involved in the degradation of prenylated proteins. Cleaves the thioether bond of prenyl-L-cysteines, such as farnesylcysteine and geranylgeranylcysteine"

PDLIM1 9606.ENSP00000360305 0.34550329 0.664877868 "rgb(117,239,130)" "PDZ and LIM domain protein 1; Cytoskeletal protein that may act as an adapter that brings other proteins (like kinases) to the cytoskeleton. Involved in assembly, disassembly and directioning of stress fibers in fibroblasts. Required for the localization of ACTN1 and PALLD to stress fibers. Required for cell migration and in maintaining cell polarity of fibroblasts (By similarity); LIM domain containing"

PIPOX 9606.ENSP00000317721 0.562633833 0.508327165 "rgb(239,208,117)" "Peroxisomal sarcosine oxidase; Metabolizes sarcosine, L-pipecolic acid and L-proline"

PNKP 9606.ENSP00000323511 0.340364026 0.277831236 "rgb(117,117,239)" "Bifunctional polynucleotide phosphatase/kinase; Plays a key role in the repair of DNA damage, functioning as part of both the non-homologous end-joining (NHEJ) and base excision repair (BER) pathways. Through its two catalytic activities, PNK ensures that DNA termini are compatible with extension and ligation by either removing 3'-phosphates from, or by phosphorylating 5'-hydroxyl groups on, the ribose sugar of the DNA backbone; HAD Asp-based non-protein phosphatases"

PNPO 9606.ENSP00000225573 0.388543897 0.293819393 "rgb(101,199,255)" Pyridoxine-5'-phosphate oxidase; Catalyzes the oxidation of either pyridoxine 5'- phosphate (PNP) or pyridoxamine 5'-phosphate (PMP) into pyridoxal 5'-phosphate (PLP)

PON1 9606.ENSP00000222381 0.438650964 0.545632865 "rgb(117,239,205)" "Serum paraoxonase/arylesterase 1; Hydrolyzes the toxic metabolites of a variety of organophosphorus insecticides. Capable of hydrolyzing a broad spectrum of organophosphate substrates and lactones, and a number of aromatic carboxylic acid esters. Mediates an enzymatic protection of low density lipoproteins against oxidative modification and the consequent series of events leading to atheroma formation; Belongs to the paraoxonase family"

PON2 9606.ENSP00000222572 0.468843683 0.555625463 "rgb(117,202,239)" "Serum paraoxonase/arylesterase 2; Capable of hydrolyzing lactones and a number of aromatic carboxylic acid esters. Has antioxidant activity. Is not associated with high density lipoprotein. Prevents LDL lipid peroxidation, reverses the oxidation of mildly oxidized LDL, and inhibits the ability of MM-LDL to induce monocyte chemotaxis; Paraoxonases"

PPARGC1A 9606.ENSP00000264867 0.525374732 0.558956329 "rgb(153,239,117)" Peroxisome proliferator-activated receptor gamma coactivator 1-alpha; Transcriptional coactivator for steroid receptors and nuclear receptors. Greatly increases the transcriptional activity of PPARG and thyroid hormone receptor on the uncoupling protein promoter. Can regulate key mitochondrial genes that contribute to the program of adaptive thermogenesis. Plays an essential role in metabolic reprogramming in response to dietary availability through coordination of the expression of a wide array of genes involved in glucose and fatty acid metabolism. Induces the expression of PERM1 in [...]

PRDX2 9606.ENSP00000301522 0.32366167 0.542968172 "rgb(117,211,239)" "Peroxiredoxin-2; Thiol-specific peroxidase that catalyzes the reduction of hydrogen peroxide and organic hydroperoxides to water and alcohols, respectively. Plays a role in cell protection against oxidative stress by detoxifying peroxides and as sensor of hydrogen peroxide-mediated signaling events. Might participate in the signaling cascades of growth factors and tumor necrosis factor-alpha by regulating the intracellular concentrations of H(2)O(2); Belongs to the peroxiredoxin family. AhpC/Prx1 subfamily"

PRDX5 9606.ENSP00000265462 0.285117773 0.528312361 "rgb(117,239,172)" "Peroxiredoxin-5, mitochondrial; Thiol-specific peroxidase that catalyzes the reduction of hydrogen peroxide and organic hydroperoxides to water and alcohols, respectively. Plays a role in cell protection against oxidative stress by detoxifying peroxides and as sensor of hydrogen peroxide-mediated signaling events; Belongs to the peroxiredoxin family. Prx5 subfamily"

PRDX6 9606.ENSP00000342026 0.315952891 0.365766099 "rgb(220,239,117)" "Peroxiredoxin-6; Thiol-specific peroxidase that catalyzes the reduction of hydrogen peroxide and organic hydroperoxides to water and alcohols, respectively. Can reduce H(2)O(2) and short chain organic, fatty acid, and phospholipid hydroperoxides. Also has phospholipase activity, and can therefore either reduce the oxidized sn-2 fatty acyl grup of phospholipids (peroxidase activity) or hydrolyze the sn-2 ester bond of phospholipids (phospholipase activity). These activities are dependent on binding to phospholipids at acidic pH and to oxidized phospholipds at cytosolic pH. Plays a role [...] "

PRNP 9606.ENSP00000368752 0.318522484 0.57161362 "rgb(117,165,239)" Major prion protein; Its primary physiological function is unclear. May play a role in neuronal development and synaptic plasticity. May be required for neuronal myelin sheath maintenance. May promote myelin homeostasis through acting as a agonist for ADGRG6 receptor. May play a role in iron uptake and iron homeostasis. Soluble oligomers are toxic to cultured neuroblastoma cells and induce apoptosis (in vitro) (By similarity). Association with GPC1 (via its heparan sulfate chains) targets PRNP to lipid rafts. Also provides Cu(2+) or ZN(2+) for the ascorbate-mediated GPC1 deaminase degr [...]

QSOX1 9606.ENSP00000356574 0.355781585 0.608919319 "rgb(239,171,117)" "Sulfhydryl oxidase 1; Catalyzes the oxidation of sulfhydryl groups in peptide and protein thiols to disulfides with the reduction of oxygen to hydrogen peroxide. May contribute to disulfide bond formation in a variety of secreted proteins. In fibroblasts, it may have tumor- suppressing capabilities being involved in growth regulation; Belongs to the quiescin-sulfhydryl oxidase (QSOX) family"

QSOX2 9606.ENSP00000351536 0.364775161 0.64022946 "rgb(117,231,239)" Sulfhydryl oxidase 2; Catalyzes the oxidation of sulfhydryl groups in peptide and protein thiols to disulfides with the reduction of oxygen to hydrogen peroxide. May contribute to disulfide bond formation in a variety of secreted proteins. Also seems to play a role in regulating the sensitization of neuroblastoma cells for interferon-gamma-induced apoptosis

RNF7 9606.ENSP00000273480 0.65706646 0.648889711 "rgb(117,239,118)" "RING-box protein 2; Probable component of the SCF (SKP1-CUL1-F-box protein) E3 ubiquitin ligase complex which mediates the ubiquitination and subsequent proteasomal degradation of target proteins involved in cell cycle progression, signal transduction and transcription. CRLs complexes and ARIH1 collaborate in tandem to mediate ubiquitination of target proteins, ARIH1 mediating addition of the first ubiquitin on CRLs targets (By similarity). Through the RING-type zinc finger, seems to recruit the E2 ubiquitination enzyme to the complex and brings it into close proximity to the substrate [...] "

RUNX2 9606.ENSP00000360493 0.732226981 0.505662472 "rgb(117,156,239)" "Runt-related transcription factor 2; Transcription factor involved in osteoblastic differentiation and skeletal morphogenesis. Essential for the maturation of osteoblasts and both intramembranous and endochondral ossification. CBF binds to the core site, 5'-PYGPYGGT-3', of a number of enhancers and promoters, including murine leukemia virus, polyomavirus enhancer, T-cell receptor enhancers, osteocalcin, osteopontin, bone sialoprotein, alpha 1(I) collagen, LCK, IL-3 and GM-CSF promoters. In osteoblasts, supports transcription activation: synergizes with SPEN/MINT to enhance FGFR2-mediat [...] "

S100A7 9606.ENSP00000357712 0.62751606 0.637564767 "rgb(117,173,239)" S100 calcium binding protein A7; EF-hand domain containing

S100A8 9606.ENSP00000357722 0.64614561 0.510325685 "rgb(117,127,239)" "Protein S100-A8; S100A8 is a calcium- and zinc-binding protein which plays a prominent role in the regulation of inflammatory processes and immune response. It can induce neutrophil chemotaxis and adhesion. Predominantly found as calprotectin (S100A8/A9) which has a wide plethora of intra- and extracellular functions. The intracellular functions include: facilitating leukocyte arachidonic acid trafficking and metabolism, modulation of the tubulin-dependent cytoskeleton during migration of phagocytes and activation of the neutrophilic NADPH-oxidase. Activates NADPH- oxidase by facilitat [...] "

S100A9 9606.ENSP00000357727 0.675695931 0.462361214 "rgb(117,181,239)" "Protein S100-A9; S100A9 is a calcium- and zinc-binding protein which plays a prominent role in the regulation of inflammatory processes and immune response. It can induce neutrophil chemotaxis, adhesion, can increase the bactericidal activity of neutrophils by promoting phagocytosis via activation of SYK, PI3K/AKT, and ERK1/2 and can induce degranulation of neutrophils by a MAPK-dependent mechanism. Predominantly found as calprotectin (S100A8/A9) which has a wide plethora of intra- and extracellular functions. The intracellular functions include: facilitating leukocyte arachidonic acid [...] "

SCARA3 9606.ENSP00000301904 0.438008565 0.693523316 "rgb(187,255,101)" Scavenger receptor class A member 3; Seems to protect cells by scavenging oxidative molecules or harmful products of oxidation; Scavenger receptors

SEPP1 9606.ENSP00000420939 0.335867238 0.598260548 "rgb(117,239,143)" Selenoprotein P; Might be responsible for some of the extracellular antioxidant defense properties of selenium or might be involved in the transport of selenium. May supply selenium to tissues such as brain and testis

SESN2 9606.ENSP00000253063 0.249785867 0.539637306 "rgb(145,239,117)" "Sestrin-2; Functions as an intracellular leucine sensor that negatively regulates the TORC1 signaling pathway through the GATOR complex. In absence of leucine, binds the GATOR subcomplex GATOR2 and prevents TORC1 signaling. Binding of leucine to SESN2 disrupts its interaction with GATOR2 thereby activating the TORC1 signaling pathway. This stress-inducible metabolic regulator also plays a role in protection against oxidative and genotoxic stresses. May negatively regulate protein translation in response to endoplasmic reticulum stress, via TORC1. May positively regulate the transcripti [...] "

SGK2 9606.ENSP00000340608 0.542077088 0.698186528 "rgb(239,138,117)" "Serine/threonine-protein kinase Sgk2; Serine/threonine-protein kinase which is involved in the regulation of a wide variety of ion channels, membrane transporters, cell growth, survival and proliferation. Up- regulates Na(+) channels: SCNN1A/ENAC, K(+) channels: KCNA3/Kv1.3, KCNE1 and KCNQ1, amino acid transporter: SLC6A19, glutamate transporter: SLC1A6/EAAT4, glutamate receptors: GRIA1/GLUR1 and GRIK2/GLUR6, Na(+)/H(+) exchanger: SLC9A3/NHE3, and the Na(+)/K(+) ATPase"

SGK3 9606.ENSP00000379842 0.604389722 0.67553664 "rgb(117,117,239)" "Serum/glucocorticoid regulated kinase family, member 3; Serine/threonine-protein kinase which is involved in the regulation of a wide variety of ion channels, membrane transporters, cell growth, proliferation, survival and migration. Up-regulates Na(+) channels: SCNN1A/ENAC and SCN5A, K(+) channels: KCNA3/KV1.3, KCNE1, KCNQ1 and KCNH2/HERG, epithelial Ca(2+) channels: TRPV5 and TRPV6, chloride channel: BSND, creatine transporter: SLC6A8, Na(+)/dicarboxylate cotransporter: SLC13A2/NADC1, Na(+)-dependent phosphate cotransporter: SLC34A2/NAPI-2B, amino acid transporters: SLC1A5/ASCT2 and [...] "

SMOX 9606.ENSP00000478305 0.495182013 0.658882309 "rgb(117,239,164)" "Spermine oxidase; Flavoenzyme which catalyzes the oxidation of spermine to spermidine. Can also use N(1)-acetylspermine and spermidine as substrates, with different affinity depending on the isoform (isozyme) and on the experimental conditions. Plays an important role in the regulation of polyamine intracellular concentration and has the potential to act as a determinant of cellular sensitivity to the antitumor polyamine analogs. May contribute to beta-alanine production via aldehyde dehydrogenase conversion of 3-amino-propanal; Belongs to the flavin monoamine oxidase family"

SOD1 9606.ENSP00000270142 0.386616702 0.51765359 "rgb(117,239,135)" Superoxide dismutase [Cu-Zn]; Destroys radicals which are normally produced within the cells and which are toxic to biological systems

SOD2 9606.ENSP00000446252 0.410385439 0.568948927 "rgb(117,117,239)" "Superoxide dismutase [Mn], mitochondrial; Destroys superoxide anion radicals which are normally produced within the cells and which are toxic to biological systems"

SOD3 9606.ENSP00000371554 0.491327623 0.487675796 "rgb(117,117,239)" Extracellular superoxide dismutase [Cu-Zn]; Protect the extracellular space from toxic effect of reactive oxygen intermediates by converting superoxide radicals into hydrogen peroxide and oxygen

SP1 9606.ENSP00000329357 0.693683084 0.502331606 "rgb(239,192,117)" "Transcription factor Sp1; Transcription factor that can activate or repress transcription in response to physiological and pathological stimuli. Binds with high affinity to GC-rich motifs and regulates the expression of a large number of genes involved in a variety of processes such as cell growth, apoptosis, differentiation and immune responses. Highly regulated by post-translational modifications (phosphorylations, sumoylation, proteolytic cleavage, glycosylation and acetylation). Binds also the PDGFR- alpha G-box promoter. May have a role in modulating the cellular response to DNA d [...] "

SRXN1 9606.ENSP00000371388 0.270985011 0.340451517 "rgb(117,239,126)" "Sulfiredoxin-1; Contributes to oxidative stress resistance by reducing cysteine-sulfinic acid formed under exposure to oxidants in the peroxiredoxins PRDX1, PRDX2, PRDX3 and PRDX4. Does not act on PRDX5 or PRDX6. May catalyze the reduction in a multi-step process by acting both as a specific phosphotransferase and a thioltransferase; Belongs to the sulfiredoxin family"

STK25 9606.ENSP00000325748 0.487473233 0.700851221 "rgb(117,177,239)" "Serine/threonine-protein kinase 25; Oxidant stress-activated serine/threonine kinase that may play a role in the response to environmental stress. Targets to the Golgi apparatus where it appears to regulate protein transport events, cell adhesion, and polarity complexes important for cell migration"

SUOX 9606.ENSP00000377668 0.471413276 0.44703923 "rgb(117,117,239)" "Sulfite oxidase, mitochondrial; Sulfite oxidase"

TIMP2 9606.ENSP00000262768 0.838222698 0.600925241 "rgb(132,239,117)" "Metalloproteinase inhibitor 2; Complexes with metalloproteinases (such as collagenases) and irreversibly inactivates them by binding to their catalytic zinc cofactor. Known to act on MMP-1, MMP-2, MMP-3, MMP-7, MMP-8, MMP-9, MMP-10, MMP-13, MMP-14, MMP-15, MMP-16 and MMP-19; Belongs to the protease inhibitor I35 (TIMP) family"

TIMP3 9606.ENSP00000266085 0.783618844 0.555625463 "rgb(117,186,239)" "Metalloproteinase inhibitor 3; Complexes with metalloproteinases (such as collagenases) and irreversibly inactivates them by binding to their catalytic zinc cofactor. May form part of a tissue-specific acute response to remodeling stimuli. Known to act on MMP-1, MMP-2, MMP-3, MMP-7, MMP-9, MMP-13, MMP-14 and MMP-15"

TIMP4 9606.ENSP00000287814 0.875481799 0.58826795 "rgb(239,117,117)" "Metalloproteinase inhibitor 4; Complexes with metalloproteinases (such as collagenases) and irreversibly inactivates them by binding to their catalytic zinc cofactor. Known to act on MMP-1, MMP-2, MMP-3, MMP-7 and MMP- 9; Tissue inhibitor of metallopeptidases"

TXN 9606.ENSP00000363641 0.405888651 0.487675796 "rgb(14,178,0)" "Thioredoxin; Participates in various redox reactions through the reversible oxidation of its active center dithiol to a disulfide and catalyzes dithiol-disulfide exchange reactions. Plays a role in the reversible S-nitrosylation of cysteine residues in target proteins, and thereby contributes to the response to intracellular nitric oxide. Nitrosylates the active site Cys of CASP3 in response to nitric oxide (NO), and thereby inhibits caspase-3 activity. Induces the FOS/JUN AP-1 DNA-binding activity in ionizing radiation (IR) cells through its oxidation/reduction status and stimulates A [...] "

TXN2 9606.ENSP00000216185 0.358993576 0.331125093 "rgb(232,239,117)" "Thioredoxin, mitochondrial; Important for the control of mitochondrial reactive oxygen species homeostasis, apoptosis regulation and cell viability. Possesses a dithiol-reducing activity; Belongs to the thioredoxin family"

TXNRD1 9606.ENSP00000434516 0.355139186 0.510991858 "rgb(228,239,117)" "Thioredoxin reductase 1, cytoplasmic; Isoform 1 may possess glutaredoxin activity as well as thioredoxin reductase activity and induces actin and tubulin polymerization, leading to formation of cell membrane protrusions. Isoform 4 enhances the transcriptional activity of estrogen receptors alpha and beta while isoform 5 enhances the transcriptional activity of the beta receptor only. Isoform 5 also mediates cell death induced by a combination of interferon-beta and retinoic acid; Glutaredoxin domain containing"

TXNRD2 9606.ENSP00000383365 0.444432548 0.485011103 "rgb(117,239,168)" "Thioredoxin reductase 2, mitochondrial; Maintains thioredoxin in a reduced state. Implicated in the defenses against oxidative stress. May play a role in redox- regulated cell signaling; Belongs to the class-I pyridine nucleotide-disulfide oxidoreductase family"

UCP2 9606.ENSP00000312029 0.48875803 0.406402665 "rgb(239,121,117)" "Mitochondrial uncoupling protein 2; UCP are mitochondrial transporter proteins that create proton leaks across the inner mitochondrial membrane, thus uncoupling oxidative phosphorylation from ATP synthesis. As a result, energy is dissipated in the form of heat; Belongs to the mitochondrial carrier (TC 2.A.29) family"

VIMP 9606.ENSP00000381282 0.255567452 0.37642487 "rgb(149,239,117)" "Selenoprotein S; Involved in the degradation process of misfolded endoplasmic reticulum (ER) luminal proteins. Participates in the transfer of misfolded proteins from the ER to the cytosol, where they are destroyed by the proteasome in a ubiquitin-dependent manner. Probably acts by serving as a linker between DERL1, which mediates the retrotranslocation of misfolded proteins into the cytosol, and the ATPase complex VCP, which mediates the translocation and ubiquitination"
