# Supplementary material for: Rebalance of the Polyamine Metabolism Suppresses Oxidative Stress and Delays Senescence in Nucleus Pulposus Cells
Source: Oxid Med Cell Longev. 2022 Feb 7;2022:8033353. doi: 10.1155/2022/8033353 (PMC8844099; doi:10.1155/2022/8033353)
Supplement: Supplementary Materials — The raw data of the bioinformatics analysis containing DEGs, GO, KEGG, and PPI are provided in the supplementary file. [file 8033353.f1.zip › 8033353.f1/Supplementary file 1..docx]

**ID IDD2 Avg (log2) CON2 Avg (log2) Fold Change P-val FDR P-val Gene Symbol Description**

202437_s_at 7.79 3.39 21.19 0.04 0.9011 CYP1B1 "cytochrome P450, family 1, subfamily B, polypeptide 1"

210809_s_at 9.96 6.54 10.77 0.0031 0.9011 POSTN "periostin, osteoblast specific factor"

212353_at 6.04 2.84 9.15 0.0025 0.9011 SULF1 sulfatase 1

202436_s_at 8.72 5.61 8.67 0.0387 0.9011 CYP1B1 "cytochrome P450, family 1, subfamily B, polypeptide 1"

202207_at 7.63 4.57 8.35 0.0073 0.9011 ARL4C ADP-ribosylation factor like GTPase 4C

201842_s_at 8.25 5.25 8.03 0.0357 0.9011 EFEMP1 EGF containing fibulin-like extracellular matrix protein 1

212354_at 8.36 5.49 7.35 0.002 0.9011 SULF1 sulfatase 1

202435_s_at 7.45 5.01 5.41 0.0437 0.9011 CYP1B1 "cytochrome P450, family 1, subfamily B, polypeptide 1"

202609_at 7.04 4.72 4.97 0.0031 0.9011 EPS8 epidermal growth factor receptor pathway substrate 8

208966_x_at 5.72 3.82 3.74 0.0136 0.9011 IFI16 "interferon, gamma-inducible protein 16"

219602_s_at 5.21 3.37 3.58 0.0013 0.9011 PIEZO2 piezo-type mechanosensitive ion channel component 2

201069_at 7.04 5.22 3.54 0.0391 0.9011 MMP2 matrix metallopeptidase 2

201667_at 7.61 5.82 3.44 0.0046 0.9011 GJA1 gap junction protein alpha 1

213791_at 5.84 4.11 3.31 0.003 0.9011 PENK proenkephalin

202206_at 6.72 5.02 3.23 0.0223 0.9011 ARL4C ADP-ribosylation factor like GTPase 4C

210861_s_at 5.23 3.54 3.21 0.0041 0.9011 WISP3 WNT1 inducible signaling pathway protein 3

211161_s_at 8.34 6.66 3.19 0.0036 0.9011 COL3A1 "collagen, type III, alpha 1"

203477_at 8.32 6.65 3.18 0.0368 0.9011 COL15A1 "collagen, type XV, alpha 1"

212344_at 6.49 4.85 3.12 0.0009 0.9011 SULF1 sulfatase 1

218468_s_at 9.91 8.28 3.1 0.017 0.9011 GREM1 "gremlin 1, DAN family BMP antagonist"

212942_s_at 4.23 2.65 3 0.0073 0.9011 CEMIP "cell migration inducing protein, hyaluronan binding"

218469_at 8.99 7.43 2.95 0.0399 0.9011 GREM1 "gremlin 1, DAN family BMP antagonist"

213258_at 4.34 2.82 2.88 0.039 0.9011 TFPI tissue factor pathway inhibitor (lipoprotein-associated coagulation inhibitor)

205443_at 5.71 4.22 2.8 0.042 0.9011 SNAPC1 small nuclear RNA activating complex polypeptide 1

203438_at 5.6 4.16 2.72 0.0162 0.9011 STC2 stanniocalcin 2

201273_s_at 7.66 6.29 2.58 0.0307 0.9011 SRP9 signal recognition particle 9kDa

209655_s_at 5.1 3.81 2.45 0.0085 0.9011 TMEM47 transmembrane protein 47

209656_s_at 7.69 6.39 2.45 0.0183 0.9011 TMEM47 transmembrane protein 47

219049_at 6.8 5.51 2.45 0.0262 0.9011 CSGALNACT1 chondroitin sulfate N-acetylgalactosaminyltransferase 1

206332_s_at 5.09 3.81 2.43 0.0144 0.9011 IFI16 "interferon, gamma-inducible protein 16"

200063_s_at 9.77 8.49 2.42 0.0494 0.9011 NPM1 "nucleophosmin (nucleolar phosphoprotein B23, numatrin)"

205991_s_at 6.15 4.89 2.39 0.0166 0.9011 PRRX1 paired related homeobox 1

202729_s_at 6.29 5.04 2.36 0.0102 0.9011 LTBP1 latent transforming growth factor beta binding protein 1

212160_at 7.68 6.44 2.36 0.0325 0.9011 XPOT "exportin, tRNA"

201852_x_at 9.02 7.79 2.35 0.0174 0.9011 COL3A1 "collagen, type III, alpha 1"

208694_at 4.73 3.5 2.35 0.0409 0.9011 PRKDC "protein kinase, DNA-activated, catalytic polypeptide"

203889_at 4.97 3.76 2.32 0.0096 0.9011 SCG5 secretogranin V

215076_s_at 9.85 8.65 2.29 0.0218 0.9011 COL3A1 "collagen, type III, alpha 1"

204023_at 5.7 4.53 2.25 0.0021 0.9011 RFC4 replication factor C subunit 4

201745_at 5.89 4.72 2.25 0.0316 0.9011 TWF1 twinfilin actin binding protein 1

217915_s_at 7.25 6.09 2.24 0.0125 0.9011 RSL24D1 ribosomal L24 domain containing 1

200777_s_at 7.7 6.54 2.24 0.0236 0.9011 BZW1 basic leucine zipper and W2 domains 1

207657_x_at 7.62 6.45 2.24 0.028 0.9011 TNPO1 transportin 1

212610_at 6.94 5.77 2.24 0.0487 0.9011 PTPN11 "protein tyrosine phosphatase, non-receptor type 11"

201008_s_at 5.6 4.44 2.23 0.0053 0.9011 TXNIP thioredoxin interacting protein

209448_at 5.13 3.98 2.22 0.0009 0.9011 HTATIP2 HIV-1 Tat interactive protein 2

213909_at 6.51 5.38 2.2 0.0061 0.9011 LRRC15 leucine rich repeat containing 15

200873_s_at 8.58 7.44 2.2 0.0485 0.9011 CCT8 "chaperonin containing TCP1, subunit 8 (theta)"

201263_at 7.53 6.4 2.18 0.0486 0.9011 TARS threonyl-tRNA synthetase

202214_s_at 6.43 5.34 2.14 0.0154 0.9011 CUL4B cullin 4B

209392_at 3.83 2.74 2.13 0.026 0.9011 ENPP2 ectonucleotide pyrophosphatase/phosphodiesterase 2

202557_at 6.14 5.05 2.12 0.0265 0.9011 HSPA13 "heat shock protein 70kDa family, member 13"

206026_s_at 3.29 2.22 2.11 0.0113 0.9011 TNFAIP6 "tumor necrosis factor, alpha-induced protein 6"

201010_s_at 7.15 6.07 2.11 0.0355 0.9011 TXNIP thioredoxin interacting protein

203305_at 4.72 3.64 2.11 0.0364 0.9011 F13A1 "coagulation factor XIII, A1 polypeptide"

209095_at 6.42 5.35 2.1 0.0157 0.9011 DLD dihydrolipoamide dehydrogenase

207180_s_at 6.03 4.96 2.09 0.0181 0.9011 HTATIP2 HIV-1 Tat interactive protein 2

204944_at 3.92 2.87 2.08 0.0185 0.9011 PTPRG "protein tyrosine phosphatase, receptor type, G"

202171_at 5.46 4.4 2.08 0.0292 0.9011 VEZF1 vascular endothelial zinc finger 1

213009_s_at 6.02 4.97 2.08 0.0485 0.9011 TRIM37 tripartite motif containing 37

219293_s_at 7.73 6.68 2.07 0.0478 0.9011 OLA1 Obg-like ATPase 1

218645_at 3.68 2.66 2.02 0.008 0.9011 ZNF277 zinc finger protein 277

210145_at 4.72 3.7 2.02 0.0354 0.9011 PLA2G4A "phospholipase A2, group IVA (cytosolic, calcium-dependent)"

205053_at 4.94 3.93 2.02 0.0445 0.9011 PRIM1 "primase, DNA, polypeptide 1 (49kDa)"

201930_at 7.19 6.19 2 0.0225 0.9011 MCM6 minichromosome maintenance complex component 6

212582_at 5.47 4.47 2 0.0241 0.9011 OSBPL8 oxysterol binding protein-like 8

207483_s_at 6.1 5.1 2 0.028 0.9011 CAND1 cullin-associated and neddylation-dissociated 1

209955_s_at 5.78 4.78 1.99 0.0195 0.9011 FAP fibroblast activation protein alpha

209004_s_at 7.38 6.41 1.96 0.0031 0.9011 FBXL5 F-box and leucine-rich repeat protein 5

209598_at 5.55 4.58 1.96 0.0085 0.9011 PNMA2 paraneoplastic Ma antigen 2

219926_at 6.13 5.16 1.96 0.0382 0.9011 POPDC3 popeye domain containing 3

221829_s_at 7.96 7 1.94 0.0199 0.9011 TNPO1 transportin 1

201307_at 7.08 6.13 1.94 0.0212 0.9011 11-Sep septin 11

205174_s_at 3.07 2.13 1.91 0.0254 0.9011 QPCT glutaminyl-peptide cyclotransferase

201327_s_at 6.63 5.7 1.91 0.0341 0.9011 CCT6A "chaperonin containing TCP1, subunit 6A (zeta 1)"

216033_s_at 6.07 5.14 1.9 0.009 0.9011 FYN "FYN proto-oncogene, Src family tyrosine kinase"

202911_at 6.13 5.21 1.89 0.0141 0.9011 MSH6 mutS homolog 6

213150_at 5.42 4.51 1.89 0.0458 0.9011 HOXA10 homeobox A10

207305_s_at 5.63 4.72 1.88 0.0082 0.9011 TRAPPC8 trafficking protein particle complex 8

213429_at 4.66 3.75 1.88 0.0086 0.9011 BICC1 BicC family RNA binding protein 1

211954_s_at 7.85 6.94 1.88 0.0348 0.9011 IPO5 importin 5

214938_x_at 8.93 8.02 1.88 0.0443 0.9011 HMGB1 high mobility group box 1

204237_at 3.72 2.81 1.88 0.0469 0.9011 GULP1 "GULP, engulfment adaptor PTB domain containing 1"

213168_at 4.81 3.91 1.87 0.0079 0.9011 SP3 Sp3 transcription factor

213655_at 10.19 9.29 1.87 0.0102 0.9011

211971_s_at 6.27 5.37 1.87 0.0456 0.9011 LRPPRC leucine-rich pentatricopeptide repeat containing

218461_at 3.8 2.89 1.87 0.0462 0.9011 GPN3 GPN-loop GTPase 3

218323_at 5.01 4.12 1.86 0.0274 0.9011 RHOT1 ras homolog family member T1

211559_s_at 5.79 4.9 1.86 0.046 0.9011 CCNG2 cyclin G2

201291_s_at 4.18 3.3 1.84 0.0226 0.9011 TOP2A topoisomerase (DNA) II alpha

204048_s_at 4.91 4.03 1.84 0.0449 0.9011 PHACTR2 phosphatase and actin regulator 2

201215_at 7.42 6.56 1.82 0.0009 0.9011 PLS3 plastin 3

218578_at 4.38 3.51 1.82 0.0096 0.9011 CDC73 cell division cycle 73

201546_at 6.8 5.95 1.81 0.0097 0.9011 TRIP12 thyroid hormone receptor interactor 12

202969_at 5.47 4.61 1.81 0.0165 0.9011 DYRK2 dual specificity tyrosine-(Y)-phosphorylation regulated kinase 2

200953_s_at 4.35 3.49 1.81 0.0189 0.9011 CCND2 cyclin D2

201877_s_at 5.7 4.84 1.81 0.0295 0.9011 PPP2R5C "protein phosphatase 2, regulatory subunit B', gamma"

200008_s_at 8.4 7.55 1.81 0.038 0.9011 GDI2 GDP dissociation inhibitor 2

218718_at 8.29 7.44 1.8 0.0009 0.9011 PDGFC platelet derived growth factor C

208374_s_at 7.71 6.87 1.8 0.0031 0.9011 CAPZA1 "capping protein (actin filament) muscle Z-line, alpha 1"

217879_at 6 5.15 1.8 0.0312 0.9011 CDC27 cell division cycle 27

202173_s_at 4.24 3.4 1.79 0.0042 0.9011 VEZF1 vascular endothelial zinc finger 1

217966_s_at 4.82 3.98 1.79 0.0224 0.9011 FAM129A "family with sequence similarity 129, member A"

203075_at 5.19 4.36 1.79 0.029 0.9011 SMAD2 SMAD family member 2

202973_x_at 3.79 2.95 1.79 0.0376 0.9011 FAM13A "family with sequence similarity 13, member A"

214587_at 3.64 2.81 1.78 0.0202 0.9011 COL8A1 "collagen, type VIII, alpha 1"

204198_s_at 3.63 2.8 1.77 0.0059 0.9011 RUNX3 runt-related transcription factor 3

202727_s_at 4.72 3.9 1.77 0.0405 0.9011 IFNGR1 interferon gamma receptor 1

222148_s_at 4.86 4.05 1.76 0.0258 0.9011 RHOT1 ras homolog family member T1

200016_x_at 10.5 9.68 1.76 0.045 0.9011 HNRNPA1 heterogeneous nuclear ribonucleoprotein A1

212138_at 5.09 4.27 1.76 0.0496 0.9011 PDS5A PDS5 cohesin associated factor A

209579_s_at 5.28 4.46 1.75 0.0012 0.9011 MBD4 methyl-CpG binding domain 4 DNA glycosylase

218236_s_at 4.54 3.72 1.75 0.0221 0.9011 PRKD3 protein kinase D3

207983_s_at 4.87 4.06 1.75 0.0264 0.9011 STAG2 stromal antigen 2

217972_at 7.11 6.3 1.75 0.0271 0.9011 CHCHD3 coiled-coil-helix-coiled-coil-helix domain containing 3

202375_at 5.68 4.87 1.75 0.028 0.9011 SEC24D "SEC24 homolog D, COPII coat complex component"

219679_s_at 5.75 4.95 1.75 0.0284 0.9011 WAC WW domain containing adaptor with coiled-coil

203087_s_at 4.15 3.35 1.74 0.0153 0.9011 KIF2A kinesin heavy chain member 2A

217492_s_at 6.36 5.57 1.74 0.0211 0.9011 PTEN; PTENP1 phosphatase and tensin homolog; phosphatase and tensin homolog pseudogene 1 (functional)

218379_at 5.59 4.79 1.74 0.0485 0.9011 RBM7 RNA binding motif protein 7

212718_at 7.11 6.32 1.73 0.0221 0.9011 PAPOLA poly(A) polymerase alpha

201493_s_at 5.66 4.87 1.73 0.0242 0.9011 PUM2 pumilio RNA binding family member 2

200776_s_at 7.58 6.8 1.72 0.0007 0.9011 BZW1 basic leucine zipper and W2 domains 1

203300_x_at 5.41 4.62 1.72 0.0064 0.9011 AP1S2 adaptor-related protein complex 1 sigma 2 subunit

201101_s_at 5 4.22 1.72 0.0341 0.9011 BCLAF1 BCL2-associated transcription factor 1

203465_at 3.32 2.54 1.72 0.0405 0.9011 MRPL19 mitochondrial ribosomal protein L19

201653_at 8.72 7.93 1.72 0.0441 0.9011 CNIH1 cornichon family AMPA receptor auxiliary protein 1

209849_s_at 5.49 4.72 1.71 0.0319 0.9011 RAD51C RAD51 paralog C

207173_x_at 7.57 6.8 1.71 0.0372 0.9011 CDH11 "cadherin 11, type 2, OB-cadherin (osteoblast)"

221577_x_at 5.92 5.14 1.71 0.0417 0.9011 GDF15 growth differentiation factor 15

218953_s_at 5.84 5.07 1.71 0.0013 0.9011 PCYOX1L prenylcysteine oxidase 1 like

218603_at 4.97 4.2 1.7 0.0123 0.9011 HECA "hdc homolog, cell cycle regulator"

219147_s_at 4.79 4.03 1.7 0.0134 0.9011 NMRK1 nicotinamide riboside kinase 1

203688_at 3.87 3.1 1.7 0.0227 0.9011 PKD2 polycystic kidney disease 2 (autosomal dominant)

209687_at 3.97 3.2 1.7 0.0432 0.9011 CXCL12 chemokine (C-X-C motif) ligand 12

212984_at 3.38 2.61 1.7 0.0464 0.9011 ATF2 activating transcription factor 2

210257_x_at 4.28 3.52 1.69 0.0016 0.9011 CUL4B cullin 4B

213016_at 3.31 2.56 1.69 0.0182 0.9011 BBX bobby sox homolog (Drosophila)

203095_at 5.43 4.67 1.69 0.0195 0.9011 MTIF2 mitochondrial translational initiation factor 2

202172_at 4.87 4.11 1.69 0.0237 0.9011 VEZF1 vascular endothelial zinc finger 1

204726_at 4.34 3.58 1.69 0.0351 0.9011 CDH13 cadherin 13

211974_x_at 6.99 6.23 1.69 0.0408 0.9011 RBPJ recombination signal binding protein for immunoglobulin kappa J region

201009_s_at 6.59 5.83 1.69 0.0412 0.9011 TXNIP thioredoxin interacting protein

209033_s_at 6.34 5.59 1.68 0.0253 0.9011 DYRK1A dual specificity tyrosine-(Y)-phosphorylation regulated kinase 1A

214356_s_at 6.37 5.62 1.68 0.0406 0.9011 KIAA0368 KIAA0368

209822_s_at 5.42 4.67 1.67 0.0196 0.9011 VLDLR very low density lipoprotein receptor

218106_s_at 5.56 4.84 1.66 0.0013 0.9011 MRPS10 mitochondrial ribosomal protein S10

205475_at 9.41 8.68 1.66 0.0215 0.9011 SCRG1 stimulator of chondrogenesis 1

212836_at 5.19 4.45 1.66 0.0443 0.9011 POLD3 "polymerase (DNA-directed), delta 3, accessory subunit"

211988_at 7.23 6.5 1.65 0.0082 0.9011 SMARCE1 "SWI/SNF related, matrix associated, actin dependent regulator of chromatin, subfamily e, member 1"

211015_s_at 5.54 4.81 1.65 0.0278 0.9011 HSPA4 heat shock 70kDa protein 4

218643_s_at 4.39 3.66 1.65 0.0322 0.9011 CRIPT cysteine-rich PDZ-binding protein

209226_s_at 7.21 6.5 1.65 0.0396 0.9011 TNPO1 transportin 1

213594_x_at 4.81 4.09 1.65 0.0476 0.9011 SRSF10 serine/arginine-rich splicing factor 10

213026_at 4.24 3.53 1.64 0.0011 0.9011 ATG12 autophagy related 12

212740_at 4.53 3.82 1.64 0.0258 0.9011 PIK3R4 "phosphoinositide-3-kinase, regulatory subunit 4"

211612_s_at 4.91 4.19 1.64 0.0411 0.9011 IL13RA1 "interleukin 13 receptor, alpha 1"

218263_s_at 6.04 5.33 1.63 0.0065 0.9011 ZBED5 "zinc finger, BED-type containing 5"

219939_s_at 7.24 6.54 1.63 0.029 0.9011 CSDE1 "cold shock domain containing E1, RNA binding"

218090_s_at 4.87 4.18 1.62 0.0027 0.9011 WDR11 WD repeat domain 11

203269_at 6.86 6.17 1.62 0.0039 0.9011 NSMAF neutral sphingomyelinase activation associated factor

217908_s_at 6.09 5.4 1.62 0.0077 0.9011 DCAF6 DDB1 and CUL4 associated factor 6

201626_at 7.88 7.18 1.62 0.0111 0.9011 INSIG1 insulin induced gene 1

210283_x_at 5.63 4.94 1.62 0.0298 0.9011 PAIP1 poly(A) binding protein interacting protein 1

208095_s_at 7.96 7.27 1.61 0.0025 0.9011 SRP72 signal recognition particle 72kDa

211450_s_at 4.21 3.52 1.61 0.0116 0.9011 MSH6 mutS homolog 6

200624_s_at 7.25 6.56 1.61 0.0269 0.9011 MATR3 matrin 3

201761_at 8.13 7.44 1.61 0.0306 0.9011 MTHFD2 "methylenetetrahydrofolate dehydrogenase (NADP+ dependent) 2, methenyltetrahydrofolate cyclohydrolase"

203177_x_at 3.02 2.33 1.61 0.0467 0.9011 TFAM "transcription factor A, mitochondrial"

213229_at 4.21 3.53 1.61 0.0469 0.9011 DICER1 "dicer 1, ribonuclease type III"

213194_at 4.96 4.28 1.61 0.0478 0.9011 ROBO1 roundabout guidance receptor 1

218905_at 5.91 5.23 1.6 0.0043 0.9011 INTS8 integrator complex subunit 8

201515_s_at 7.26 6.58 1.6 0.0152 0.9011 TSN translin

202213_s_at 5.45 4.77 1.6 0.0158 0.9011 CUL4B cullin 4B

213170_at 6.3 5.63 1.59 0.0051 0.9011 GPX7 glutathione peroxidase 7

205240_at 4.52 3.85 1.59 0.0057 0.9011 GPSM2 G-protein signaling modulator 2

202739_s_at 4.02 3.36 1.59 0.0071 0.9011 PHKB "phosphorylase kinase, beta"

217887_s_at 6.37 5.7 1.59 0.0089 0.9011 EPS15 epidermal growth factor receptor pathway substrate 15

221970_s_at 6.13 5.46 1.59 0.0169 0.9011 NOL11 nucleolar protein 11

202147_s_at 3.92 3.25 1.59 0.0186 0.9011 IFRD1 interferon-related developmental regulator 1

213624_at 3.63 2.95 1.59 0.0452 0.9011 SMPDL3A "sphingomyelin phosphodiesterase, acid-like 3A"

205048_s_at 6.85 6.19 1.59 0.0464 0.9011 PSPH phosphoserine phosphatase

220329_s_at 4.17 3.52 1.58 0.0013 0.9011 RMND1 required for meiotic nuclear division 1 homolog

206562_s_at 6.83 6.17 1.58 0.0051 0.9011 CSNK1A1 "casein kinase 1, alpha 1"

208800_at 6.13 5.48 1.58 0.0113 0.9011 SRP72 signal recognition particle 72kDa

209484_s_at 6.16 5.5 1.58 0.0254 0.9011 NSL1 "NSL1, MIS12 kinetochore complex component"

214323_s_at 4.4 3.74 1.58 0.029 0.9011 UPF3A UPF3 regulator of nonsense transcripts homolog A (yeast)

212350_at 6.2 5.54 1.58 0.0296 0.9011 TBC1D1 "TBC1 (tre-2/USP6, BUB2, cdc16) domain family, member 1"

201917_s_at 4.54 3.88 1.58 0.0336 0.9011 SLC25A36 "solute carrier family 25 (pyrimidine nucleotide carrier), member 36"

201319_at 8.07 7.4 1.58 0.0449 0.9011 MYL12A myosin light chain 12A

218710_at 6.11 5.46 1.57 0.0124 0.9011 TTC27 tetratricopeptide repeat domain 27

213086_s_at 7.59 6.93 1.57 0.0163 0.9011 CSNK1A1 "casein kinase 1, alpha 1"

209112_at 4.86 4.2 1.57 0.0236 0.9011 CDKN1B "cyclin-dependent kinase inhibitor 1B (p27, Kip1)"

207956_x_at 3.79 3.14 1.57 0.0263 0.9011 PDS5B PDS5 cohesin associated factor B

201737_s_at 5.79 5.13 1.57 0.032 0.9011 6-Mar membrane associated ring finger 6

205608_s_at 4.08 3.43 1.57 0.0333 0.9011 ANGPT1 angiopoietin 1

200599_s_at 7.67 7.01 1.57 0.0374 0.9011 HSP90B1 "heat shock protein 90kDa beta (Grp94), member 1"

204774_at 3.38 2.73 1.57 0.0434 0.9011 EVI2A ecotropic viral integration site 2A

214429_at 5.79 5.13 1.57 0.0497 0.9011 MTMR6 myotubularin related protein 6

209669_s_at 6.56 5.91 1.56 0.0026 0.9011 SERBP1 SERPINE1 mRNA binding protein 1

210105_s_at 7.17 6.53 1.56 0.0094 0.9011 FYN "FYN proto-oncogene, Src family tyrosine kinase"

201386_s_at 7.71 7.07 1.56 0.0239 0.9011 DHX15 DEAH (Asp-Glu-Ala-His) box helicase 15

206555_s_at 3.48 2.84 1.56 0.031 0.9011 THUMPD1 THUMP domain containing 1

200728_at 7.07 6.43 1.56 0.0336 0.9011 ACTR2 ARP2 actin-related protein 2 homolog (yeast)

211969_at 8.55 7.91 1.56 0.0371 0.9011 HSP90AA1 "heat shock protein 90kDa alpha (cytosolic), class A member 1"

211953_s_at 6.86 6.21 1.56 0.0405 0.9011 IPO5 importin 5

206874_s_at 3.33 2.69 1.56 0.0433 0.9011 SLK STE20-like kinase

209310_s_at 6.08 5.45 1.55 0.0057 0.9011 CASP4 caspase 4

202614_at 5.44 4.8 1.55 0.029 0.9011 SLC30A9 "solute carrier family 30 (zinc transporter), member 9"

209098_s_at 5.58 4.95 1.55 0.0323 0.9011 JAG1 jagged 1

212459_x_at 6.74 6.1 1.55 0.0392 0.9011 SUCLG2 "succinate-CoA ligase, GDP-forming, beta subunit"

209316_s_at 6.04 5.42 1.54 0.0011 0.9011 HBS1L HBS1-like translational GTPase

206796_at 5.75 5.12 1.54 0.002 0.9011 WISP1 WNT1 inducible signaling pathway protein 1

212251_at 7.73 7.1 1.54 0.002 0.9011 MTDH metadherin

200605_s_at 7.31 6.69 1.54 0.0111 0.9011 PRKAR1A "protein kinase, cAMP-dependent, regulatory, type I, alpha"

217742_s_at 5.56 4.94 1.54 0.0114 0.9011 WAC WW domain containing adaptor with coiled-coil

202049_s_at 4.32 3.7 1.54 0.0181 0.9011 ZMYM4 "zinc finger, MYM-type 4"

204297_at 6.02 5.4 1.54 0.0298 0.9011 PIK3C3 "phosphatidylinositol 3-kinase, catalytic subunit type 3"

209056_s_at 5.4 4.78 1.54 0.0314 0.9011 CDC5L cell division cycle 5-like

202558_s_at 3.99 3.37 1.54 0.0359 0.9011 HSPA13 "heat shock protein 70kDa family, member 13"

217789_at 6.48 5.85 1.54 0.0469 0.9011 LOC105370447; SNX6 uncharacterized LOC105370447; sorting nexin 6

202981_x_at 5.72 5.1 1.53 0.0018 0.9011 SIAH1 siah E3 ubiquitin protein ligase 1

210681_s_at 4.29 3.68 1.53 0.0025 0.9011 USP15 ubiquitin specific peptidase 15

204185_x_at 5.15 4.53 1.53 0.0071 0.9011 PPID peptidylprolyl isomerase D

211955_at 7.59 6.97 1.53 0.0144 0.9011 IPO5 importin 5

212535_at 5.94 5.32 1.53 0.0168 0.9011 MEF2A myocyte enhancer factor 2A

200778_s_at 8.09 7.48 1.53 0.0179 0.9011 2-Sep septin 2

217127_at 3.25 2.64 1.53 0.0216 0.9011 CTH cystathionine gamma-lyase

203247_s_at 5.68 5.07 1.53 0.0258 0.9011 ZNF24 zinc finger protein 24

212449_s_at 6.29 5.68 1.53 0.0314 0.9011 LYPLA1 lysophospholipase I

202766_s_at 9.86 9.25 1.53 0.0391 0.9011 FBN1 fibrillin 1

210664_s_at 4.05 3.43 1.53 0.0417 0.9011 TFPI tissue factor pathway inhibitor (lipoprotein-associated coagulation inhibitor)

206025_s_at 3.49 2.89 1.52 0.0049 0.9011 TNFAIP6 "tumor necrosis factor, alpha-induced protein 6"

203338_at 6.02 5.42 1.52 0.0127 0.9011 PPP2R5E "protein phosphatase 2, regulatory subunit B', epsilon isoform"

204835_at 4.09 3.49 1.52 0.0225 0.9011 POLA1 "polymerase (DNA directed), alpha 1, catalytic subunit"

216221_s_at 5.7 5.09 1.52 0.0274 0.9011 PUM2 pumilio RNA binding family member 2

203076_s_at 6.95 6.35 1.52 0.0325 0.9011 SMAD2 SMAD family member 2

221492_s_at 6.3 5.69 1.52 0.0337 0.9011 ATG3 autophagy related 3

209115_at 5.24 4.63 1.52 0.0338 0.9011 UBA3 ubiquitin-like modifier activating enzyme 3

209284_s_at 3.6 3 1.52 0.036 0.9011 FAM208A "family with sequence similarity 208, member A"

212008_at 6.18 5.57 1.52 0.0374 0.9011 UBXN4 UBX domain protein 4

212851_at 5 4.39 1.52 0.038 0.9011 DCUN1D4 "DCN1, defective in cullin neddylation 1, domain containing 4"

201803_at 6.71 6.1 1.52 0.0462 0.9011 POLR2B "polymerase (RNA) II (DNA directed) polypeptide B, 140kDa"

213391_at 4.52 3.93 1.51 0.0014 0.9011 DPY19L4 dpy-19-like 4 (C. elegans)

213161_at 5.4 4.81 1.51 0.0038 0.9011 TMOD1; TSTD2 tropomodulin 1; thiosulfate sulfurtransferase (rhodanese)-like domain containing 2

211719_x_at 10.93 10.33 1.51 0.0045 0.9011 FN1 fibronectin 1

219596_at 3.88 3.29 1.51 0.0052 0.9011 THAP10 THAP domain containing 10

202368_s_at 6.99 6.4 1.51 0.006 0.9011 TRAM2 translocation associated membrane protein 2

213015_at 4.54 3.95 1.51 0.0103 0.9011 BBX bobby sox homolog (Drosophila)

218171_at 4.37 3.78 1.51 0.0182 0.9011 VPS4B vacuolar protein sorting 4 homolog B (S. cerevisiae)

214949_at 5.27 4.68 1.51 0.0225 0.9011

213860_x_at 7.61 7.02 1.51 0.0348 0.9011 CSNK1A1 "casein kinase 1, alpha 1"

217047_s_at 3.98 3.38 1.51 0.0381 0.9011 FAM13A "family with sequence similarity 13, member A"

216241_s_at 8.72 8.13 1.51 0.043 0.9011 TCEA1 "transcription elongation factor A (SII), 1"

205805_s_at 4.37 3.79 1.5 0.0088 0.9011 ROR1 receptor tyrosine kinase-like orphan receptor 1

201518_at 6.65 6.06 1.5 0.0095 0.9011 CBX1 chromobox homolog 1

217496_s_at 5.05 4.46 1.5 0.0143 0.9011 IDE insulin-degrading enzyme

221782_at 3.95 3.36 1.5 0.0198 0.9011 DNAJC10 "DnaJ (Hsp40) homolog, subfamily C, member 10"

208852_s_at 6.78 6.2 1.5 0.0303 0.9011 CANX calnexin

213246_at 4.94 4.36 1.5 0.0344 0.9011 TMEM251 transmembrane protein 251

219007_at 5.13 4.55 1.49 0.01 0.9011 NUP43 nucleoporin 43kDa

203567_s_at 4.94 4.36 1.49 0.0106 0.9011 TRIM38 tripartite motif containing 38

201506_at 10.77 10.2 1.49 0.013 0.9011 TGFBI "transforming growth factor, beta-induced, 68kDa"

202915_s_at 4.91 4.33 1.49 0.0196 0.9011 FAM20B "family with sequence similarity 20, member B"

203374_s_at 5.05 4.48 1.49 0.0271 0.9011 TPP2 tripeptidyl peptidase II

204194_at 3.09 2.52 1.49 0.0275 0.9011 BACH1 "BTB and CNC homology 1, basic leucine zipper transcription factor 1"

201166_s_at 7.38 6.81 1.49 0.0299 0.9011 PUM1 pumilio RNA binding family member 1

203007_x_at 5.8 5.24 1.48 0.0072 0.9011 LYPLA1 lysophospholipase I

203238_s_at 5.76 5.19 1.48 0.0089 0.9011 NOTCH3 notch 3

218329_at 5.88 5.31 1.48 0.0115 0.9011 PRDM4 PR domain containing 4

217881_s_at 4.99 4.43 1.48 0.0168 0.9011 CDC27 cell division cycle 27

209274_s_at 5.53 4.96 1.48 0.0178 0.9011 ISCA1 iron-sulfur cluster assembly 1

218534_s_at 3.85 3.28 1.48 0.0302 0.9011 AGGF1 angiogenic factor with G-patch and FHA domains 1

202453_s_at 5.74 5.17 1.48 0.0328 0.9011 GTF2H1 general transcription factor IIH subunit 1

200723_s_at 8.77 8.2 1.48 0.0335 0.9011 CAPRIN1 cell cycle associated protein 1

217845_x_at 8.26 7.7 1.48 0.0369 0.9011 HIGD1A "HIG1 hypoxia inducible domain family, member 1A"

210154_at 4.6 4.04 1.48 0.042 0.9011 ME2 "malic enzyme 2, NAD(+)-dependent, mitochondrial"

221568_s_at 4.13 3.57 1.48 0.0423 0.9011 LIN7C lin-7 homolog C (C. elegans)

200015_s_at 8.91 8.34 1.48 0.0448 0.9011 2-Sep septin 2

209545_s_at 6.76 6.2 1.47 0.0035 0.9011 RIPK2 receptor-interacting serine-threonine kinase 2

218251_at 6.15 5.6 1.47 0.0057 0.9011 MID1IP1 MID1 interacting protein 1

200833_s_at 8.46 7.9 1.47 0.0077 0.9011 RAP1B "RAP1B, member of RAS oncogene family"

203610_s_at 4.91 4.35 1.47 0.0202 0.9011 TRIM38 tripartite motif containing 38

212022_s_at 4.47 3.91 1.47 0.0331 0.9011 MKI67 marker of proliferation Ki-67

202565_s_at 5.8 5.24 1.47 0.0406 0.9011 SVIL supervillin

202124_s_at 5.16 4.6 1.47 0.0491 0.9011 TRAK2 "trafficking protein, kinesin binding 2"

221896_s_at 7.28 6.73 1.47 0.0498 0.9011 HIGD1A "HIG1 hypoxia inducible domain family, member 1A"

201919_at 6.14 5.6 1.46 0.0066 0.9011 SLC25A36 "solute carrier family 25 (pyrimidine nucleotide carrier), member 36"

212746_s_at 4.53 3.98 1.46 0.0216 0.9011 CEP170 centrosomal protein 170kDa

214724_at 5.67 5.13 1.46 0.026 0.9011 DIXDC1 DIX domain containing 1

212181_s_at 5.97 5.42 1.46 0.033 0.9011 NUDT4 nudix hydrolase 4

202265_at 4.19 3.65 1.46 0.0331 0.9011 BMI1 "BMI1 proto-oncogene, polycomb ring finger"

222182_s_at 5.72 5.17 1.46 0.0356 0.9011 CNOT2 CCR4-NOT transcription complex subunit 2

221749_at 5.56 5.01 1.46 0.0444 0.9011 YTHDF3 YTH N(6)-methyladenosine RNA binding protein 3

214720_x_at 5.18 4.63 1.46 0.0471 0.9011 10-Sep septin 10

215160_x_at 4.61 4.06 1.46 0.0492 0.9011 FRG1CP "FSHD region gene 1 family member C, pseudogene"

201133_s_at 5.53 4.99 1.45 0.0036 0.9011 PJA2 "praja ring finger 2, E3 ubiquitin protein ligase"

217718_s_at 9.36 8.83 1.45 0.0271 0.9011 YWHAB "tyrosine 3-monooxygenase/tryptophan 5-monooxygenase activation protein, beta"

200040_at 6.97 6.44 1.45 0.0322 0.9011 KHDRBS1 "KH domain containing, RNA binding, signal transduction associated 1"

218975_at 5.56 5.02 1.45 0.0353 0.9011 COL5A3 "collagen, type V, alpha 3"

212676_at 4.12 3.59 1.44 0.0017 0.9011 NF1 neurofibromin 1

203835_at 5.73 5.2 1.44 0.0131 0.9011 LRRC32 leucine rich repeat containing 32

218929_at 3.77 3.24 1.44 0.018 0.9011 CDKN2AIP CDKN2A interacting protein

208731_at 6.84 6.32 1.44 0.0263 0.9011 RAB2A "RAB2A, member RAS oncogene family"

202125_s_at 5.49 4.97 1.44 0.029 0.9011 TRAK2 "trafficking protein, kinesin binding 2"

208753_s_at 6.29 5.77 1.44 0.0331 0.9011 NAP1L1 nucleosome assembly protein 1-like 1

202271_at 4.52 4 1.44 0.0375 0.9011 FBXO28 F-box protein 28

220761_s_at 3.56 3.04 1.44 0.0395 0.9011 TAOK3 TAO kinase 3

203232_s_at 4.49 3.97 1.44 0.0407 0.9011 ATXN1 ataxin 1

218768_at 4.94 4.41 1.44 0.0456 0.9011 NUP107 nucleoporin 107kDa

201121_s_at 7.66 7.13 1.44 0.0492 0.9011 PGRMC1 progesterone receptor membrane component 1

202813_at 4.81 4.3 1.43 0.0121 0.9011 TARBP1 TAR (HIV-1) RNA binding protein 1

205070_at 4.55 4.03 1.43 0.0162 0.9011 ING3 inhibitor of growth family member 3

208485_x_at 4.17 3.65 1.43 0.023 0.9011 CFLAR CASP8 and FADD like apoptosis regulator

200841_s_at 5.15 4.63 1.43 0.0284 0.9011 EPRS glutamyl-prolyl-tRNA synthetase

200662_s_at 7.61 7.1 1.43 0.0354 0.9011 TOMM20 translocase of outer mitochondrial membrane 20 homolog (yeast)

204222_s_at 7.21 6.69 1.43 0.0486 0.9011 GLIPR1 GLI pathogenesis-related 1

208642_s_at 8.52 8.01 1.42 0.0041 0.9011 XRCC5 X-ray repair complementing defective repair in Chinese hamster cells 5 (double-strand-break rejoining)

215773_x_at 6.14 5.63 1.42 0.0167 0.9011 PARP2 poly(ADP-ribose) polymerase 2

202499_s_at 3.92 3.41 1.42 0.0169 0.9011 SLC2A3 "solute carrier family 2 (facilitated glucose transporter), member 3"

217743_s_at 5.1 4.6 1.42 0.0187 0.9011 COX7A2; TMEM30A cytochrome c oxidase subunit VIIa polypeptide 2 (liver); transmembrane protein 30A

212408_at 6.31 5.8 1.42 0.0261 0.9011 TOR1AIP1 torsin A interacting protein 1

201652_at 7.51 7.01 1.42 0.0278 0.9011 COPS5 COP9 signalosome subunit 5

209630_s_at 4.44 3.94 1.42 0.0292 0.9011 FBXW2 F-box and WD repeat domain containing 2

204703_at 3.59 3.08 1.42 0.0307 0.9011 IFT88 intraflagellar transport 88

213388_at 4.55 4.04 1.42 0.0339 0.9011 PDE4DIP phosphodiesterase 4D interacting protein

213356_x_at 10.33 9.82 1.42 0.0341 0.9011 HNRNPA1; HNRNPA1L2; HNRNPA1P10 heterogeneous nuclear ribonucleoprotein A1; heterogeneous nuclear ribonucleoprotein A1-like 2; heterogeneous nuclear ribonucleoprotein A1 pseudogene 10

201918_at 4.6 4.1 1.41 0.0068 0.9011 SLC25A36 "solute carrier family 25 (pyrimidine nucleotide carrier), member 36"

213093_at 6.83 6.34 1.41 0.0155 0.9011 PRKCA "protein kinase C, alpha"

221873_at 4.02 3.53 1.41 0.0174 0.9011 ZNF143 zinc finger protein 143

201051_at 5.77 5.27 1.41 0.0182 0.9011 ANP32A acidic nuclear phosphoprotein 32 family member A

203321_s_at 4.45 3.95 1.41 0.0213 0.9011 ADNP2 ADNP homeobox 2

212958_x_at 6.62 6.13 1.41 0.0329 0.9011 PAM peptidylglycine alpha-amidating monooxygenase

210261_at 3.34 2.85 1.41 0.0346 0.9011 KCNK2 "potassium channel, two pore domain subfamily K, member 2"

203250_at 5.12 4.62 1.41 0.0401 0.9011 SCAF8 SR-related CTD-associated factor 8

219206_x_at 7.26 6.76 1.41 0.0421 0.9011 TMBIM4 transmembrane BAX inhibitor motif containing 4

219032_x_at 4.85 4.36 1.4 0.004 0.9011 OPN3 opsin 3

200773_x_at 9.88 9.39 1.4 0.0092 0.9011 PTMA "prothymosin, alpha"

203328_x_at 4.82 4.33 1.4 0.0094 0.9011 IDE insulin-degrading enzyme

209659_s_at 7.18 6.7 1.4 0.0127 0.9011 CDC16 cell division cycle 16

209862_s_at 3.6 3.12 1.4 0.0129 0.9011 CEP57 centrosomal protein 57kDa

202874_s_at 5.24 4.75 1.4 0.0206 0.9011 ATP6V1C1 "ATPase, H+ transporting, lysosomal 42kDa, V1 subunit C1"

202521_at 5.86 5.38 1.4 0.025 0.9011 CTCF CCCTC-binding factor (zinc finger protein)

218454_at 5.52 5.03 1.4 0.0404 0.9011 PLBD1 phospholipase B domain containing 1

203428_s_at 4.77 4.28 1.4 0.0405 0.9011 ASF1A anti-silencing function 1A histone chaperone

202864_s_at 5.39 4.92 1.39 0.0102 0.9011 SP100 SP100 nuclear antigen

205505_at 5.1 4.63 1.39 0.011 0.9011 GCNT1 "glucosaminyl (N-acetyl) transferase 1, core 2"

203704_s_at 5.4 4.93 1.39 0.0173 0.9011 RREB1 ras responsive element binding protein 1

200009_at 10.3 9.83 1.39 0.0221 0.9011 GDI2 GDP dissociation inhibitor 2

204797_s_at 5.56 5.09 1.39 0.0231 0.9011 EML1 echinoderm microtubule associated protein like 1

214109_at 2.94 2.46 1.39 0.0271 0.9011 LRBA "LPS-responsive vesicle trafficking, beach and anchor containing"

215641_at 2.5 2.03 1.39 0.0337 0.9011 SEC24D "SEC24 homolog D, COPII coat complex component"

208743_s_at 7.49 7.01 1.39 0.0383 0.9011 YWHAB "tyrosine 3-monooxygenase/tryptophan 5-monooxygenase activation protein, beta"

212794_s_at 3.21 2.74 1.39 0.0411 0.9011 KIAA1033 KIAA1033

208638_at 7.88 7.41 1.39 0.0434 0.9011 PDIA6 "protein disulfide isomerase family A, member 6"

218946_at 6.52 6.06 1.38 0.0073 0.9011 NFU1 NFU1 iron-sulfur cluster scaffold

211938_at 7.6 7.14 1.38 0.02 0.9011 EIF4B eukaryotic translation initiation factor 4B

208839_s_at 4.74 4.27 1.38 0.039 0.9011 CAND1 cullin-associated and neddylation-dissociated 1

221311_x_at 4.8 4.35 1.37 0.0044 0.9011 LYRM2 LYR motif containing 2

218139_s_at 4.4 3.94 1.37 0.0045 0.9011 AP5M1 "adaptor-related protein complex 5, mu 1 subunit"

201306_s_at 9.71 9.25 1.37 0.0104 0.9011 ANP32B acidic nuclear phosphoprotein 32 family member B

213435_at 2.95 2.5 1.37 0.0127 0.9011 SATB2 SATB homeobox 2

219770_at 4.36 3.91 1.37 0.0151 0.9011 GTDC1 glycosyltransferase like domain containing 1

202971_s_at 3.07 2.61 1.37 0.0304 0.9011 DYRK2 dual specificity tyrosine-(Y)-phosphorylation regulated kinase 2

202900_s_at 6.79 6.34 1.37 0.0344 0.9011 NUP88 nucleoporin 88kDa

218854_at 6.33 5.87 1.37 0.0344 0.9011 DSE dermatan sulfate epimerase

201928_at 5.24 4.78 1.37 0.035 0.9011 PKP4 plakophilin 4

214352_s_at 4.99 4.54 1.37 0.0388 0.9011 KRAS Kirsten rat sarcoma viral oncogene homolog

206860_s_at 4.88 4.43 1.37 0.0436 0.9011 MIOS "missing oocyte, meiosis regulator, homolog (Drosophila)"

209709_s_at 4.07 3.61 1.37 0.0462 0.9011 HMMR hyaluronan-mediated motility receptor (RHAMM)

209268_at 5.33 4.88 1.37 0.0478 0.9011 VPS45 vacuolar protein sorting 45 homolog (S. cerevisiae)

216044_x_at 4.33 3.88 1.37 0.0489 0.9011 FAM69A "family with sequence similarity 69, member A"

212330_at 7.24 6.8 1.36 0.0052 0.9011 TFDP1 transcription factor Dp-1

202717_s_at 6.48 6.03 1.36 0.0079 0.9011 CDC16 cell division cycle 16

202583_s_at 4.98 4.53 1.36 0.013 0.9011 RANBP9 RAN binding protein 9

212217_at 4.52 4.08 1.36 0.0151 0.9011 PREPL prolyl endopeptidase-like

212073_at 4.91 4.47 1.36 0.0175 0.9011 CSNK2A1 "casein kinase 2, alpha 1 polypeptide"

201687_s_at 5.55 5.11 1.36 0.0206 0.9011 API5 apoptosis inhibitor 5

218211_s_at 7.6 7.16 1.36 0.0267 0.9011 MLPH melanophilin

212072_s_at 6.65 6.21 1.36 0.0278 0.9011 CSNK2A1 "casein kinase 2, alpha 1 polypeptide"

209682_at 4.17 3.73 1.36 0.0315 0.9011 CBLB "Cbl proto-oncogene B, E3 ubiquitin protein ligase"

219599_at 6.1 5.66 1.36 0.0344 0.9011

212216_at 6.31 5.87 1.36 0.0392 0.9011 PREPL prolyl endopeptidase-like

202816_s_at 4.12 3.68 1.36 0.0412 0.9011 SS18 "synovial sarcoma translocation, chromosome 18"

212447_at 4.47 4.02 1.36 0.0431 0.9011 KBTBD2 kelch repeat and BTB (POZ) domain containing 2

208853_s_at 6.6 6.15 1.36 0.0432 0.9011 CANX calnexin

212184_s_at 4.38 3.94 1.36 0.0491 0.9011 TAB2 TGF-beta activated kinase 1/MAP3K7 binding protein 2

206158_s_at 8.68 8.24 1.35 0.0082 0.9011 CNBP "CCHC-type zinc finger, nucleic acid binding protein"

201089_at 7.49 7.07 1.35 0.0111 0.9011 ATP6V1B2 "ATPase, H+ transporting, lysosomal 56/58kDa, V1 subunit B2"

222230_s_at 8.4 7.96 1.35 0.0143 0.9011 ACTR10 actin-related protein 10 homolog (S. cerevisiae)

205964_at 3.21 2.78 1.35 0.0223 0.9011 ZNF426 zinc finger protein 426

202566_s_at 4.95 4.52 1.35 0.0349 0.9011 SVIL supervillin

200854_at 4.91 4.48 1.35 0.0362 0.9011 NCOR1 nuclear receptor corepressor 1

203595_s_at 4.83 4.4 1.35 0.0363 0.9011 IFIT5 interferon-induced protein with tetratricopeptide repeats 5

212853_at 3.9 3.46 1.35 0.0422 0.9011 DCUN1D4 "DCN1, defective in cullin neddylation 1, domain containing 4"

212688_at 4.2 3.76 1.35 0.0425 0.9011 PIK3CB "phosphatidylinositol-4,5-bisphosphate 3-kinase, catalytic subunit beta"

204900_x_at 5.43 4.99 1.35 0.0431 0.9011 SAP30 Sin3A associated protein 30kDa

212131_at 5.05 4.61 1.35 0.0465 0.9011 LSM14A LSM14A mRNA processing body assembly factor

212632_at 5.04 4.61 1.35 0.0478 0.9011 STX7 syntaxin 7

208993_s_at 2.88 2.45 1.35 0.0485 0.9011 PPIG peptidylprolyl isomerase G (cyclophilin G)

201956_s_at 7.36 6.94 1.34 0.011 0.9011 GNPAT glyceronephosphate O-acyltransferase

218352_at 2.59 2.17 1.34 0.015 0.9011 RCBTB1 regulator of chromosome condensation (RCC1) and BTB (POZ) domain containing protein 1

201120_s_at 6.74 6.31 1.34 0.0163 0.9011 PGRMC1 progesterone receptor membrane component 1

218947_s_at 3.15 2.73 1.34 0.022 0.9011 MTPAP mitochondrial poly(A) polymerase

202333_s_at 6.24 5.81 1.34 0.0306 0.9011 UBE2B ubiquitin conjugating enzyme E2B

206247_at 4.93 4.51 1.34 0.0313 0.9011 MICB MHC class I polypeptide-related sequence B

201241_at 7.02 6.6 1.34 0.0356 0.9011 DDX1 DEAD (Asp-Glu-Ala-Asp) box helicase 1

221558_s_at 3.7 3.27 1.34 0.0396 0.9011 LEF1 lymphoid enhancer-binding factor 1

208643_s_at 7.15 6.73 1.34 0.0472 0.9011 XRCC5 X-ray repair complementing defective repair in Chinese hamster cells 5 (double-strand-break rejoining)

204353_s_at 3.71 3.29 1.34 0.0492 0.9011 POT1 protection of telomeres 1

210495_x_at 11.01 10.6 1.33 0.0101 0.9011 FN1 fibronectin 1

207438_s_at 7.06 6.65 1.33 0.0117 0.9011 SNUPN snurportin 1

219418_at 5.36 4.95 1.33 0.0125 0.9011 NHEJ1 nonhomologous end-joining factor 1

221699_s_at 6.16 5.75 1.33 0.0182 0.9011 DDX50 DEAD (Asp-Glu-Ala-Asp) box polypeptide 50

217895_at 5.69 5.28 1.33 0.0259 0.9011 PTCD3 pentatricopeptide repeat domain 3

217878_s_at 5.41 5 1.33 0.0294 0.9011 CDC27 cell division cycle 27

210153_s_at 6.08 5.67 1.33 0.0387 0.9011 ME2 "malic enzyme 2, NAD(+)-dependent, mitochondrial"

217920_at 3.55 3.14 1.33 0.0416 0.9011 MAN1A2 "mannosidase, alpha, class 1A, member 2"

203401_at 5.13 4.72 1.33 0.0435 0.9011 PRPS2 phosphoribosyl pyrophosphate synthetase 2

203259_s_at 7.79 7.39 1.32 0.0041 0.9011 HDDC2 HD domain containing 2

208965_s_at 2.92 2.51 1.32 0.0058 0.9011 IFI16 "interferon, gamma-inducible protein 16"

218102_at 6.76 6.36 1.32 0.0071 0.9011 DERA deoxyribose-phosphate aldolase (putative)

202530_at 6.84 6.44 1.32 0.0125 0.9011 MAPK14 mitogen-activated protein kinase 14

217717_s_at 6.7 6.3 1.32 0.0146 0.9011 YWHAB "tyrosine 3-monooxygenase/tryptophan 5-monooxygenase activation protein, beta"

204391_x_at 3.74 3.34 1.32 0.0172 0.9011 TRIM24 tripartite motif containing 24

211563_s_at 4.11 3.71 1.32 0.0185 0.9011 URI1 "URI1, prefoldin-like chaperone"

219913_s_at 4.41 4.01 1.32 0.019 0.9011 CRNKL1 crooked neck pre-mRNA splicing factor 1

212557_at 3.22 2.82 1.32 0.0194 0.9011 ZNF451 zinc finger protein 451

218595_s_at 5.68 5.29 1.32 0.0235 0.9011 HEATR1 HEAT repeat containing 1

202231_at 7.37 6.97 1.32 0.027 0.9011 EIF3M "eukaryotic translation initiation factor 3, subunit M"

205794_s_at 4.12 3.72 1.32 0.0305 0.9011 NOVA1 neuro-oncological ventral antigen 1

204045_at 5.36 4.96 1.32 0.0336 0.9011 TCEAL1 transcription elongation factor A (SII)-like 1

216640_s_at 7.46 7.07 1.32 0.0422 0.9011 PDIA6 "protein disulfide isomerase family A, member 6"

218005_at 5.84 5.44 1.32 0.0481 0.9011 ZNF22 zinc finger protein 22

204771_s_at 4.62 4.22 1.32 0.049 0.9011 TTF1 "transcription termination factor, RNA polymerase I"

209007_s_at 3.09 2.69 1.32 0.0494 0.9011 RSRP1 arginine/serine-rich protein 1

222157_s_at 4.1 3.71 1.32 0.0498 0.9011 WDR48 WD repeat domain 48

212887_at 6.66 6.27 1.31 0.0156 0.9011 SEC23A "Sec23 homolog A, COPII coat complex component"

208445_s_at 5.2 4.81 1.31 0.0169 0.9011 BAZ1B bromodomain adjacent to zinc finger domain 1B

209513_s_at 6.12 5.73 1.31 0.0171 0.9011 HSDL2 hydroxysteroid dehydrogenase like 2

221787_at 4.25 3.85 1.31 0.0197 0.9011 C6orf120 chromosome 6 open reading frame 120

201575_at 6.46 6.07 1.31 0.0203 0.9011 SNW1 SNW domain containing 1

219133_at 5.42 5.03 1.31 0.0221 0.9011 OXSM "3-oxoacyl-ACP synthase, mitochondrial"

218735_s_at 5.06 4.68 1.31 0.0252 0.9011 ZNF544 zinc finger protein 544

200607_s_at 5.84 5.45 1.31 0.0304 0.9011 RAD21 RAD21 cohesin complex component

219143_s_at 4.97 4.57 1.31 0.0306 0.9011 RPP25 ribonuclease P/MRP 25kDa subunit

204234_s_at 2.96 2.57 1.31 0.0321 0.9011 ZNF195 zinc finger protein 195

204881_s_at 7.08 6.69 1.31 0.0335 0.9011 UGCG UDP-glucose ceramide glucosyltransferase

212166_at 6.53 6.14 1.31 0.0376 0.9011 XPO7 exportin 7

204566_at 2.99 2.6 1.31 0.0401 0.9011 PPM1D "protein phosphatase, Mg2+/Mn2+ dependent, 1D"

201672_s_at 7.83 7.44 1.31 0.0413 0.9011 USP14 ubiquitin specific peptidase 14 (tRNA-guanine transglycosylase)

204769_s_at 4.94 4.55 1.31 0.0471 0.9011 TAP2 "transporter 2, ATP-binding cassette, sub-family B (MDR/TAP)"

221925_s_at 2.45 2.06 1.31 0.0484 0.9011 CSPP1 centrosome and spindle pole associated protein 1

212414_s_at 4.36 3.98 1.3 0.0136 0.9011 GLYR1; SEPT6 glyoxylate reductase 1 homolog (Arabidopsis); septin 6

208658_at 5.34 4.96 1.3 0.0154 0.9011 PDIA4 "protein disulfide isomerase family A, member 4"

218519_at 5.57 5.19 1.3 0.0185 0.9011 SLC35A5 "solute carrier family 35, member A5"

204119_s_at 7.17 6.79 1.3 0.0211 0.9011 ADK adenosine kinase

217970_s_at 4.05 3.67 1.3 0.0289 0.9011 CNOT6 CCR4-NOT transcription complex subunit 6

214577_at 5.83 5.45 1.3 0.0309 0.9011 MAP1B microtubule associated protein 1B

210111_s_at 5.28 4.9 1.3 0.0349 0.9011 KLHDC10 kelch domain containing 10

205930_at 4.25 3.88 1.3 0.0463 0.9011 GTF2E1 general transcription factor IIE subunit 1

200722_s_at 7.57 7.19 1.3 0.047 0.9011 CAPRIN1 cell cycle associated protein 1

212919_at 3.42 3.05 1.29 0.0096 0.9011 DCP2 decapping mRNA 2

202784_s_at 4.89 4.52 1.29 0.012 0.9011 NNT nicotinamide nucleotide transhydrogenase

218096_at 4.62 4.25 1.29 0.0194 0.9011 AGPAT5 1-acylglycerol-3-phosphate O-acyltransferase 5

209974_s_at 7.61 7.25 1.29 0.0235 0.9011 BUB3 BUB3 mitotic checkpoint protein

204640_s_at 6.21 5.84 1.29 0.024 0.9011 SPOP speckle-type POZ protein

202130_at 6.72 6.35 1.29 0.0247 0.9011 RIOK3 RIO kinase 3

205803_s_at 2.51 2.15 1.29 0.0256 0.9011 TRPC1 "transient receptor potential cation channel, subfamily C, member 1"

203291_at 4.54 4.18 1.29 0.0291 0.9011 CNOT4 CCR4-NOT transcription complex subunit 4

201358_s_at 8.62 8.25 1.29 0.0293 0.9011 COPB1 coatomer protein complex subunit beta 1

213452_at 2.73 2.37 1.29 0.0305 0.9011 ZNF184 zinc finger protein 184

209397_at 6.26 5.89 1.29 0.0317 0.9011 ME2 "malic enzyme 2, NAD(+)-dependent, mitochondrial"

218079_s_at 5 4.63 1.29 0.0347 0.9011 GGNBP2 gametogenetin binding protein 2

202321_at 3.69 3.33 1.29 0.0359 0.9011 GGPS1 geranylgeranyl diphosphate synthase 1

204216_s_at 6.79 6.42 1.29 0.0399 0.9011 ZC3H14 zinc finger CCCH-type containing 14

201534_s_at 5.62 5.26 1.29 0.0421 0.9011 UBL3 ubiquitin-like 3

201549_x_at 6.01 5.64 1.29 0.0437 0.9011 KDM5B lysine (K)-specific demethylase 5B

221542_s_at 4.39 4.02 1.29 0.0445 0.9011 ERLIN2 ER lipid raft associated 2

218013_x_at 5.73 5.37 1.29 0.0477 0.9011 DCTN4 dynactin 4 (p62)

202386_s_at 4.74 4.38 1.29 0.0496 0.9011 KIAA0430 KIAA0430

205512_s_at 5.67 5.31 1.28 0.0098 0.9011 AIFM1 "apoptosis-inducing factor, mitochondrion-associated, 1"

218138_at 5.42 5.06 1.28 0.0178 0.9011 MKKS McKusick-Kaufman syndrome

218439_s_at 6.36 6.01 1.28 0.0192 0.9011 COMMD10 COMM domain containing 10

213025_at 3.1 2.74 1.28 0.0207 0.9011 THUMPD1 THUMP domain containing 1

208709_s_at 6.99 6.64 1.28 0.0217 0.9011 NRDC nardilysin convertase

208838_at 4.89 4.54 1.28 0.0224 0.9011 CAND1 cullin-associated and neddylation-dissociated 1

218297_at 5.13 4.77 1.28 0.0257 0.9011 FAM188A "family with sequence similarity 188, member A"

218181_s_at 3.63 3.27 1.28 0.0263 0.9011 MAP4K4 mitogen-activated protein kinase kinase kinase kinase 4

205171_at 4.02 3.66 1.28 0.0286 0.9011 PTPN4 "protein tyrosine phosphatase, non-receptor type 4 (megakaryocyte)"

213218_at 4.6 4.24 1.28 0.0387 0.9011 ZSCAN26 zinc finger and SCAN domain containing 26

218043_s_at 3 2.64 1.28 0.0401 0.9011 AZI2 5-azacytidine induced 2

206695_x_at 2.34 1.98 1.28 0.0406 0.9011 ZNF43 zinc finger protein 43

201023_at 6.97 6.62 1.28 0.0431 0.9011 TAF7 "TAF7 RNA polymerase II, TATA box binding protein (TBP)-associated factor, 55kDa"

212731_at 3.75 3.38 1.28 0.0474 0.9011 ANKRD46 ankyrin repeat domain 46

202532_s_at 3.93 3.58 1.27 0.0069 0.9011 DHFR dihydrofolate reductase

213574_s_at 6.69 6.34 1.27 0.018 0.9011 KPNB1 karyopherin (importin) beta 1

219635_at 4.28 3.93 1.27 0.0186 0.9011 ZNF606 zinc finger protein 606

211084_x_at 4.1 3.75 1.27 0.0213 0.9011 PRKD3 protein kinase D3

203883_s_at 4.4 4.05 1.27 0.0315 0.9011 RAB11FIP2 RAB11 family interacting protein 2 (class I)

202232_s_at 7.76 7.41 1.27 0.0422 0.9011 EIF3M "eukaryotic translation initiation factor 3, subunit M"

219802_at 3.04 2.7 1.27 0.0431 0.9011 PYROXD1 pyridine nucleotide-disulphide oxidoreductase domain 1

202131_s_at 4.14 3.79 1.27 0.0441 0.9011 RIOK3 RIO kinase 3

213019_at 4.18 3.84 1.27 0.0459 0.9011 RANBP6 RAN binding protein 6

212720_at 4.13 3.78 1.27 0.0467 0.9011 PAPOLA poly(A) polymerase alpha

203533_s_at 3.38 3.03 1.27 0.0494 0.9011 CUL5 cullin 5

209786_at 5.86 5.53 1.26 0.0126 0.9011 HMGN4 high mobility group nucleosomal binding domain 4

217812_at 6.06 5.73 1.26 0.015 0.9011 YTHDF2 YTH N(6)-methyladenosine RNA binding protein 2

208875_s_at 3.91 3.57 1.26 0.02 0.9011 PAK2 p21 protein (Cdc42/Rac)-activated kinase 2

205214_at 3.74 3.41 1.26 0.0227 0.9011 STK17B serine/threonine kinase 17b

202664_at 3.9 3.56 1.26 0.0245 0.9011 WIPF1 "WAS/WASL interacting protein family, member 1"

220141_at 3.08 2.74 1.26 0.0321 0.9011 C11orf63 chromosome 11 open reading frame 63

212692_s_at 3.45 3.11 1.26 0.035 0.9011 LRBA "LPS-responsive vesicle trafficking, beach and anchor containing"

203629_s_at 3.7 3.36 1.26 0.0353 0.9011 COG5 component of oligomeric golgi complex 5

201290_at 9.03 8.7 1.26 0.0357 0.9011 SEC11A "SEC11 homolog A, signal peptidase complex subunit"

206958_s_at 3.6 3.27 1.26 0.0377 0.9011 UPF3A UPF3 regulator of nonsense transcripts homolog A (yeast)

203710_at 2.99 2.66 1.26 0.0395 0.9011 ITPR1 "inositol 1,4,5-trisphosphate receptor, type 1"

202395_at 6.29 5.96 1.26 0.0401 0.9011 NSF N-ethylmaleimide-sensitive factor

210817_s_at 6.79 6.46 1.26 0.0413 0.9011 CALCOCO2 calcium binding and coiled-coil domain 2

221816_s_at 6.88 6.55 1.26 0.0496 0.9011 PHF11 PHD finger protein 11

213220_at 6.07 5.73 1.26 0.0499 0.9011 BBIP1 BBSome interacting protein 1

212534_at 5.02 4.7 1.25 0.0156 0.9011 ZNF24 zinc finger protein 24

212464_s_at 10.77 10.45 1.25 0.0171 0.9011 FN1 fibronectin 1

212554_at 5.28 4.96 1.25 0.0215 0.9011 CAP2 "CAP, adenylate cyclase-associated protein, 2 (yeast)"

218254_s_at 6.99 6.67 1.25 0.0249 0.9011 SAR1B "secretion associated, Ras related GTPase 1B"

212474_at 3.27 2.94 1.25 0.0257 0.9011 AVL9 AVL9 homolog (S. cerevisiase)

201485_s_at 6.33 6.01 1.25 0.0284 0.9011 RCN2 "reticulocalbin 2, EF-hand calcium binding domain"

209366_x_at 6.78 6.46 1.25 0.0332 0.9011 CYB5A cytochrome b5 type A (microsomal)

213117_at 4.96 4.63 1.25 0.0339 0.9011 KLHL9 kelch-like family member 9

204759_at 3.44 3.12 1.25 0.0342 0.9011 RCBTB2 regulator of chromosome condensation (RCC1) and BTB (POZ) domain containing protein 2

202786_at 4.6 4.28 1.25 0.0351 0.9011 STK39 serine threonine kinase 39

212036_s_at 3.86 3.54 1.25 0.0365 0.9011 PNN "pinin, desmosome associated protein"

201177_s_at 6.82 6.5 1.25 0.0402 0.9011 UBA2 ubiquitin-like modifier activating enzyme 2

202863_at 5.22 4.9 1.25 0.0464 0.9011 SP100 SP100 nuclear antigen

205761_s_at 2.96 2.64 1.25 0.0496 0.9011 DUS4L dihydrouridine synthase 4-like

212107_s_at 4.35 4.03 1.24 0.018 0.9011 DHX9 DEAH (Asp-Glu-Ala-His) box helicase 9

200828_s_at 8.47 8.16 1.24 0.0185 0.9011 ZNF207 zinc finger protein 207

203620_s_at 4.58 4.27 1.24 0.0189 0.9011 FCHSD2 FCH and double SH3 domains 2

209155_s_at 5.1 4.79 1.24 0.0229 0.9011 NT5C2 "5'-nucleotidase, cytosolic II"

216515_x_at 8.6 8.29 1.24 0.0235 0.9011 MIR1244-1; MIR1244-2; MIR1244-3; MIR1244-4 microRNA 1244-1; microRNA 1244-2; microRNA 1244-3; microRNA 1244-4

212241_at 3.71 3.4 1.24 0.0269 0.9011 GCOM1; POLR2M GRINL1A complex locus 1; polymerase (RNA) II (DNA directed) polypeptide M

212822_at 5.52 5.21 1.24 0.0359 0.9011 HEG1 heart development protein with EGF-like domains 1

214773_x_at 4.79 4.48 1.24 0.0367 0.9011 TIPRL TOR signaling pathway regulator

201513_at 3.17 2.86 1.24 0.0394 0.9011 TSN translin

200640_at 9.09 8.78 1.24 0.0451 0.9011 YWHAZ "tyrosine 3-monooxygenase/tryptophan 5-monooxygenase activation protein, zeta"

207168_s_at 8.54 8.23 1.24 0.0485 0.9011 H2AFY "H2A histone family, member Y"

212840_at 3.71 3.41 1.23 0.0128 0.9011 UBXN7 UBX domain protein 7

222239_s_at 2.16 1.86 1.23 0.017 0.9011 INTS6 integrator complex subunit 6

201305_x_at 8.27 7.98 1.23 0.0228 0.9011 ANP32B acidic nuclear phosphoprotein 32 family member B

205573_s_at 7.4 7.09 1.23 0.0253 0.9011 SNX7 sorting nexin 7

209005_at 5.6 5.3 1.23 0.0269 0.9011 FBXL5 F-box and leucine-rich repeat protein 5

218968_s_at 6.65 6.36 1.23 0.0286 0.9011 ZFP64 ZFP64 zinc finger protein

220952_s_at 2.52 2.23 1.23 0.0373 0.9011 PLEKHA5 "pleckstrin homology domain containing, family A member 5"

219356_s_at 7.19 6.89 1.23 0.0375 0.9011 CHMP5 charged multivesicular body protein 5

213689_x_at 4.09 3.79 1.23 0.0395 0.9011 FAM69A "family with sequence similarity 69, member A"

213694_at 2.71 2.4 1.23 0.0457 0.9011 RSBN1 round spermatid basic protein 1

217933_s_at 5.92 5.62 1.23 0.0484 0.9011 LAP3 leucine aminopeptidase 3

202512_s_at 3.65 3.35 1.23 0.0498 0.9011 ATG5 autophagy related 5

210639_s_at 4.39 4.1 1.22 0.0098 0.9011 ATG5 autophagy related 5

219902_at 4.04 3.76 1.22 0.025 0.9011 BHMT2 betaine--homocysteine S-methyltransferase 2

222150_s_at 2.35 2.06 1.22 0.0295 0.9011 GSAP gamma-secretase activating protein

219311_at 3.35 3.06 1.22 0.0295 0.9011 CEP76 centrosomal protein 76kDa

207614_s_at 5.35 5.06 1.22 0.0361 0.9011 CUL1 cullin 1

205308_at 4.8 4.51 1.22 0.0378 0.9011 ZC2HC1A "zinc finger, C2HC-type containing 1A"

219641_at 6.12 5.83 1.22 0.0382 0.9011 DET1 de-etiolated homolog 1 (Arabidopsis)

221573_at 4.86 4.58 1.22 0.0384 0.9011 C7orf25 chromosome 7 open reading frame 25

203204_s_at 4.99 4.7 1.22 0.0388 0.9011 KDM4A lysine (K)-specific demethylase 4A

218264_at 4.49 4.21 1.22 0.0406 0.9011 BCCIP BRCA2 and CDKN1A interacting protein

206554_x_at 3.18 2.89 1.22 0.0423 0.9011 SETMAR SET domain and mariner transposase fusion gene

200838_at 7.86 7.57 1.22 0.0436 0.9011 CTSB cathepsin B

212629_s_at 3.71 3.42 1.22 0.0467 0.9011 PKN2 protein kinase N2

201594_s_at 6.75 6.46 1.22 0.0476 0.9011 PPP4R1 "protein phosphatase 4, regulatory subunit 1"

221941_at 5.49 5.2 1.22 0.0379 0.9011 PAOX polyamine oxidase (exo-N4-amino)

209272_at 4.52 4.24 1.21 0.0146 0.9011 NAB1 NGFI-A binding protein 1

219378_at 2.57 2.3 1.21 0.016 0.9011 NAA16 "N(alpha)-acetyltransferase 16, NatA auxiliary subunit"

201501_s_at 5.3 5.02 1.21 0.0202 0.9011 GRSF1 G-rich RNA sequence binding factor 1

210568_s_at 3.3 3.03 1.21 0.0216 0.9011 RECQL RecQ helicase-like

213652_at 2.34 2.06 1.21 0.0255 0.9011 PCSK5 proprotein convertase subtilisin/kexin type 5

214499_s_at 3.32 3.05 1.21 0.0261 0.9011 BCLAF1 BCL2-associated transcription factor 1

208974_x_at 7.12 6.84 1.21 0.0285 0.9011 KPNB1 karyopherin (importin) beta 1

220099_s_at 4.6 4.32 1.21 0.0319 0.9011 C7orf55-LUC7L2; LUC7L2 C7orf55-LUC7L2 readthrough; LUC7-like 2 pre-mRNA splicing factor

202970_at 3.43 3.16 1.21 0.0342 0.9011 DYRK2 dual specificity tyrosine-(Y)-phosphorylation regulated kinase 2

202728_s_at 4.9 4.62 1.21 0.0376 0.9011 LTBP1 latent transforming growth factor beta binding protein 1

213376_at 4.49 4.22 1.21 0.0405 0.9011 ZBTB1 zinc finger and BTB domain containing 1

208803_s_at 5.86 5.59 1.21 0.045 0.9011 SRP72 signal recognition particle 72kDa

204001_at 3.49 3.21 1.21 0.0492 0.9011 SNAPC3 small nuclear RNA activating complex polypeptide 3

204112_s_at 2.49 2.23 1.2 0.0247 0.9011 HNMT histamine N-methyltransferase

212997_s_at 4.07 3.8 1.2 0.0277 0.9011 TLK2 tousled-like kinase 2

219590_x_at 7.23 6.97 1.2 0.0322 0.9011 DPH5 diphthamide biosynthesis 5

213238_at 4.25 4 1.2 0.0332 0.9011 ATP10D "ATPase, class V, type 10D"

207056_s_at 2.61 2.35 1.2 0.0372 0.9011 SLC4A8 "solute carrier family 4, sodium bicarbonate cotransporter, member 8"

201434_at 6.63 6.37 1.2 0.0382 0.9011 TTC1 tetratricopeptide repeat domain 1

204093_at 6.1 5.84 1.2 0.0477 0.9011 CCNH cyclin H

211354_s_at 3.22 2.96 1.2 0.0485 0.9011 LEPR leptin receptor

218719_s_at 4.8 4.54 1.2 0.0489 0.9011 GINS3 GINS complex subunit 3 (Psf3 homolog)

206015_s_at 5.87 5.61 1.2 0.0492 0.9011 FOXJ3 forkhead box J3

211732_x_at 2.99 2.74 1.19 0.0157 0.9011 HNMT histamine N-methyltransferase

201499_s_at 6.38 6.13 1.19 0.0207 0.9011 USP7 ubiquitin specific peptidase 7 (herpes virus-associated)

219204_s_at 4.28 4.03 1.19 0.0242 0.9011 SRR serine racemase

221483_s_at 6.98 6.72 1.19 0.0272 0.9011 ARPP19 cAMP-regulated phosphoprotein 19kDa

201927_s_at 3.46 3.21 1.19 0.0329 0.9011 PKP4 plakophilin 4

213233_s_at 7.1 6.85 1.19 0.035 0.9011 KLHL9 kelch-like family member 9

200705_s_at 10.85 10.6 1.19 0.0387 0.9011 EEF1B2 eukaryotic translation elongation factor 1 beta 2

212603_at 4.3 4.05 1.19 0.0393 0.9011 MRPS31 mitochondrial ribosomal protein S31

204510_at 3 2.75 1.19 0.0404 0.9011 CDC7 cell division cycle 7

213341_at 3.63 3.39 1.19 0.0451 0.9011 FEM1C fem-1 homolog c (C. elegans)

203829_at 4.45 4.2 1.19 0.049 0.9011 ELP4 elongator acetyltransferase complex subunit 4

203654_s_at 4.3 4.05 1.19 0.0495 0.9011 COIL coilin

218250_s_at 7.13 6.89 1.18 0.023 0.9011 CNOT7 CCR4-NOT transcription complex subunit 7

215227_x_at 6.19 5.95 1.18 0.0256 0.9011 ACP1 "acid phosphatase 1, soluble"

219347_at 2.8 2.56 1.18 0.0384 0.9011 NUDT15 nudix hydrolase 15

209091_s_at 8.26 8.02 1.18 0.0385 0.9011 SH3GLB1 SH3-domain GRB2-like endophilin B1

219221_at 5.36 5.12 1.18 0.0392 0.9011 ZBTB38 zinc finger and BTB domain containing 38

205871_at 2.35 2.11 1.18 0.0392 0.9011 PLGLB1; PLGLB2 plasminogen-like B1; plasminogen-like B2

209907_s_at 3.51 3.27 1.18 0.0419 0.9011 ITSN2 intersectin 2

221234_s_at 2.4 2.16 1.18 0.0474 0.9011 BACH2 "BTB and CNC homology 1, basic leucine zipper transcription factor 2"

209943_at 3.05 2.82 1.18 0.0496 0.9011 FBXL4 F-box and leucine-rich repeat protein 4

216442_x_at 10.83 10.6 1.17 0.0205 0.9011 FN1 fibronectin 1

208260_at 5.78 5.55 1.17 0.0264 0.9011 AVPR1B arginine vasopressin receptor 1B

218819_at 2.93 2.71 1.17 0.0265 0.9011 INTS6 integrator complex subunit 6

206085_s_at 3.63 3.4 1.17 0.0461 0.9011 CTH cystathionine gamma-lyase

200090_at 7.23 7.01 1.16 0.0338 0.9011 FNTA "farnesyltransferase, CAAX box, alpha"

210839_s_at 3.48 3.27 1.16 0.0392 0.9011 ENPP2 ectonucleotide pyrophosphatase/phosphodiesterase 2

221046_s_at 4.85 4.63 1.16 0.0416 0.9011 GTPBP8 GTP-binding protein 8 (putative)

212612_at 3.3 3.09 1.16 0.0458 0.9011 RCOR1 REST corepressor 1

212264_s_at 4.21 4 1.16 0.0458 0.9011 WAPL WAPL cohesin release factor

212199_at 5.04 4.83 1.16 0.0461 0.9011 MRFAP1L1 Morf4 family associated protein 1-like 1

204604_at 4.36 4.16 1.15 0.0349 0.9011 CDK14 cyclin-dependent kinase 14

202179_at 5.88 5.69 1.14 0.0416 0.9011 BLMH bleomycin hydrolase

211202_s_at 6.49 6.31 1.13 0.0365 0.9011 KDM5B lysine (K)-specific demethylase 5B

214585_s_at 8.26 8.42 -1.12 0.045 0.9011 VPS52 vacuolar protein sorting 52 homolog (S. cerevisiae)

208457_at 4.51 4.68 -1.12 0.049 0.9011 GABRD "gamma-aminobutyric acid (GABA) A receptor, delta"

220807_at 3.4 3.58 -1.13 0.0382 0.9011 HBQ1 "hemoglobin, theta 1"

205910_s_at 2.45 2.62 -1.13 0.0473 0.9011 CEL carboxyl ester lipase

218318_s_at 2.28 2.47 -1.14 0.033 0.9011 NLK nemo-like kinase

221887_s_at 5.22 5.4 -1.14 0.0332 0.9011 DFNB31 "deafness, autosomal recessive 31"

215121_x_at 3.51 3.7 -1.14 0.0343 0.9011 CYAT1; IGLC1; IGLC2; IGLC3; IGLC7; IGLJ3; IGLL5; IGLV1-44; IGLV3-25; IGLV4-3 immunoglobulin lambda light chain-like; immunoglobulin lambda constant 1 (Mcg marker); immunoglobulin lambda constant 2 (Kern-Oz- marker); immunoglobulin lambda constant 3 (Kern-Oz+ marker); immunoglobulin lambda constant 7; immunoglobulin lambda joining 3; immunoglobulin lambda-like polypeptide 5; immunoglobulin lambda variable 1-44; immunoglobulin lambda variable 3-25; immunoglobulin lambda variable 4-3

220479_at 3.09 3.28 -1.14 0.039 0.9011 CPS1-IT1 CPS1 intronic transcript 1

218144_s_at 6.9 7.08 -1.14 0.0413 0.9011 INF2 "inverted formin, FH2 and WH2 domain containing"

205681_at 2.5 2.69 -1.14 0.0427 0.9011 BCL2A1 BCL2-related protein A1

207102_at 1.81 2 -1.14 0.0472 0.9011 AKR1D1 "aldo-keto reductase family 1, member D1"

201207_at 5.98 6.19 -1.15 0.033 0.9011 TNFAIP1 "tumor necrosis factor, alpha-induced protein 1 (endothelial)"

201373_at 7.85 8.05 -1.15 0.0433 0.9011 PLEC plectin

35148_at 3.16 3.37 -1.16 0.0363 0.9011 TJP3 tight junction protein 3

207095_at 2.66 2.88 -1.16 0.0376 0.9011 SLC10A2 "solute carrier family 10 (sodium/bile acid cotransporter), member 2"

209650_s_at 6.76 6.98 -1.16 0.039 0.9011 TBC1D22A "TBC1 domain family, member 22A"

207625_s_at 5.07 5.29 -1.16 0.0469 0.9011 CBFA2T2 "core-binding factor, runt domain, alpha subunit 2; translocated to, 2"

215593_at 4.42 4.63 -1.16 0.0491 0.9011

207733_x_at 3.47 3.7 -1.17 0.0194 0.9011 PSG9 pregnancy specific beta-1-glycoprotein 9

219591_at 5.62 5.85 -1.17 0.0204 0.9011 CEND1 cell cycle exit and neuronal differentiation 1

211819_s_at 3.1 3.32 -1.17 0.0212 0.9011 SORBS1 sorbin and SH3 domain containing 1

211230_s_at 5.44 5.66 -1.17 0.0256 0.9011 PIK3CD "phosphatidylinositol-4,5-bisphosphate 3-kinase, catalytic subunit delta"

205622_at 6.06 6.29 -1.17 0.0305 0.9011 SMPD2 "sphingomyelin phosphodiesterase 2, neutral membrane (neutral sphingomyelinase)"

205075_at 4.88 5.1 -1.17 0.0316 0.9011 SERPINF2 "serpin peptidase inhibitor, clade F (alpha-2 antiplasmin, pigment epithelium derived factor), member 2"

214676_x_at 5.08 5.31 -1.17 0.0333 0.9011 MUC3B "mucin 3B, cell surface associated"

209051_s_at 4.75 4.97 -1.17 0.0342 0.9011 RALGDS ral guanine nucleotide dissociation stimulator

210245_at 3.9 4.13 -1.17 0.0357 0.9011 ABCC8 ATP binding cassette subfamily C member 8

221828_s_at 4.12 4.35 -1.17 0.0359 0.9011 MVB12B multivesicular body subunit 12B

220288_at 2.76 2.99 -1.17 0.0426 0.9011 MYO15A myosin XVA

214234_s_at 2.61 2.84 -1.17 0.0449 0.9011 CYP3A5 "cytochrome P450, family 3, subfamily A, polypeptide 5"

34406_at 5.97 6.19 -1.17 0.0463 0.9011 PACS2 phosphofurin acidic cluster sorting protein 2

209217_s_at 7.01 7.23 -1.17 0.0467 0.9011 WDR45 WD repeat domain 45

220608_s_at 2.19 2.42 -1.17 0.0475 0.9011

208751_at 7.49 7.72 -1.17 0.0485 0.9011 NAPA "N-ethylmaleimide-sensitive factor attachment protein, alpha"

213499_at 3.95 4.19 -1.18 0.0274 0.9011 CLCN2 "chloride channel, voltage-sensitive 2"

214275_at 3.88 4.12 -1.18 0.0276 0.9011 MED12 mediator complex subunit 12

216495_x_at 5.78 6.02 -1.18 0.0328 0.9011

214236_at 2.41 2.65 -1.18 0.0374 0.9011

217020_at 3.16 3.4 -1.18 0.0405 0.9011 RARB "retinoic acid receptor, beta"

206872_at 3.42 3.66 -1.18 0.0412 0.9011 SLC17A1 "solute carrier family 17 (organic anion transporter), member 1"

221879_at 2.68 2.92 -1.18 0.0414 0.9011 CALML4 calmodulin-like 4

203308_x_at 6.84 7.08 -1.18 0.0423 0.9011 HPS1 Hermansky-Pudlak syndrome 1

211258_s_at 4.38 4.62 -1.18 0.0467 0.9011 TGFA transforming growth factor alpha

215156_at 3.8 4.03 -1.18 0.0472 0.9011 WDR61 WD repeat domain 61

214154_s_at 3.83 4.06 -1.18 0.0488 0.9011 PKP2 plakophilin 2

220665_at 3.93 4.17 -1.18 0.0489 0.9011 LUZP4 leucine zipper protein 4

204384_at 3.98 4.23 -1.19 0.0308 0.9011 GOLGA2 golgin A2

206358_at 2.62 2.87 -1.19 0.034 0.9011 PRM1 protamine 1

203495_at 3.77 4.02 -1.19 0.042 0.9011 LRRC14 leucine rich repeat containing 14

201140_s_at 6.87 7.11 -1.19 0.0434 0.9011 RAB5C "RAB5C, member RAS oncogene family"

31837_at 8.09 8.34 -1.19 0.045 0.9011 LMF2 lipase maturation factor 2

217780_at 8.8 9.05 -1.19 0.0483 0.9011 WDR83OS WD repeat domain 83 opposite strand

216654_at 3.22 3.48 -1.19 0.0492 0.9011 TNXB tenascin XB

203524_s_at 7.4 7.66 -1.2 0.0259 0.9011 MPST mercaptopyruvate sulfurtransferase

206311_s_at 2.15 2.42 -1.2 0.0296 0.9011 PLA2G1B "phospholipase A2, group IB (pancreas)"

216365_x_at 3.39 3.66 -1.2 0.0323 0.9011 IGLJ3; IGLL5; IGLV3-19 immunoglobulin lambda joining 3; immunoglobulin lambda-like polypeptide 5; immunoglobulin lambda variable 3-19

202472_at 5.51 5.77 -1.2 0.0363 0.9011 MPI mannose phosphate isomerase

214219_x_at 3.77 4.03 -1.2 0.0366 0.9011 MAP4K1 mitogen-activated protein kinase kinase kinase kinase 1

208978_at 7.72 7.98 -1.2 0.0377 0.9011 CRIP2 cysteine-rich protein 2

206065_s_at 2.73 3 -1.2 0.038 0.9011 DPYS dihydropyrimidinase

203136_at 8.91 9.18 -1.2 0.0383 0.9011 RABAC1 Rab acceptor 1 (prenylated)

219415_at 2.93 3.2 -1.2 0.0446 0.9011 TTYH1 tweety family member 1

214287_s_at 2.47 2.73 -1.2 0.0449 0.9011 CDK13 cyclin-dependent kinase 13

218913_s_at 4.56 4.82 -1.2 0.0457 0.9011 GMIP GEM interacting protein

206580_s_at 8.56 8.82 -1.2 0.0464 0.9011 EFEMP2 EGF containing fibulin-like extracellular matrix protein 2

208222_at 4.29 4.57 -1.21 0.0214 0.9011 ACVR1B activin A receptor type IB

221996_s_at 3.37 3.64 -1.21 0.0234 0.9011 CLTB "clathrin, light chain B"

215481_s_at 3.66 3.93 -1.21 0.0238 0.9011 PEX5 peroxisomal biogenesis factor 5

221316_at 2.72 3 -1.21 0.028 0.9011 CATSPERG catsper channel auxiliary subunit gamma

208858_s_at 8.04 8.32 -1.21 0.0321 0.9011 ESYT1 extended synaptotagmin-like protein 1

53720_at 5.86 6.14 -1.21 0.033 0.9011 C19orf66 chromosome 19 open reading frame 66

52078_at 7.71 7.98 -1.21 0.0339 0.9011 TMEM222 transmembrane protein 222

210475_at 4.19 4.46 -1.21 0.0349 0.9011 POU3F1 POU class 3 homeobox 1

203461_at 2.74 3.02 -1.21 0.0364 0.9011 CHD2 chromodomain helicase DNA binding protein 2

212715_s_at 3.61 3.88 -1.21 0.039 0.9011 MICAL3 "microtubule associated monooxygenase, calponin and LIM domain containing 3"

219403_s_at 2.98 3.25 -1.21 0.0407 0.9011 HPSE heparanase

219783_at 5.22 5.5 -1.21 0.0427 0.9011 SLC35F6 "solute carrier family 35, member F6"

207369_at 3.45 3.72 -1.21 0.0432 0.9011 BRS3 bombesin-like receptor 3

210577_at 4.32 4.59 -1.21 0.0444 0.9011 CASR calcium-sensing receptor

203421_at 5.73 6.01 -1.21 0.0474 0.9011 TP53I11 tumor protein p53 inducible protein 11

220101_x_at 4.16 4.43 -1.21 0.0486 0.9011

217023_x_at 3.91 4.19 -1.21 0.049 0.9011 TPSAB1; TPSB2 tryptase alpha/beta 1; tryptase beta 2 (gene/pseudogene)

221309_at 4.12 4.41 -1.22 0.0115 0.9011 RBM17 RNA binding motif protein 17

215891_s_at 4.08 4.37 -1.22 0.0185 0.9011 GM2A GM2 ganglioside activator

204187_at 3.64 3.93 -1.22 0.0238 0.9011 GMPR guanosine monophosphate reductase

207634_at 4.8 5.09 -1.22 0.0238 0.9011 PDCD1 programmed cell death 1

215817_at 2.99 3.28 -1.22 0.0274 0.9011 SERPINB13 "serpin peptidase inhibitor, clade B (ovalbumin), member 13"

207473_at 4.19 4.48 -1.22 0.0319 0.9011 MLN motilin

209930_s_at 2.67 2.96 -1.22 0.033 0.9011 NFE2 "nuclear factor, erythroid 2"

218010_x_at 6.43 6.72 -1.22 0.034 0.9011 PPDPF pancreatic progenitor cell differentiation and proliferation factor

206054_at 2.95 3.24 -1.22 0.0351 0.9011 KNG1 kininogen 1

217134_at 3.16 3.44 -1.22 0.0361 0.9011 MTAP methylthioadenosine phosphorylase

209802_at 3.59 3.88 -1.22 0.0392 0.9011 PHLDA2 "pleckstrin homology-like domain, family A, member 2"

205576_at 2.69 2.97 -1.22 0.041 0.9011 SERPIND1 "serpin peptidase inhibitor, clade D (heparin cofactor), member 1"

220031_at 3.49 3.78 -1.22 0.0447 0.9011 OTUD7B OTU deubiquitinase 7B

210234_at 4.59 4.89 -1.23 0.0097 0.9011 GRM4 "glutamate receptor, metabotropic 4"

207126_x_at 4.8 5.1 -1.23 0.0139 0.9011 UGT1A1; UGT1A10; UGT1A4; UGT1A6; UGT1A8; UGT1A9 "UDP glucuronosyltransferase 1 family, polypeptide A1; UDP glucuronosyltransferase 1 family, polypeptide A10; UDP glucuronosyltransferase 1 family, polypeptide A4; UDP glucuronosyltransferase 1 family, polypeptide A6; UDP glucuronosyltransferase 1 family, polypeptide A8; UDP glucuronosyltransferase 1 family, polypeptide A9"

201641_at 3.47 3.76 -1.23 0.015 0.9011 BST2 bone marrow stromal cell antigen 2

215502_at 4.28 4.58 -1.23 0.0201 0.9011

201895_at 7.41 7.71 -1.23 0.0206 0.9011 ARAF "A-Raf proto-oncogene, serine/threonine kinase"

222221_x_at 7.39 7.68 -1.23 0.0217 0.9011 EHD1 EH domain containing 1

218143_s_at 7.33 7.63 -1.23 0.0223 0.9011 SCAMP2 secretory carrier membrane protein 2

219860_at 3.24 3.53 -1.23 0.0258 0.9011 LY6G5C "lymphocyte antigen 6 complex, locus G5C"

210010_s_at 7.16 7.46 -1.23 0.0277 0.9011 SLC25A1 "solute carrier family 25 (mitochondrial carrier; citrate transporter), member 1"

209038_s_at 5.74 6.04 -1.23 0.0299 0.9011 EHD1 EH domain containing 1

211677_x_at 4.88 5.18 -1.23 0.0316 0.9011 CADM3 cell adhesion molecule 3

207258_at 4.43 4.72 -1.23 0.0319 0.9011 DSCR4 Down syndrome critical region 4

216646_at 4.67 4.96 -1.23 0.0335 0.9011 DSCC1 DNA replication and sister chromatid cohesion 1

209420_s_at 6.64 6.94 -1.23 0.0389 0.9011 SMPD1 "sphingomyelin phosphodiesterase 1, acid lysosomal"

207960_at 3.76 4.06 -1.23 0.0416 0.9011

220482_s_at 5.09 5.38 -1.23 0.0418 0.9011 SERGEF secretion regulating guanine nucleotide exchange factor

205983_at 4.47 4.78 -1.23 0.0421 0.9011 DPEP1 dipeptidase 1 (renal)

206566_at 3.19 3.5 -1.23 0.0441 0.9011 SLC7A1 "solute carrier family 7 (cationic amino acid transporter, y+ system), member 1"

201189_s_at 7.3 7.59 -1.23 0.047 0.9011 ITPR3 "inositol 1,4,5-trisphosphate receptor, type 3"

208479_at 3.76 4.06 -1.23 0.049 0.9011 KCNA1 "potassium channel, voltage gated shaker related subfamily A, member 1"

221444_at 2.67 2.99 -1.24 0.0221 0.9011 TAS2R16 "taste receptor, type 2, member 16"

207005_s_at 4.87 5.18 -1.24 0.0248 0.9011 BCL2 B-cell CLL/lymphoma 2

205549_at 3.8 4.11 -1.24 0.0296 0.9011 PCP4 Purkinje cell protein 4

217017_at 3.88 4.19 -1.24 0.0318 0.9011 OSBPL10 oxysterol binding protein-like 10

220654_at 3.45 3.76 -1.24 0.0326 0.9011 PPY2P "pancreatic polypeptide 2, pseudogene"

210846_x_at 3.07 3.37 -1.24 0.0374 0.9011 TRIM14 tripartite motif containing 14

50221_at 5.09 5.4 -1.24 0.0417 0.9011 TFEB transcription factor EB

214242_at 2.58 2.89 -1.24 0.0483 0.9011 MAN1A2 "mannosidase, alpha, class 1A, member 2"

214237_x_at 5.11 5.43 -1.25 0.0123 0.9011 PAWR "PRKC, apoptosis, WT1, regulator"

210443_x_at 6.21 6.52 -1.25 0.0183 0.9011 OGFR opioid growth factor receptor

215487_x_at 3.26 3.58 -1.25 0.0188 0.9011 FAM182B "family with sequence similarity 182, member B"

210499_s_at 4.62 4.94 -1.25 0.0203 0.9011 PQBP1 polyglutamine binding protein 1

215278_at 3 3.31 -1.25 0.0229 0.9011

211020_at 2.69 3.01 -1.25 0.037 0.9011 GCNT2 "glucosaminyl (N-acetyl) transferase 2, I-branching enzyme (I blood group)"

209712_at 3.39 3.71 -1.25 0.0406 0.9011 SLC35D1 "solute carrier family 35 (UDP-GlcA/UDP-GalNAc transporter), member D1"

204144_s_at 5.02 5.34 -1.25 0.043 0.9011 PIGQ phosphatidylinositol glycan anchor biosynthesis class Q

221110_x_at 2.92 3.24 -1.25 0.0438 0.9011 PDE11A phosphodiesterase 11A

220052_s_at 6.08 6.4 -1.25 0.0448 0.9011 TINF2 TERF1 (TRF1)-interacting nuclear factor 2

205254_x_at 3.58 3.9 -1.25 0.0465 0.9011 TCF7 "transcription factor 7 (T-cell specific, HMG-box)"

220557_s_at 4.24 4.56 -1.25 0.0498 0.9011 PACS1 phosphofurin acidic cluster sorting protein 1

211499_s_at 5.23 5.57 -1.26 0.0173 0.9011 MAPK11 mitogen-activated protein kinase 11

220189_s_at 6.56 6.89 -1.26 0.0184 0.9011 MGAT4B "mannosyl (alpha-1,3-)-glycoprotein beta-1,4-N-acetylglucosaminyltransferase, isozyme B"

215106_at 3.78 4.11 -1.26 0.0208 0.9011 TTC22 tetratricopeptide repeat domain 22

211395_x_at 4.41 4.74 -1.26 0.0209 0.9011 FCGR2C "Fc fragment of IgG, low affinity IIc, receptor for (CD32) (gene/pseudogene)"

210129_s_at 4.5 4.84 -1.26 0.0238 0.9011 ARPC4-TTLL3; TTLL3 ARPC4-TTLL3 readthrough; tubulin tyrosine ligase-like family member 3

200800_s_at 6.55 6.88 -1.26 0.0259 0.9011 HSPA1A; HSPA1B heat shock 70kDa protein 1A; heat shock 70kDa protein 1B

219835_at 4.29 4.62 -1.26 0.0267 0.9011 PRDM8 PR domain containing 8

207726_at 3.39 3.72 -1.26 0.0301 0.9011 ESRRB estrogen-related receptor beta

214763_at 5.34 5.67 -1.26 0.0308 0.9011 ACOT11 acyl-CoA thioesterase 11

219719_at 4.39 4.72 -1.26 0.0317 0.9011 HIGD1B "HIG1 hypoxia inducible domain family, member 1B"

209695_at 5.3 5.63 -1.26 0.0329 0.9011 PTP4A3 "protein tyrosine phosphatase type IVA, member 3"

208582_s_at 2.74 3.06 -1.26 0.0331 0.9011 DUX1; DUX3; DUX5 double homeobox 1; double homeobox 3; double homeobox 5

220875_at 3.21 3.55 -1.26 0.0346 0.9011

221313_at 3.87 4.21 -1.26 0.0354 0.9011 GPR52 G protein-coupled receptor 52

205968_at 4.91 5.24 -1.26 0.0398 0.9011 KCNS3 "potassium voltage-gated channel, modifier subfamily S, member 3"

203025_at 7.39 7.72 -1.26 0.0424 0.9011 NAA10 "N(alpha)-acetyltransferase 10, NatA catalytic subunit"

209499_x_at 3.95 4.27 -1.26 0.045 0.9011 TNFSF12-TNFSF13; TNFSF13 "TNFSF12-TNFSF13 readthrough; tumor necrosis factor (ligand) superfamily, member 13"

202340_x_at 4.56 4.9 -1.27 0.0158 0.9011 NR4A1 "nuclear receptor subfamily 4, group A, member 1"

203144_s_at 3.26 3.61 -1.27 0.021 0.9011 KIAA0040 KIAA0040

213200_at 3.93 4.28 -1.27 0.0214 0.9011 SYP synaptophysin

216470_x_at 3.82 4.16 -1.27 0.0228 0.9011 PRSS3P1 "protease, serine, 3 pseudogene 1"

217729_s_at 6.85 7.2 -1.27 0.0247 0.9011 AES amino-terminal enhancer of split

206819_at 2.82 3.17 -1.27 0.0252 0.9011 POM121L9P "POM121 transmembrane nucleoporin-like 9, pseudogene"

204360_s_at 6.26 6.61 -1.27 0.0362 0.9011 NAGLU "N-acetylglucosaminidase, alpha"

221360_s_at 3.68 4.02 -1.27 0.0465 0.9011 GHSR growth hormone secretagogue receptor

206813_at 6.17 6.53 -1.28 0.0121 0.9011 CTF1 cardiotrophin 1

215603_x_at 4.48 4.84 -1.28 0.0192 0.9011 GGT1; GGT2; GGT3P; GGTLC1; GGTLC2; GGTLC3; LOC102724197; LOC102724823 gamma-glutamyltransferase 1; gamma-glutamyltransferase 2; gamma-glutamyltransferase 3 pseudogene; gamma-glutamyltransferase light chain 1; gamma-glutamyltransferase light chain 2; gamma-glutamyltransferase light chain 3; inactive gamma-glutamyltranspeptidase 2-like; inactive gamma-glutamyltranspeptidase 2

204398_s_at 4.94 5.3 -1.28 0.0202 0.9011 EML2 echinoderm microtubule associated protein like 2

209759_s_at 7.69 8.05 -1.28 0.023 0.9011 ECI1 enoyl-CoA delta isomerase 1

207873_x_at 3.92 4.28 -1.28 0.0303 0.9011 SEZ6L seizure related 6 homolog (mouse)-like

202895_s_at 4.83 5.18 -1.28 0.0319 0.9011 SIRPA signal-regulatory protein alpha

212843_at 4.4 4.75 -1.28 0.0378 0.9011 NCAM1 neural cell adhesion molecule 1

209597_s_at 3.03 3.39 -1.28 0.0383 0.9011 PNMA2 paraneoplastic Ma antigen 2

210083_at 4.45 4.81 -1.28 0.0385 0.9011 SEMA7A "semaphorin 7A, GPI membrane anchor (John Milton Hagen blood group)"

204158_s_at 7.98 8.34 -1.28 0.0438 0.9011 TCIRG1 "T-cell, immune regulator 1, ATPase, H+ transporting, lysosomal V0 subunit A3"

205145_s_at 6.4 6.75 -1.28 0.0464 0.9011 MYL5 myosin light chain 5

222144_at 3.65 4.01 -1.28 0.0483 0.9011 KIF17 kinesin family member 17

207713_s_at 7.08 7.44 -1.28 0.049 0.9011 RBCK1 RanBP-type and C3HC4-type zinc finger containing 1

219229_at 4.62 4.97 -1.28 0.0491 0.9011 SLCO3A1 "solute carrier organic anion transporter family, member 3A1"

222076_at 3.28 3.64 -1.28 0.0492 0.9011 HBEGF heparin-binding EGF-like growth factor

210859_x_at 6.78 7.13 -1.28 0.05 0.9011 CLN3 "ceroid-lipofuscinosis, neuronal 3"

203726_s_at 2.75 3.11 -1.29 0.0107 0.9011 LAMA3 "laminin, alpha 3"

219051_x_at 6.62 6.99 -1.29 0.0157 0.9011 METRN "meteorin, glial cell differentiation regulator"

212647_at 7.82 8.19 -1.29 0.0165 0.9011 RRAS related RAS viral (r-ras) oncogene homolog

203309_s_at 5.28 5.65 -1.29 0.0165 0.9011 HPS1 Hermansky-Pudlak syndrome 1

210775_x_at 5.26 5.63 -1.29 0.0186 0.9011 CASP9 caspase 9

218045_x_at 6.21 6.58 -1.29 0.0192 0.9011 PTMS parathymosin

209462_at 5.27 5.64 -1.29 0.0214 0.9011 APLP1 amyloid beta (A4) precursor-like protein 1

219249_s_at 6.48 6.84 -1.29 0.0217 0.9011 FKBP10 FK506 binding protein 10

219520_s_at 5.61 5.98 -1.29 0.0226 0.9011 WWC3 WWC family member 3

200656_s_at 8.3 8.66 -1.29 0.0235 0.9011 P4HB "prolyl 4-hydroxylase, beta polypeptide"

214367_at 3.78 4.14 -1.29 0.0323 0.9011 RASGRP2 RAS guanyl releasing protein 2 (calcium and DAG-regulated)

214341_at 3.47 3.84 -1.29 0.034 0.9011 AP1G2 "adaptor-related protein complex 1, gamma 2 subunit"

202186_x_at 2.85 3.21 -1.29 0.0384 0.9011 PPP2R5A "protein phosphatase 2, regulatory subunit B', alpha"

209500_x_at 5.43 5.8 -1.29 0.0462 0.9011 TNFSF12-TNFSF13; TNFSF13 "TNFSF12-TNFSF13 readthrough; tumor necrosis factor (ligand) superfamily, member 13"

205396_at 4.32 4.7 -1.3 0.0073 0.9011 SMAD3 SMAD family member 3

210628_x_at 4.23 4.61 -1.3 0.0095 0.9011 LTBP4 latent transforming growth factor beta binding protein 4

215319_at 3.21 3.59 -1.3 0.0097 0.9011 ATP8B3 "ATPase, aminophospholipid transporter, class I, type 8B, member 3"

222025_s_at 4.38 4.76 -1.3 0.0099 0.9011 OPLAH 5-oxoprolinase (ATP-hydrolysing)

213078_x_at 4.41 4.78 -1.3 0.0165 0.9011 LPCAT4 lysophosphatidylcholine acyltransferase 4

219577_s_at 4.22 4.6 -1.3 0.0219 0.9011 ABCA7 ATP binding cassette subfamily A member 7

211621_at 2.41 2.79 -1.3 0.0225 0.9011 AR androgen receptor

202756_s_at 7.46 7.84 -1.3 0.027 0.9011 GPC1 glypican 1

215682_at 4.27 4.65 -1.3 0.0295 0.9011 LOC440792 proline dehydrogenase (oxidase) 1 pseudogene

218044_x_at 3.45 3.83 -1.3 0.0357 0.9011 PTMS parathymosin

210345_s_at 3.82 4.21 -1.3 0.0374 0.9011 DNAH9 "dynein, axonemal, heavy chain 9"

205314_x_at 2.99 3.37 -1.3 0.0432 0.9011 SNTB2 "syntrophin, beta 2 (dystrophin-associated protein A1, 59kDa, basic component 2)"

205057_s_at 3.34 3.71 -1.3 0.0479 0.9011 IDUA "iduronidase, alpha-L-"

220628_s_at 5.45 5.84 -1.31 0.0155 0.9011 SDK2 sidekick cell adhesion molecule 2

206352_s_at 6.58 6.97 -1.31 0.0288 0.9011 PEX10 peroxisomal biogenesis factor 10

203500_at 5.56 5.96 -1.31 0.0323 0.9011 GCDH glutaryl-CoA dehydrogenase

202027_at 6.96 7.35 -1.31 0.0418 0.9011 TMEM184B transmembrane protein 184B

202671_s_at 7.98 8.37 -1.31 0.0462 0.9011 LOC105372824; PDXK "uncharacterized protein C21orf124; pyridoxal (pyridoxine, vitamin B6) kinase"

206987_x_at 3.88 4.26 -1.31 0.0472 0.9011 FGF18 fibroblast growth factor 18

211893_x_at 3.14 3.54 -1.31 0.0476 0.9011 CD6 CD6 molecule

210448_s_at 4.25 4.63 -1.31 0.0481 0.9011 P2RX5 "purinergic receptor P2X, ligand gated ion channel, 5"

204043_at 4.73 5.13 -1.32 0.0095 0.9011 TCN2 transcobalamin II

217284_x_at 4.69 5.09 -1.32 0.0128 0.9011 SERHL2 serine hydrolase-like 2

218175_at 8.16 8.56 -1.32 0.0186 0.9011 CCDC92 coiled-coil domain containing 92

204541_at 3.93 4.33 -1.32 0.0333 0.9011 SEC14L2 SEC14-like lipid binding 2

200710_at 7.76 8.16 -1.32 0.0429 0.9011 ACADVL "acyl-CoA dehydrogenase, very long chain"

205172_x_at 6.37 6.78 -1.33 0.004 0.9011 CLTB "clathrin, light chain B"

203317_at 4.82 5.24 -1.33 0.0099 0.9011 PSD4 pleckstrin and Sec7 domain containing 4

217007_s_at 6.19 6.6 -1.33 0.011 0.9011 ADAM15 ADAM metallopeptidase domain 15

203876_s_at 3.4 3.81 -1.33 0.0118 0.9011 MMP11 matrix metallopeptidase 11

212879_x_at 4.62 5.04 -1.33 0.0129 0.9011 PIAS4 protein inhibitor of activated STAT 4

205199_at 3.73 4.14 -1.33 0.017 0.9011 CA9 carbonic anhydrase IX

212550_at 3.66 4.07 -1.33 0.0197 0.9011 STAT5B signal transducer and activator of transcription 5B

45653_at 4.16 4.58 -1.33 0.0216 0.9011 KCTD13 potassium channel tetramerization domain containing 13

219113_x_at 5.79 6.2 -1.33 0.023 0.9011 HSD17B14 hydroxysteroid (17-beta) dehydrogenase 14

220265_at 2.5 2.91 -1.33 0.0249 0.9011 GPR107 G protein-coupled receptor 107

219188_s_at 6.32 6.73 -1.33 0.0264 0.9011 MACROD1 MACRO domain containing 1

212575_at 4.08 4.49 -1.33 0.0272 0.9011 TMEM259 transmembrane protein 259

209246_at 4.36 4.77 -1.33 0.0277 0.9011 ABCF2 ATP binding cassette subfamily F member 2

202509_s_at 3.59 4.02 -1.34 0.0041 0.9011 TNFAIP2 "tumor necrosis factor, alpha-induced protein 2"

213326_at 3 3.42 -1.34 0.0052 0.9011 VAMP1 vesicle associated membrane protein 1

210483_at 5.04 5.45 -1.34 0.0128 0.9011 LOC254896; TNFRSF10C "uncharacterized LOC254896; tumor necrosis factor receptor superfamily, member 10c, decoy without an intracellular domain"

206453_s_at 5.2 5.63 -1.34 0.0151 0.9011 NDRG2 NDRG family member 2

205330_at 6.88 7.3 -1.34 0.0209 0.9011 MN1 meningioma (disrupted in balanced translocation) 1

205125_at 7.54 7.96 -1.34 0.0234 0.9011 PLCD1 "phospholipase C, delta 1"

201565_s_at 8.78 9.2 -1.34 0.0263 0.9011 ID2 "inhibitor of DNA binding 2, dominant negative helix-loop-helix protein"

201645_at 8.04 8.46 -1.34 0.0282 0.9011 TNC tenascin C

222212_s_at 8.34 8.76 -1.34 0.0287 0.9011 CERS2 ceramide synthase 2

219047_s_at 5.83 6.26 -1.34 0.0319 0.9011 ZNF668 zinc finger protein 668

209968_s_at 4.6 5.02 -1.34 0.038 0.9011 NCAM1 neural cell adhesion molecule 1

208851_s_at 8.98 9.41 -1.35 0.0057 0.9011 THY1 Thy-1 cell surface antigen

204462_s_at 5.58 6.01 -1.35 0.0083 0.9011 SLC16A2 "solute carrier family 16, member 2 (thyroid hormone transporter)"

214215_s_at 3.48 3.91 -1.35 0.0143 0.9011 LARP4B "La ribonucleoprotein domain family, member 4B"

209253_at 7.61 8.04 -1.35 0.0197 0.9011 SORBS3 sorbin and SH3 domain containing 3

201655_s_at 7.2 7.63 -1.35 0.0207 0.9011 HSPG2 heparan sulfate proteoglycan 2

211431_s_at 4.37 4.8 -1.35 0.0299 0.9011 TYRO3 TYRO3 protein tyrosine kinase

207826_s_at 8.84 9.27 -1.35 0.0321 0.9011 ID3 "inhibitor of DNA binding 3, dominant negative helix-loop-helix protein"

205461_at 3.36 3.78 -1.35 0.0384 0.9011 RAB35 "RAB35, member RAS oncogene family"

211143_x_at 4.72 5.15 -1.35 0.0439 0.9011 NR4A1 "nuclear receptor subfamily 4, group A, member 1"

215205_x_at 4.62 5.06 -1.36 0.0062 0.9011 NCOR2 nuclear receptor corepressor 2

202545_at 5.17 5.62 -1.36 0.0092 0.9011 PRKCD "protein kinase C, delta"

215690_x_at 8.44 8.88 -1.36 0.0163 0.9011 GPAA1 glycosylphosphatidylinositol anchor attachment 1

32811_at 7.71 8.16 -1.36 0.0166 0.9011 MYO1C myosin IC

202248_at 5.13 5.57 -1.36 0.0167 0.9011 E2F4 "E2F transcription factor 4, p107/p130-binding"

218825_at 5.46 5.91 -1.36 0.0193 0.9011 EGFL7 "EGF-like-domain, multiple 7"

204657_s_at 5.96 6.4 -1.36 0.028 0.9011 SHB Src homology 2 domain containing adaptor protein B

213512_at 5.68 6.12 -1.36 0.0328 0.9011 C14orf79 chromosome 14 open reading frame 79

220605_s_at 7.62 8.06 -1.36 0.0384 0.9011 SIRT2 sirtuin 2

205026_at 4.03 4.48 -1.36 0.0425 0.9011 STAT5B signal transducer and activator of transcription 5B

204936_at 4.28 4.73 -1.36 0.0426 0.9011 MAP4K2 mitogen-activated protein kinase kinase kinase kinase 2

201276_at 5.73 6.17 -1.36 0.0464 0.9011 RAB5B "RAB5B, member RAS oncogene family"

202320_at 6.43 6.88 -1.37 0.0073 0.9011 GTF3C1 general transcription factor IIIC subunit 1

216789_at 4.28 4.73 -1.37 0.0084 0.9011 TMEM92-AS1 TMEM92 antisense RNA 1

211269_s_at 3.17 3.62 -1.37 0.0148 0.9011 IL2RA "interleukin 2 receptor, alpha"

206857_s_at 4.57 5.03 -1.37 0.0156 0.9011 FKBP1B FK506 binding protein 1B

208230_s_at 4.9 5.36 -1.37 0.016 0.9011 NRG1 neuregulin 1

221709_s_at 3.68 4.13 -1.37 0.0168 0.9011 ZNF839 zinc finger protein 839

201350_at 6.67 7.12 -1.37 0.0195 0.9011 FLOT2 flotillin 2

205398_s_at 7.17 7.62 -1.37 0.0208 0.9011 SMAD3 SMAD family member 3

219165_at 8.58 9.04 -1.37 0.0231 0.9011 PDLIM2 PDZ and LIM domain 2 (mystique)

206236_at 3.5 3.96 -1.37 0.0292 0.9011 GPR4 G protein-coupled receptor 4

209359_x_at 4.14 4.6 -1.37 0.031 0.9011 LOC100506403; RUNX1 uncharacterized LOC100506403; runt-related transcription factor 1

214184_at 2.87 3.33 -1.37 0.0322 0.9011 NPFF neuropeptide FF-amide peptide precursor

212705_x_at 6 6.45 -1.37 0.0355 0.9011 PNPLA2 patatin-like phospholipase domain containing 2

214270_s_at 4.19 4.65 -1.37 0.0415 0.9011 MAPRE3 "microtubule-associated protein, RP/EB family, member 3"

205109_s_at 3.62 4.09 -1.38 0.0027 0.9011 ARHGEF4 Rho guanine nucleotide exchange factor 4

218548_x_at 6.69 7.15 -1.38 0.0061 0.9011 TEX264 testis expressed 264

201937_s_at 6.85 7.31 -1.38 0.0064 0.9011 DNPEP aspartyl aminopeptidase

208129_x_at 4.21 4.68 -1.38 0.0183 0.9011 LOC100506403; RUNX1 uncharacterized LOC100506403; runt-related transcription factor 1

203682_s_at 5.35 5.81 -1.38 0.0235 0.9011 IVD isovaleryl-CoA dehydrogenase

206402_s_at 4.35 4.81 -1.38 0.0247 0.9011 NPFF neuropeptide FF-amide peptide precursor

207159_x_at 5.24 5.7 -1.38 0.0253 0.9011 CRTC1 CREB regulated transcription coactivator 1

213280_at 4.54 5.01 -1.38 0.0387 0.9011 RAP1GAP2 RAP1 GTPase activating protein 2

204922_at 4.59 5.07 -1.39 0.013 0.9011 C11orf80 chromosome 11 open reading frame 80

221655_x_at 4.2 4.67 -1.39 0.0161 0.9011 EPS8L1 EPS8-like 1

221519_at 5.65 6.13 -1.39 0.0172 0.9011 FBXW4 F-box and WD repeat domain containing 4

203222_s_at 3.44 3.92 -1.39 0.0173 0.9011 TLE1 "transducin-like enhancer of split 1 (E(sp1) homolog, Drosophila)"

204636_at 4.04 4.51 -1.39 0.0199 0.9011 COL17A1 "collagen, type XVII, alpha 1"

214978_s_at 2.96 3.43 -1.39 0.0205 0.9011 PPFIA4 "protein tyrosine phosphatase, receptor type, f polypeptide (PTPRF), interacting protein (liprin), alpha 4"

209438_at 4.38 4.86 -1.39 0.0304 0.9011 PHKA2 "phosphorylase kinase, alpha 2 (liver)"

211019_s_at 4.36 4.83 -1.39 0.033 0.9011 LSS "lanosterol synthase (2,3-oxidosqualene-lanosterol cyclase)"

204022_at 6.35 6.83 -1.39 0.0358 0.9011 WWP2 WW domain containing E3 ubiquitin protein ligase 2

203700_s_at 3.83 4.3 -1.39 0.0371 0.9011 DIO2 "deiodinase, iodothyronine, type II"

209407_s_at 5.51 5.98 -1.39 0.0393 0.9011 DEAF1 DEAF1 transcription factor

216610_at 2.42 2.9 -1.39 0.0411 0.9011 SLC6A2 "solute carrier family 6 (neurotransmitter transporter), member 2"

202201_at 8.06 8.53 -1.39 0.0478 0.9011 BLVRB biliverdin reductase B

212259_s_at 5.22 5.7 -1.4 0.0119 0.9011 PBXIP1 pre-B-cell leukemia homeobox interacting protein 1

214755_at 5.45 5.94 -1.4 0.0258 0.9011 UAP1L1 UDP-N-acetylglucosamine pyrophosphorylase 1 like 1

219278_at 5.52 6 -1.4 0.0406 0.9011 MAP3K6 mitogen-activated protein kinase kinase kinase 6

203507_at 3.66 4.14 -1.4 0.043 0.9011 CD68 CD68 molecule

220753_s_at 5.29 5.78 -1.4 0.0484 0.9011 CRYL1 crystallin lambda 1

219136_s_at 5.22 5.71 -1.41 0.0079 0.9011 LMF1 lipase maturation factor 1

203733_at 6.75 7.25 -1.41 0.0147 0.9011 DEXI Dexi homolog (mouse)

221333_at 4.37 4.87 -1.41 0.0231 0.9011 FOXP3 forkhead box P3

215144_at 2.79 3.28 -1.41 0.0248 0.9011

219807_x_at 6.13 6.62 -1.41 0.0267 0.9011 MIA-RAB4B; RAB4B "MIA-RAB4B readthrough (NMD candidate); RAB4B, member RAS oncogene family"

202196_s_at 7.17 7.66 -1.41 0.0286 0.9011 DKK3 dickkopf WNT signaling pathway inhibitor 3

220182_at 4.88 5.38 -1.41 0.0362 0.9011 SLC25A23 "solute carrier family 25 (mitochondrial carrier; phosphate carrier), member 23"

213264_at 5.56 6.05 -1.41 0.0444 0.9011 PCBP2 poly(rC) binding protein 2

208258_s_at 4.26 4.75 -1.41 0.0458 0.9011 GAS2L1 growth arrest-specific 2 like 1

202260_s_at 6.58 7.09 -1.42 0.0077 0.9011 STXBP1 syntaxin binding protein 1

214203_s_at 3.78 4.29 -1.42 0.0126 0.9011 LOC102724788; PRODH "proline dehydrogenase 1, mitochondrial; proline dehydrogenase (oxidase) 1"

203669_s_at 6.47 6.98 -1.42 0.0165 0.9011 DGAT1 diacylglycerol O-acyltransferase 1

218821_at 5.18 5.69 -1.42 0.0196 0.9011 NPEPL1; STX16-NPEPL1 aminopeptidase-like 1; STX16-NPEPL1 readthrough (NMD candidate)

209641_s_at 6 6.51 -1.42 0.0293 0.9011 ABCC3 ATP binding cassette subfamily C member 3

204148_s_at 4.45 4.95 -1.42 0.031 0.9011 POMZP3; ZP3 POM121 and ZP3 fusion; zona pellucida glycoprotein 3 (sperm receptor)

201255_x_at 8.34 8.85 -1.42 0.0408 0.9011 BAG6 BCL2 associated athanogene 6

218756_s_at 4.03 4.54 -1.43 0.0086 0.9011 DHRS11 dehydrogenase/reductase (SDR family) member 11

218529_at 6.64 7.16 -1.43 0.0097 0.9011 CD320 CD320 molecule

210269_s_at 3.4 3.92 -1.43 0.0165 0.9011 AKAP17A A kinase (PRKA) anchor protein 17A

207788_s_at 5.98 6.49 -1.43 0.0186 0.9011 SORBS3 sorbin and SH3 domain containing 3

216230_x_at 4.8 5.32 -1.43 0.0274 0.9011 SMPD1 "sphingomyelin phosphodiesterase 1, acid lysosomal"

213986_s_at 4.48 5 -1.43 0.0474 0.9011 TMEM259 transmembrane protein 259

214619_at 4.52 5.04 -1.44 0.0125 0.9011 CRHR1; MGC57346-CRHR1 corticotropin releasing hormone receptor 1; MGC57346-CRHR1 readthrough

212974_at 4 4.53 -1.44 0.0156 0.9011 DENND3 DENN/MADD domain containing 3

209578_s_at 6.31 6.83 -1.44 0.0307 0.9011 POFUT2 protein O-fucosyltransferase 2

205293_x_at 5.08 5.61 -1.44 0.0326 0.9011 BAIAP2 BAI1-associated protein 2

221666_s_at 6.95 7.48 -1.44 0.0378 0.9011 PYCARD PYD and CARD domain containing

211728_s_at 3.9 4.42 -1.44 0.0387 0.9011 HYAL3 hyaluronoglucosaminidase 3

218086_at 6.84 7.37 -1.44 0.0405 0.9011 NPDC1 "neural proliferation, differentiation and control, 1"

217223_s_at 5.14 5.66 -1.44 0.0494 0.9011 BCR breakpoint cluster region

204428_s_at 3.35 3.88 -1.45 0.0327 0.9011 LCAT lecithin-cholesterol acyltransferase

214667_s_at 3.11 3.65 -1.45 0.0409 0.9011 TP53I11 tumor protein p53 inducible protein 11

206397_x_at 3.85 4.39 -1.45 0.0431 0.9011 CERS1; GDF1 ceramide synthase 1; growth differentiation factor 1

213641_at 3.58 4.12 -1.45 0.0457 0.9011 ZNF500 zinc finger protein 500

204029_at 3.44 3.99 -1.46 0.0026 0.9011 CELSR2 "cadherin, EGF LAG seven-pass G-type receptor 2"

211899_s_at 3.66 4.2 -1.46 0.0069 0.9011 TRAF4 TNF receptor-associated factor 4

336_at 5.87 6.42 -1.46 0.0222 0.9011 TBXA2R thromboxane A2 receptor

206981_at 3.23 3.78 -1.46 0.0354 0.9011 SCN4A "sodium channel, voltage gated, type IV alpha subunit"

209457_at 5.67 6.22 -1.47 0.014 0.9011 DUSP5 dual specificity phosphatase 5

207765_s_at 5.4 5.96 -1.47 0.0328 0.9011 FAM214B "family with sequence similarity 214, member B"

221267_s_at 8.52 9.07 -1.47 0.0362 0.9011 ABHD17A abhydrolase domain containing 17A

203379_at 4.59 5.15 -1.47 0.0464 0.9011 RPS6KA1 "ribosomal protein S6 kinase, 90kDa, polypeptide 1"

219088_s_at 5.79 6.34 -1.47 0.0499 0.9011 ZNF576 zinc finger protein 576

203148_s_at 4.94 5.5 -1.48 0.0127 0.9011 TRIM14 tripartite motif containing 14

218018_at 7.06 7.63 -1.48 0.0318 0.9011 LOC105372824; PDXK "uncharacterized protein C21orf124; pyridoxal (pyridoxine, vitamin B6) kinase"

203256_at 3.66 4.23 -1.49 0.0077 0.9011 CDH3 "cadherin 3, type 1, P-cadherin (placental)"

221000_s_at 4.36 4.94 -1.49 0.01 0.9011 KAZALD1 Kazal-type serine peptidase inhibitor domain 1

209417_s_at 6.71 7.29 -1.49 0.0388 0.9011 IFI35 interferon-induced protein 35

218524_at 4.49 5.06 -1.49 0.0412 0.9011 E4F1 E4F transcription factor 1

221270_s_at 6.08 6.65 -1.49 0.048 0.9011 QTRT1 queuine tRNA-ribosyltransferase 1

214656_x_at 7.3 7.89 -1.5 0.0041 0.9011 MYO1C myosin IC

204248_at 6.08 6.66 -1.5 0.034 0.9011 GNA11 "guanine nucleotide binding protein (G protein), alpha 11 (Gq class)"

216439_at 4.38 4.97 -1.5 0.0364 0.9011 TNK2 "tyrosine kinase, non-receptor, 2"

212574_x_at 5.11 5.7 -1.51 0.0223 0.9011 TMEM259 transmembrane protein 259

204464_s_at 3.36 3.96 -1.51 0.026 0.9011 EDNRA endothelin receptor type A

203886_s_at 4.61 5.21 -1.52 0.0072 0.9011 FBLN2 fibulin 2

213263_s_at 6.32 6.93 -1.52 0.0252 0.9011 PCBP2 poly(rC) binding protein 2

209037_s_at 5.51 6.11 -1.52 0.0353 0.9011 EHD1 EH domain containing 1

201050_at 6.06 6.67 -1.52 0.0461 0.9011 PLD3 "phospholipase D family, member 3"

221747_at 5.8 6.42 -1.53 0.0216 0.9011 TNS1 tensin 1

221882_s_at 6.43 7.04 -1.53 0.0289 0.9011 TMEM8A transmembrane protein 8A

204188_s_at 6.48 7.09 -1.53 0.0397 0.9011 RARG "retinoic acid receptor, gamma"

214175_x_at 6.84 7.45 -1.53 0.0434 0.9011 PDLIM4 PDZ and LIM domain 4

215489_x_at 7 7.62 -1.54 0.0119 0.9011 HOMER3 homer scaffolding protein 3

220565_at 3.31 3.94 -1.55 0.012 0.9011 CCR10 chemokine (C-C motif) receptor 10

1861_at 5.34 5.97 -1.55 0.0301 0.9011 BAD BCL2-associated agonist of cell death

218417_s_at 4.17 4.81 -1.56 0.0043 0.9011 SLC48A1 "solute carrier family 48 (heme transporter), member 1"

203628_at 3.64 4.29 -1.56 0.0068 0.9011 IGF1R insulin-like growth factor 1 receptor

220588_at 4.77 5.4 -1.56 0.0359 0.9011 BCAS4 breast carcinoma amplified sequence 4

218019_s_at 7.1 7.75 -1.57 0.0049 0.9011 LOC105372824; PDXK "uncharacterized protein C21orf124; pyridoxal (pyridoxine, vitamin B6) kinase"

206027_at 5.71 6.36 -1.57 0.0194 0.9011 S100A3 S100 calcium binding protein A3

204854_at 5.34 6 -1.57 0.0298 0.9011 P3H3 prolyl 3-hydroxylase 3

207510_at 4.67 5.33 -1.58 0.0012 0.9011 BDKRB1 bradykinin receptor B1

216264_s_at 6.01 6.67 -1.58 0.0144 0.9011 LAMB2 "laminin, beta 2 (laminin S)"

202793_at 4.75 5.43 -1.6 0.008 0.9011 LPCAT3 lysophosphatidylcholine acyltransferase 3

211029_x_at 3.95 4.62 -1.6 0.0205 0.9011 FGF18 fibroblast growth factor 18

204867_at 4.17 4.85 -1.6 0.0257 0.9011 GCHFR GTP cyclohydrolase I feedback regulator

219845_at 5.98 6.66 -1.6 0.0257 0.9011 BARX1 BARX homeobox 1

40837_at 5.69 6.39 -1.62 0.016 0.9011 TLE2 transducin-like enhancer of split 2

203623_at 5.08 5.77 -1.62 0.0223 0.9011 PLXNA3 plexin A3

202898_at 4.77 5.48 -1.63 0.0113 0.9011 SDC3 syndecan 3

219305_x_at 5.35 6.05 -1.63 0.0401 0.9011 FBXO2 F-box protein 2

215037_s_at 5.96 6.68 -1.64 0.0204 0.9011 BCL2L1 BCL2-like 1

205691_at 3.1 3.82 -1.64 0.0215 0.9011 SYNGR3 synaptogyrin 3

221953_s_at 6.57 7.28 -1.64 0.0367 0.9011 MMP24-AS1 MMP24 antisense RNA 1

207993_s_at 4.26 4.97 -1.64 0.0459 0.9011 CHP1 calcineurin-like EF-hand protein 1

212968_at 6.6 7.33 -1.65 0.0055 0.9011 RFNG RFNG O-fucosylpeptide 3-beta-N-acetylglucosaminyltransferase

204698_at 5.01 5.73 -1.65 0.0447 0.9011 ISG20 interferon stimulated exonuclease gene 20kDa

202017_at 8.73 9.48 -1.68 0.0281 0.9011 EPHX1 "epoxide hydrolase 1, microsomal (xenobiotic)"

202219_at 6.08 6.85 -1.7 0.0331 0.9011 SLC6A8 "solute carrier family 6 (neurotransmitter transporter), member 8"

221892_at 5 5.76 -1.7 0.0453 0.9011 H6PD hexose-6-phosphate dehydrogenase (glucose 1-dehydrogenase)

219044_at 5.99 6.78 -1.73 0.0334 0.9011 THNSL2 threonine synthase-like 2

201149_s_at 6.62 7.41 -1.74 0.0296 0.9011 TIMP3 TIMP metallopeptidase inhibitor 3

206139_at 5.49 6.29 -1.74 0.0375 0.9011 PI4KB "phosphatidylinositol 4-kinase, catalytic, beta"

217478_s_at 3.62 4.42 -1.74 0.0458 0.9011 HLA-DMA "major histocompatibility complex, class II, DM alpha"

1007_s_at 7.2 8.01 -1.76 0.0488 0.9011 DDR1 discoidin domain receptor tyrosine kinase 1

217991_x_at 4.97 5.79 -1.77 0.0266 0.9011 SSBP3 single stranded DNA binding protein 3

221551_x_at 6.69 7.52 -1.78 0.0244 0.9011 ST6GALNAC4 "ST6 (alpha-N-acetyl-neuraminyl-2,3-beta-galactosyl-1,3)-N-acetylgalactosaminide alpha-2,6-sialyltransferase 4"

208937_s_at 9.14 9.97 -1.78 0.029 0.9011 ID1 "inhibitor of DNA binding 1, dominant negative helix-loop-helix protein"

33322_i_at 8.07 8.9 -1.78 0.0364 0.9011 SFN stratifin

211320_s_at 4.72 5.57 -1.81 0.0485 0.9011 PTPRU "protein tyrosine phosphatase, receptor type, U"

219354_at 4.88 5.75 -1.82 0.0441 0.9011 KLHL26 kelch-like family member 26

205014_at 5.61 6.49 -1.83 0.045 0.9011 FGFBP1 fibroblast growth factor binding protein 1

201502_s_at 7.98 8.89 -1.88 0.0391 0.9011 NFKBIA "nuclear factor of kappa light polypeptide gene enhancer in B-cells inhibitor, alpha"

206655_s_at 4.79 5.72 -1.9 0.0118 0.9011 GP1BB; SEPT5; SEPT5-GP1BB "glycoprotein Ib (platelet), beta polypeptide; septin 5; SEPT5-GP1BB readthrough"

214297_at 5.47 6.41 -1.91 0.0091 0.9011 CSPG4 chondroitin sulfate proteoglycan 4

202804_at 7.76 8.71 -1.93 0.0425 0.9011 ABCC1 ATP binding cassette subfamily C member 1

203851_at 8.97 9.94 -1.95 0.0066 0.9011 IGFBP6 insulin like growth factor binding protein 6

215938_s_at 4.01 4.97 -1.95 0.0342 0.9011 PLA2G6 "phospholipase A2, group VI (cytosolic, calcium-independent)"

202805_s_at 5.91 6.91 -2 0.002 0.9011 ABCC1 ATP binding cassette subfamily C member 1

209293_x_at 4.98 5.98 -2.01 0.015 0.9011 ID4 "inhibitor of DNA binding 4, dominant negative helix-loop-helix protein"

206665_s_at 4.58 5.61 -2.03 0.0496 0.9011 BCL2L1 BCL2-like 1

207147_at 4.38 5.46 -2.11 0.0152 0.9011 DLX2 distal-less homeobox 2

219480_at 5.83 6.92 -2.12 0.043 0.9011 SNAI1 snail family zinc finger 1

204379_s_at 4.52 5.66 -2.2 0.0086 0.9011 FGFR3 fibroblast growth factor receptor 3

211385_x_at 4.96 6.11 -2.23 0.0237 0.9011 SULT1A2 sulfotransferase family 1A member 2

207169_x_at 6.33 7.49 -2.24 0.0258 0.9011 DDR1 discoidin domain receptor tyrosine kinase 1

220937_s_at 7.05 8.24 -2.29 0.0462 0.9011 ST6GALNAC4 "ST6 (alpha-N-acetyl-neuraminyl-2,3-beta-galactosyl-1,3)-N-acetylgalactosaminide alpha-2,6-sialyltransferase 4"

209981_at 5.19 6.45 -2.4 0.0499 0.9011 CSDC2 "cold shock domain containing C2, RNA binding"

219298_at 4.51 5.81 -2.45 0.0034 0.9011 ECHDC3 enoyl-CoA hydratase domain containing 3

204736_s_at 6.15 7.45 -2.45 0.0041 0.9011 CSPG4 chondroitin sulfate proteoglycan 4

201650_at 3.6 4.9 -2.48 0.0487 0.9011 KRT19 "keratin 19, type I"

218537_at 7.23 8.69 -2.75 0.0365 0.9011 HCFC1R1 host cell factor C1 regulator 1 (XPO1 dependent)

219529_at 6.81 8.65 -3.57 0.0243 0.9011 CLIC3 chloride intracellular channel 3

218380_at 6.33 8.37 -4.1 0.0063 0.9011 LOC728392 uncharacterized LOC728392

#%result_name=GSE34095

#%array_type=HG-U133A

#%annotation=HG-U133A.na36.annot.csv

#%comparison=IDD2 vs CON2
